# Proteomics analysis of bladder cancer invasion: Targeting EIF3D for therapeutic intervention

**DOI:** 10.18632/oncotarget.17279

**Published:** 2017-04-20

**Authors:** Agnieszka Latosinska, Marika Mokou, Manousos Makridakis, William Mullen, Jerome Zoidakis, Vasiliki Lygirou, Maria Frantzi, Ioannis Katafigiotis, Konstantinos Stravodimos, Marie C. Hupe, Maciej Dobrzynski, Walter Kolch, Axel S. Merseburger, Harald Mischak, Maria G. Roubelakis, Antonia Vlahou

**Affiliations:** ^1^ Biotechnology Division, Biomedical Research Foundation, Academy of Athens, Athens, Greece; ^2^ Mosaiques Diagnostics GmbH, Hannover, Germany; ^3^ Laboratory of Biology, Department of Basic Medical Sciences, National and Kapodistrian University of Athens, School of Medicine, Athens, Greece; ^4^ BHF Glasgow Cardiovascular Research Centre, University of Glasgow, Glasgow, United Kingdom; ^5^ Department of Urology, Medical School of Athens, Laikon Hospital, Athens, Greece; ^6^ Department of Urology, Campus Lübeck, University Hospital Schleswig-Holstein, Lübeck, Germany; ^7^ Systems Biology Ireland, Conway Institute, and School of Medicine, University College Dublin, Belfield, Dublin, Ireland

**Keywords:** EIF3D, translation, bladder cancer, tissue proteomics, RHEB

## Abstract

Patients with advanced bladder cancer have poor outcomes, indicating a need for more efficient therapeutic approaches. This study characterizes proteomic changes underlying bladder cancer invasion aiming for the better understanding of disease pathophysiology and identification of drug targets. High resolution liquid chromatography coupled to tandem mass spectrometry analysis of tissue specimens from patients with non-muscle invasive (NMIBC, stage pTa) and muscle invasive bladder cancer (MIBC, stages pT2+) was conducted. Comparative analysis identified 144 differentially expressed proteins between analyzed groups. These included proteins previously associated with bladder cancer and also additional novel such as PGRMC1, FUCA1, BROX and PSMD12, which were further confirmed by immunohistochemistry. Pathway and interactome analysis predicted strong activation in muscle invasive bladder cancer of pathways associated with protein synthesis e.g. eIF2 and mTOR signaling. Knock-down of eukaryotic translation initiation factor 3 subunit D (EIF3D) (overexpressed in muscle invasive disease) in metastatic T24M bladder cancer cells inhibited cell proliferation, migration, and colony formation *in vitro* and decreased tumor growth in xenograft models. By contrast, knocking down GTP-binding protein Rheb (which is upstream of EIF3D) recapitulated the effects of EIF3D knockdown *in vitro*, but not *in vivo*. Collectively, this study represents a comprehensive analysis of NMIBC and MIBC providing a resource for future studies. The results highlight EIF3D as a potential therapeutic target.

## INTRODUCTION

Bladder Cancer (BC) is the second most frequently reported malignancy of the genitourinary system, with an estimate of 429,800 new cases and 165,100 deaths in 2012 [1, 2]. Based on the penetration depth into the bladder wall, 70% of the newly diagnosed tumors are classified as non-muscle invasive bladder cancer (NMIBC, stages pTa, pT1, pTis) and are treated by transurethral resection and intravesical therapies [3]; whereas the remaining 30% are categorized as muscle invasive bladder cancer (MIBC, stages pT2-4) and are treated by radical cystectomy, (neo)adjuvant chemotherapy or (chemo) radiotherapy [4–6]. Patients harboring MIBC are associated with poor outcome: when the cancer is diagnosed at a localized stage (cancer has not spread beyond the bladder wall), the 5-year survival rate is 47% for patients with muscle-invasive disease in comparison to 81% in the case of non-muscle invasive disease [7]. Considering the severity of the disease, some novel therapies are currently investigated in clinical trials, including cancer immunotherapies targeting proteins such as programmed cell death protein 1 (PD-1), cytotoxic T-Lymphocyte associated protein 4 (CTLA-4) [8]; cell cycle checkpoint inhibitors targeting Aurora kinase A [9] and signal transduction inhibitors targeting fibroblast growth factor receptors (FGFR) [10].

To date, extensive efforts have been made to characterize the molecular background of BC [11]. Early efforts in the interpretation of BC molecular profiling data recapitulated a dual-track model, which proposes that the disease develops via two distinct forms [12, 13]. The papillary NMIBCs originate from urothelial hyperplasia as a result of the alteration of the phosphatidylinositol 3-kinase (PI3K)- protein kinase B (Akt)-mTOR (PI3K-AKT-mTOR) pathway, mutations in FGFR3 and HRas proto-oncogene [14]. On the other hand, the non-papillary MIBCs are developing from flat dysplasia and carcinoma *in situ* (CIS) and are characterized by genetic alterations in tumor suppressor genes such as tumor protein p53 (TP53), cyclin dependent kinase inhibitor 2A (CDKN2A), Cyclin D1 (CCND1), cyclin dependent kinase inhibitor 1B (CDKN1B) and RB transcriptional corepressor 1 (RB1) [14]. Although, this model explains many features of BC, it does not adequately address the heterogeneity of the disease [13]. Emerging evidence from next-generation sequencing data, mainly from MIBC, indicates its high phenotypic diversity and sub-clonal cancer evolution [11, 15–20]. Consequently, the presence of distinct molecular disease subtypes have been suggested by various groups (as summarized in [19, 21]) opening up new research avenues towards better patient stratification and tailored therapy selection [22].

Investigations at the protein level are attractive, since proteins manifest the functional state of the disease-related molecular alterations and are direct targets for pharmaceutical intervention [23]. Tissue samples represent the site of cancer initiation and progression and, therefore, serve as a very appropriate biological source for studying disease-associated alterations. Currently, there is a growing number of studies exploring BC tissue specimens using proteomics techniques [24–34]. Over the past years, emphasis has been placed on investigating the differences between BC and the adjacent normal urothelial tissue or non-cancerous specimens. As a result of these studies, novel biomarkers for cancer diagnosis [e.g. stathmin 1 (STMN1), transgelin 2 (TAGLN2) [25]] or potential targets for therapeutic intervention were proposed (e.g. phosphoglycerate mutase 1 (PGAM1) [24]). Furthermore, efforts have been made towards the proteomic characterization of individual profiles of NMIBC and MIBC [27, 31, 32, 34], in the context of both cellular and stromal changes. For example, comparative proteomic analysis of non-muscle invasive cancer cells and normal urothelial cells revealed changes in pathways related to oxidative phosphorylation, focal adhesion, ribosome biogenesis, and leukocyte transendothelial migration [31]. In a follow-up study, proteomic characterization of NMIBC was performed, aiming at the investigation of cellular (purified normal urothelial cells versus non-muscle invasive cancer cells) and stromal changes (normal stromal cells versus non-muscle invasive cancer stromal cells) [27]. Alteration of several pathways was predicted including metabolic pathways, endocytosis, oxidative phosphorylation, and spliceosome function [27]. In another study, Niu et al. performed a global characterization of the stromal proteome of MIBC [32]. Pathway analysis of differentially expressed proteins between cancer and normal stromal cells indicated changes in metabolic pathways, actin cytoskeleton remodeling, adhesion, and endocytosis [32]. Changes in focal adhesion and extracellular matrix (ECM)-receptor interaction, based on analysis of stromal cells from MIBC were associated with the risk of cancer metastasis [34].

A comprehensive, high resolution, direct comparison of tissue proteomic profiles between NMIBC and MIBC has not been performed yet, to the best of our knowledge. Moreover, using the tissue adjacent to the tumor as normal control might not be an optimal experimental set up to discover what molecular changes make BC aggressive, as these areas have frequently cancer-related genetic characteristics [35]. Therefore, when aiming at the investigation of the molecular events underlying disease progression and subsequently key molecules that could also be “druggable” targets for therapeutic intervention, evaluation of tissue specimens that represent different stages of disease appears to be well justified.

The main objective of this study was the global characterization of the proteomic changes underlying BC invasion that could ultimately lead to a better understanding of disease pathophysiology and subsequent identification of biology-driven therapeutic targets. Towards that end, a comparative proteomic analysis of tissue specimens from NMIBC and MIBC was conducted. *In silico* analysis of differentially expressed proteins predicted, among others, a significant up-regulation of protein synthesis. By using *in vitro* assays and *in vivo* models, the functional relevance of eukaryotic translation initiation factor 3 subunit D (EIF3D) was evaluated in detail.

## RESULTS

### Tissue proteomic profiling

**Figure 1:**
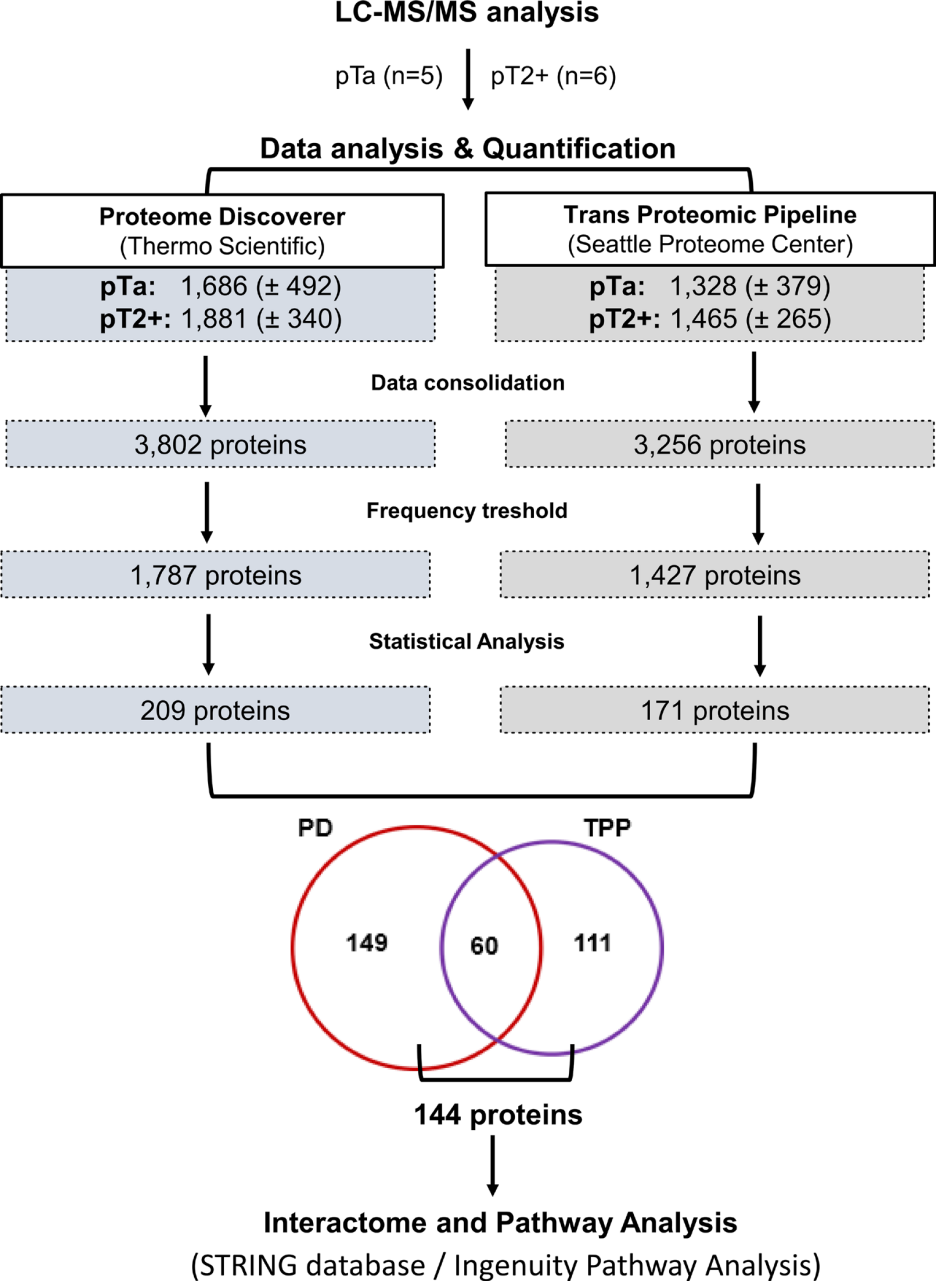
Schematic representation of the MS data analysis workflow A total of 11 bladder cancer tissue proteomic profiles were generated and analyzed using two independent approaches. This includes analysis using Proteome Discoverer and Trans Proteomic Pipeline followed by quantification based on peak area and spectral counting (i.e. APEX), respectively. Following consolidation of the individual proteomics profiles, proteins identified in at least 60% of samples of at least one group (pTa/pT2+) were considered in differential expression analysis. For those, statistical analysis was performed to identify disease-associated proteins (*p <* 0.05). A total of 60 proteins were found to be significantly altered according to both approaches. The overlap increases to 144 proteins, when considering proteins found to be differentially expressed (statistically significant level) by at least one approach and exhibiting the same regulation trend based on the other quantification approach (up/down-regulation by at least 1.5 fold). These latter 144 proteins were further analyzed by pathway and interactome approaches.

**Figure 2:**
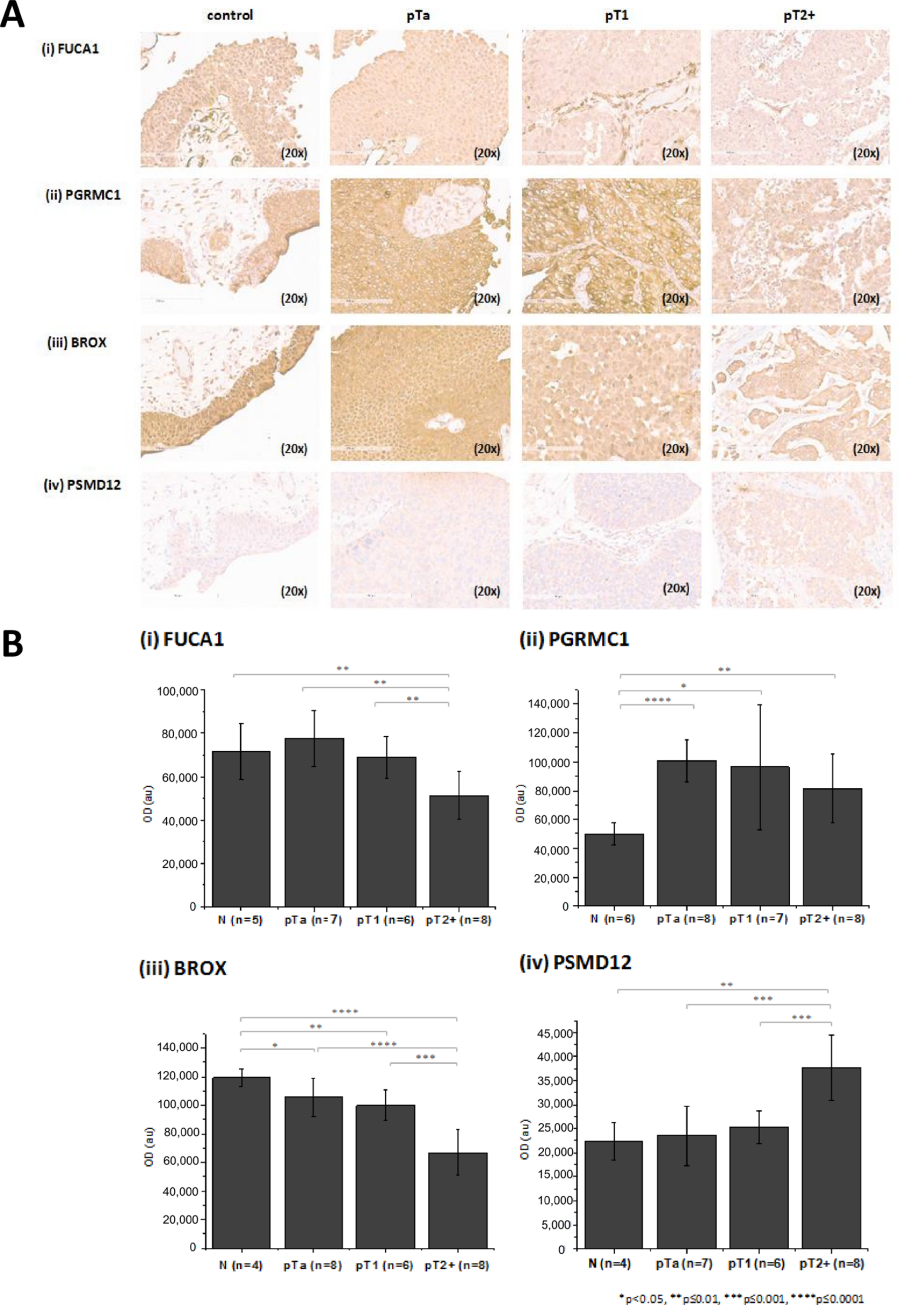
Verification of proteomics findings using immunohistochemistry (**A**) Stained sections and (**B**) quantification results for (i) FUCA 1, (ii) PGRMC1 (iii) BROX and (iv) PSMD12 from control, pTa, pT1 and pT2+ human samples are presented. Immunohistochemistry analysis was performed using tissue microarrays (total *n =* 30 including 6 controls, 8 pTa, 8 pT1, 8 pT2+). For each protein, the exact number of tissue sections included for the quantification is presented in the figure. Quantification of the staining intensity was performed using the Image J software. Mean staining intensities and standard deviations per analyzed group are presented. Statistical analysis was performed using an independent sample *t*-test.

Untargeted proteome analysis of BC tissue specimens (NMIBC; pTa, *n =* 5 versus MIBC pT2+ *n =* 6) was performed using high resolution liquid chromatography coupled to tandem mass spectrometry (LC-MS/MS) analysis in combination with two different software packages for protein identification and quantification, in order to maximize the reliability of differential protein expression analysis (workflow presented in Figure 1). As shown (Figure 1), comparable numbers of proteins were identified in the experimental groups (pTa and pT2+) by both software packages (Proteome Discoverer, PD, and Trans Proteomic Pipeline, TPP). To increase the reliability of the subsequent differential expression analysis, only proteins detected in ≥ 60% of specimens (3/5 for pTa and 4/6 for pT2+) in at least one group (pTa, pT2+) were considered further. Proteins were defined as differentially expressed between the two groups based on statistical significance (*p <* 0.05, independent sample *t*-test; Figure 1). As depicted in Figure 1, sixty proteins were statistically significant according to both approaches (PD and TPP) and were considered of high validity and reliability. Thus, for these proteins thorough literature mining was performed to identify novel proteins associated with BC invasion (listed in Supplementary Table 1). Numerous proteins previously associated with BC were part of this list including, but not limited to hydroxyprostaglandin dehydrogenase 15-(NAD) (HPGD) [36], thymidine phosphorylase (TYMP) [37], annexins (e.g. ANXA1 [38], ANXA5 [39], ANXA10 [40]), alpha actinins (ACTN) (e.g. ACTN1 [41], ACTN4 [42, 43]), membrane-associated progesterone receptor component 1 (PGRMC1) [44], transforming growth factor-beta-induced protein ig-h3 (TGFBI) [45], cadherin 13 (CDH13) [46], or cathepsin E (CTSE) [47], serving as a positive control for the performed study. In addition, proteins that have been related to other cancers but not to BC yet, were also identified, such as fibulin-2 (FBLN2), tissue alpha-L-fucosidase (FUCA1), staphylococcal nuclease domain-containing protein 1 (SND1), and EIF3D. Importantly, we also identified differentially expressed proteins not yet described in BC or other types of malignancy, such as BRO1 domain-containing protein BROX (BROX), vesicle-trafficking protein SEC22b (SEC22B), RNA 3′-terminal phosphate cyclase (RTCA) (described below in more detail). A detailed categorization of the proteomics findings with the representative references supporting their grouping is presented in Supplementary Table 1. We subsequently performed validation of the differential expression of some of the proteins that had not been previously linked to BC or BC tissue using immunohistochemistry analysis of a small, yet new (e.g. not used for the proteomic analysis) set of tissue samples. Based on appropriate antibody availability (as confirmed by using Western blot, *data not shown*), the difference in the expression level between MIBC and NMIBC could be confirmed for FUCA1, BROX, PGRMC1 and 26S proteasome non-ATPase regulatory subunit 12 (PSMD12). As shown in Figure 2, in agreement with the proteomics results, a decline in the expression levels of the former 3 proteins in invasive versus non-invasive cancers could be observed, reaching statistical significance in the case of BROX, and FUCA1, whereas a statistically significant up-regulation was observed in the case of PSMD12.

### Assessment of biological relevance of proteomics findings

To assess the biological relevance of the observed molecular changes in BC invasion and predict potentially new targets, *in silico* pathway and interactome analyses of the differentially expressed proteins was performed. To maximize coverage, the list of 60 proteins was expanded to include proteins found to be differentially expressed (statistically significant) by one quantification strategy and characterized by the same expression trend (up- or down- regulation and at least 1.5 fold change) in the second. Applying these criteria, 144 proteins were identified as differentially regulated between the two groups (Supplementary Table 2; full datasets per quantification approach are available in Supplementary Table 3). Seventy-three out of the 144 proteins were mapped to 208 pathways, 52 of which were predicted to be significantly altered (*p <* 0.05, right-tailed Fisher Exact Test) in MIBC. Pathways with at least 3 molecules assigned were prioritized according to the significance level (*p <* 0.05) and the top 20 findings are presented in Table 1. These included pathways related to protein synthesis [i.e. eukaryotic initiation factor 2 (eIF2) signaling, mammalian target of rapamycin kinase (mTOR) signaling, tRNA charging, regulation of eukaryotic initiation factor 4 (eIF4) and ribosomal protein S6 kinase beta-1 (p70S6K) signaling], endocytosis (i.e. caveolar and clathrin-mediated endocytosis signaling), cell-ECM interactions, cytoskeletal remodeling, cell adhesion (i.e. integrin signaling, actin cytoskeleton signaling, paxillin signaling, remodeling of epithelial adherens junctions, epithelial adherens junction signaling), oxidative response / xenobiotic metabolism [nuclear factor, erythroid 2 like 2 (NRF2)-mediated oxidative stress response, glutathione-mediated detoxification, aryl hydrocarbon receptor signaling], and angiogenesis [vascular endothelial growth factor (VEGF) signaling]. High consistency in the expression trend (up-/down-regulation in MIBC versus NMIBC) of molecules mapped to the pathways was most evident for pathways targeting protein synthesis including eIF2 signaling, tRNA charging, regulation of eIF4 and p70S6K, as well as mTOR signaling (Table 1). All proteins mapped to these pathways were up-regulated in MIBC, with an exception of 5′-AMP-activated protein kinase subunit gamma-2 (PRKAG2) which was down-regulated in MIBC. However, the down-regulation of PRKAG2 is expected, when the mTOR pathway is activated [48] (Supplementary Figure 1).

**Table 1:** The top 20 pathways, with at least 3 molecules assigned, predicted based on proteomics data

Pathway	*p*-value	# associated molecules	Pathway Activation (z-score)	Molecules	
				Up-regulated in MIBC	Down-regulated in MIBC
Protein synthesis-related pathways:					
EIF2 Signaling	6.9E-07	10/185	Activated (2.24)	RPL12, RPS13, EIF3D, RPS9, RPS18, RPL22, RPL31, RPS14, RPL27A, RPL10A	
tRNA Charging	2.5E-03	3/39		TARS, VARS, WARS	
Regulation of eIF4 and p70S6K Signaling	3.6E-03	5/146		RPS13, EIF3D, RPS9, RPS18, RPS14	
mTOR Signaling	2.0E-03	6/188		RPS13, EIF3D, RPS9, RPS18, RPS14	PRKAG2
Oxidative response/xenobiotic metabolism -related pathways:					
NRF2-mediated Oxidative Stress Response	5.5E-07	10/180	Activated (1.00)	EPHX1, FTL, PPIB, ACTG2	GSTM1, GSTM5, UBB, GSTM2, GSTM3, GSTM4
Glutathione-mediated Detoxification	1.9E-06	5/30			GSTM1, GSTM5, GSTM2, GSTM3, GSTM4
Aryl Hydrocarbon Receptor Signaling	5.8E-05	7/140			SRC, GSTM1, GSTM5, GSTM2, GSTM3, GSTM4, NEDD8
Endocytosis-related pathways:					
Caveolar-mediated Endocytosis Signaling	1.6E-03	4/72		COPA, COPG1, ACTG2	SRC
Clathrin-mediated Endocytosis Signaling	1.9E-03	6/185		ARPC2, AP2A1, ACTG2	UBB, SRC, CD2AP
Cell-ECM interactions, cytoskeletal remodeling, cell adhesion -related pathways:					
Remodeling of Epithelial Adherens Junctions	7.9E-06	6/68		ARPC2, ACTN2, ACTN1, ACTN4, ACTG2	SRC
Epithelial Adherens Junction Signaling	7.4E-05	7/146		ARPC2,ACTN2, MYH10, ACTN1, ACTN4, ACTG2	SRC
Integrin Signaling	8.7E-05	8/202	Activated (2.12)	ARPC2,MYLK, ACTN2, ACTN1, ACTN4, ACTG2	SRC, VASP
Actin Cytoskeleton Signaling	1.4E-04	8/217		ARPC2, GSN, MYLK, ACTN2, MYH10, ACTN1, ACTN4, ACTG2	
Paxillin Signaling	7.4E-04	5/102	Activated (1.34)	ACTN2, ACTN1, ACTN4, ACTG2	SRC
Regulation of Cellular Mechanics by Calpain Protease	6.8E-04	4/57		ACTN2, ACTN1, ACTN4	SRC
Angiogenesis -related pathways:					
VEGF Signaling	4.4E-04	5/91	Activated (1.34)	ACTN2, ACTN1, ACTN4, ACTG2	SRC
Other pathways:					
Leukocyte Extravasation Signaling	4.8E-04	7/198	Activated (1.89)	ACTN2, ACTN1, ACTN4, GNAI3, ACTG2	SRC, VASP
Germ Cell-Sertoli Cell Junction Signaling	9.1E-04	6/160		GSN, ACTN2, ACTN1, ACTN4, ACTG2	SRC
Sertoli Cell-Sertoli Cell Junction Signaling	1.5E-03	6/178		ACTN2, ACTN1, ACTN4, ACTG2	SRC, PRKAG2
Protein Ubiquitination Pathway	7.6E-05	9/255		PSMD12, PSMA7,PSMA2,PSMB2, PSMB3, UBE2D3	UBB,UCHL3, HSPE1

The findings were prioritized based on the significance level and number of associated proteins (≥ 3). The latter’s (e.g. associated proteins) trend of expression (up- or down- regulated) in MIBC versus NMIBC is indicated. Pathways were generated through the use of QIAGEN’s Ingenuity Pathway Analysis (IPA^®^, QIAGEN Redwood City, www.qiagen.com/ingenuity).

**Figure 3:**
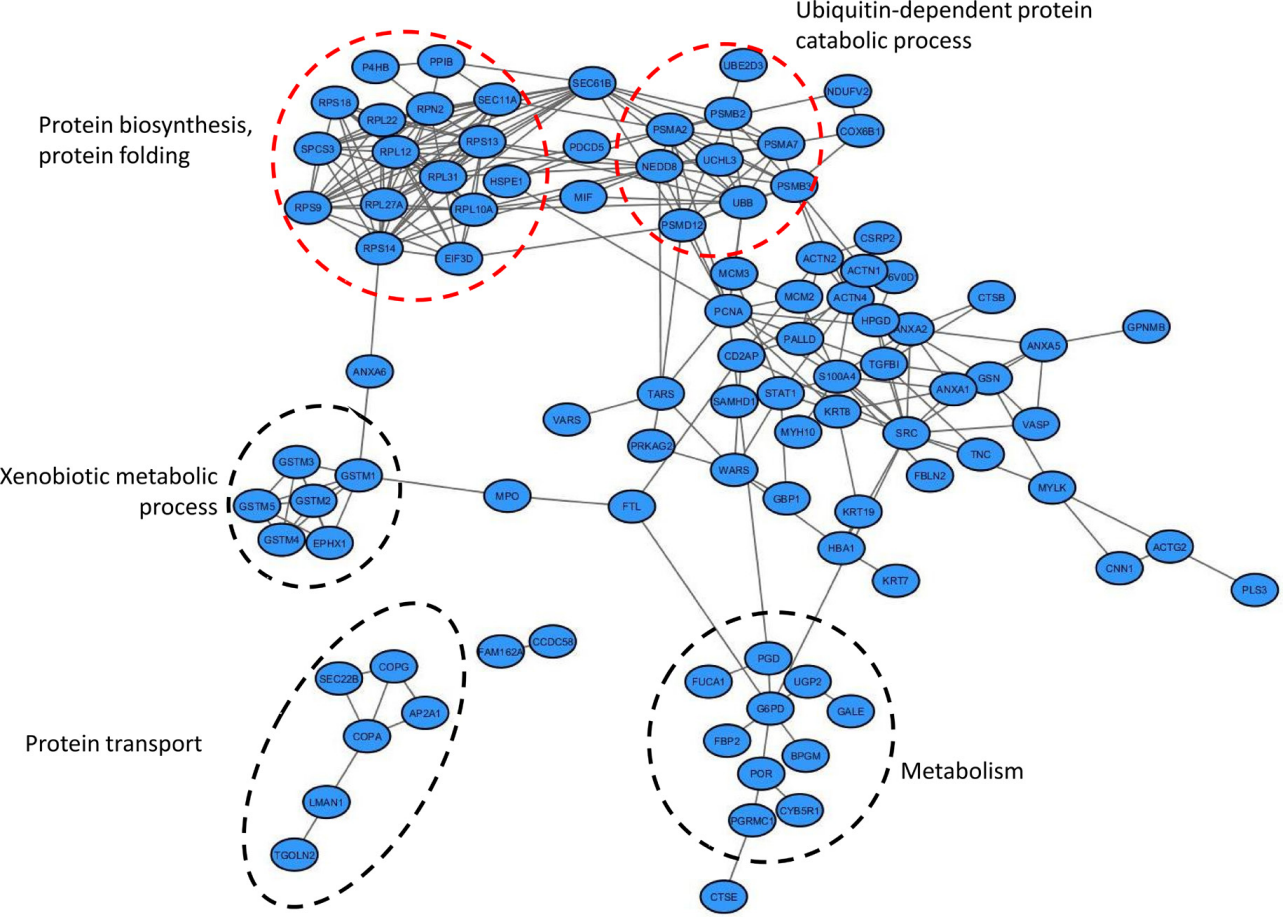
Interactome network of proteins altered during bladder cancer invasion Functional annotations of the proteins included in the interaction network was conducted using Gene Ontology Annotation (UniProt-GOA) Database [123]. Proteins involved in protein degradation/protein synthesis clusters are marked in red circles.

A parallel analysis of predicted protein-protein interactions revealed that 97 out of the aforementioned 144 proteins are involved in an interactome network (261 interactions were retrieved), as shown in Figure 3. In line with the pathway analysis, multiple proteins associated with the interactome network create functional clusters related to protein synthesis (e.g. EIF3D, numerous ribosomal subunits), protein degradation [e.g. polyubiquitin-B (UBB), enzymes such as ubiquitin carboxyl-terminal hydrolase isozyme L3 (UCHL3), ubiquitin-conjugating enzyme E2 D3 (UBE2D3), numerous subunits of proteasome], glutathione detoxification (e.g. glutathione transferases), metabolism [e.g. enzymes such as UTP--glucose-1-phosphate uridylyltransferase (UGP2), glucose-6-phosphate 1-dehydrogenase (G6PD), bisphosphoglycerate mutase (BPGM)] and endocytosis (e.g. SEC22B, subunits of coatamer, Protein ERGIC-53). Of note, many of the proteins (36 out of the 97) that were included in the interactome network were not mapped to pathways. This includes proteins that have not been previously investigated in BC tissue specimens, such as FUCA1, PGRMC1 and PSMD12 [confirmed by immunohistochemistry (IHC) as detailed above], but also trans-Golgi network integral membrane protein 2 (TGOLN2), transmembrane glycoprotein NMB (GPNMB), and TGFBI.

Collectively, the molecular processes (the aforementioned: protein synthesis, degradation, glutathione detoxification etc., shown in Table 1) were further shortlisted based on their predicted activation score (z-score using Ingenuity Pathway Analysis), significance level and agreement in the fold change direction of protein findings mapped to these pathways. Among the top 20 shortlisted pathways (based on the significance level), eIF2 signaling (ranked as 2^nd^ based on the significance level) was characterized by the highest activation z-score. Additionally, all detected proteins involved in this pathway were up-regulated in MIBC versus NMIBC. Interestingly, eIF2 signaling partially overlaps with the mTOR pathway, which is under investigation in the context of BC, with mTOR inhibitors being tested as potential targets for BC therapeutic intervention [49]. Given, in addition, the existing interest on protein synthesis as a source of promising anticancer drugs [50], we shortlisted pathways related to protein synthesis for further investigation, focusing on eIF2 and mTOR signaling. Among the proteins indicated by the tissue proteomic analysis, EIF3D, not earlier associated with BC, was overexpressed in MIBC versus NMIBC (Supplementary Figure 2) and was selected as candidate for further investigation.

### Lentivirus-mediated RNAi knockdown of EIF3D in T24M cells

**Figure 4:**
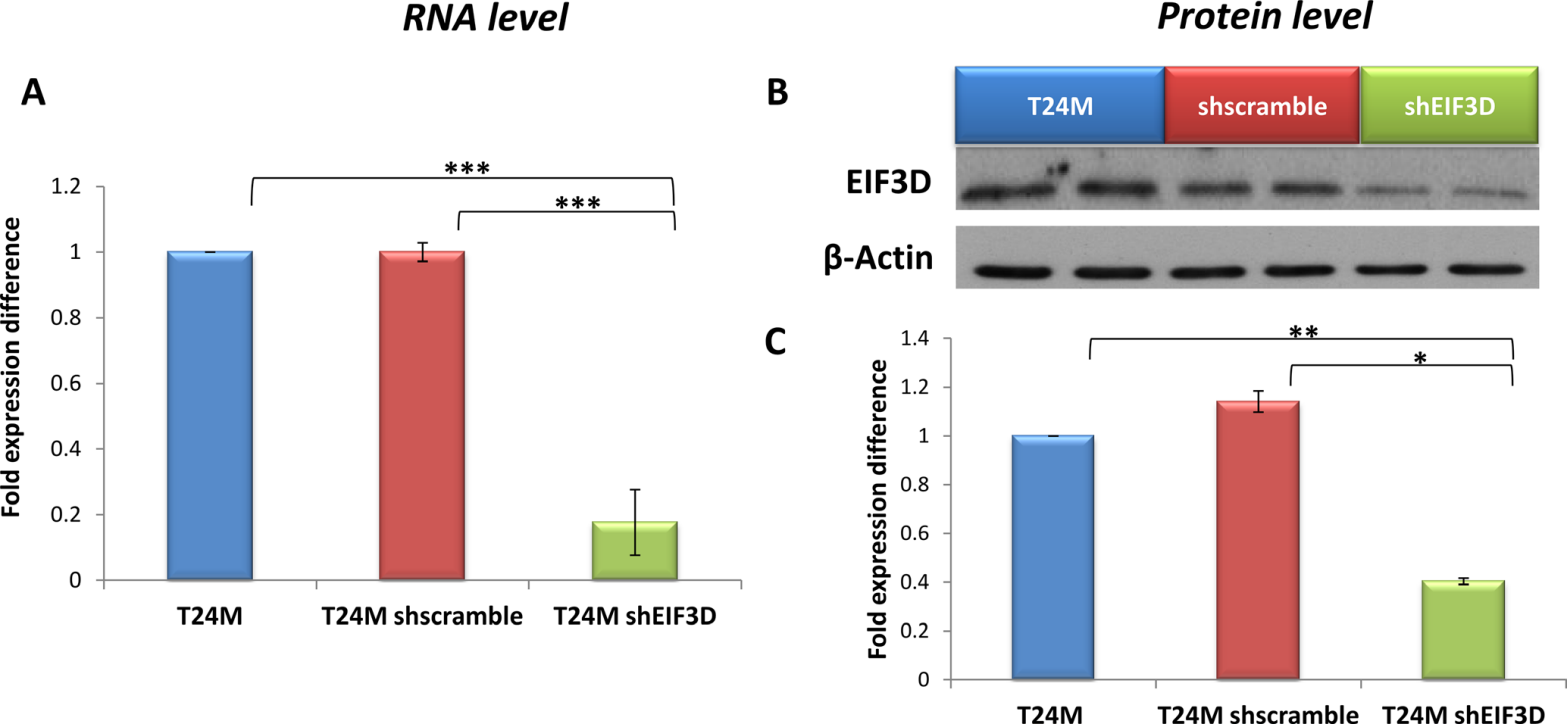
Evaluation of EIF3D knockdown in T24M cells at the RNA and protein level (**A**) Bar graph representing the downregulation of EIF3D in T24M shEIF3D cells in comparison to T24M shscramble and untransduced T24M cells analysed by real-time PCR. The data were normalized to the human GAPDH reference gene and then to the control T24M untransduced cells. (**B**) Western blot analysis for EIF3D in cell extracts derived from T24M, T24M shscramble and T24M shEIF3D cells. (**C**) Bar chart showing data from the quantification analysis of EIF3D protein bands detected in T24M, T24M shscramble and T24M shEIF3D. The quantification of the proteins was performed by using the Quantity One software (BioRad) and the results were normalized to β-Actin loading control and then to the T24M untransduced cells. The values represent the means ± SD from three independent experiments performed in duplicate (two-tailed Student’s *t*-test, *p ≤ 0.05, **p ≤ 0.01, ***p ≤ 0.001).

In order to investigate the therapeutic impact of EIF3D in BC progression, we performed its stable knockdown through lentivirus-mediated RNA interference, in the metastatic T24M BC cell line (given that enhanced expression of this protein was observed in MIBC). T24M cells were transduced with the shEIF3D lentivirus, while shscramble lentivirus-transduced and/or untransduced T24M cells were used as controls. The knockdown efficiency was assessed 4 days after the transduction at the RNA level by real-time PCR and at the protein level by Western blot analysis. As shown in Figure 4, EIF3D expression was significantly reduced at the mRNA level by 82% ± 10% in T24M shEIF3D cells compared to T24M cells shscramble (*p ≤* 0.001, Student’s *t*-test) and at the protein level by 64.6% ± 2.46%, respectively (*p ≤* 0.01, Student’s *t*-test). No significant differences were detected between T24M shscramble and T24M untransduced cells either at the EIF3D RNA or at the protein level (*p >* 0.05, Student’s *t*-test).

### EIF3D knockdown attenuates the tumorigenic properties of T24M cells

**Figure 5:**
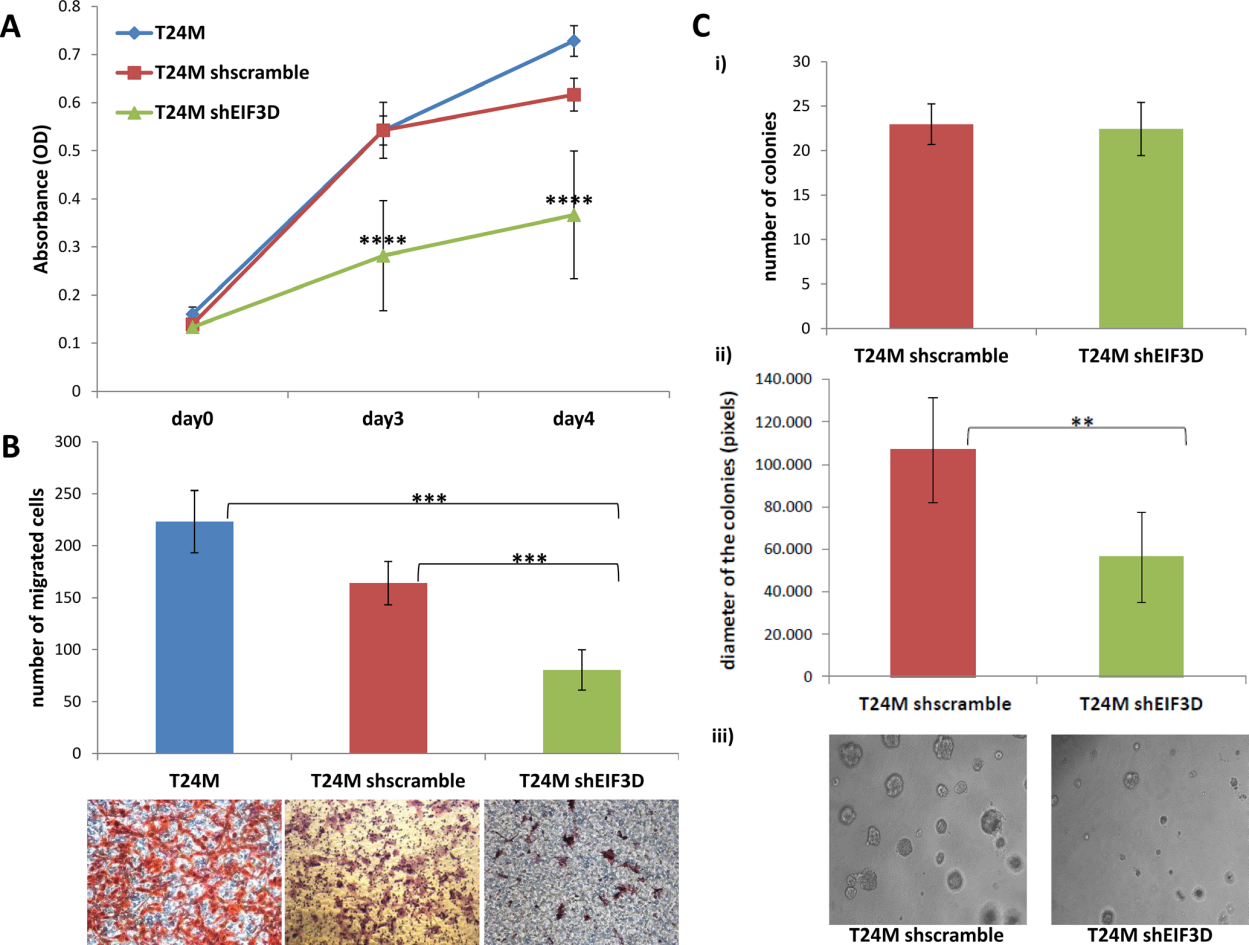
Impact of EIF3D downregulation on cell proliferation, migration and colony forming ability of T24M cells (**A**) The knockdown of EIF3D significantly reduced the proliferation rate of T24M cells. The bar graph represents the proliferation rate of T24M, T24M shscramble and T24M shRHEB cells at three different time points (Day 0, Day 3, Day 4). The values represent the means ± SD from three independent experiments performed in five replicates (two-tailed Student’s *t*-test, ****p ≤ 0.0001). (**B**) A significant reduction was also observed in the migratory capacity of T24M cells following EIF3D knockdown. The graph illustrates the number of cells migrated towards conditioned media derived from T24M cells. The cells were allowed to migrate for 6h toward the CM. Representative images of the migrated cells from each condition are displayed below the graph. Magnification: 10×. The values represent the means ± SD from two independent experiments performed in duplicate (two-tailed Student’s *t*-test, ***p ≤ 0.001). (**C**) The colony forming ability of T24M cells was significantly reduced in the case of EIF3D knockdown after 10 days of growth on matrigel. i) The bar graph illustrates the mean number of colonies formed by T24M shscramble and T24M shEIF3D cells. ii) Bar graph presenting the average diameter of the colonies formed by T24M shscramble and T24M shEIF3D cells. Although no significant difference in the number of colonies was detected, a remarkable decrease in the diameter of the colonies was observed upon to EIF3D knockdown. Colony diameters were measured by using ImageJ software and their length was given in pixels. iii) Representative images of the colonies formed by T24M shscramble and T24M shEIF3D cells. Magnification: 10x. The values represent the means ± SD from two independent experiments performed in duplicate (two-tailed Student’s *t*-test, **p ≤ 0.01).

To elucidate the possible impact of EIF3D on BC, we further investigated the impact of its knockdown onto the malignant phenotype of T24M cells, including impact on cell proliferation, migration and colony forming ability *in vitro* and tumor growth in NOD/SCID T24M xenografts. As shown in Figure 5A, the stable knockdown of EIF3D in T24M cells resulted in a 63% decreased proliferation rate compared to T24M shscramble cells after culture for 3 days (0.282 ± 0.115 au versus 0.543 ± 0.058 au; *p ≤* 0.0001, Student’s *t*-test) and in a 51% decrease after 4 days (0.367 ± 0.133 au versus 0.617 ± 0.034 au; *p ≤* 0.0001, Student’s *t*-test), respectively. The data from both time points were normalized to the measurements from day 0 (0.139 ± 0.010 au for T24M shscramble and 0.133 ± 0.007 au for T24M shEIF3D). No significant difference was observed between T24M shscramble and T24M untransduced cells. A significant reduction was also observed in the migratory capacity of T24M cells towards their conditioned media (CM) following EIF3D knockdown compared to T24M shscramble cells (80 ± 19 cells versus 164 ± 21 cells; *p ≤* 0.001, Student’s *t*-test), (Figure 5B), whereas, no significant difference in the migration of T24M shscramble and T24M untransduced cells was detected. With respect to T24M colony forming ability (Figure 5C), no significant difference in the number of colonies formed by T24M shEIF3D and T24M shscramble cells (23 ± 2 versus 22 ± 3; *p* = 0.86, Student’s *t*-test) was observed. Nevertheless, the diameter of the colonies was remarkably decreased in the former (56.459 ± 10.965 pixels versus 100.362 ± 17.516 pixels; *p ≤* 0.01, Student’s *t*-test), indicating impaired colony growth. These findings clearly indicate that EIF3D has a significant influence on the *in vitro* properties of T24M cells such as proliferation, migration and colony forming ability.

**Figure 6:**
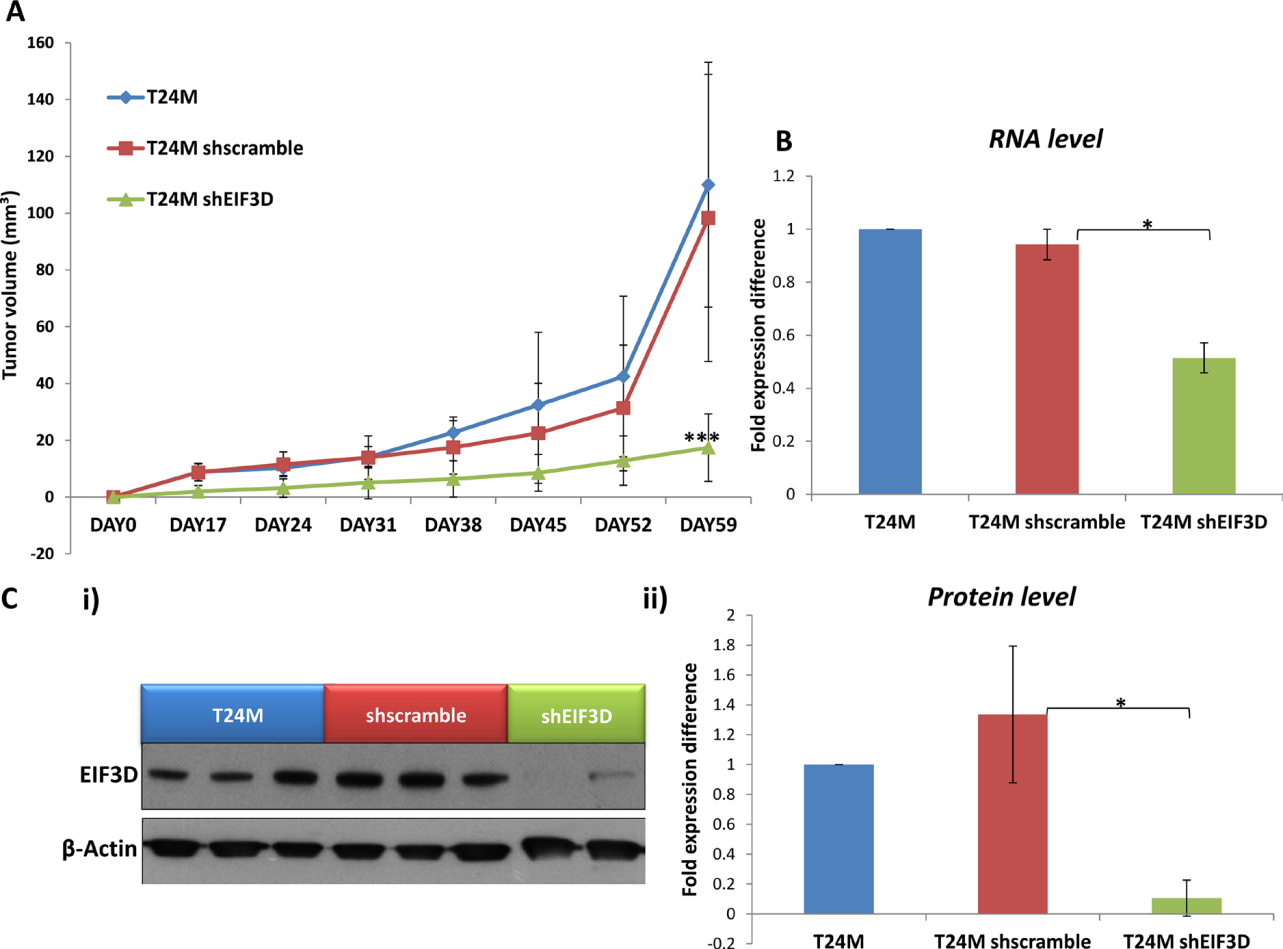
The knockdown of EIF3D impairs tumor growth *in vivo* (**A**) Tumor growth in T24M, T24M shscramble and T24M shEIF3D tumor bearing NOD/SCID mice. Tumor volume was significantly smaller (***p < 0.01, Student’s *t*-test) in T24MshEIF3D compared with T24M or T24M shscramble tumor bearing animals 59 days after the injections. (**B**) The expression of EIF3D was estimated in excised tumors from all groups of mice, 60 days after the injections, at the RNA level. Bar graph representing the downregulation of EIF3D in T24M shEIF3D tumors in comparison to T24M shscramble and T24M tumors analyzed with real-time PCR (*p < 0.05, Student’s *t*-test). (**C**) The knockdown of EIF3D in the tumors was also confirmed 60 days after the injections at the protein level. i) Western blot analysis for EIF3D in T24M, T24M shscramble and T24M shEIF3D tumors. ii) Bar chart showing the decreased levels of EIF3D in T24M shEIF3D tumors compared to T24M and T24M shscramble tumors (*p < 0.05, Student’s *t*-test).

Based on these *in vitro* results, the impact of EIF3D knockdown was further investigated *in vivo*. T24M (*n =* 12), T24M shscramble (*n =* 12) and T24M shEIF3D (*n =* 14) tumor bearing mice were generated and examined for tumor growth over a period of at least 60 days. A significantly reduced tumor volume (17.41 ± 11.88 mm³) was observed in the T24M shEIF3D mice compared to the T24M (110.03 ± 43.13 mm³; *p ≤* 0.0001, Student’s *t*-test) or the T24M shscramble (98.32 ± 50.59 mm³; *p ≤* 0.001, Student’s *t*-test) tumor bearing mice (Figure 6A). The expression levels of EIF3D were further analysed in the excised tumors from all groups, 60 days after the injections, at the RNA level by real-time PCR and at the protein level by Western blot analyses. As shown in Figure 6B, EIF3D was significantly reduced at the mRNA level by 45% ± 2.6% in T24M shEIF3D compared to T24M shscramble tumors (*p ≤* 0.05, Student’s *t*-test) whereas no significant difference was observed between the T24M and the T24M shscramble tumors (*p* = 0.39, Student’s *t*-test). Additionally, in another set of tumors the expression of EIF3D was examined at the protein level and, as illustrated in Figure 6C, it was found reduced by 92.78% ± 6.74% in T24M shEIF3D tumors compared to T24M shscramble (*p ≤* 0.01, Student’s *t*-test). No significant difference was detected between the T24M and the T24M shscramble tumors (*p* = 0.33, Student’s *t*-test).

The specificity of the EIF3D findings was further supported by testing the impact of knock-down of an additional potential target selected from the pathway analysis: GTP-binding protein Rheb (RHEB), not previously associated with BC, laying upstream of EIF3D and considered one of the key molecules activating the mTOR complex ([51–54]; Supplementary Figure 1). In agreement with our predictions, RHEB was overexpressed in invasive versus non-invasive BC by Western blot analysis of a small set of tissue specimens (Supplementary Figure 3). However, while the lentivirus-mediated RNAi knockdown of RHEB (Supplementary Figure 4) decreased cell proliferation, migration and colony forming ability *in vitro* (Supplementary Figure 5), no impact on tumor growth *in vivo* was observable (Supplementary Figure 6).

## DISCUSSION

The tissue proteome is a rich source of information about disease, as tissue is the site of disease initiation and progression (reviewed in [55]). In this study, we performed LC-MS/MS analysis of tissue specimens from NMIBC and MIBC in order to identify proteins significantly altered in MIBC and predict additional molecules and pathways altered during BC progression.

Considering the clinical relevance (limited treatment options for MIBC and associated significant decrease in the survival rate for patients that progress to MIBC), we focused our investigation on the comparative analysis between muscle-invasive (case group) and non-muscle invasive BC (control group). Inclusion of the non-malignant tissue specimens in the proteomic analysis was not possible due to restricted sample availability, nevertheless this was performed during the more targeted IHC studies (Figure 2). By performing a comprehensive proteomic analysis, 144 proteins were found to be significantly differentially expressed between MIBC and NMIBC samples. Many of these proteins including annexin A10 (ANXA10) [40], HPGD [36], actinin alpha 4 (ACTN4) [42, 43], CTSE [47] and CDH13 [46] and a total of 21 others [including transforming growth factor-beta-induced protein ig-h3 (TGFBI), palladin (PALLD), adipogenesis regulatory factor (ADIRF), anterior gradient protein 2 homolog (AGR2), keratin type I cytoskeletal 19 (KRT19)] have already been associated with BC, when compared with a database on MIBC [56], serving as “positive controls” for the applied approach. Our results also revealed numerous proteins that have not been associated with or reported in BC tissue, including PGRMC1, FUCA1, BROX, coatomer protein complex subunit alpha (COPA), or threonyl-TRNA synthetase (TARS), with the differential expression of FUCA1, BROX, PGRMC1, and PSMD12 having been further confirmed in a small set of additional tumors by IHC. For the latter (PGRMC1), similar expression trends with a decrease in MIBC in comparison to NMIBC have also been observed in urine [44, 57]. FUCA1 is a lysosomal enzyme responsible for removal of terminal L-fucose residues from oligosaccharide chains of glycosylated proteins [58]. It has been reported that treatment of highly invasive breast cancer cells (MDA MB 231) with alpha-L-fucosidase significantly decreases their invasive potential [59]. Additionally, adhesion of breast cancer cells to ECM components was reduced upon treatment with FUCA1 [60], supporting its inhibitory potential in cancer invasion (reviewed in [61]). In contrast to FUCA1, the biological function of BROX has not been elucidated yet [62–64]. The majority of proteins predicted to be a part of BROX interactome (based on STRING analysis) are components of the endosomal-sorting complex required for transport (ESCTR) system, which may suggest an involvement of this factor in sorting and recycling of growth factors. Finally, PSMD12 (also known as Rpn5), recently predicted to promote proliferation and metastasis based on network analysis [65], is a component of the multi-protein complex - 26 proteasome, involved in the ATP-dependent degradation of ubiquitinated proteins. Specifically, PSMD12 is one of the particles that builds the “lid” of the proteasome cap, with the latter being involved in the de-ubiquitination of targeted proteins (reviewed in [66]). Although, numerous inhibitors targeting the proteolytic activity of 26S proteasome complex have been investigated as potential anti-tumor agents (reviewed in [67, 68]), the relevance of PSMD12 in cancer has not been investigated yet. It appears that further investigation of the biological relevance of these proteins in BC would be of interest.

An in-depth analysis of the proteomics findings revealed that a wide range of the aberrantly expressed proteins are involved in cancer hallmark processes such as cell cycle, proliferation, cell metabolism, apoptosis and furthermore in processes that are associated with cancer invasion, such as cytoskeleton remodeling, cell adhesion and migration. *In silico* analysis of the proteomics findings at pathway and interactome levels revealed an activation of protein synthesis pathways (eIF2 signaling, tRNA charging, regulation of eIF4 and p70S6K signaling and mTOR signaling), which have been previously reported to be deranged in the context of BC [69–79]. Collectively, these data enhance the validity of the proteomic analysis, and increase the credibility of the novel findings such as the observed decrease of FUCA1, BROX, PGRMC1 and increase of PSMD12 (also supported by the IHC analysis, Figure 2) in MIBC.

Given that protein synthesis pathways were clearly predicted to be activated in tumor growth and progression [80], they were more thoroughly investigated in order to better understand the contribution of individual proteins. Cancer cells are characterized by translational alterations that increase the rate of protein synthesis, sustaining in this way, cancer progression and survival [81–83]. One of the most complex and rate-limiting steps in this process is translation initiation, in which a great number of eukaryotic initiation factors are involved [84, 85]. Aberrant expression, mutations and post-translational modifications of many translation initiation factors have been previously described in different cancer types [50, 80]. Among the translation initiation factors, eukaryotic initiation factor 3 (eIF3) is the largest and most complex component. The human eIF3 translation initiation factor is an 800 kDa complex that consists of 13 subunits and acts as a scaffold for the assembly of the initiation complex [86, 87]. The aberrant expression of eIF3 subunits in different cancer types has been previously described in numerous studies [88]. In our study, EIF3D subunit was found to be up-regulated in MIBC compared to NMIBC and the knockdown of this factor resulted in the reduction of T24M cell proliferation, migration and colony forming ability *in vitro*, and decreased tumor growth in a xenograft mouse model *in vivo*. These findings are novel in the field of BC and demonstrate for the first time that EIF3D may promote the progression of this malignancy. Similarly with our findings, EIF3D was found to be up-regulated in recent studies in other cancer types including prostate cancer [89], non-small cell lung cancer [90], colon cancer [91], breast cancer [92], renal cell carcinomas [93], melanomas [94] and gliomas [95]. In the aforementioned studies, it was reported that the depletion of EIF3D resulted in cell cycle arrest and reduced the proliferation rate and colony forming ability of the cancer cells. Furthermore, a decreased migratory capacity of the cancer cells upon suppression of EIF3D was also described [89, 92]. All the previously reported data concerning the EIF3D in other cancer types are consistent with our findings. The increasing number of studies indicates that increased level of EIF3D in cancer may be a common feature in several malignancies. In BC, other eIF3 subunits have been investigated and the results are in accordance with our data [96, 97]. Theodorescu’s group identified eukaryotic initiation factor 3 subunit B (EIF3B) expression elevated in human bladder and prostate cancers [97]. The depletion of this factor *in vitro* resulted in decreased proliferation rate due to cell cycle arrest at the G1/S transition, inhibited cell migration, and *in vivo* delayed tumor growth [97]. In another study, Spilka et al. observed that the upregulation of eukaryotic initiation factor 3 subunit A (EIF3A) in BC was correlated with tumor grade, prompting the authors to suggest that EIF3A could serve as a prognostic biomarker in low grade tumors [96]. Knocking down EIF3A decreased proliferation rate, and reduced invasion and tumor formation in mice [96]. The above data and our findings suggest that BC progression may be supported by increased expression and activity of the eIF3 complex. Thus, EIF3D could be potentially amenable to pharmacological targeting.

So far, therapeutic strategies targeting the translational machinery components in cancer focused on the eukaryotic translation initiation factor 4F complex and specifically the EIF4E subunit (reviewed in [50]). Recent evidence, however, supports the existence of an alternative cap-dependent translation mechanism, independent from EIF4E [98]. This recently described model, suggests that translation is driven by EIF3D, through recognition of the 5′ mRNA cap in a subset of mRNAs (such as the cell proliferation regulator c-Jun) that are eIF3-specialized and where EIF4E recruitment is blocked [98]. This gives rise to another layer of cap-dependent translation. Collectively, our results suggest that the development of inhibitors for the subunits of the eIF3 complex (EIF3B, EIF3D) may be a promising research avenue toward pharmaceutical intervention in BC especially in cases where EIF4E is apparently missing.

The predicted activation of the translational machinery based on the observed proteomics changes in MIBC also included a predicted activation of the mTOR pathway. As illustrated in Supplementary Figure 1, the *in silico* analysis of the proteomics findings, predicted a hyperactivation state for both the mammalian target of rapamycin complex 1 (mTORC1) and mammalian target of rapamycin complex 2 (mTORC2) with their negative regulator 5′ AMP-activated protein kinase (AMPK) detected from the proteomic analysis being down-regulated. RHEB is a member of the small Ras GTPase family [99] that serves as an upstream activator of mTORC1 [100]. The predicted overexpression of RHEB in MIBC compared to NMIBC based on the *in silico* analysis was confirmed by Western blot. However, interestingly, inactivation of RHEB failed to impact tumor growth *in vivo*. Existence of mechanisms bypassing mTOR inhibition have been suggested to explain negative results from respective clinical trials (reviewed in [101]). Based on our results, the overexpression of downstream proteins in protein synthesis-related pathways, such as EIF3D, may be contributing to the acquired resistance of cancer cells to mTOR inhibition, a hypothesis meriting further investigation.

Collectively, our study represents a comprehensive analysis of BC proteome from patients with muscle invasive and non- muscle invasive disease providing a proteomic component to support molecular integrative studies in the future. Functional analysis suggested a potential therapeutic effect for EIF3D. This finding requires further investigation in animal models, as well as in tumor specimens in relation to muscle invasive molecular subtypes. Determining EIF3D therapeutic potential, especially in tumors where eIF4 inhibitors apparently fail, may open up exciting avenues for further research toward personalized treatment.

## MATERIALS AND METHODS

### Patients and tumor characteristics

BC tissue specimens were collected from patients undergoing cystectomy or transurethral resection of BC in medical centers in Greece (Laikon Hospital, Athens) and Germany (Department of Urology and Urological Oncology, Hannover Medical School). Sample collection was approved by the respective local ethics committees (for Athens Ε.S 618–2012 and for Hannover 614–2009), and all individuals gave written informed consent. Samples from tumor tissue from 11 patients were employed for the proteomic analysis including non-muscle invasive (stage pTa, *n =* 5) and muscle invasive bladder cancer cases (stage pT2+, *n =* 6); whereas for the IHC, from a total of 8 patients with pTa, 8 with pT1 and 8 patients with pT2+. Normal controls (*n =* 6); corresponded to normal adjacent epithelium from patients that underwent cystectomy. Tumor stage was determined according to TNM classification of malignant tumors [102], whereas grading was performed in accordance to World Health Organization (WHO) Grading System 2004 [103].

### Sample preparation

Approximately 20 mg of BC tissue was homogenized in 150 μl of lysis buffer (4% SDS, 0.1M DTE, 0.1M Tris-HCl pH 7.6) using a blade homogenizer (three cycles of 30–40 s) followed by sonication (15 s per sample). Undissolved materials were removed by centrifugation at 16,000 rcf for 10 min. Protein concentration was determined by the Bradford assay (BioRad, California, USA). Protein extracts (200 µg) were processed using filter aided sample preparation (FASP) as described previously [104], with some minor modifications [105]. Briefly, buffer exchange was performed in Amicon Ultra Centrifugal filter devices (0.5 ml, 30 kDa MWCO; Merck, NJ, USA) at 16,000 rcf for 15 min at room temperature. The protein extract was mixed with urea buffer (8M urea in 0.1M Tris-HCl pH 8.5) and centrifuged. The concentrate was diluted with urea buffer and centrifugation was repeated. Alkylation of proteins was performed by adding 0.05M iodoacetamide in urea buffer followed by 20 min incubation in the dark and centrifugation at 16,000 rcf for 10 min. Additional series of washes were conducted with urea buffer (2 times) and ammonium bicarbonate buffer (50 mM NH_4_HCO_3_ pH 8, 2 times). Tryptic digestion was performed overnight using trypsin to protein ratio 1:100. Peptides were eluted by centrifugation at 16000 rcf for 10 min, lyophilized and stored at –80°C until further use.

### LC-MS/MS analysis

Tryptic digests were loaded onto a Dionex Ultimate 3000 RSLS nano flow system (Dionex, Camberly, UK). After loading onto a Dionex 0.1 × 20 mm 5 μm C18 nano trap column at a flow rate of 5 μl/min in 0.1% formic acid and 2% acetonitrile, samples were applied onto an Acclaim PepMap C18 nano column 75 μm × 50 cm, 2 μm 100 Å at a flow rate of 0.3 μl/min. The trap and nano flow column were maintained at 35°C. The samples were eluted with a gradient of solvent A: 0.1% formic acid versus solvent B: 80% acetonitrile starting at 1% B for 5 min rising to 5% B at 10 min then to 25% B at 360 min and 65% B at 480 min.

The eluent was ionized using a Proxeon nano spray ESI source operating in positive ion mode into an Orbitrap Velos FTMS (Thermo Finnigan, Bremen, Germany). Ionization voltage was 2.6 kV and the capillary temperature was 200°C. The mass spectrometer was operated in MS/MS mode scanning from 380 to 2,000 m/z. The top 20 multiply charged ions were selected from each scan for MS/MS analysis using CID at 40% collision energy. The resolution in MS1 was 60,000 and 7,500 at m/z 400 for CID in MS2.

### Data processing and quantification

The raw data from LC-MS/MS analysis were evaluated using commercially available Proteome Discoverer (PD) v. 1.2 (Thermo Scientific) and open source Trans-Proteomic Pipeline (TPP) v. 4.6.3 (Institute for Systems Biology, Seattle Proteomic Center; http://tools.proteomecenter.org/wiki/index.php?title=Software:TPP). Database search was carried out against Human Swiss-Prot Database [106, 107] (30/10/2013) containing only the canonical sequences with 20,277 reviewed entries using Sequest [108] and X!Tandem [109] search engines in PD and TPP [110, 111], respectively. Both analyses, using PD and TPP, were performed using comparable parameters including a) precursor mass tolerance 10 ppm, b) fragment mass tolerance: 0.8 Da, c) fixed modification: carbamidomethylation of cysteine, d) variable modifications: oxidation of methionine and proline, e) not allowing for semitryptic peptides, and f) allowing one missed cleavage. Moreover, using TPP, another database search was also performed against a concatenated database with a shuffled version of decoy database as created using COMPASS [112]. Additionally, to generate appropriate input files, raw data were converted to mzML format using Msconvert (ProteoWizard). Results from TPP were further validated using PeptideProphet [113] and ProteinProphet [114] which are incorporated in the TPP software. Proteins with false positive rate less than 5% were included for subsequent quantification. For the analysis in PD, dataset was filtered requiring mass deviation below 5 ppm between experimental and theoretical mass and only peptides characterized by high confidence were included. Peptide confidence was assessed based on Xcorr score and charge. Peptides having the charge of 1, 2, 3, >4 and the respective XCorr value higher than 1.5, 2, 2.5, 3 were defined as high confidence. In both cases, protein grouping was disabled and proteins identified by a single peptide (unique or shared with other proteins) were also considered.

Quantification analysis was performed using peak area-based quantification (for PD) and spectral counting (for TPP). Given these stringent conditions of data analysis, employing two independent approaches for quantification, all peptides (unique or shared with other proteins) assigned to a protein were taken into consideration in order to maximize proteome coverage. Of note, the inclusion of non-unique peptides seems to not have a significant impact on quantification results (as revealed by correlation analysis performed upon inclusion/exclusion of shared peptides, *data not shown*). The peak area-based quantification uses precursor ions to assess the relative abundance of identified proteins in the label-free data. For each precursor ion, peak area (i.e. area under the curve) is calculated from the extracted ion chromatogram during data processing in PD by using the Precursor Ions Area Detector node. Protein quantification was based on the protein area values calculated as an average of the area for three most abundant peptides. When the peptide was not identified in the particular sample, the missing values were replaced with zero. Part per million (ppm)-normalization was conducted for the proteins identified in individual samples according to the following formula: Normalized peak area = (Peptide peak area/Total peak area) × 10^6^. The spectral count-based quantification was performed using the Absolute Protein Expression Quantification algorithm (APEX), as described previously [115]. The normalized APEX score was obtained by dividing individual protein values by the total APEX score for each sample. The mean protein abundance per groups (pTa or pT2+) and SD were then calculated. The changes in the relative abundance were represented by the fold change, calculated as a ratio for muscle invasive (pT2+) to non-muscle invasive cases (pTa). In both approaches, only proteins consistently reported in ≥ 60% of the samples (at least in one group: pTa and/or pT2+) were considered as credible and included for further statistical assessment.

### Statistical analysis

Statistical analysis was performed using the SPSS Statistical Software (SPSS 17.0, IBM). The distribution of the data was evaluated using the Kolmogorov-Smirnov test. Since a normal distribution was observed, independent sample *t*-test was applied. Proteins with a *p*-value below 0.05 were considered as statistically significant.

### Bioinformatics analysis

The impact of the differentially expressed proteins was further evaluated in the context of pathway as well as interactome network analyses. The pathways were generated using QIAGEN’s Ingenuity Pathway Analysis (IPA®, QIAGEN Redwood City, www.qiagen.com/ingenuity). Statistical analysis was conducted by using right-tailed Fisher’s exact test and pathways with a *p*-value below 0.05 were considered as significant. The protein interaction network was created using STRING v. 9.1 (Search Tool for the Retrieval of Interacting Genes/Proteins, http://string-db.org/) using default settings [116].

### Immunohistochemistry on tissue microarrays

Verification of proteomics findings was performed using immunohistochemistry (IHC) staining on tissue microarrays, as described previously [105]. Briefly, immunochistochemical analysis was performed for (i) BRO1 domain-containing protein BROX (BROX; rabbit polyclonal anti-BROX, Novus Biologicals, dilution 1:300), (ii) Membrane-associated progesterone receptor component 1 (PGRMC1; rabbit polyclonal anti-PGRMC1, Proteintech, dilution 1:50), (iii) Tissue alpha-L-fucosidase (FUCA1; rabbit polyclonal anti-FUCA1, Proteintech, dilution 1:100) and (iv) 26S proteasome non-ATPase regulatory subunit 12 (PSMD12; rabbit polyclonal anti-PSMD12, Novus Biologicals, dilution 1:400). Staining intensity was quantified using ImageJ software as described [105].

### Cell lines and conditioned media

The human embryonic kidney cell line 293T and the human fibrosarcoma cell line HT1080 were obtained from American Type Culture Collection (ATCC). The human bladder cancer cell line T24M, represents the metastatic variant of T24 cells and was developed by our group after following cycles of subcutaneous injections of T24 cells in mice and re-isolation and culture of metastatic cells *in vitro* as previously described [117]. All the cell lines were cultured in DMEM media (Gibco-BRL, Paisley, Scotland UK) supplemented with 10% fetal bovine serum (FBS; Gibco-BRL, Paisley, Scotland UK) and 1% Penicillin-Streptomycin (Pen-Strep; Gibco-Invitrogen) at 37°C in 5% CO_2_ under sterile conditions. For the preparation of the conditioned media (CM), T24M cells were incubated in DMEM supplemented with 2% FBS, 1% Pen-strep for 48h at 37°C in 5% CO_2_. Subsequently, the CM was collected after centrifugation at 2000 rcf for 10 min in order to remove dead cells and debris, as described before [118].

### Production of shRNA lentiviruses and transduction of the target cell lines

The knockdown study was performed using lentiviral-mediated RNA interference. The lentiviral vectors with the sequences for the shRNAs were purchased from the Erasmus Center for Biomics. The shRNA sequences targeting EIF3D and RHEB were 5′-CCGGCCTCAGACATACTCCATAGATCTCGAGATCTATGGAGTATGTCTGAGGTTTTTG-3′, respectively. A scrambled shRNA (shscramble) was used as control, as previously described [119]. For the lentivirus production, a four plasmid system was used for the transient transfection of 293T cells as previously described [120, 121], followed by concentration with Amicon Ultra Centrifugal Filters-100K Units (Merck Millipore). The titers of the concentrated lentiviruses were determined after infection of HT1080 cells with serial dilutions of the viral stock. The titers were estimated at 5×10^8^ – 10^9^ infection units (IU)/ml. The lentiviruses were then used for the transduction of the target cell line T24M. As control T24M cells transduced with a lentivirus for shscramble was used. The knockdown effect was evaluated at the RNA level by real-time PCR and at the protein level by Western blot analyses as described in the following sections.

### Real-time PCR

Total RNA was isolated from T24Mshscramble, T24MshEIF3D and T24MshRHEB cells 4 days after the transduction and from untransduced T24M cells using the TRI Reagent (Sigma-Aldrich) according to the manufacturer’s instructions. Total RNA was also isolated from T24M, T24Mshscramble, T24MshEIF3D and T24MshRHEB tumors from the respective groups of mice by using the same protocol. For the cDNA synthesis 1μg of total RNA was used as starting material for SuperScript II reverse transcriptase (Invitrogen). Real-time PCR was performed on a SaCycler-96 (Sacace Biotechnologies) using the SYBR Green master mix (Kapa Biosystems) and the following primers: EIF3D (forward): 5′-CAGCGGAATCGAATGAGATTTGC-3′ EIF3D (reverse): 5′-GTTTGGCACTCTTAGGCAGGA-3′, RHEB (forward): 5′-TTGTGGACTCCTACGATCCAA-3′, RHEB (reverse): 5′-GGCTGTGTCTACAAGTTGAAGAT-3′ according to the manufacturer’s protocol. GAPDH was used as an internal control. The relative gene expression analysis was performed by implementing the 2^–ΔΔCt^ method.

### Western blot analysis

Total cell extracts were obtained from T24Mshscramble, T24MshEIF3D and T24MshRHEB cells 4 days after the lentiviral transduction and from untransduced T24M cells. Total cell extracts were also obtained from T24M, T24M shscramble, T24M shEIF3D and T24M shRHEB tumors from the respective groups of mice. Protein extracts were separated by 10% SDS-PAGE under reducing conditions and transferred to Hybond-ECL nitrocellulose membrane (Amersham Biosciences). The membranes were blocked in TBS-0.1% Tween 5% milk for 2 hours at room temperature, then they were washed with TBS-0.1% Tween and incubated overnight at 4°C with the following primary antibodies: rabbit anti-human EIF3D (Proteintech, dilution 1:400), mouse anti-human RHEB (Santa Cruz, dilution 1:500). Membranes were washed again as described above and then incubated for 2 h at room temperature with secondary antibodies conjugated with HRP (anti-rabbit, Amersham, dilution 1:10,000; anti-mouse, Santa Cruz, dilution 1:2,000). After washing membranes with TBS-0.1% Tween, the target proteins were detected with an ECL detection kit (Thermo Scientific). B-actin was used as a loading control. The films were scanned at a GS-800 imaging densitometer (BioRad) in transmission mode and densitometry analysis of the results was performed using the Quantity One software (BioRad).

### MTS cell proliferation assay

T24M, T24Mshscramble, T24MshEIF3D and T24MshRHEB cells were seeded in 96-well plates at a density of 1,000 cells per well and cultured for 3 and 4 days, respectively. At the indicated time points the recommended amount of MTS reagent (Promega, Madison, USA) was added into each well of the 96-well plate and the cells were then incubated for additional 3 hours at 37°C in a humidified, 5% CO_2_ atmosphere. The absorbance was then recorded at 490 nm with a 96-well plate reader (SPECTROstar Nano, BMG LABTECH). For the calculation of the percentage difference in the proliferation rate the following formula was used: [(OD_day__×_ – OD_day__0_)/(ODcontrol_day__×_ - ODcontrol_day__0_)] ×100. Each experiment was performed in five replicates and repeated two times.

### Colony formation assay

T24M, T24Mshscramble, T24MshEIF3D and T24MshRHEB cells were plated at a density of 1000 cells per well in 96-well plates pre-coated with matrigel (BD Biosciences, New Jersey, USA) according to the manufacturer’s instructions. The cells were incubated for 10 days at 37°C in a humidified, 5% CO_2_ atmosphere. Photographs were taken from 5 fields of view (×10 or ×5) for each well, using a Leica CTR MIC microscope. The number and the size of the colonies were counted by using the Image J 1.49v software. Two independent experiments were performed, each including three replicates.

### Transwell migration assay

Transwell migration assays were performed as previously described [118, 120, 122]. In brief, 5 × 10^4^ cells T24M, T24Mshscramble, T24MshEIF3D and T24MshRHEB were added to the inside compartment of transwell inserts with 5μm pores (Corning-Costar, Cambridge, MA). As stimulus, CM derived from T24M cells was added at the bottom chamber of the transwell plate. Cells were allowed to migrate for 6 hours. Subsequently, the top of each insert was washed with PBS and the cells that had not migrated through the membrane were removed with a wet cotton swab. The migrated cells at the bottom of the insert were fixed with 4% paraformaldehyde (Sigma Aldrich) and stained with eosin-haematoxylin. The migratory capacity of the cells was assessed by counting the number of the cells that passed through the pore membrane. Photographs from 5 different fields (10x) of each insert were taken using a Leica CTR MIC microscope. Stained cells were counted using the Image J 1.49v software. Two independent experiments were performed, each including two replicates. Statistical analysis was performed using Student's *t*-test.

### Animal experiments

The NOD-SCID mice used for the *in vivo* experiments were housed and maintained at the Animal Facility of the Biomedical Research Foundation of the Academy of Athens. The procedures applied for the animal care and treatment were according to the recommendations of the Federation of European Laboratory Animal Science Associations (FELASA) and also approved by the Institutional Animal Care and Use Committee. For the development of the tumors, 3.3 × 10^6^ T24M shEIF3D (*n =* 14), T24M shRHEB (*n =* 13), T24M shscramble (*n =* 12) and T24M (*n =* 12) cells were administered subcutaneously into the tail base of 8–10 weeks old male NOD/SCID mice, as described previously [119, 120]. The tumor size was measured on a weekly basis by using a caliper until the tumors reached an average diameter of 12–15mm. At that time point the animals were sacrificed and tumors, lungs, livers, spleens and kidneys were collected. In the presence of tumor ulceration or bleeding the mice were sacrificed earlier.

## SUPPLEMENTARY MATERIALS FIGURES AND TABLES







## Abbreviations

ACTN: alpha actinins
ACTN4: actinin alpha 4
ADIRF: adipogenesis regulatory factor
AGR2: anterior gradient protein 2 homolog
AMPK: 5′ AMP-activated protein kinase
ANXA: annexins
ANXA10: annexin A10
APEX: Absolute Protein Expression Quantification algorithm
ATCC: American Type Culture Collection
BC: bladder cancer
BPGM: bisphosphoglycerate mutase
BROX: BRO1 domain-containing protein BROX
CCND1: cyclin D1
CDH13: cadherin 13
CDKN1B: cyclin dependent kinase inhibitor 1B
CDKN2A: cyclin dependent kinase inhibitor 2A
CIS: carcinoma *in situ*
CM: conditioned media
COPA: coatomer protein complex subunit alpha
CTLA-4: cytotoxic T-lymphocyte associated protein 4
CTSE: cathepsin E
ECM: extracellular matrix
eIF2: eukaryotic initiation factor 2
eIF3: eukaryotic initiation factor 3
EIF3A: eukaryotic translation initiation factor 3 subunit A
EIF3B: eukaryotic translation initiation factor 3 subunit B
EIF3D: eukaryotic translation initiation factor 3 subunit D
eIF4: eukaryotic initiation factor 4
ESCTR: endosomal sorting complex required for transport
FASP: filter aided sample preparation
FBLN2: fibulin 2
FBS: fetal bovine serum
FELASA: Federation of European Laboratory Animal Science Associations
FGFR: fibroblast growth factor receptors
FUCA1: tissue alpha-L-fucosidase
G6PD: glucose-6-phosphate 1-dehydrogenase
GPNMB: transmembrane glycoprotein NMB
HPGD: hydroxyprostaglandin dehydrogenase 15-(NAD)
HRAS: HRas proto-oncogene, GTPase
IHC: immunohistochemistry
IU: infection units
KRT19: keratin type I cytoskeletal 19
LC-MS/MS: liquid chromatography-tandem mass spectrometry
MIBC: muscle invasive bladder cancers
mTOR: mammalian target of rapamycin;
mTORC1: mammalian target of rapamycin complex 1
mTORC2: mammalian target of rapamycin complex 2
NMIBC: non-muscle invasive bladder cancers
NRF2: nuclear factor, erythroid 2 like 2
p70S6K: ribosomal protein S6 kinase beta-1
PALLD: palladin
PD: Proteome Discoverer
PD-1: programmed cell death protein 1
Pen-Strep: Penicillin-Streptomycin
PGAM1: phosphoglycerate mutase 1
PGRMC1: membrane-associated progesterone receptor component 1
PI3K-AKT-mTOR: phosphatidylinositol 3-kinase (PI3K)- protein kinase B (Akt)-mTOR
PRKAG2: 5′-AMP-activated protein kinase subunit gamma-2
PSMD12: 26S proteasome non-ATPase regulatory subunit 12
RB1: RB transcriptional corepressor 1
RHEB: Ras homolog enriched in brain
RTCA: RNA 3′-terminal phosphate cyclase
SEC22B: vesicle-trafficking protein SEC22b
SND1: staphylococcal nuclease domain-containing protein 1
STMN1: stathmin 1
TAGLN2: transgelin 2
TARS: threonyl-TRNA synthetase
TGFBI: transforming growth factor-beta-induced protein ig-h3
TGOLN2: trans-golgi network integral membrane protein 2
TNM: TNM Classification of Malignant Tumours
TP53: tumor protein P53
TPP: Trans-Proteomic Pipeline
TYMP: thymidine phosphorylase
UBB: polyubiquitin-B
UBE2D3: ubiquitin-conjugating enzyme E2 D3
UCHL3: ubiquitin carboxyl-terminal hydrolase isozyme L3
UGP2: UTP-glucose-1-phosphate uridylyltransferase
VEGF: vascular endothelial growth factor
WHO: World Health Organization
