# Supplementary material for: Proteomics analysis of bladder cancer invasion: Targeting EIF3D for therapeutic intervention
Source: Oncotarget. 2017 Apr 20;8(41):69435–55. doi: 10.18632/oncotarget.17279 (PMC5642490; doi:10.18632/oncotarget.17279)
Supplement: Supplementary file 1 [file oncotarget-08-69435-s001.pdf]

Proteomics analysis of bladder cancer invasion: Targeting EIF3D for therapeutic intervention

Supplementary Materials

REFERENCES

1. Arima J, Imazono Y, Takebayashi Y, Nishiyama K, Shirahama T, Akiba S, Furukawa T, Akiyama S, Ohi Y. Expression of thymidine phosphorylase as an indicator of poor prognosis for patients with transitional cell carcinoma of the bladder. Cancer. 2000; 88:1131–1138.
2. Yoshii H, Ito K, Asano T, Horiguchi A, Hayakawa M, Asano T. Increased expression of alpha-actinin-4 is associated with unfavorable pathological features and invasiveness of bladder cancer. Oncol Rep. 2013; 30:1073–1080.
3. Koizumi T, Nakatsuji H, Fukawa T, Avirmed S, Fukumori T, Takahashi M, Kanayama H. The role of actinin-4 in bladder cancer invasion. Urology. 2010; 75:357–364.
4. Wang R, Morris DS, Tomlins SA, Lonigro RJ, Tsodikov A, Mehra R, Giordano TJ, Kunju LP, Lee CT, Weizer AZ, Chinnaiyan AM. Development of a multiplex quantitative PCR signature to predict progression in non-muscle-invasive bladder cancer. Cancer Res. 2009; 69:3810–3818.
5. Tseng-Rogenski S, Gee J, Ignatoski KW, Kunju LP, Bucheit A, Kintner HJ, Morris D, Tallman C, Evron J, Wood CG, Grossman HB, Lee CT, Liebert M. Loss of 15-hydroxyprostaglandin dehydrogenase expression contributes to bladder cancer progression. Am J Pathol. 2010; 176:1462–1468.
6. Li CF, Shen KH, Huang LC, Huang HY, Wang YH, Wu TF. Annexin-I overexpression is associated with tumour progression and independently predicts inferior disease-specific and metastasis-free survival in urinary bladder urothelial carcinoma. Pathology. 2010; 42:43–49.
7. Munksgaard PP, Mansilla F, Brems Eskildsen AS, Fristrup N, Birkenkamp-Demtroder K, Ulhoi BP, Borre M, Agerbaek M, Hermann GG, Orntoft TF, Dyrskjot L. Low ANXA10 expression is associated with disease aggressiveness in bladder cancer. Br J Cancer. 2011; 105:1379–1387.
8. Schiffer E, Vlahou A, Petrolekas A, Stravodimos K, Tauber R, Geschwend JE, Neuhaus J, Stolzenburg JU, Conaway MR, Mischak H, Theodorescu D. Prediction of muscle-invasive bladder cancer using urinary proteomics. Clin Cancer Res. 2009; 15:4935–4943.
9. Bhagirath D, Abrol N, Khan R, Sharma M, Seth A, Sharma A. Expression of CD147, BIGH3 and Stathmin and their potential role as diagnostic marker in patients with urothelial carcinoma of the bladder. Clin Chim Acta. 2012; 413:1641–1646.
10. Lin YL, Sun G, Liu XQ, Li WP, Ma JG. Clinical significance of CDH13 promoter methylation in serum samples from patients with bladder transitional cell carcinoma. J Int Med Res. 2011; 39:179–186.
11. Fristrup N, Ulhoi BP, Birkenkamp-Demtroder K, Mansilla F, Sanchez-Carbayo M, Segersten U, Malmstrom PU, Hartmann A, Palou J, Alvarez-Mugica M, Zieger K, Borre M, Orntoft TF, et al. Cathepsin E, maspin, Plk1, and survivin are promising prognostic protein markers for progression in non-muscle invasive bladder cancer. Am J Pathol. 2012; 180:1824–1834.
12. Sun Y, Cheng MK, Griffiths TR, Mellon JK, Kai B, Kriajevska M, Manson MM. Inhibition of STAT signalling in bladder cancer by diindolylmethane: relevance to cell adhesion, migration and proliferation. Curr Cancer Drug Targets. 2013; 13:57–68.
13. Jiang Z, Li C, Wang X. Glutathione S-transferase M1 polymorphism and bladder cancer risk: a meta-analysis involving 33 studies. Exp Biol Med (Maywood). 2011; 236:723–728.
14. Kang HW, Song PH, Ha YS, Kim WT, Kim YJ, Yun SJ, Lee SC, Choi YH, Moon SK, Kim WJ. Glutathione S-transferase M1 and T1 polymorphisms: susceptibility and outcomes in muscle invasive bladder cancer patients. Eur J Cancer. 2013; 49:3010–3019.
15. Iwaki H, Kageyama S, Isono T, Wakabayashi Y, Okada Y, Yoshimura K, Terai A, Arai Y, Iwamura H, Kawakita M, Yoshiki T. Diagnostic potential in bladder cancer of a panel of tumor markers (calreticulin, gamma -synuclein, and catechol-o-methyltransferase) identified by proteomic analysis. Cancer Sci. 2004; 95:955–961.
16. Dokun OY, Florl AR, Seifert HH, Wolff I, Schulz WA. Relationship of SNCG, S100A4, S100A9 and LCN2 gene expression and DNA methylation in bladder cancer. Int J Cancer. 2008; 123:2798–2807.
17. Kumar P, Nandi S, Tan TZ, Ler SG, Chia KS, Lim WY, Butow Z, Vordos D, De la Taille A, Al-Haddawi M, Raida M, Beyer B, Ricci E, et al. Highly sensitive and specific novel biomarkers for the diagnosis of transitional bladder carcinoma. Oncotarget. 2015; 6:13539–13549.
18. Chung H, Kim B, Jung SH, Won KJ, Jiang X, Lee CK, Lim SD, Yang SK, Song KH, Kim HS. Does phosphorylation of cofilin affect the progression of human bladder cancer? BMC Cancer. 2013; 13: 45.
19. Sheng KH, Yao YC, Chuang SS, Wu H, Wu TF. Search for the tumor-related proteins of transition cell carcinoma

in Taiwan by proteomic analysis. Proteomics. 2006; 6:1058–1065.

20. Marin-Aguilera M, Mengual L, Ribal MJ, Ars E, Rios J, Gazquez C, Villavicencio H, Alcaraz A. Utility of urothelial mRNA markers in blood for staging and monitoring bladder cancer. Urology. 2012; 79:240 e249–215.
21. Morsi MI, Youssef AI, Hassouna ME, El-Sedafi AS, Ghazal AA, Zaher ER. Telomerase activity, cytokeratin 20 and cytokeratin 19 in urine cells of bladder cancer patients. J Egypt Natl Canc Inst. 2006; 18:82–92.
22. Senga Y, Kimura G, Hattori T, Yoshida K. Clinical evaluation of soluble cytokeratin 19 fragments (CYFRA 21–1) in serum and urine of patients with bladder cancer. Urology. 1996; 48:703–710.
23. Aaboe M, Marcussen N, Jensen KM, Thykjaer T, Dyrskjot L, Orntoft TF. Gene expression profiling of noninvasive primary urothelial tumours using microarrays. Br J Cancer. 2005; 93:1182–1190.
24. Jiang J, Ulbright TM, Younger C, Sanchez K, Bostwick DG, Koch MO, Eble JN, Cheng L. Cytokeratin 7 and cytokeratin 20 in primary urinary bladder carcinoma and matched lymph node metastasis. Arch Pathol Lab Med. 2001; 125:921–923.
25. Schaafsma HE, Ramaekers FC, van Muijen GN, Robben H, Lane EB, Leigh IM, Ooms EC, Schalken JA, van Moorselaar RJ, Ruiter DJ. Cytokeratin expression patterns in metastatic transitional cell carcinoma of the urinary tract. An immunohistochemical study comparing local tumor and autologous metastases. Am J Pathol. 1991; 139:1389–1400.
26. Mitra AP, Pagliarulo V, Yang D, Waldman FM, Datar RH, Skinner DG, Groshen S, Cote RJ. Generation of a concise gene panel for outcome prediction in urinary bladder cancer. J Clin Oncol. 2009; 27:3929–3937.
27. Schnakenberg E, Breuer R, Werdin R, Dreikorn K, Schloot W. Susceptibility genes: GSTM1 and GSTM3 as genetic risk factors in bladder cancer. Cytogenet Cell Genet. 2000; 91:234–238.
28. Hu H, Zhao J, Zhang M. Expression of Annexin A2 and Its Correlation With Drug Resistance and Recurrence of Bladder Cancer. Technol Cancer Res Treat. 2016; 15: NP61-NP68.
29. Lu Y, Liu P, Wen W, Grubbs CJ, Townsend RR, Malone JP, Lubet RA, You M. Cross-species comparison of orthologous gene expression in human bladder cancer and carcinogen-induced rodent models. Am J Transl Res. 2010; 3:8–27.
30. Zhang Z, Yu XY, Zhang GJ, Guo KF, Kong CZ. Low microsomal epoxide hydrolase expression is associated with bladder carcinogenesis and recurrence. Asian Pac J Cancer Prev. 2012; 13:521–525.
31. Davies BR, O'Donnell M, Durkan GC, Rudland PS, Barraclough R, Neal DE, Mellon JK. Expression of S100A4 protein is associated with metastasis and reduced survival in human bladder cancer. J Pathol. 2002; 196:292–299.
32. Matsumoto K, Irie A, Satoh T, Ishii J, Iwabuchi K, Iwamura M, Egawa S, Baba S. Expression of S100A2 and S100A4 predicts for disease progression and patient survival in bladder cancer. Urology. 2007; 70:602–607.
33. Yao R, Davidson DD, Lopez-Beltran A, MacLennan GT, Montironi R, Cheng L. The S100 proteins for screening and prognostic grading of bladder cancer. Histol Histopathol. 2007; 22:1025–1032.
34. Oguztuzun S, Sezgin Y, Yazici S, Firat P, Ozhavzali M, Ozen H. Expression of glutathione-S-transferases isoenzymes and p53 in exfoliated human bladder cancer cells. Urol Oncol. 2011; 29:538–544.
35. Meyer-Siegler KL, Leifheit EC, Vera PL. Inhibition of macrophage migration inhibitory factor decreases proliferation and cytokine expression in bladder cancer cells. BMC Cancer. 2004; 4: 34.
36. Taylor JA, 3rd, Kuchel GA, Hegde P, Voznesensky OS, Claffey K, Tsimikas J, Leng L, Bucala R, Pilbeam C. Null mutation for macrophage migration inhibitory factor (MIF) is associated with less aggressive bladder cancer in mice. BMC Cancer. 2007; 7: 135.
37. Latosinska A, Makridakis M, Frantzi M, Borras DM, Janssen B, Mullen W, Zoidakis J, Merseburger AS, Jankowski V, Mischak H, Vlahou A. Integrative analysis of extracellular and intracellular bladder cancer cell line proteome with transcriptome: improving coverage and validity of -omics findings. Sci Rep. 2016; 6: 25619.
38. Burger M, Denzinger S, Hartmann A, Wieland WF, Stoehr R, Obermann EC. Mcm2 predicts recurrence hazard in stage Ta/T1 bladder cancer more accurately than CK20, Ki67 and histological grade. Br J Cancer. 2007; 96:1711–1715.
39. Kruger S, Thorns C, Stocker W, Muller-Kunert E, Bohle A, Feller AC. Prognostic value of MCM2 immunoreactivity in stage T1 transitional cell carcinoma of the bladder. Eur Urol. 2003; 43:138–145.
40. Latosinska A, Vougas K, Makridakis M, Klein J, Mullen W, Abbas M, Stravodimos K, Katafigiotis I, Merseburger AS, Zoidakis J, Mischak H, Vlahou A, Jankowski V. Comparative Analysis of Label-Free and 8-Plex iTRAQ Approach for Quantitative Tissue Proteomic Analysis. PLoS One. 2015; 10: e0137048.
41. Li CW, Chen BS. Network Biomarkers of Bladder Cancer Based on a Genome-Wide Genetic and Epigenetic Network Derived from Next-Generation Sequencing Data. Dis Markers. 2016; 2016: 4149608.
42. Yi CH, Smith DJ, West WW, Hollingsworth MA. Loss of fibulin-2 expression is associated with breast cancer progression. Am J Pathol. 2007; 170:1535–1545.
43. Baird BN, Schliekelman MJ, Ahn YH, Chen Y, Roybal JD, Gill BJ, Mishra DK, Erez B, O'Reilly M, Yang Y, Patel M, Liu X, Thilaganathan N, et al. Fibulin-2 is a driver of malignant progression in lung adenocarcinoma. PLoS One. 2013; 8: e67054.
44. Maric G, Rose AA, Annis MG, Siegel PM. Glycoprotein non-metastatic b (GPNMB): A metastatic mediator and emerging therapeutic target in cancer. Onco Targets Ther. 2013; 6:839–852.
45. Otero-Estevez O, Martinez-Fernandez M, Vazquez-Iglesias L, Paez de la Cadena M, Rodriguez-Berrocal FJ,

Martinez-Zorzano VS. Decreased expression of alpha-L-fucosidase gene FUCA1 in human colorectal tumors. Int J Mol Sci. 2013; 14:16986–16998.

46. Yuan K, Kucik D, Singh RK, Listinsky CM, Listinsky JJ, Siegal GP. Alterations in human breast cancer adhesion-motility in response to changes in cell surface glycoproteins displaying alpha-L-fucose moieties. Int J Oncol. 2008; 32:797–807.

47. Blanco MA, Aleckovic M, Hua Y, Li T, Wei Y, Xu Z, Cristea IM, Kang Y. Identification of staphylococcal nuclease domain-containing 1 (SND1) as a Metadherin-interacting protein with metastasis-promoting functions. J Biol Chem. 2011; 286:19982–19992.

48. Kuruma H, Kamata Y, Takahashi H, Igarashi K, Kimura T, Miki K, Miki J, Sasaki H, Hayashi N, Egawa S. Staphylococcal nuclease domain-containing protein 1 as a potential tissue marker for prostate cancer. Am J Pathol. 2009; 174:2044–2050.

49. Voisin SN, Krakovska O, Matta A, DeSouza LV, Romaschin AD, Colgan TJ, Siu KW. Identification of novel molecular targets for endometrial cancer using a drill-down LC-MS/MS approach with iTRAQ. PLoS One. 2011; 6:e16352.

50. Li Z, Huang C, Bai S, Pan X, Zhou R, Wei Y, Zhao X. Prognostic evaluation of epidermal fatty acid-binding protein and calcyphosine, two proteins implicated in endometrial cancer using a proteomic approach. Int J Cancer. 2008; 123:2377–2383.

51. Mojica W, Hawthorn L. Normal colon epithelium: a dataset for the analysis of gene expression and alternative splicing events in colon disease. BMC Genomics. 2010; 11: 5.

52. Harisi R, Kenessey I, Olah JN, Timar F, Babo I, Pogany G, Paku S, Jeney A. Differential inhibition of single and cluster type tumor cell migration. Anticancer Res. 2009; 29:2981–2985.

53. Wellman TL, Eckenstein M, Wong C, Rincon M, Ashikaga T, Mount SL, Francklyn CS, Lounsbury KM. Threonyl-tRNA synthetase overexpression correlates with angiogenic markers and progression of human ovarian cancer. BMC Cancer. 2014; 14: 620.

54. Sudo H, Tsuji AB, Sugyo A, Kohda M, Sogawa C, Yoshida C, Harada YN, Hino O, Saga T. Knockdown of COPA, identified by loss-of-function screen, induces apoptosis and suppresses tumor growth in mesothelioma mouse model. Genomics. 2010; 95:210–216.

55. Maresh EL, Mah V, Alavi M, Horvath S, Bagryanova L, Liebeskind ES, Knutzen LA, Zhou Y, Chia D, Liu AY, Goodglick L. Differential expression of anterior gradient gene AGR2 in prostate cancer. BMC Cancer. 2010; 10: 680.

56. Wang Z, Hao Y, Lowe AW. The adenocarcinoma-associated antigen, AGR2, promotes tumor growth, cell migration, and cellular transformation. Cancer Res. 2008; 68:492–497.

57. Yang M, Sun H, Wang H, Zhang S, Yu X, Zhang L. Down-regulation of ribosomal protein L22 in non-small cell lung cancer. Med Oncol. 2013; 30: 646.

58. Lin SJ, Chang KP, Hsu CW, Chi LM, Chien KY, Liang Y, Tsai MH, Lin YT, Yu JS. Low-molecular-mass secretome profiling identifies C-C motif chemokine 5 as a potential plasma biomarker and therapeutic target for nasopharyngeal carcinoma. J Proteomics. 2013; 94:186–201.

59. Fan Y, Guo Y. Knockdown of eIF3D inhibits breast cancer cell proliferation and invasion through suppressing the Wnt/beta-catenin signaling pathway. Int J Clin Exp Pathol. 2015; 8:10420–10427.

60. Lin Z, Xiong L, Lin Q. Knockdown of eIF3d inhibits cell proliferation through G2/M phase arrest in non-small cell lung cancer. Med Oncol. 2015; 32: 183.

61. Pan XW, Chen L, Hong Y, Xu DF, Liu X, Li L, Huang Y, Cui LM, Gan SS, Yang QW, Huang H, Qu FJ, Ye JQ, et al. EIF3D silencing suppresses renal cell carcinoma tumorigenesis via inducing G2/M arrest through downregulation of Cyclin B1/CDK1 signaling. Int J Oncol. 2016; 48:2580–2590.

62. Rentoft M, Lindell K, Tran P, Chabes AL, Buckland RJ, Watt DL, Marjavaara L, Nilsson AK, Melin B, Trygg J, Johansson E, Chabes A. Heterozygous colon cancer-associated mutations of SAMHD1 have functional significance. Proc Natl Acad Sci USA. 2016; 113:4723–4728.

63. Shaikh D, Zhou Q, Chen T, Ibe JC, Raj JU, Zhou G. cAMP-dependent protein kinase is essential for hypoxia-mediated epithelial-mesenchymal transition, migration, and invasion in lung cancer cells. Cell Signal. 2012; 24:2396–2406.

64. Williams PD, Owens CR, Dziegielewski J, Moskaluk CA, Read PW, Larner JM, Story MD, Brock WA, Amundson SA, Lee JK, Theodorescu D. Cyclophilin B expression is associated with in vitro radioresistance and clinical outcome after radiotherapy. Neoplasia. 2011; 13:1122–1131.

65. Huang SK, Darfler MM, Nicholl MB, You J, Bemis KG, Tegeler TJ, Wang M, Wery JP, Chong KK, Nguyen L, Scolyer RA, Hoon DS. LC/MS-based quantitative proteomic analysis of paraffin-embedded archival melanomas reveals potential proteomic biomarkers associated with metastasis. PLoS One. 2009; 4: e4430.

66. Shukla A, Edwards R, Yang Y, Hahn A, Folkers K, Ding J, Padmakumar VC, Cataisson C, Suh KS, Yuspa SH. CLIC4 regulates TGF-beta-dependent myofibroblast differentiation to produce a cancer stroma. Oncogene. 2013.

67. Suh KS, Crutchley JM, Koochek A, Ryscavage A, Bhat K, Tanaka T, Oshima A, Fitzgerald P, Yuspa SH. Reciprocal modifications of CLIC4 in tumor epithelium and stroma mark malignant progression of multiple human cancers. Clin Cancer Res. 2007; 13:121–131.

68. Yao Q, Qu X, Yang Q, Wei M, Kong B. CLIC4 mediates TGF-beta1-induced fibroblast-to-myofibroblast trans differentiation in ovarian cancer. Oncol Rep. 2009; 22:541–548.

69. Chiang PC, Chou RH, Chien HF, Tsai T, Chen CT. Chloride intracellular channel 4 involves in the reduced invasiveness of cancer cells treated by photodynamic therapy. Lasers Surg Med. 2013; 45:38–47.

70. Shiao YM, Chang YH, Liu YM, Li JC, Su JS, Liu KJ, Liu YF, Lin MW, Tsai SF. Dysregulation of GIMAP genes in non-small cell lung cancer. Lung Cancer. 2008; 62:287–294.

71. Li C, Liu VW, Chiu PM, Chan DW, Ngan HY. Over-expressions of AMPK subunits in ovarian carcinomas with significant clinical implications. BMC Cancer. 2012; 12:357.

72. Sakabe T, Tsuchiya H, Kanki K, Azumi J, Gonda K, Mizuta Y, Yamada D, Wada H, Shomori K, Nagano H, Shiota G. Identification of the genes chemosensitizing hepatocellular carcinoma cells to interferon-alpha/5-fluorouracil and their clinical significance. PLoS One. 2013; 8: e56197.

73. Tian SY, Chen SH, Shao BF, Cai HY, Zhou Y, Zhou YL, Xu AB. Expression of leucine aminopeptidase 3 (LAP3) correlates with prognosis and malignant development of human hepatocellular carcinoma (HCC). Int J Clin Exp Pathol. 2014; 7:3752–3762.

74. Wang X, Shi L, Deng Y, Qu M, Mao S, Xu L, Xu W, Fang C. Inhibition of leucine aminopeptidase 3 suppresses invasion of ovarian cancer cells through down-regulation of fascin and MMP-2/9. Eur J Pharmacol. 2015; 768:116–122.

75. Craven RA, Stanley AJ, Hanrahan S, Totty N, Jackson DP, Popescu R, Taylor A, Frey J, Selby PJ, Patel PM, Banks RE. Identification of proteins regulated by interferon-alpha in resistant and sensitive malignant melanoma cell lines. Proteomics. 2004; 4:3998–4009.

76. Paley EL, Paley DE, Merkulova-Rainon T, Subbarayan PR. Hypoxia signature of splice forms of tryptophanyl-tRNA synthetase marks pancreatic cancer cells with distinct metastatic abilities. Pancreas. 2011; 40:1043–1056.

77. Jonas SK, Benedetto C, Flatman A, Hammond RH, Micheletti L, Riley C, Riley PA, Spargo DJ, Zonca M, Slater TF. Increased activity of 6-phosphogluconate dehydrogenase and glucose-6-phosphate dehydrogenase in purified cell suspensions and single cells from the uterine cervix in cervical intraepithelial neoplasia. Br J Cancer. 1992; 66:185–191.

78. Takemasa I, Kittaka N, Hitora T, Watanabe M, Matsuo E, Mizushima T, Ikeda M, Yamamoto H, Sekimoto M, Nishimura O, Doki Y, Mori M. Potential biological insights revealed by an integrated assessment of proteomic and transcriptomic data in human colorectal cancer. Int J Oncol. 2012; 40:551–559.

79. Newnham GM, Conron M, McLachlan S, Dobrovic A, Do H, Li J, Opeskin K, Thompson N, Wright GM, Thomas DM. Integrated mutation, copy number and expression profiling in resectable non-small cell lung cancer. BMC Cancer. 2011; 11:93.

80. Crnogorac-Jurcevic T, Efthimiou E, Capelli P, Blaveri E, Baron A, Terris B, Jones M, Tyson K, Bassi C, Scarpa A, Lemoine NR. Gene expression profiles of pancreatic cancer and stromal desmoplasia. Oncogene. 2001; 20:7437–7446.

Supplementary Table 1: Categorization of proteomics findings based on available literature

Group	Protein Name [References]
Known bladder cancer associated proteins	1. Thymidine phosphorylase [1]<br>2. Alpha-actinin-4 [2, 3]<br>3. Alpha-actinin-1 [4]<br>4. 15-hydroxyprostaglandin dehydrogenase [NAD(+)] [5]<br>5. Annexin A1 [6]<br>6. Annexin A10 [7]<br>7. Membrane-associated progesterone receptor component 1 [8]<br>8. Transforming growth factor-beta-induced protein ig-h3 [9]<br>9. Cadherin 13 [10]<br>10. Cathepsin E [11]<br>11. Signal transducer and activator of transcription 1-alpha/beta [12]<br>12. Glutathione S-transferase Mu 1 [13, 14]<br>13. Gamma-synuclein [15-17]<br>14. Annexin A5 [18, 19]<br>15. Keratin, type I cytoskeletal 19 [20-22]<br>16. Keratin, type I cytoskeletal 7 [23, 24]<br>17. Keratin, type I cytoskeletal 8 [25]<br>18. Glutathione S-transferase Mu 3[26, 27]<br>19. Annexin A2 [28, 29]<br>20. Epoxide hydrolase 1 [30]<br>21. Protein S100-A4 [16, 31-33]<br>22. Glutathione S-transferase Mu 4 [34]<br>23. Macrophage migration inhibitory factor [35, 36]<br>24. Adipogenesis regulatory factor [20]<br>25. Cytochrome c oxidase subunit 6B1<br>26. Vasodilator-stimulated phosphoprotein [37]<br>27. DNA replication licensing factor MCM2 [38, 39]<br>28. Annexin A6 [40]<br>29. 26S proteasome non-ATPase regulatory subunit 12 [41]
Proteins described in the context of other cancers yet not in BC	1. Fibulin-2 [42, 43]<br>2. Transmembrane glycoprotein NMB [44]<br>3. Tissue alpha-L-fucosidase [45, 46]<br>4. Staphylococcal nuclease domain-containing protein 1 [47, 48]<br>5. Calcyphosin [49, 50]<br>6. Calcium-activated chloride channel regulator 4 [51]<br>7. Threonine--tRNA ligase, cytoplasmic [52, 53]<br>8. Coatomer subunit alpha [54]<br>9. Anterior gradient protein 2 homolog, AGR2 [55, 56]<br>10. 60S ribosomal protein L22 [57, 58]<br>11. Eukaryotic translation initiation factor 3 subunit D [59-61]<br>12. Deoxynucleoside triphosphate triphosphohydrolase SAMHD1 [62]<br>13. cAMP-dependent protein kinase catalytic subunit alpha (P17612) [63]<br>14. Peptidyl- prolyl cis-trans isomerase B [64, 65]<br>15. Chloride intracellular channel protein 4 [66-69]<br>16. GTPase IMAP family member 4 [70]<br>17. 5'-AMP-activated protein kinase subunit gamma-2 [71, 72]<br>18. Cytosol aminopeptidase [73, 74]<br>19. Tryptophan-tRNA ligase, cytoplasmic [75, 76]<br>20. 6-phosphogluconate dehydrogenase, decarboxylating [77]<br>21. 40S ribosomal proteins S18 [78]<br>22. 40S ribosomal protein S9 [79, 80]
Potentially novel finding not yet described in bladder or other type of malignancy	1. BRO I domain-containing protein BROX<br>2. Signal peptidase complex subunit 3<br>3. Vesicle-trafficking protein SEC22b<br>4. RNA 3'-terminal phosphate cyclase<br>5. Proteasome subunit beta type-3<br>6. Trans-Golgi network integral membrane protein 2<br>7. Transmembrane protein 33<br>8. Keratin, type II cytoskeletal 79<br>9. Coiled-coil domain containing protein 58

Sixty differentially present proteins ($p < 0.05$), as defined by both analytical workflows (overlap presented on Venn diagram – Figure 1), were thoroughly evaluated in the context of available literature.

Supplementary Table 2: Shortlist of 144 differentially expressed proteins identified by shotgun proteomics analysis of BC tissue specimens. Proteins found to be differentially expressed at statistically significant levels in both approaches are marked in bold. Changes in the protein expression level are represented by fold change (pT2+ vs. pTa). See Supplementary_Table_2

Supplementary Table 3: Full list of proteins identified using (A) Proteome Discoverer and (B) Trans-Proteomic Pipeline. See Supplementary_Table_3

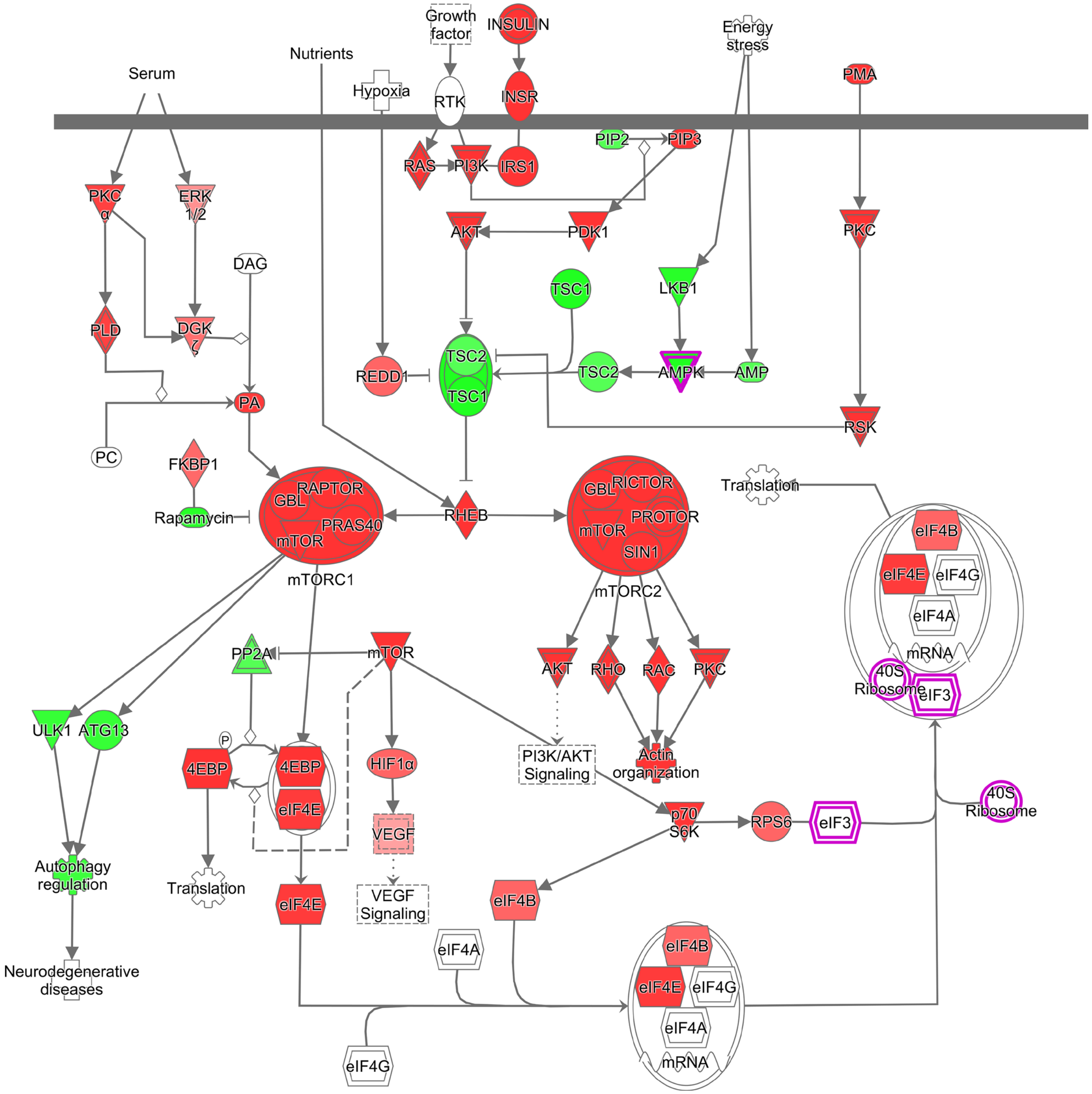

Supplementary Figure 1: mTOR signaling overlaid with the expected activation state using Ingenuity Pathway Analysis. The analysis was performed using Molecule Activity Predictor (MAP), which allows to predict the upstream/downstream effects of activation/inhibition of molecules in the pathway. According to the predicted activation state, when the mTOR pathway is activated, RHEB protein is expected to be up-regulated. Molecules predicted to be up-regulated when the pathway is activated are marked in red, while those predicted to be down-regulated are marked in green. Molecules identified by the proteomics experiments are marked in purple frame.

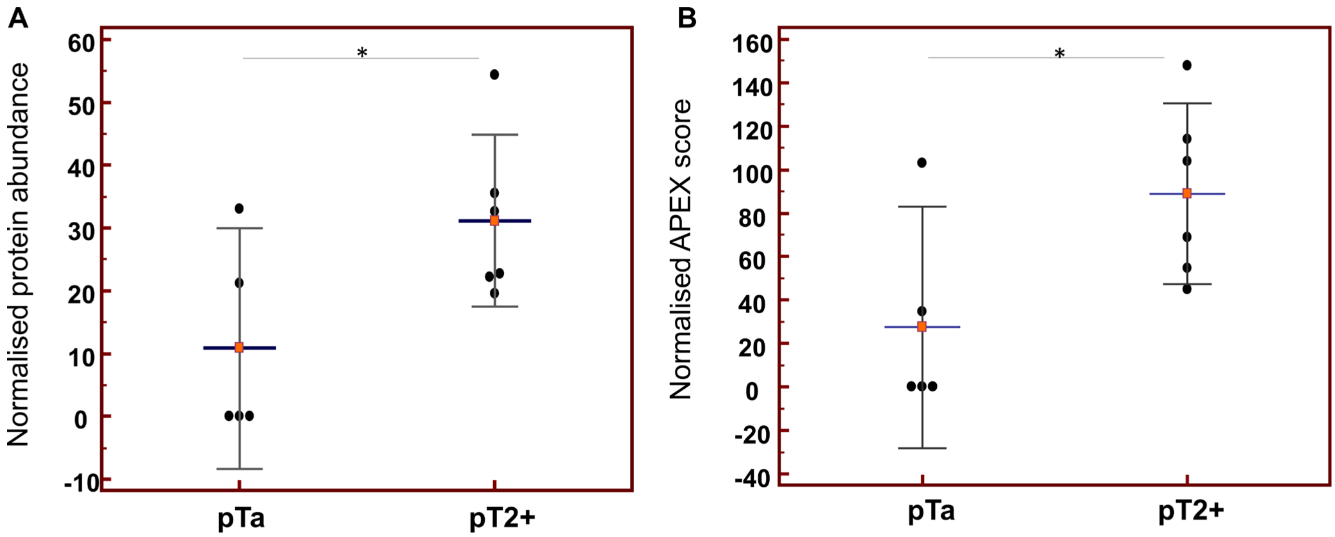

Supplementary Figure 2: Dot plot presenting the abundance of EIF3D in human samples based on the LC-MS/MS data. The protein abundance was calculated based on (**A**) the average area of the top 3 peptides for dataset analyzed using Proteome Discoverer and (**B**) the Apex score for dataset analyzed using Trans Proteomics Pipeline.

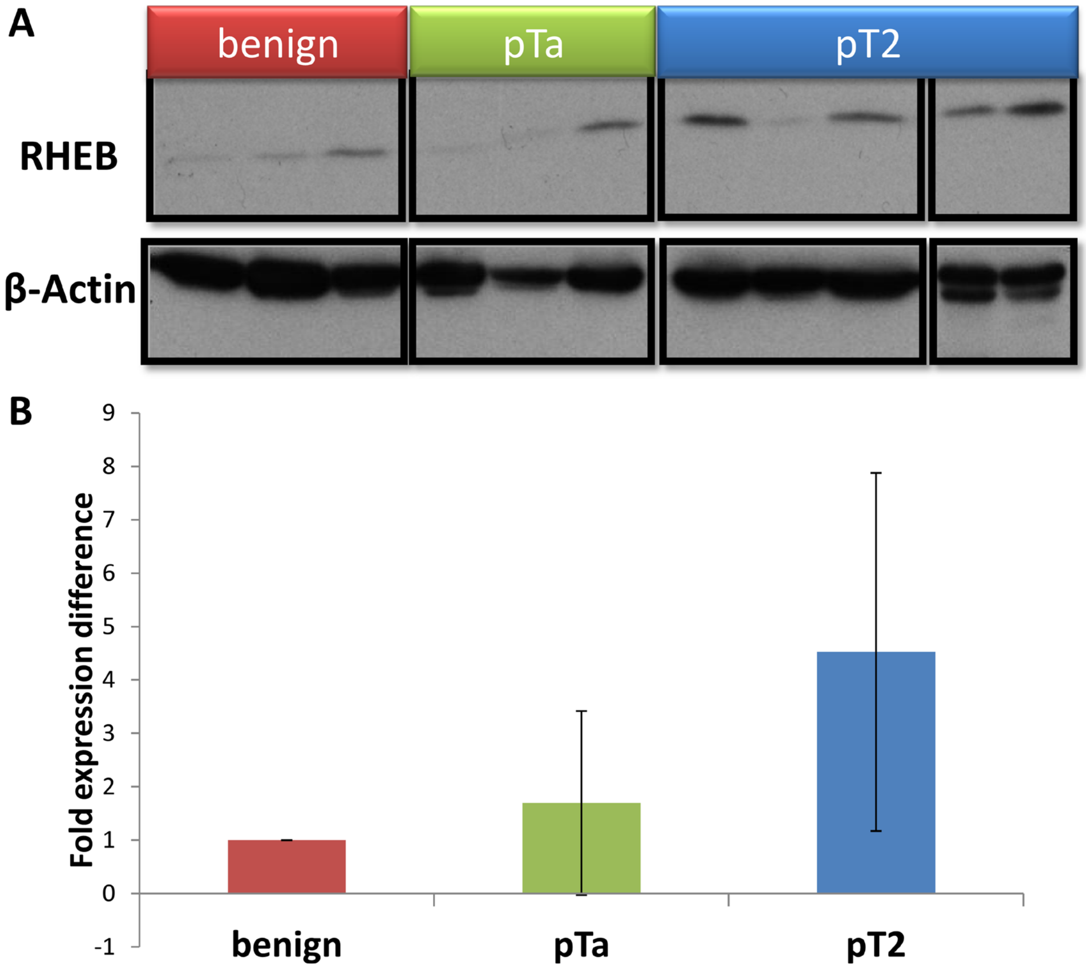

Supplementary Figure 3: Western blot analysis for RHEB in bladder cancer tissue specimens. (**A**) Western blot analysis for RHEB in protein extracts from benign (three different samples), pTa (three different samples) and pT2 (five different samples) tumors. β-Actin was used as loading control. (**B**) The quantification of the proteins was performed by using the Quantity One software and the results were normalized to β-Actin loading control and then to benign samples. The values represent the means ± SD from the different samples.

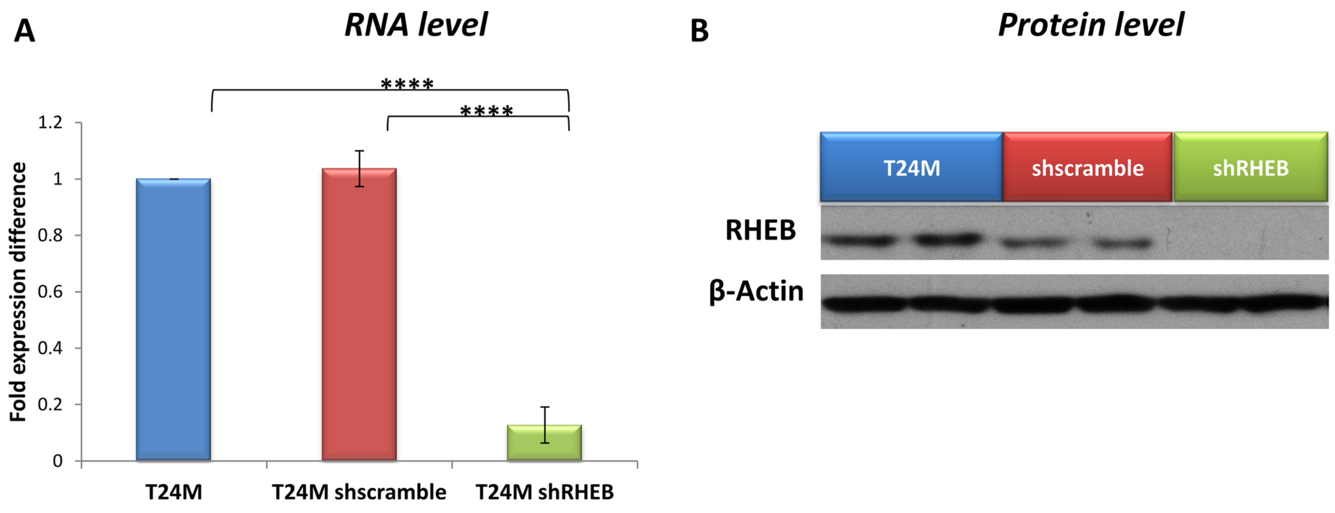

Supplementary Figure 4: Knockdown of RHEB in T24M cells. (**A**) Bar graph representing the decreased expression levels of RHEB in T24M shRHEB cells in comparison with T24M shscramble and untransduced T24M cells analysed by real-time PCR. The knockdown of RHEB in T24M cells resulted in 87.7% ± 5.6% reduction of its mRNA levels in T24M shRHEB cells compared to T24M shscramble ($p \leq 0.0001$, Student's *t*-test). The real-time PCR data were normalized to the human GAPDH reference gene and then to the control T24M untransduced cells. (**B**) Western blot analysis for RHEB in cell extracts from T24M, T24M shscramble and T24M shRHEB cells. The knockdown of RHEB in T24M cells resulted in a 100% reduction at the protein level ($p \leq 0.0001$, Student's *t*-test). The values represent the means ± SD from three independent experiments performed in duplicate (two-tailed Student's *t*-test, ****$p \leq 0.0001$).

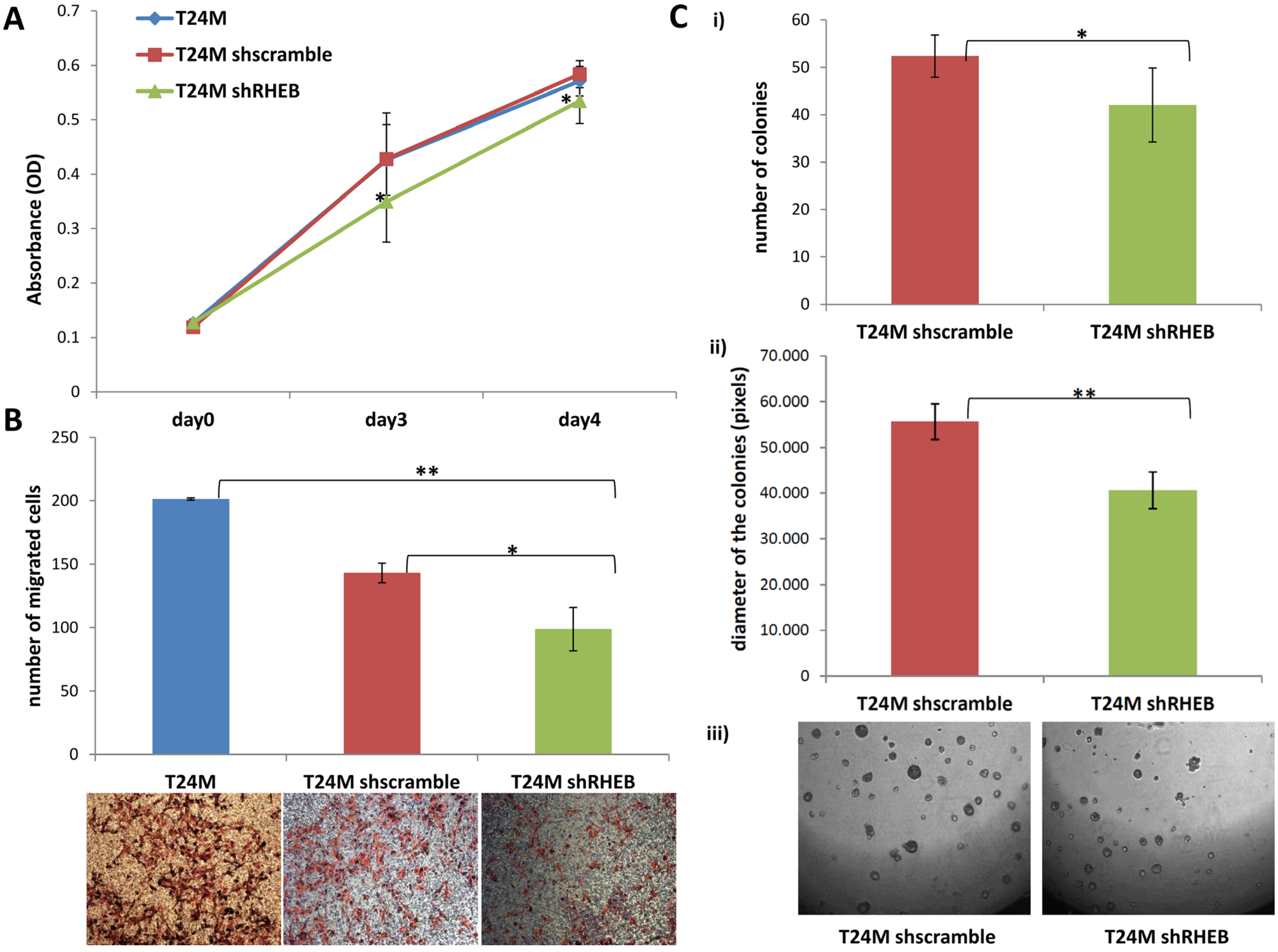

Supplementary Figure 5: The knockdown of RHEB reduces cell proliferation, migration and colony forming ability of T24M cells. (**A**) Knocking down of RHEB resulted in a 28% reduction in the proliferation rate of T24M shRHEB cells compared to T24M shscramble cells after 3-day culture (0.35 ± 0.074au versus 0.428 ± 0.085au; $p \leq 0.05$, Student's *t*-test) and in 12% decrease after 4 days (0.534 ± 0.041au versus 0.584 ± 0.025au; $p \leq 0.05$, Student's *t*-test); normalized to Day 0 (0.119 ± 0.009au for T24M shscramble, 0.127 ± 0.005au for T24M shRHEB). The values represent the means ± SD from two independent experiments performed in five replicates (two-tailed Student's *t*-test, *$p \leq 0.05$). (**B**) The migratory capacity of T24M cells decreased upon RHEB knockdown in T24M shRHEB cells compared to T24M shscramble cells (99 ± 17 cells versus 143 ± 8 cells, respectively; $p \leq 0.05$, Student's *t*-test). The graph illustrates the number of the cells migrated toward conditioned media derived from T24M cells. The cells were allowed to migrate for 6h toward the conditioned medium. Representative images of the migrated cells from each condition are displayed below the graph. Magnification: 10×. The values represent the means ± SD from two independent experiments performed in duplicate (two-tailed Student's *t*-test, *$p \leq 0.05$, **$p \leq 0.01$). (**C**) The downregulation of RHEB also decreased the colony formation ability of T24M cells after 10 days of growth on matrigel. Both i) the number (42 ± 8 in T24M shRHEB versus 52 ± 4 in T24M shscramble; $p \leq 0.05$, Student's *t*-test) and ii) the diameter of the colonies (40.567 ± 4.032 pixels in T24M shRHEB versus 55.631 ± 3.894pixels in T24M shscramble; $p \leq 0.01$, Student's *t*-test) were reduced upon RHEB knockdown. The diameters of the colonies were calculated on ImageJ software and their diameter was given in pixels. iii) Representative images of the colonies formed by T24M shscramble and T24M shRHEB cells. Magnification: 5×. The values represent the means ± SD from two independent experiments performed in duplicate (two-tailed Student's *t*-test, *$p \leq 0.05$, **$p \leq 0.01$).

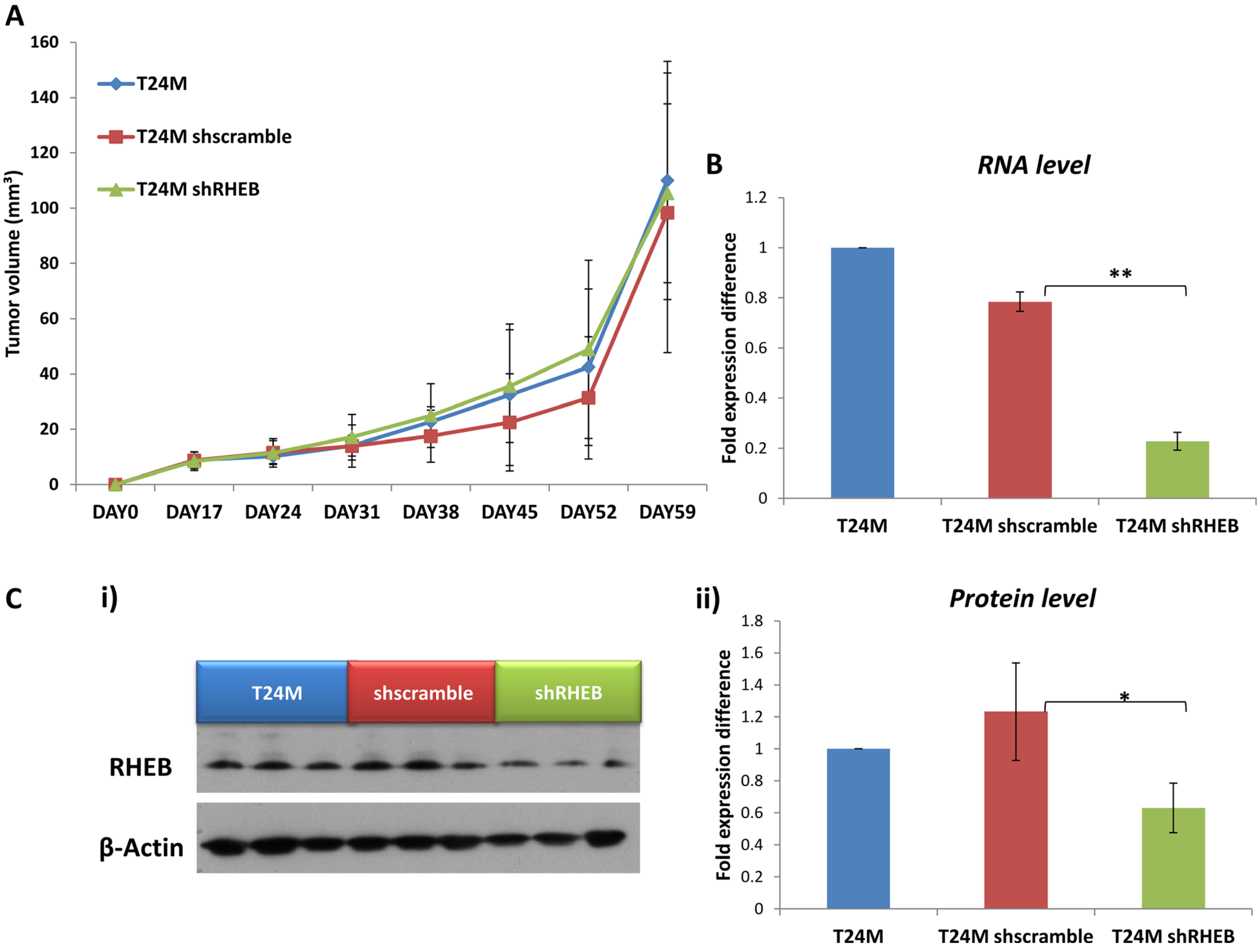

Supplementary Figure 6: The knockdown of RHEB has no impact on tumor growth *in vivo*. (**A**) Tumor growth in T24M, T24M shscramble and T24M shRHEB tumor bearing NOD/SCID mice. No statistically significant difference was observed in the tumor volume of T24M shRHEB group (105.93 ± 32.37 mm^3) compared with T24M (110.03 ± 43.13mm^3; $p \geq 0.05$, Student's t-test) or T24M shscramble (98.32 ± 50.59mm^3; $p \geq 0.05$, Student's t-test) tumor bearing mice. (**B**) The expression of RHEB was investigated in excised tumors from all groups of mice, 60 days after the injections, at the RNA level. RHEB was significantly reduced at the mRNA level by 71% ± 3.1% in T24M shRHEB tumors compared to T24M shscramble (**$p \leq 0.01$, Student's t-test). No significant difference was observed between the T24M and the T24M shscramble tumors. (**C**) The knockdown of RHEB in the tumors was also examined at the protein level 60 days after the injections in a different set of tumors from the one that was used for the RNA expression analysis. i) Western blot analysis for RHEB in T24M, T24M shscramble and T24M shRHEB tumors. ii) The levels of RHEB were found decreased by 47.82% ± 10.44% in T24M shRHEB tumors compared to T24M shscramble (*$p = 0.05$, Student's t-test) with no significant difference between the T24M and the T24M shscramble tumors ($p = 0.32$, Student's t-test).
