# Supplementary material for: Proteomics analysis of bladder cancer invasion: Targeting EIF3D for therapeutic intervention
Source: Oncotarget. 2017 Apr 20;8(41):69435–55. doi: 10.18632/oncotarget.17279 (PMC5642490; doi:10.18632/oncotarget.17279)
Supplement: Supplementary file 2 [file oncotarget-08-69435-s002.doc]

Supplementary Table 2. Shortlist of 144 differentially expressed proteins identified by shotgun proteomics analysis of BC tissue specimens. Proteins found to be differentially expressed at statistically significant levels in both approaches are marked in bold. Changes in the protein expression level are represented by fold change (pT2+ vs. pTa).

| **Uniprot Accession** | **Uniprot ID** | **Protein name** | **Proteome Discoverer** | | **Trans Proteomic Pipeline** | |
| --- | --- | --- | --- | --- | --- | --- |
|  |  |  | **Fold Change (pT2+/pTa)** | **p-value** | **Fold Change (pT2+/pTa)** | **p-value** |
|  |  |  |  |  |  |  |
| **Proteins Up-regulated in pT2+ versus pTa** | | |  |  |  |  |
| **P17612** | **KAPCA_HUMAN** | **cAMP-dependent protein kinase catalytic subunit alpha** | **Only pT2+** | **0.01** | **Only pT2+** | ***p<*0.01** |
| **Q14956** | **GPNMB_HUMAN** | **Transmembrane glycoprotein NMB** | **Only pT2+** | **0.03** | **Only pT2+** | ***p<*0.01** |
| **P57088** | **TMM33_HUMAN** | **Transmembrane protein 33** | **Only pT2+** | **0.01** | **Only pT2+** | **0.01** |
| **P07099** | **HYEP_HUMAN** | **Epoxide hydrolase 1** | **Only pT2+** | **0.03** | **Only pT2+** | **0.01** |
| **Q9Y3Z3** | **SAMH1_HUMAN** | **Deoxynucleoside triphosphate triphosphohydrolase SAMHD1** | **Only pT2+** | **0.03** | **104.33** | **0.03** |
| **O00442** | **RTCA_HUMAN** | **RNA 3'-terminal phosphate cyclase** | **Only pT2+** | **0.03** | **Only pT2+** | **0.03** |
| **P49736** | **MCM2_HUMAN** | **DNA replication licensing factor MCM2** | **Only pT2+** | **0.03** | **Only pT2+** | **0.03** |
| **P98095** | **FBLN2_HUMAN** | **Fibulin-2** | **Only pT2+** | **0.03** | **Only pT2+** | **0.03** |
| **Q9NUV9** | **GIMA4_HUMAN** | **GTPase IMAP family member 4** | | **Only pT2+** | | --- | | **0.03** | **Only pT2+** | **0.03** |
| **Q9Y696** | **CLIC4_HUMAN** | **Chloride intracellular channel protein 4** | **34.96** | **0.02** | **14.81** | **0.01** |
| **P19971** | **TYPH_HUMAN** | **Thymidine phosphorylase** | **13.24** | **0.02** | **14.94** | **0.04** |
| **P42224** | **STAT1_HUMAN** | **Signal transducer and activator of transcription 1-alpha/beta** | **7.37** | **0.02** | **15.71** | ***p<*0.05** |
| **Q15582** | **BGH3_HUMAN** | **Transforming growth factor-beta-induced protein ig-h3** | **6.84** | **0.01** | **5.95** | **0.01** |
| **P08133** | **ANXA6_HUMAN** | **Annexin A6** | **5.73** | **0.04** | **5.77** | ***p<*0.05** |
| **P23381** | **SYWC_HUMAN** | **Tryptophan--tRNA ligase, cytoplasmic** | **5.51** | **0.04** | **11.29** | ***p<*0.05** |
| **P28838** | **AMPL_HUMAN** | **Cytosol aminopeptidase** | **4.63** | **0.03** | **4.06** | ***p<*0.05** |
| **P26639** | **SYTC_HUMAN** | **Threonine--tRNA ligase, cytoplasmic** | **4.24** | ***p<*0.05** | **7.98** | **0.01** |
| **P61009** | **SPCS3_HUMAN** | **Signal peptidase complex subunit 3** | **4.18** | **0.02** | **4.88** | **0.01** |
| **Q7KZF4** | **SND1_HUMAN** | **Staphylococcal nuclease domain-containing protein 1** | **4.08** | **0.04** | **5.21** | ***p<*0.01** |
| **O00232** | **PSD12_HUMAN** | **26S proteasome non-ATPase regulatory subunit 12** | **3.82** | **0.03** | **5.28** | ***p<*0.05** |
| **O43493** | **TGON2_HUMAN** | **Trans-Golgi network integral membrane protein 2** | **3.72** | **0.01** | **4.67** | ***p<*0.01** |
| **P49720** | **PSB3_HUMAN** | **Proteasome subunit beta type-3** | **3.28** | **0.02** | **5.48** | ***p<*0.01** |
| **P04083** | **ANXA1_HUMAN** | **Annexin A1** | **3.25** | **0.01** | **2.46** | **0.01** |
| **P52209** | **6PGD_HUMAN** | **6-phosphogluconate dehydrogenase, decarboxylating** | **3.00** | **0.04** | **2.52** | **0.01** |
| **O15371** | **EIF3D_HUMAN** | **Eukaryotic translation initiation factor 3 subunit D** | **2.87** | **0.04** | **3.23** | **0.04** |
| **P08758** | **ANXA5_HUMAN** | **Annexin A5** | **2.82** | **0.01** | **2.12** | **0.02** |
| **O75396** | **SC22B_HUMAN** | **Vesicle-trafficking protein SEC22b** | **2.77** | **0.02** | **2.27** | **0.01** |
| **P53621** | **COPA_HUMAN** | **Coatomer subunit alpha** | **2.76** | **0.02** | **5.07** | **0.01** |
| **P12814** | **ACTN1_HUMAN** | **Alpha-actinin-1** | **2.13** | **0.03** | **3.18** | **0.03** |
| **O43707** | **ACTN4_HUMAN** | **Alpha-actinin-4** | **2.09** | **0.03** | **1.67** | **0.02** |
| **P46781** | **RS9_HUMAN** | **40S ribosomal protein S9** | **1.90** | ***p<*0.05** | **1.64** | **0.02** |
| **P07355** | **ANXA2_HUMAN** | **Annexin A2** | **1.71** | **0.02** | **1.49** | **0.01** |
| **P62269** | **RS18_HUMAN** | **40S ribosomal protein S18** | **1.64** | **0.03** | **1.46** | **0.03** |
| **P35268** | **RL22_HUMAN** | **60S ribosomal protein L22** | **1.51** | ***p<*0.05** | **1.51** | ***p<*0.05** |
| **P23284** | **PPIB_HUMAN** | **Peptidyl-prolyl cis-trans isomerase B** | **1.44** | **0.01** | **1.41** | ***p<*0.05** |
| P02792 | FRIL_HUMAN | Ferritin light chain | 43.44 | 0.08 | 9.69 | 0.03 |
| Q9Y3I0 | RTCB_HUMAN | tRNA-splicing ligase RtcB homolog | 2.61 | 0.17 | 4.06 | *p<*0.01 |
| O43852 | CALU_HUMAN | Calumenin | 2.92 | 0.36 | 3.75 | 0.01 |
| P49721 | PSB2_HUMAN | Proteasome subunit beta type-2 | 1.45 | 0.19 | 3.66 | 0.01 |
| P49257 | LMAN1_HUMAN | Protein ERGIC-53 | 2.26 | 0.10 | 4.50 | 0.01 |
| P07237 | PDIA1_HUMAN | Protein disulfide-isomerase | 1.58 | 0.06 | 1.58 | 0.01 |
| P05164 | PERM_HUMAN | Myeloperoxidase | 23.69 | 0.14 | Only pT2+ | 0.01 |
| P32455 | GBP1_HUMAN | Interferon-induced guanylate-binding protein 1 | 2.06 | 0.48 | Only pT2+ | 0.01 |
| P46776 | RL27A_HUMAN | 60S ribosomal protein L27a | 1.55 | 0.20 | 1.93 | 0.01 |
| P12004 | PCNA_HUMAN | Proliferating cell nuclear antigen | 3.20 | 0.06 | 3.68 | 0.02 |
| Q96CX2 | KCD12_HUMAN | BTB/POZ domain-containing protein KCTD12 | 4.30 | 0.07 | 15.88 | 0.02 |
| P62263 | RS14_HUMAN | 40S ribosomal protein S14 | 1.54 | 0.13 | 2.14 | 0.02 |
| P04844 | RPN2_HUMAN | Dolichyl-diphosphooligosaccharide--protein glycosyltransferase subunit 2 | 1.74 | 0.08 | 2.41 | 0.02 |
| P62899 | RL31_HUMAN | 60S ribosomal protein L31 | 1.79 | 0.28 | 3.17 | 0.03 |
| Q9Y678 | COPG1_HUMAN | Coatomer subunit gamma-1 | 2.79 | 0.11 | 6.26 | 0.03 |
| P25787 | PSA2_HUMAN | Proteasome subunit alpha type-2 | 2.46 | 0.11 | 2.68 | 0.03 |
| P35580 | MYH10_HUMAN | Myosin-10 | 1.86 | 0.11 | 2.75 | 0.03 |
| Q15746 | MYLK_HUMAN | Myosin light chain kinase, smooth muscle | 8.66 | 0.06 | Only pT2+ | 0.03 |
| Q14194 | DPYL1_HUMAN | Dihydropyrimidinase-related protein 1 | 5.84 | 0.10 | Only pT2+ | 0.03 |
| P63267 | ACTH_HUMAN | Actin, gamma-enteric smooth muscle | 1.53 | 0.12 | Only pT2+ | 0.03 |
| P62277 | RS13_HUMAN | 40S ribosomal protein S13 | 1.73 | 0.12 | 2.02 | 0.04 |
| O95782 | AP2A1_HUMAN | AP-2 complex subunit alpha-1 | 1.81 | 0.13 | 3.87 | 0.04 |
| O15144 | ARPC2_HUMAN | Actin-related protein 2/3 complex subunit 2 | 1.51 | 0.31 | 1.94 | 0.04 |
| P25205 | MCM3_HUMAN | DNA replication licensing factor MCM3 | 13.08 | 0.06 | 13.95 | 0.04 |
| Q15063 | POSTN_HUMAN | Periostin | 13.98 | 0.06 | 18.61 | *p<*0.05 |
| P13797 | PLST_HUMAN | Plastin-3 | 1.88 | 0.14 | 2.03 | *p<*0.05 |
| P30740 | ILEU_HUMAN | Leukocyte elastase inhibitor | 3.51 | 0.25 | 10.33 | *p<*0.05 |
| Q16851 | UGPA_HUMAN | UTP--glucose-1-phosphate uridylyltransferase | 1.60 | 0.09 | 2.00 | *p<*0.05 |
| Q9Y295 | DRG1_HUMAN | Developmentally-regulated GTP-binding protein 1 | 2.71 | 0.26 | 10.44 | *p<*0.05 |
| P30050 | RL12_HUMAN | 60S ribosomal protein L12 | 1.48 | 0.10 | 1.61 | *p<*0.05 |
| P06899 | H2B1J_HUMAN | Histone H2B type 1-J | 1.52 | 0.11 | 1.48 | *p<*0.05 |
| O14818 | PSA7_HUMAN | Proteasome subunit alpha type-7 | 1.50 | 0.14 | 1.76 | *p<*0.05 |
| P24821 | TENA_HUMAN | Tenascin | Only pT2+ | 0.01 | 642.04 | 0.06 |
| P11413 | G6PD_HUMAN | Glucose-6-phosphate 1-dehydrogenase | Only pT2+ | *p<*0.01 | 31.46 | 0.08 |
| Q9Y6N5 | SQRD_HUMAN | Sulfide:quinone oxidoreductase, mitochondrial | Only pT2+ | 0.03 | 30.82 | 0.09 |
| P13727 | PRG2_HUMAN | Bone marrow proteoglycan | Only pT2+ | 0.01 | 65.14 | 0.10 |
| Q16527 | CSRP2_HUMAN | Cysteine and glycine-rich protein 2 | Only pT2+ | 0.03 | 7.86 | 0.12 |
| Q8WX93 | PALLD_HUMAN | Palladin | Only pT2+ | 0.03 | 28.92 | 0.12 |
| P16435 | NCPR_HUMAN | NADPH--cytochrome P450 reductase | Only pT2+ | 0.03 | 7.23 | 0.19 |
| P61421 | VA0D1_HUMAN | V-type proton ATPase subunit d 1 | Only pT2+ | 0.03 | 3.61 | 0.28 |
| P62906 | RL10A_HUMAN | 60S ribosomal protein L10a | 2.04 | 0.04 | 1.90 | 0.07 |
| P06396 | GELS_HUMAN | Gelsolin | 2.17 | 0.03 | 1.61 | 0.07 |
| P35609 | ACTN2_HUMAN | Alpha-actinin-2 | 2.00 | 0.02 | 6.86 | 0.07 |
| P07858 | CATB_HUMAN | Cathepsin B | 2.06 | *p<*0.05 | 1.73 | 0.07 |
| P40616 | ARL1_HUMAN | ADP-ribosylation factor-like protein 1 | 4.76 | 0.01 | 3.93 | 0.08 |
| P51911 | CNN1_HUMAN | Calponin-1 | 3.80 | *p<*0.05 | 4.54 | 0.09 |
| Q15631 | TSN_HUMAN | Translin | 7.55 | 0.01 | 2.58 | 0.10 |
| O60506 | HNRPQ_HUMAN | Heterogeneous nuclear ribonucleoprotein Q | 1.73 | *p<*0.05 | 1.97 | 0.10 |
| P60468 | SC61B_HUMAN | Protein transport protein Sec61 subunit beta | 7.26 | 0.02 | 2.41 | 0.12 |
| P61077 | UB2D3_HUMAN | Ubiquitin-conjugating enzyme E2 D3 | 4.88 | 0.04 | 4.83 | 0.12 |
| P08754 | GNAI3_HUMAN | Guanine nucleotide-binding protein G(k) subunit alpha | 3.31 | 0.03 | 1.91 | 0.18 |
| Q9Y6E2 | BZW2_HUMAN | Basic leucine zipper and W2 domain-containing protein 2 | 7.15 | 0.04 | 2.77 | 0.21 |
| P26640 | SYVC_HUMAN | Valine--tRNA ligase | 2.82 | 0.04 | 1.94 | 0.22 |
| P67812 | SC11A_HUMAN | Signal peptidase complex catalytic subunit SEC11A | 2.65 | *p<*0.05 | 1.81 | 0.29 |
| P07942 | LAMB1_HUMAN | Laminin subunit beta-1 | 9.11 | 0.03 | 2.15 | 0.49 |
| **Proteins Down-regulated in pT2+ versus pTa** | | |  |  |  |  |
| **P05787** | **K2C8_HUMAN** | **Keratin, type II cytoskeletal 8** | **0.45** | ***p<*0.05** | **0.56** | **0.02** |
| **Q9UGJ0** | **AAKG2_HUMAN** | **5'-AMP-activated protein kinase subunit gamma-2** | **0.42** | **0.02** | **0.32** | **0.01** |
| **Q5XKE5** | **K2C79_HUMAN** | **Keratin, type II cytoskeletal 79** | **0.40** | **0.02** | **0.58** | ***p<*0.05** |
| **P08729** | **K2C7_HUMAN** | **Keratin, type II cytoskeletal 7** | **0.37** | **0.04** | **0.58** | **0.03** |
| **O00264** | **PGRC1_HUMAN** | **Membrane-associated progesterone receptor component 1** | **0.36** | **0.01** | **0.37** | **0.03** |
| **P14174** | **MIF_HUMAN** | **Macrophage migration inhibitory factor** | **0.36** | **0.03** | **0.40** | **0.03** |
| **P08727** | **K1C19_HUMAN** | **Keratin, type I cytoskeletal 19** | **0.35** | **0.03** | **0.48** | **0.01** |
| **P26447** | **S10A4_HUMAN** | **Protein S100-A4** | **0.31** | **0.02** | **0.36** | **0.03** |
| **Q03013** | **GSTM4_HUMAN** | **Glutathione S-transferase Mu 4** | **0.24** | **0.02** | **0.18** | **0.03** |
| **P09488** | **GSTM1_HUMAN** | **Glutathione S-transferase Mu 1** | **0.23** | **0.02** | **0.28** | **0.02** |
| **Q13938** | **CAYP1_HUMAN** | **Calcyphosin** | **0.21** | ***p<*0.05** | **0.30** | **0.01** |
| **P50552** | **VASP_HUMAN** | **Vasodilator-stimulated phosphoprotein** | **0.21** | **0.01** | **0.26** | ***p<*0.01** |
| **P14854** | **CX6B1_HUMAN** | **Cytochrome c oxidase subunit 6B1** | **0.20** | ***p<*0.05** | **0.17** | **0.01** |
| **P21266** | **GSTM3_HUMAN** | **Glutathione S-transferase Mu 3** | **0.17** | ***p<*0.05** | **0.20** | **0.02** |
| **P04066** | **FUCO_HUMAN** | **Tissue alpha-L-fucosidase** | **0.16** | ***p<*0.01** | **0.31** | **0.04** |
| **O76070** | **SYUG_HUMAN** | **Gamma-synuclein** | **0.13** | **0.02** | **0.21** | **0.01** |
| **Q15847** | **ADIRF_HUMAN** | **Adipogenesis regulatory factor** | **0.12** | **0.01** | **0.15** | ***p<*0.05** |
| **P15428** | **PGDH_HUMAN** | **15-hydroxyprostaglandin dehydrogenase [NAD(+)]** | **0.01** | **0.04** | **0.02** | **0.01** |
| **O95994** | **AGR2_HUMAN** | **Anterior gradient protein 2 homolog** | **only pTa** | ***p<*0.01** | **0.02** | ***p<*0.01** |
| **Q5VW32** | **BROX_HUMAN** | **BRO1 domain-containing protein BROX** | **only pTa** | **0.01** | **only pTa** | **0.01** |
| **P14091** | **CATE_HUMAN** | **Cathepsin E** | **only pTa** | **0.04** | **only pTa** | **0.01** |
| **P55290** | **CAD13_HUMAN** | **Cadherin-13** | **only pTa** | **0.04** | **only pTa** | **0.04** |
| **Q14CN2** | **CLCA4_HUMAN** | **Calcium-activated chloride channel regulator 4** | **only pTa** | **0.04** | **only pTa** | **0.04** |
| **Q4VC31** | **CCD58_HUMAN** | **Coiled-coil domain-containing protein 58** | **only pTa** | **0.04** | **only pTa** | **0.04** |
| **Q9UJ72** | **ANX10_HUMAN** | **Annexin A10** | **only pTa** | **0.04** | **only pTa** | **0.04** |
| Q96A26 | F162A_HUMAN | Protein FAM162A | 0.65 | 0.38 | 0.33 | 0.02 |
| P07738 | PMGE_HUMAN | Bisphosphoglycerate mutase | 0.33 | 0.24 | 0.13 | 0.02 |
| Q9NYL9 | TMOD3_HUMAN | Tropomodulin-3 | 0.64 | 0.37 | 0.34 | 0.03 |
| Q9UHQ9 | NB5R1_HUMAN | NADH-cytochrome b5 reductase 1 | 0.27 | 0.06 | 0.11 | 0.03 |
| O43169 | CYB5B_HUMAN | Cytochrome b5 type B | 0.29 | 0.11 | 0.17 | 0.03 |
| P08779 | K1C16_HUMAN | Keratin, type I cytoskeletal 16 | 0.56 | 0.06 | 0.40 | 0.03 |
| Q8WUY1 | THEM6_HUMAN | Protein THEM6 | 0.40 | 0.31 | 0.20 | 0.04 |
| P12931 | SRC_HUMAN | Proto-oncogene tyrosine-protein kinase Src | 0.47 | 0.27 | only pTa | 0.04 |
| Q8TB22 | SPT20_HUMAN | Spermatogenesis-associated protein 20 | 0.35 | 0.26 | 0.21 | 0.04 |
| Q9BRA2 | TXD17_HUMAN | Thioredoxin domain-containing protein 17 | 0.30 | 0.16 | 0.38 | 0.04 |
| P30626 | SORCN_HUMAN | Sorcin | 0.40 | 0.09 | 0.51 | 0.04 |
| P15374 | UCHL3_HUMAN | Ubiquitin carboxyl-terminal hydrolase isozyme L3 | 0.23 | 0.12 | 0.20 | 0.04 |
| O00515 | LAD1_HUMAN | Ladinin-1 | 0.18 | 0.02 | 0.24 | 0.06 |
| Q9Y5K6 | CD2AP_HUMAN | CD2-associated protein | only pTa | 0.04 | 0.15 | 0.07 |
| P61604 | CH10_HUMAN | 10 kDa heat shock protein, mitochondrial | 0.52 | *p<*0.05 | 0.54 | 0.07 |
| Q8NFU3 | TSTD1_HUMAN | Thiosulfate sulfurtransferase/rhodanese-like domain-containing protein 1 | 0.04 | *p<*0.01 | 0.07 | 0.08 |
| Q13423 | NNTM_HUMAN | NAD(P) transhydrogenase, mitochondrial | only pTa | 0.04 | 0.07 | 0.08 |
| P69905 | HBA_HUMAN | Hemoglobin subunit alpha | 0.40 | 0.04 | 0.40 | 0.10 |
| Q32MZ4 | LRRF1_HUMAN | Leucine-rich repeat flightless-interacting protein 1 | 0.34 | 0.02 | 0.42 | 0.11 |
| O00757 | F16P2_HUMAN | Fructose-1,6-bisphosphatase isozyme 2 | only pTa | 0.04 | 0.12 | 0.12 |
| P46439 | GSTM5_HUMAN | Glutathione S-transferase Mu 5 | 0.23 | 0.02 | 0.36 | 0.13 |
| Q14376 | GALE_HUMAN | UDP-glucose 4-epimerase | only pTa | 0.04 | 0.15 | 0.14 |
| Q15843 | NEDD8_HUMAN | NEDD8 | only pTa | 0.04 | 0.16 | 0.14 |
| P19404 | NDUV2_HUMAN | NADH dehydrogenase [ubiquinone] flavoprotein 2, mitochondrial | only pTa | 0.04 | 0.27 | 0.16 |
| P28161 | GSTM2_HUMAN | Glutathione S-transferase Mu 2 | 0.22 | 0.01 | 0.38 | 0.18 |
| O95861 | BPNT1_HUMAN | 3'(2'),5'-bisphosphate nucleotidase 1 | 0.12 | 0.02 | 0.26 | 0.18 |
| O14737 | PDCD5_HUMAN | Programmed cell death protein 5 | only pTa | 0.04 | 0.04 | 0.19 |
| Q9H773 | DCTP1_HUMAN | dCTP pyrophosphatase 1 | only pTa | 0.04 | 0.41 | 0.28 |
| P0CG47 | UBB_HUMAN | Polyubiquitin-B | 0.61 | 0.03 | 0.56 | 0.44 |
