# Supplementary material for: Proteomics analysis of bladder cancer invasion: Targeting EIF3D for therapeutic intervention
Source: Oncotarget. 2017 Apr 20;8(41):69435–55. doi: 10.18632/oncotarget.17279 (PMC5642490; doi:10.18632/oncotarget.17279)
Supplement: Supplementary file 3 [file oncotarget-08-69435-s003.doc]

**Supplementary Table 3. Full list of proteins identified using (A) Proteome Discoverer and (B) Trans-Proteomic Pipeline.**

1. **Proteome Discoverer**

| **Accession** | **Protein Name** | **Fold change (pT2+/pTa)** | **p-value** | **Mean_pTa** | **SD_pTa** | **Mean_pT2+** | **SD_pT2+** | **pTa_3** | **pTa_6** | **pTa_11** | **pTa_16** | **pTa_19** | **pT2+_9** | **pT2+_12** | **pT2+_13** | **pT2+_14** | **pT2+_15** | **pT2+_17** |
| --- | --- | --- | --- | --- | --- | --- | --- | --- | --- | --- | --- | --- | --- | --- | --- | --- | --- | --- |
| Q8NFU3 | Thiosulfate sulfurtransferase/rhodanese-like domain-containing protein 1 | **0.04** | **0.00** | 46.91 | 12.89 | 2.10 | 5.14 | 61.74 | 34.82 | 32.02 | 54.27 | 51.70 | 0.00 | 12.58 | 0.00 | 0.00 | 0.00 | 0.00 |
| Q15678 | Tyrosine-protein phosphatase non-receptor type 14 | **17.80** | **0.00** | 16.17 | 36.16 | 287.88 | 134.59 | 0.00 | 0.00 | 0.00 | 80.86 | 0.00 | 84.18 | 311.22 | 376.13 | 468.79 | 288.58 | 198.37 |
| O95994 | Anterior gradient protein 2 homolog | **only in pTa** | **0.00** | 198.59 | 85.97 | 0.00 | 0.00 | 200.72 | 100.59 | 133.84 | 241.04 | 316.75 | 0.00 | 0.00 | 0.00 | 0.00 | 0.00 | 0.00 |
| P11413 | Glucose-6-phosphate 1-dehydrogenase | **only in pT2+** | **0.00** | 0.00 | 0.00 | 65.92 | 49.93 | 0.00 | 0.00 | 0.00 | 0.00 | 0.00 | 146.77 | 107.97 | 48.39 | 20.94 | 37.30 | 34.15 |
| P04066 | Tissue alpha-L-fucosidase | **0.16** | **0.00** | 28.98 | 12.90 | 4.75 | 8.08 | 32.70 | 26.37 | 14.62 | 48.87 | 22.36 | 0.00 | 9.00 | 0.00 | 0.00 | 19.53 | 0.00 |
| P08758 | Annexin A5 | **2.82** | **0.01** | 161.60 | 66.51 | 455.74 | 170.14 | 113.13 | 272.75 | 165.05 | 108.61 | 148.45 | 269.26 | 338.46 | 698.77 | 592.74 | 505.50 | 329.69 |
| O43493 | Trans-Golgi network integral membrane protein 2 | **3.72** | **0.01** | 3.48 | 4.92 | 12.94 | 4.14 | 0.00 | 0.00 | 0.00 | 7.02 | 10.40 | 13.97 | 19.69 | 8.88 | 9.66 | 10.31 | 15.13 |
| Q9NSB2 | Keratin, type II cuticular Hb4 | **0.52** | **0.01** | 2081.44 | 478.59 | 1081.85 | 463.03 | 2816.20 | 1876.01 | 2284.08 | 1827.59 | 1603.30 | 1270.29 | 1201.76 | 1237.08 | 272.41 | 1630.95 | 878.64 |
| P50552 | Vasodilator-stimulated phosphoprotein | **0.21** | **0.01** | 65.56 | 25.89 | 13.85 | 23.43 | 35.72 | 38.81 | 84.10 | 82.57 | 86.60 | 0.00 | 26.68 | 0.00 | 0.00 | 56.44 | 0.00 |
| Q15847 | Adipogenesis regulatory factor | **0.12** | **0.01** | 292.68 | 171.97 | 34.32 | 58.88 | 535.19 | 263.97 | 377.46 | 84.69 | 202.10 | 0.00 | 0.00 | 0.00 | 0.00 | 142.95 | 62.99 |
| Q15582 | Transforming growth factor-beta-induced protein ig-h3 | **6.84** | **0.01** | 30.39 | 32.65 | 207.91 | 112.35 | 66.94 | 0.00 | 62.34 | 22.67 | 0.00 | 212.82 | 182.48 | 261.28 | 318.26 | 272.63 | 0.00 |
| P12035 | Keratin, type II cytoskeletal 3 | **0.51** | **0.01** | 2091.40 | 466.52 | 1062.86 | 534.06 | 2816.20 | 1876.01 | 2284.08 | 1827.59 | 1653.11 | 1470.39 | 1201.76 | 1237.08 | 249.29 | 1630.95 | 587.69 |
| O00264 | Membrane-associated progesterone receptor component 1 | **0.36** | **0.01** | 94.36 | 31.90 | 33.55 | 27.22 | 142.11 | 110.98 | 66.10 | 70.65 | 81.96 | 65.90 | 44.47 | 0.00 | 0.00 | 46.65 | 44.29 |
| Q9UI15 | Transgelin-3 | **0.20** | **0.01** | 209.08 | 95.78 | 42.17 | 68.13 | 264.02 | 230.84 | 321.69 | 78.33 | 150.52 | 157.03 | 0.00 | 0.00 | 0.00 | 96.02 | 0.00 |
| P28161 | Glutathione S-transferase Mu 2 | **0.22** | **0.01** | 1134.96 | 577.45 | 247.44 | 279.69 | 1457.18 | 211.32 | 956.36 | 1382.20 | 1667.74 | 0.00 | 778.15 | 126.96 | 69.37 | 286.06 | 224.10 |
| P17516 | Aldo-keto reductase family 1 member C4 | **0.17** | **0.01** | 110.82 | 61.48 | 19.28 | 26.07 | 206.22 | 84.35 | 40.78 | 97.05 | 125.70 | 30.86 | 65.64 | 0.00 | 0.00 | 0.00 | 19.18 |
| Q15631 | Translin | **7.55** | **0.01** | 10.19 | 13.95 | 76.93 | 44.36 | 25.78 | 0.00 | 0.00 | 25.16 | 0.00 | 101.33 | 122.20 | 51.68 | 0.00 | 85.73 | 100.63 |
| Q5VW32 | BRO1 domain-containing protein BROX | **only in pTa** | **0.01** | 32.16 | 18.35 | 0.00 | 0.00 | 41.53 | 0.00 | 34.17 | 44.19 | 40.91 | 0.00 | 0.00 | 0.00 | 0.00 | 0.00 | 0.00 |
| Q8TD06 | Anterior gradient protein 3 homolog | **only in pTa** | **0.01** | 127.11 | 111.65 | 0.00 | 0.00 | 211.00 | 0.00 | 129.28 | 261.25 | 34.00 | 0.00 | 0.00 | 0.00 | 0.00 | 0.00 | 0.00 |
| P40616 | ADP-ribosylation factor-like protein 1 | **4.76** | **0.01** | 5.29 | 11.82 | 25.19 | 9.21 | 0.00 | 0.00 | 0.00 | 26.43 | 0.00 | 26.25 | 31.35 | 37.67 | 20.53 | 24.52 | 10.80 |
| P23284 | Peptidyl-prolyl cis-trans isomerase B | **1.44** | **0.01** | 366.70 | 91.16 | 527.13 | 77.51 | 328.89 | 306.99 | 452.20 | 474.84 | 270.58 | 617.49 | 476.43 | 575.98 | 548.08 | 399.98 | 544.83 |
| A6NI79 | Coiled-coil domain-containing protein 69 | **only in pT2+** | **0.01** | 0.00 | 0.00 | 531.12 | 541.87 | 0.00 | 0.00 | 0.00 | 0.00 | 0.00 | 566.73 | 314.03 | 1561.29 | 486.24 | 258.40 | 0.00 |
| O15078 | Centrosomal protein of 290 kDa | **only in pT2+** | **0.01** | 0.00 | 0.00 | 531.12 | 541.87 | 0.00 | 0.00 | 0.00 | 0.00 | 0.00 | 566.73 | 314.03 | 1561.29 | 486.24 | 258.40 | 0.00 |
| Q5JR59 | Microtubule-associated tumor suppressor candidate 2 | **only in pT2+** | **0.01** | 0.00 | 0.00 | 531.12 | 541.87 | 0.00 | 0.00 | 0.00 | 0.00 | 0.00 | 566.73 | 314.03 | 1561.29 | 486.24 | 258.40 | 0.00 |
| Q9H2F5 | Enhancer of polycomb homolog 1 | **only in pT2+** | **0.01** | 0.00 | 0.00 | 488.22 | 502.81 | 0.00 | 0.00 | 0.00 | 0.00 | 0.00 | 526.96 | 467.91 | 1436.20 | 322.75 | 175.52 | 0.00 |
| P53350 | Serine/threonine-protein kinase PLK1 | **only in pT2+** | **0.01** | 0.00 | 0.00 | 310.11 | 286.28 | 0.00 | 0.00 | 0.00 | 0.00 | 0.00 | 179.18 | 220.66 | 712.48 | 617.42 | 130.92 | 0.00 |
| P24821 | Tenascin | **only in pT2+** | **0.01** | 0.00 | 0.00 | 242.61 | 244.37 | 0.00 | 0.00 | 0.00 | 0.00 | 0.00 | 65.09 | 205.49 | 560.86 | 531.08 | 93.15 | 0.00 |
| P13727 | Bone marrow proteoglycan | **only in pT2+** | **0.01** | 0.00 | 0.00 | 120.20 | 148.72 | 0.00 | 0.00 | 0.00 | 0.00 | 0.00 | 26.00 | 33.13 | 349.14 | 267.47 | 45.49 | 0.00 |
| P17612 | cAMP-dependent protein kinase catalytic subunit alpha | **only in pT2+** | **0.01** | 0.00 | 0.00 | 44.89 | 28.02 | 0.00 | 0.00 | 0.00 | 0.00 | 0.00 | 0.00 | 80.95 | 66.19 | 46.91 | 32.83 | 42.45 |
| P22694 | cAMP-dependent protein kinase catalytic subunit beta | **only in pT2+** | **0.01** | 0.00 | 0.00 | 44.89 | 28.02 | 0.00 | 0.00 | 0.00 | 0.00 | 0.00 | 0.00 | 80.95 | 66.19 | 46.91 | 32.83 | 42.45 |
| P57088 | Transmembrane protein 33 | **only in pT2+** | **0.01** | 0.00 | 0.00 | 33.67 | 30.62 | 0.00 | 0.00 | 0.00 | 0.00 | 0.00 | 9.84 | 86.00 | 43.53 | 22.92 | 0.00 | 39.77 |
| P04083 | Annexin A1 | **3.25** | **0.01** | 145.54 | 58.49 | 472.73 | 233.31 | 178.36 | 153.31 | 94.09 | 221.12 | 80.83 | 195.05 | 507.14 | 473.18 | 524.41 | 274.22 | 862.35 |
| P60468 | Protein transport protein Sec61 subunit beta | **7.26** | **0.02** | 11.29 | 25.25 | 81.96 | 46.95 | 0.00 | 0.00 | 0.00 | 0.00 | 56.47 | 94.49 | 128.97 | 115.38 | 95.96 | 0.00 | 57.00 |
| P07948 | Tyrosine-protein kinase Lyn | **7.00** | **0.02** | 6.40 | 14.31 | 44.77 | 25.32 | 0.00 | 0.00 | 31.99 | 0.00 | 0.00 | 53.83 | 55.22 | 30.15 | 0.00 | 65.94 | 63.46 |
| P08631 | Tyrosine-protein kinase HCK | **7.00** | **0.02** | 6.40 | 14.31 | 44.77 | 25.32 | 0.00 | 0.00 | 31.99 | 0.00 | 0.00 | 53.83 | 55.22 | 30.15 | 0.00 | 65.94 | 63.46 |
| P49720 | Proteasome subunit beta type-3 | **3.28** | **0.02** | 14.55 | 21.25 | 47.68 | 15.21 | 0.00 | 0.00 | 25.90 | 46.83 | 0.00 | 36.24 | 56.67 | 36.94 | 44.60 | 37.27 | 74.36 |
| Q8N1A0 | Keratin-like protein KRT222 | **0.39** | **0.02** | 1843.33 | 718.92 | 715.19 | 526.14 | 3109.40 | 1335.36 | 1568.64 | 1517.65 | 1685.61 | 1615.62 | 356.46 | 886.45 | 133.73 | 834.65 | 464.26 |
| P46439 | Glutathione S-transferase Mu 5 | **0.23** | **0.02** | 1624.49 | 877.87 | 372.67 | 493.29 | 2530.84 | 183.90 | 1605.91 | 2028.81 | 1772.99 | 0.00 | 1334.11 | 198.10 | 58.72 | 420.98 | 224.10 |
| Q9ULL5 | Proline-rich protein 12 | **4.99** | **0.02** | 142.11 | 220.71 | 708.88 | 375.74 | 0.00 | 0.00 | 502.58 | 0.00 | 207.96 | 432.37 | 801.21 | 1273.26 | 938.23 | 582.96 | 225.23 |
| Q14CN4 | Keratin, type II cytoskeletal 72 | **0.38** | **0.02** | 1879.34 | 864.07 | 704.89 | 413.22 | 3163.54 | 959.60 | 2284.08 | 1620.71 | 1368.77 | 1400.13 | 492.59 | 915.78 | 215.79 | 704.18 | 500.87 |
| Q9NY65 | Tubulin alpha-8 chain | **1.70** | **0.02** | 1091.04 | 542.98 | 1859.08 | 320.57 | 1205.85 | 556.13 | 1306.39 | 1830.86 | 555.97 | 1532.56 | 2041.00 | 1595.22 | 1639.08 | 2344.51 | 2002.08 |
| Q9UGJ0 | 5'-AMP-activated protein kinase subunit gamma-2 | **0.42** | **0.02** | 5333.33 | 2175.14 | 2266.38 | 1254.29 | 5486.85 | 6453.00 | 4776.25 | 2052.86 | 7897.69 | 3989.39 | 1433.93 | 634.15 | 3421.15 | 2306.30 | 1813.37 |
| P53621 | Coatomer subunit alpha | **2.76** | **0.02** | 24.74 | 27.77 | 68.34 | 22.57 | 23.59 | 0.00 | 33.09 | 67.04 | 0.00 | 43.87 | 102.02 | 84.59 | 74.42 | 49.82 | 55.31 |
| P68366 | Tubulin alpha-4A chain | **1.76** | **0.02** | 1070.71 | 588.36 | 1881.05 | 328.85 | 1205.85 | 391.45 | 1306.39 | 1858.13 | 591.72 | 1532.56 | 2041.00 | 1595.22 | 1669.57 | 2350.57 | 2097.38 |
| Q13748 | Tubulin alpha-3C/D chain | **1.69** | **0.02** | 1140.24 | 571.59 | 1929.05 | 321.11 | 1205.85 | 518.74 | 1350.91 | 1950.16 | 675.56 | 1606.82 | 2041.00 | 1706.84 | 1669.57 | 2421.76 | 2128.33 |
| Q6PEY2 | Tubulin alpha-3E chain | **1.69** | **0.02** | 1140.24 | 571.59 | 1929.05 | 321.11 | 1205.85 | 518.74 | 1350.91 | 1950.16 | 675.56 | 1606.82 | 2041.00 | 1706.84 | 1669.57 | 2421.76 | 2128.33 |
| Q5XKE5 | Keratin, type II cytoskeletal 79 | **0.40** | **0.02** | 2493.76 | 960.92 | 1005.63 | 762.22 | 3868.45 | 1305.42 | 2001.02 | 2872.61 | 2421.30 | 2388.79 | 846.13 | 798.42 | 219.78 | 1263.98 | 516.67 |
| P11215 | Integrin alpha-M | **0.24** | **0.02** | 1130.07 | 673.92 | 275.09 | 254.66 | 1800.74 | 774.43 | 215.56 | 1764.76 | 1094.84 | 593.33 | 0.00 | 298.33 | 0.00 | 536.47 | 222.41 |
| P09488 | Glutathione S-transferase Mu 1 | **0.23** | **0.02** | 1790.04 | 903.66 | 416.55 | 668.21 | 2700.72 | 309.93 | 2151.95 | 2110.41 | 1677.16 | 0.00 | 1723.79 | 179.61 | 48.08 | 516.30 | 31.51 |
| Q9Y696 | Chloride intracellular channel protein 4 | **34.96** | **0.02** | 1.22 | 2.72 | 42.50 | 29.87 | 6.08 | 0.00 | 0.00 | 0.00 | 0.00 | 18.84 | 0.00 | 36.09 | 53.58 | 77.76 | 68.71 |
| A6NMY6 | Putative annexin A2-like protein | **1.61** | **0.02** | 718.96 | 222.76 | 1157.67 | 274.98 | 911.27 | 626.45 | 515.91 | 1001.22 | 539.95 | 1281.13 | 892.99 | 1345.30 | 1435.19 | 742.64 | 1248.79 |
| P26447 | Protein S100-A4 | **0.31** | **0.02** | 973.42 | 466.86 | 299.13 | 317.49 | 1699.38 | 550.57 | 770.58 | 678.49 | 1168.08 | 171.23 | 145.71 | 384.21 | 197.04 | 896.58 | 0.00 |
| Q12907 | Vesicular integral-membrane protein VIP36 | **2.51** | **0.02** | 22.67 | 22.88 | 56.90 | 17.49 | 48.37 | 0.00 | 22.19 | 42.81 | 0.00 | 58.41 | 81.30 | 74.10 | 39.46 | 43.33 | 44.77 |
| O00515 | Ladinin-1 | **0.18** | **0.02** | 37.85 | 23.02 | 6.74 | 13.07 | 33.42 | 0.00 | 51.41 | 46.25 | 58.19 | 32.65 | 0.00 | 0.00 | 0.00 | 0.00 | 7.80 |
| Q13492 | Phosphatidylinositol-binding clathrin assembly protein | **0.38** | **0.02** | 777.24 | 334.27 | 298.76 | 226.77 | 908.66 | 774.43 | 215.56 | 892.68 | 1094.84 | 593.33 | 95.14 | 304.38 | 40.86 | 536.47 | 222.41 |
| P19971 | Thymidine phosphorylase | **13.24** | **0.02** | 8.58 | 19.18 | 113.57 | 81.49 | 0.00 | 42.88 | 0.00 | 0.00 | 0.00 | 0.00 | 89.83 | 151.92 | 245.25 | 83.17 | 111.25 |
| Q9NVE4 | Coiled-coil domain-containing protein 87 | **13.12** | **0.02** | 15.17 | 33.93 | 199.11 | 137.06 | 0.00 | 0.00 | 0.00 | 75.87 | 0.00 | 100.04 | 284.82 | 336.98 | 148.86 | 0.00 | 323.95 |
| O95861 | 3'(2'),5'-bisphosphate nucleotidase 1 | **0.12** | **0.02** | 40.99 | 28.94 | 5.10 | 12.49 | 57.51 | 0.00 | 28.92 | 42.17 | 76.35 | 0.00 | 0.00 | 0.00 | 0.00 | 0.00 | 30.58 |
| P35609 | Alpha-actinin-2 | **2.00** | **0.02** | 199.92 | 70.30 | 399.40 | 147.22 | 227.46 | 115.65 | 275.29 | 245.63 | 135.57 | 209.44 | 354.57 | 647.37 | 350.41 | 470.89 | 363.72 |
| P07355 | Annexin A2 | **1.71** | **0.02** | 723.34 | 217.97 | 1238.31 | 362.44 | 911.27 | 626.45 | 532.65 | 1001.22 | 545.13 | 1312.21 | 892.99 | 1610.76 | 1622.50 | 742.64 | 1248.79 |
| Q03013 | Glutathione S-transferase Mu 4 | **0.24** | **0.02** | 1646.80 | 860.54 | 396.90 | 634.71 | 2631.41 | 337.36 | 1959.17 | 1953.31 | 1352.74 | 0.00 | 1629.56 | 187.48 | 48.08 | 516.30 | 0.00 |
| P61009 | Signal peptidase complex subunit 3 | **4.18** | **0.02** | 13.32 | 29.78 | 55.71 | 21.42 | 0.00 | 0.00 | 0.00 | 66.59 | 0.00 | 75.30 | 74.14 | 24.29 | 72.96 | 43.27 | 44.30 |
| O75396 | Vesicle-trafficking protein SEC22b | **2.77** | **0.02** | 36.02 | 43.73 | 99.76 | 34.05 | 36.43 | 0.00 | 0.00 | 107.10 | 36.58 | 71.97 | 91.05 | 81.60 | 70.66 | 130.12 | 153.14 |
| P42224 | Signal transducer and activator of transcription 1-alpha/beta | **7.37** | **0.02** | 9.58 | 8.95 | 70.62 | 47.03 | 15.60 | 0.00 | 13.43 | 18.86 | 0.00 | 0.00 | 102.80 | 107.57 | 116.06 | 66.50 | 30.79 |
| Q32MZ4 | Leucine-rich repeat flightless-interacting protein 1 | **0.34** | **0.02** | 40.68 | 20.30 | 13.66 | 12.42 | 76.68 | 32.05 | 27.24 | 33.10 | 34.34 | 0.00 | 24.16 | 14.59 | 0.00 | 12.62 | 30.58 |
| P35408 | Prostaglandin E2 receptor EP4 subtype | **0.28** | **0.02** | 1130.07 | 673.92 | 318.26 | 280.08 | 1800.74 | 774.43 | 215.56 | 1764.76 | 1094.84 | 593.33 | 0.00 | 557.37 | 0.00 | 536.47 | 222.41 |
| O76070 | Gamma-synuclein | **0.13** | **0.02** | 221.28 | 125.04 | 29.63 | 38.90 | 349.36 | 72.89 | 211.72 | 127.64 | 344.78 | 96.73 | 50.39 | 0.00 | 0.00 | 30.64 | 0.00 |
| P63096 | Guanine nucleotide-binding protein G(i) subunit alpha-1 | **3.84** | **0.03** | 127.82 | 120.80 | 490.71 | 280.16 | 31.58 | 0.00 | 274.30 | 231.04 | 102.17 | 631.40 | 130.59 | 691.13 | 836.64 | 449.10 | 205.40 |
| P12814 | Alpha-actinin-1 | **2.13** | **0.03** | 295.48 | 128.44 | 629.16 | 249.84 | 326.25 | 139.72 | 477.15 | 322.66 | 211.62 | 309.57 | 490.54 | 993.81 | 840.59 | 625.62 | 514.85 |
| O43707 | Alpha-actinin-4 | **2.09** | **0.03** | 303.52 | 133.24 | 635.04 | 246.26 | 331.04 | 142.21 | 488.21 | 344.52 | 211.62 | 313.52 | 490.54 | 993.81 | 840.59 | 625.62 | 546.15 |
| P28838 | Cytosol aminopeptidase | **4.63** | **0.03** | 18.58 | 20.36 | 86.05 | 53.34 | 40.43 | 0.00 | 39.87 | 12.58 | 0.00 | 39.60 | 61.90 | 172.10 | 127.82 | 76.12 | 38.75 |
| P35998 | 26S protease regulatory subunit 7 | **1.82** | **0.03** | 15.55 | 10.39 | 28.33 | 5.11 | 24.33 | 0.00 | 12.34 | 25.69 | 15.38 | 34.50 | 31.83 | 30.92 | 20.65 | 27.49 | 24.60 |
| P13639 | Elongation factor 2 | **1.62** | **0.03** | 216.66 | 110.06 | 351.58 | 54.18 | 178.34 | 74.77 | 230.50 | 379.93 | 219.76 | 332.15 | 435.10 | 376.12 | 273.26 | 330.05 | 362.79 |
| P08727 | Keratin, type I cytoskeletal 19 | **0.35** | **0.03** | 3182.29 | 1760.08 | 1111.58 | 698.43 | 6012.32 | 2267.23 | 1624.14 | 3729.72 | 2278.06 | 2082.25 | 963.58 | 1295.53 | 137.92 | 1591.36 | 598.83 |
| Q92572 | AP-3 complex subunit sigma-1 | **0.31** | **0.03** | 1130.07 | 673.92 | 351.34 | 244.43 | 1800.74 | 774.43 | 215.56 | 1764.76 | 1094.84 | 593.33 | 198.45 | 557.37 | 0.00 | 536.47 | 222.41 |
| P61313 | 60S ribosomal protein L15 | **2.18** | **0.03** | 70.41 | 66.33 | 153.16 | 36.25 | 37.92 | 0.00 | 92.64 | 173.34 | 48.16 | 145.73 | 142.14 | 173.24 | 96.39 | 155.74 | 205.72 |
| O00232 | 26S proteasome non-ATPase regulatory subunit 12 | **3.82** | **0.03** | 5.72 | 12.80 | 21.85 | 7.66 | 0.00 | 0.00 | 0.00 | 28.62 | 0.00 | 14.69 | 28.14 | 15.14 | 21.51 | 17.86 | 33.73 |
| Q8IW92 | Beta-galactosidase-1-like protein 2 | **3.19** | **0.03** | 207.29 | 226.32 | 661.72 | 329.48 | 236.32 | 0.00 | 548.01 | 252.14 | 0.00 | 171.64 | 599.37 | 1130.34 | 909.12 | 627.49 | 532.36 |
| P19012 | Keratin, type I cytoskeletal 15 | **0.45** | **0.03** | 1958.21 | 800.29 | 883.18 | 569.53 | 3306.67 | 1333.27 | 1373.51 | 1861.72 | 1915.90 | 1734.83 | 782.46 | 1274.81 | 137.92 | 906.61 | 462.45 |
| P08754 | Guanine nucleotide-binding protein G(k) subunit alpha | **3.31** | **0.03** | 127.82 | 120.80 | 423.25 | 238.10 | 31.58 | 0.00 | 274.30 | 231.04 | 102.17 | 631.40 | 119.40 | 691.13 | 568.71 | 311.49 | 217.36 |
| P62269 | 40S ribosomal protein S18 | **1.64** | **0.03** | 164.16 | 57.08 | 268.92 | 74.10 | 124.39 | 117.46 | 175.49 | 257.94 | 145.54 | 271.14 | 381.66 | 212.24 | 186.04 | 234.56 | 327.89 |
| Q9BYT8 | Neurolysin, mitochondrial | **11.58** | **0.03** | 14.67 | 32.81 | 169.93 | 129.33 | 73.37 | 0.00 | 0.00 | 0.00 | 0.00 | 129.58 | 224.30 | 243.94 | 354.65 | 0.00 | 67.12 |
| Q08043 | Alpha-actinin-3 | **2.34** | **0.03** | 177.18 | 54.59 | 414.04 | 201.79 | 230.44 | 115.65 | 217.90 | 200.38 | 121.51 | 227.18 | 387.46 | 800.45 | 391.66 | 397.12 | 280.38 |
| P07942 | Laminin subunit beta-1 | **9.11** | **0.03** | 15.51 | 34.67 | 141.23 | 106.00 | 0.00 | 0.00 | 77.53 | 0.00 | 0.00 | 308.31 | 116.52 | 69.33 | 180.48 | 172.75 | 0.00 |
| P16989 | Y-box-binding protein 3 | **2.06** | **0.03** | 42.39 | 30.50 | 87.33 | 28.56 | 33.89 | 49.90 | 43.39 | 84.77 | 0.00 | 111.38 | 99.69 | 42.48 | 60.58 | 105.52 | 104.30 |
| Q12802 | A-kinase anchor protein 13 | **0.02** | **0.03** | 1029.25 | 702.94 | 23.50 | 57.57 | 1208.34 | 1504.31 | 674.12 | 0.00 | 1759.50 | 0.00 | 141.01 | 0.00 | 0.00 | 0.00 | 0.00 |
| P0CG47 | Polyubiquitin-B | **0.61** | **0.03** | 1617.29 | 488.36 | 994.58 | 329.88 | 1626.04 | 1992.49 | 2058.55 | 1577.46 | 831.90 | 1158.33 | 1254.22 | 1106.18 | 397.44 | 1224.29 | 827.04 |
| P0CG48 | Polyubiquitin-C | **0.61** | **0.03** | 1617.29 | 488.36 | 994.58 | 329.88 | 1626.04 | 1992.49 | 2058.55 | 1577.46 | 831.90 | 1158.33 | 1254.22 | 1106.18 | 397.44 | 1224.29 | 827.04 |
| P62979 | Ubiquitin-40S ribosomal protein S27a | **0.61** | **0.03** | 1617.29 | 488.36 | 994.58 | 329.88 | 1626.04 | 1992.49 | 2058.55 | 1577.46 | 831.90 | 1158.33 | 1254.22 | 1106.18 | 397.44 | 1224.29 | 827.04 |
| P62987 | Ubiquitin-60S ribosomal protein L40 | **0.61** | **0.03** | 1617.29 | 488.36 | 994.58 | 329.88 | 1626.04 | 1992.49 | 2058.55 | 1577.46 | 831.90 | 1158.33 | 1254.22 | 1106.18 | 397.44 | 1224.29 | 827.04 |
| P14174 | Macrophage migration inhibitory factor | **0.36** | **0.03** | 1192.52 | 637.93 | 423.40 | 362.07 | 2270.99 | 707.27 | 913.56 | 1261.27 | 809.49 | 0.00 | 561.44 | 779.24 | 388.93 | 810.80 | 0.00 |
| P04798 | Cytochrome P450 1A1 | **only in pT2+** | **0.03** | 0.00 | 0.00 | 410.39 | 457.85 | 0.00 | 0.00 | 0.00 | 0.00 | 0.00 | 0.00 | 340.18 | 716.57 | 1172.69 | 232.89 | 0.00 |
| Q86Z14 | Beta-klotho | **only in pT2+** | **0.03** | 0.00 | 0.00 | 409.48 | 570.99 | 0.00 | 0.00 | 0.00 | 0.00 | 0.00 | 248.99 | 0.00 | 1461.24 | 656.39 | 0.00 | 90.26 |
| O00743 | Serine/threonine-protein phosphatase 6 catalytic subunit | **only in pT2+** | **0.03** | 0.00 | 0.00 | 307.58 | 263.10 | 0.00 | 0.00 | 0.00 | 0.00 | 0.00 | 379.20 | 0.00 | 0.00 | 352.24 | 669.56 | 444.45 |
| P18084 | Integrin beta-5 | **only in pT2+** | **0.03** | 0.00 | 0.00 | 236.11 | 230.48 | 0.00 | 0.00 | 0.00 | 0.00 | 0.00 | 392.95 | 186.60 | 592.47 | 0.00 | 244.67 | 0.00 |
| Q8NHM5 | Lysine-specific demethylase 2B | **only in pT2+** | **0.03** | 0.00 | 0.00 | 202.07 | 250.28 | 0.00 | 0.00 | 0.00 | 0.00 | 0.00 | 26.53 | 386.45 | 610.19 | 189.27 | 0.00 | 0.00 |
| Q8IVF4 | Dynein heavy chain 10, axonemal | **only in pT2+** | **0.03** | 0.00 | 0.00 | 200.47 | 161.80 | 0.00 | 0.00 | 0.00 | 0.00 | 0.00 | 291.31 | 224.30 | 324.62 | 362.60 | 0.00 | 0.00 |
| Q9UK80 | Ubiquitin carboxyl-terminal hydrolase 21 | **only in pT2+** | **0.03** | 0.00 | 0.00 | 181.00 | 227.74 | 0.00 | 0.00 | 0.00 | 0.00 | 0.00 | 0.00 | 72.28 | 503.26 | 438.40 | 0.00 | 72.04 |
| Q5JV73 | FERM and PDZ domain-containing protein 3 | **only in pT2+** | **0.03** | 0.00 | 0.00 | 158.74 | 142.26 | 0.00 | 0.00 | 0.00 | 0.00 | 0.00 | 129.58 | 224.30 | 243.94 | 354.65 | 0.00 | 0.00 |
| Q5W0A0 | Protein FAM194B | **only in pT2+** | **0.03** | 0.00 | 0.00 | 158.74 | 142.26 | 0.00 | 0.00 | 0.00 | 0.00 | 0.00 | 129.58 | 224.30 | 243.94 | 354.65 | 0.00 | 0.00 |
| Q8IZF6 | Probable G-protein coupled receptor 112 | **only in pT2+** | **0.03** | 0.00 | 0.00 | 158.74 | 142.26 | 0.00 | 0.00 | 0.00 | 0.00 | 0.00 | 129.58 | 224.30 | 243.94 | 354.65 | 0.00 | 0.00 |
| Q8TDJ6 | DmX-like protein 2 | **only in pT2+** | **0.03** | 0.00 | 0.00 | 158.74 | 142.26 | 0.00 | 0.00 | 0.00 | 0.00 | 0.00 | 129.58 | 224.30 | 243.94 | 354.65 | 0.00 | 0.00 |
| Q9NZ52 | ADP-ribosylation factor-binding protein GGA3 | **only in pT2+** | **0.03** | 0.00 | 0.00 | 158.74 | 142.26 | 0.00 | 0.00 | 0.00 | 0.00 | 0.00 | 129.58 | 224.30 | 243.94 | 354.65 | 0.00 | 0.00 |
| Q9UJY4 | ADP-ribosylation factor-binding protein GGA2 | **only in pT2+** | **0.03** | 0.00 | 0.00 | 158.74 | 142.26 | 0.00 | 0.00 | 0.00 | 0.00 | 0.00 | 129.58 | 224.30 | 243.94 | 354.65 | 0.00 | 0.00 |
| Q9UJY5 | ADP-ribosylation factor-binding protein GGA1 | **only in pT2+** | **0.03** | 0.00 | 0.00 | 158.74 | 142.26 | 0.00 | 0.00 | 0.00 | 0.00 | 0.00 | 129.58 | 224.30 | 243.94 | 354.65 | 0.00 | 0.00 |
| Q14956 | Transmembrane glycoprotein NMB | **only in pT2+** | **0.03** | 0.00 | 0.00 | 110.44 | 151.60 | 0.00 | 0.00 | 0.00 | 0.00 | 0.00 | 30.64 | 201.15 | 0.00 | 0.00 | 51.29 | 379.56 |
| Q9BQ69 | O-acetyl-ADP-ribose deacetylase MACROD1 | **only in pT2+** | **0.03** | 0.00 | 0.00 | 90.72 | 99.05 | 0.00 | 0.00 | 0.00 | 0.00 | 0.00 | 110.10 | 83.30 | 0.00 | 0.00 | 81.11 | 269.79 |
| Q9P2D7 | Dynein heavy chain 1, axonemal | **only in pT2+** | **0.03** | 0.00 | 0.00 | 90.72 | 99.05 | 0.00 | 0.00 | 0.00 | 0.00 | 0.00 | 110.10 | 83.30 | 0.00 | 0.00 | 81.11 | 269.79 |
| P98095 | Fibulin-2 | **only in pT2+** | **0.03** | 0.00 | 0.00 | 81.73 | 98.45 | 0.00 | 0.00 | 0.00 | 0.00 | 0.00 | 34.02 | 0.00 | 126.22 | 257.15 | 72.97 | 0.00 |
| Q9Y3Z3 | Deoxynucleoside triphosphate triphosphohydrolase SAMHD1 | **only in pT2+** | **0.03** | 0.00 | 0.00 | 74.34 | 95.87 | 0.00 | 0.00 | 0.00 | 0.00 | 0.00 | 0.00 | 67.14 | 101.04 | 252.50 | 25.34 | 0.00 |
| Q5JQF8 | Polyadenylate-binding protein 1-like 2 | **only in pT2+** | **0.03** | 0.00 | 0.00 | 58.85 | 54.28 | 0.00 | 0.00 | 0.00 | 0.00 | 0.00 | 69.86 | 72.40 | 0.00 | 0.00 | 145.20 | 65.64 |
| Q9Y6N5 | Sulfide:quinone oxidoreductase, mitochondrial | **only in pT2+** | **0.03** | 0.00 | 0.00 | 54.35 | 56.67 | 0.00 | 0.00 | 0.00 | 0.00 | 0.00 | 0.00 | 71.31 | 59.20 | 42.75 | 0.00 | 152.87 |
| Q569H4 | Proline-rich protein 16 | **only in pT2+** | **0.03** | 0.00 | 0.00 | 41.84 | 33.01 | 0.00 | 0.00 | 0.00 | 0.00 | 0.00 | 69.83 | 52.30 | 0.00 | 0.00 | 60.40 | 68.51 |
| Q8WX93 | Palladin | **only in pT2+** | **0.03** | 0.00 | 0.00 | 40.75 | 34.76 | 0.00 | 0.00 | 0.00 | 0.00 | 0.00 | 0.00 | 34.80 | 64.14 | 79.28 | 66.30 | 0.00 |
| P22612 | cAMP-dependent protein kinase catalytic subunit gamma | **only in pT2+** | **0.03** | 0.00 | 0.00 | 40.06 | 33.95 | 0.00 | 0.00 | 0.00 | 0.00 | 0.00 | 0.00 | 82.49 | 66.19 | 46.91 | 0.00 | 44.77 |
| P16435 | NADPH--cytochrome P450 reductase | **only in pT2+** | **0.03** | 0.00 | 0.00 | 38.23 | 44.31 | 0.00 | 0.00 | 0.00 | 0.00 | 0.00 | 54.91 | 49.25 | 0.00 | 11.09 | 0.00 | 114.10 |
| P61421 | V-type proton ATPase subunit d 1 | **only in pT2+** | **0.03** | 0.00 | 0.00 | 27.15 | 22.89 | 0.00 | 0.00 | 0.00 | 0.00 | 0.00 | 39.80 | 24.52 | 0.00 | 0.00 | 49.06 | 49.49 |
| Q16527 | Cysteine and glycine-rich protein 2 | **only in pT2+** | **0.03** | 0.00 | 0.00 | 22.35 | 24.35 | 0.00 | 0.00 | 0.00 | 0.00 | 0.00 | 0.00 | 0.00 | 16.08 | 57.66 | 46.86 | 13.50 |
| P21281 | V-type proton ATPase subunit B, brain isoform | **only in pT2+** | **0.03** | 0.00 | 0.00 | 22.04 | 19.88 | 0.00 | 0.00 | 0.00 | 0.00 | 0.00 | 0.00 | 21.03 | 31.14 | 28.36 | 0.00 | 51.71 |
| P07099 | Epoxide hydrolase 1 | **only in pT2+** | **0.03** | 0.00 | 0.00 | 20.03 | 17.23 | 0.00 | 0.00 | 0.00 | 0.00 | 0.00 | 0.00 | 15.77 | 0.00 | 36.99 | 34.57 | 32.85 |
| O95168 | NADH dehydrogenase [ubiquinone] 1 beta subcomplex subunit 4 | **only in pT2+** | **0.03** | 0.00 | 0.00 | 13.63 | 11.63 | 0.00 | 0.00 | 0.00 | 0.00 | 0.00 | 24.21 | 0.00 | 23.73 | 11.02 | 22.79 | 0.00 |
| Q9NUV9 | GTPase IMAP family member 4 | **only in pT2+** | **0.03** | 0.00 | 0.00 | 9.89 | 8.72 | 0.00 | 0.00 | 0.00 | 0.00 | 0.00 | 0.00 | 13.40 | 16.74 | 20.95 | 8.24 | 0.00 |
| O00442 | RNA 3'-terminal phosphate cyclase | **only in pT2+** | **0.03** | 0.00 | 0.00 | 8.25 | 7.10 | 0.00 | 0.00 | 0.00 | 0.00 | 0.00 | 0.00 | 15.06 | 11.69 | 7.02 | 0.00 | 15.72 |
| P49736 | DNA replication licensing factor MCM2 | **only in pT2+** | **0.03** | 0.00 | 0.00 | 8.10 | 8.58 | 0.00 | 0.00 | 0.00 | 0.00 | 0.00 | 5.23 | 14.04 | 0.00 | 0.00 | 7.34 | 21.98 |
| P06396 | Gelsolin | **2.17** | **0.03** | 149.75 | 59.63 | 325.57 | 146.61 | 187.10 | 222.26 | 136.08 | 138.80 | 64.52 | 142.23 | 319.64 | 541.92 | 425.24 | 329.47 | 194.91 |
| P0CG38 | POTE ankyrin domain family member I | **1.38** | **0.03** | 3223.63 | 901.27 | 4463.95 | 751.36 | 3667.50 | 1897.21 | 3705.00 | 4124.39 | 2724.03 | 5447.71 | 3809.91 | 4929.32 | 4457.18 | 4740.52 | 3399.04 |
| P19013 | Keratin, type II cytoskeletal 4 | **0.48** | **0.03** | 1597.20 | 676.38 | 767.07 | 424.25 | 2692.25 | 886.14 | 1271.09 | 1660.96 | 1475.54 | 1371.90 | 501.96 | 645.05 | 198.78 | 762.69 | 1122.03 |
| P26640 | Valine--tRNA ligase | **2.82** | **0.04** | 14.78 | 22.33 | 41.65 | 13.34 | 23.58 | 0.00 | 0.00 | 50.30 | 0.00 | 43.21 | 36.07 | 40.99 | 20.77 | 61.27 | 47.60 |
| P08729 | Keratin, type II cytoskeletal 7 | **0.37** | **0.04** | 3440.76 | 1955.33 | 1289.81 | 800.57 | 6092.12 | 1876.73 | 1330.77 | 4601.10 | 3303.08 | 2249.07 | 810.55 | 1881.08 | 193.72 | 1803.92 | 800.54 |
| Q6ZMY3 | SPOC domain-containing protein 1 | **0.30** | **0.04** | 30563.65 | 20196.69 | 9172.96 | 6389.93 | 22665.80 | 32094.65 | 23685.75 | 10428.87 | 63943.15 | 5810.52 | 17028.46 | 0.00 | 6284.67 | 15194.99 | 10719.10 |
| P52209 | 6-phosphogluconate dehydrogenase, decarboxylating | **3.00** | **0.04** | 75.46 | 51.11 | 226.52 | 128.26 | 54.27 | 0.00 | 135.71 | 96.17 | 91.16 | 116.57 | 458.41 | 200.61 | 116.10 | 275.11 | 192.31 |
| P04899 | Guanine nucleotide-binding protein G(i) subunit alpha-2 | **2.82** | **0.04** | 115.20 | 111.83 | 325.13 | 160.60 | 34.94 | 0.00 | 222.33 | 245.78 | 72.94 | 339.92 | 128.34 | 315.57 | 604.36 | 344.97 | 217.60 |
| P61077 | Ubiquitin-conjugating enzyme E2 D3 | **4.88** | **0.04** | 14.74 | 32.97 | 71.99 | 42.82 | 0.00 | 0.00 | 0.00 | 0.00 | 73.72 | 96.94 | 95.39 | 117.86 | 45.67 | 0.00 | 76.08 |
| P62837 | Ubiquitin-conjugating enzyme E2 D2 | **4.88** | **0.04** | 14.74 | 32.97 | 71.99 | 42.82 | 0.00 | 0.00 | 0.00 | 0.00 | 73.72 | 96.94 | 95.39 | 117.86 | 45.67 | 0.00 | 76.08 |
| O00757 | Fructose-1,6-bisphosphatase isozyme 2 | **only in pTa** | **0.04** | 147.96 | 152.88 | 0.00 | 0.00 | 267.86 | 0.00 | 335.56 | 136.39 | 0.00 | 0.00 | 0.00 | 0.00 | 0.00 | 0.00 | 0.00 |
| O14737 | Programmed cell death protein 5 | **only in pTa** | **0.04** | 17.44 | 22.41 | 0.00 | 0.00 | 54.76 | 0.00 | 17.85 | 14.61 | 0.00 | 0.00 | 0.00 | 0.00 | 0.00 | 0.00 | 0.00 |
| P14091 | Cathepsin E | **only in pTa** | **0.04** | 73.78 | 75.29 | 0.00 | 0.00 | 176.34 | 85.01 | 0.00 | 107.54 | 0.00 | 0.00 | 0.00 | 0.00 | 0.00 | 0.00 | 0.00 |
| P19404 | NADH dehydrogenase [ubiquinone] flavoprotein 2, mitochondrial | **only in pTa** | **0.04** | 19.48 | 18.57 | 0.00 | 0.00 | 41.00 | 0.00 | 26.62 | 29.80 | 0.00 | 0.00 | 0.00 | 0.00 | 0.00 | 0.00 | 0.00 |
| P55290 | Cadherin-13 | **only in pTa** | **0.04** | 19.91 | 18.40 | 0.00 | 0.00 | 37.12 | 28.88 | 33.53 | 0.00 | 0.00 | 0.00 | 0.00 | 0.00 | 0.00 | 0.00 | 0.00 |
| Q13423 | NAD(P) transhydrogenase, mitochondrial | **only in pTa** | **0.04** | 17.87 | 17.19 | 0.00 | 0.00 | 30.06 | 0.00 | 21.97 | 37.32 | 0.00 | 0.00 | 0.00 | 0.00 | 0.00 | 0.00 | 0.00 |
| Q14376 | UDP-glucose 4-epimerase | **only in pTa** | **0.04** | 29.45 | 26.92 | 0.00 | 0.00 | 47.58 | 0.00 | 51.23 | 48.45 | 0.00 | 0.00 | 0.00 | 0.00 | 0.00 | 0.00 | 0.00 |
| Q14CN2 | Calcium-activated chloride channel regulator 4 | **only in pTa** | **0.04** | 35.01 | 42.70 | 0.00 | 0.00 | 39.70 | 0.00 | 104.32 | 0.00 | 31.04 | 0.00 | 0.00 | 0.00 | 0.00 | 0.00 | 0.00 |
| Q15843 | NEDD8 | **only in pTa** | **0.04** | 25.94 | 27.04 | 0.00 | 0.00 | 63.99 | 0.00 | 37.11 | 28.59 | 0.00 | 0.00 | 0.00 | 0.00 | 0.00 | 0.00 | 0.00 |
| Q4VC31 | Coiled-coil domain-containing protein 58 | **only in pTa** | **0.04** | 17.20 | 21.21 | 0.00 | 0.00 | 51.93 | 0.00 | 17.71 | 16.38 | 0.00 | 0.00 | 0.00 | 0.00 | 0.00 | 0.00 | 0.00 |
| Q9H773 | dCTP pyrophosphatase 1 | **only in pTa** | **0.04** | 9.70 | 9.73 | 0.00 | 0.00 | 18.53 | 0.00 | 20.30 | 9.67 | 0.00 | 0.00 | 0.00 | 0.00 | 0.00 | 0.00 | 0.00 |
| Q9UJ72 | Annexin A10 | **only in pTa** | **0.04** | 76.47 | 90.85 | 0.00 | 0.00 | 100.71 | 0.00 | 61.94 | 219.72 | 0.00 | 0.00 | 0.00 | 0.00 | 0.00 | 0.00 | 0.00 |
| Q9Y5K6 | CD2-associated protein | **only in pTa** | **0.04** | 32.08 | 32.36 | 0.00 | 0.00 | 50.09 | 0.00 | 74.42 | 35.88 | 0.00 | 0.00 | 0.00 | 0.00 | 0.00 | 0.00 | 0.00 |
| P15428 | 15-hydroxyprostaglandin dehydrogenase [NAD(+)] | **0.01** | **0.04** | 210.49 | 151.24 | 2.61 | 6.39 | 318.62 | 77.75 | 421.97 | 116.72 | 117.37 | 0.00 | 15.65 | 0.00 | 0.00 | 0.00 | 0.00 |
| O14776 | Transcription elongation regulator 1 | **52.23** | **0.04** | 3.10 | 6.93 | 161.78 | 138.34 | 0.00 | 0.00 | 0.00 | 15.49 | 0.00 | 129.58 | 224.30 | 243.94 | 354.65 | 0.00 | 18.18 |
| P62906 | 60S ribosomal protein L10a | **2.04** | **0.04** | 81.44 | 59.43 | 166.33 | 56.09 | 71.33 | 0.00 | 140.24 | 139.06 | 56.57 | 133.01 | 246.49 | 159.51 | 90.98 | 153.62 | 214.35 |
| Q3SY84 | Keratin, type II cytoskeletal 71 | **0.35** | **0.04** | 2647.86 | 1665.60 | 917.11 | 530.69 | 5598.52 | 1876.01 | 2284.08 | 1827.59 | 1653.11 | 1224.42 | 648.64 | 1237.08 | 215.79 | 1630.95 | 545.79 |
| P08133 | Annexin A6 | **5.73** | **0.04** | 29.12 | 32.09 | 166.78 | 122.36 | 27.78 | 0.00 | 83.68 | 16.26 | 17.89 | 48.36 | 140.87 | 334.97 | 286.58 | 154.98 | 34.93 |
| P13646 | Keratin, type I cytoskeletal 13 | **0.45** | **0.04** | 1867.12 | 831.63 | 837.51 | 580.11 | 3306.33 | 1328.37 | 1373.51 | 1861.72 | 1465.67 | 1827.17 | 660.72 | 1030.15 | 137.92 | 906.61 | 462.51 |
| P69905 | Hemoglobin subunit alpha | **0.40** | **0.04** | 20652.25 | 12308.93 | 8286.13 | 2830.91 | 18886.49 | 19912.35 | 16304.12 | 7346.22 | 40812.04 | 9664.38 | 8200.86 | 3603.73 | 9631.80 | 11791.88 | 6824.15 |
| Q7RTS7 | Keratin, type II cytoskeletal 74 | **0.35** | **0.04** | 2647.86 | 1665.60 | 926.65 | 537.79 | 5598.52 | 1876.01 | 2284.08 | 1827.59 | 1653.11 | 1281.64 | 648.64 | 1237.08 | 215.79 | 1630.95 | 545.79 |
| Q9H582 | Zinc finger protein 644 | **4.60** | **0.04** | 27.00 | 60.38 | 124.11 | 71.68 | 0.00 | 0.00 | 0.00 | 135.01 | 0.00 | 144.30 | 145.97 | 84.49 | 0.00 | 170.18 | 199.69 |
| P23381 | Tryptophan--tRNA ligase, cytoplasmic | **5.51** | **0.04** | 10.11 | 14.68 | 55.68 | 40.59 | 0.00 | 0.00 | 32.18 | 18.36 | 0.00 | 0.00 | 69.11 | 103.31 | 43.47 | 94.58 | 23.63 |
| Q7KZF4 | Staphylococcal nuclease domain-containing protein 1 | **4.08** | **0.04** | 10.29 | 15.07 | 41.96 | 26.20 | 0.00 | 0.00 | 18.15 | 33.28 | 0.00 | 42.96 | 43.75 | 0.00 | 27.29 | 68.41 | 69.37 |
| Q70Z35 | Phosphatidylinositol 3,4,5-trisphosphate-dependent Rac exchanger 2 protein | **2.02** | **0.04** | 225.25 | 157.10 | 455.57 | 160.75 | 329.22 | 0.00 | 203.54 | 411.81 | 181.68 | 500.38 | 396.14 | 337.36 | 403.41 | 336.08 | 760.02 |
| O15371 | Eukaryotic translation initiation factor 3 subunit D | **2.87** | **0.04** | 10.86 | 15.45 | 31.16 | 13.01 | 0.00 | 0.00 | 21.22 | 33.08 | 0.00 | 22.75 | 35.50 | 19.58 | 22.12 | 32.61 | 54.37 |
| Q86Y46 | Keratin, type II cytoskeletal 73 | **0.36** | **0.04** | 2647.86 | 1665.60 | 964.38 | 500.04 | 5598.52 | 1876.01 | 2284.08 | 1827.59 | 1653.11 | 1264.02 | 609.24 | 1237.08 | 272.41 | 1630.95 | 772.61 |
| Q9Y2T7 | Y-box-binding protein 2 | **1.92** | **0.04** | 45.52 | 30.22 | 87.33 | 28.56 | 49.54 | 49.90 | 43.39 | 84.77 | 0.00 | 111.38 | 99.69 | 42.48 | 60.58 | 105.52 | 104.30 |
| Q9Y6E2 | Basic leucine zipper and W2 domain-containing protein 2 | **7.15** | **0.04** | 3.78 | 8.45 | 27.01 | 21.15 | 0.00 | 0.00 | 18.90 | 0.00 | 0.00 | 38.41 | 41.41 | 0.00 | 0.00 | 45.74 | 36.48 |
| P0CG39 | POTE ankyrin domain family member J | **1.36** | **0.04** | 3176.02 | 869.83 | 4321.99 | 753.98 | 3510.70 | 1897.21 | 3650.30 | 4097.86 | 2724.03 | 5447.71 | 3623.41 | 4712.73 | 4178.10 | 4570.94 | 3399.04 |
| P46781 | 40S ribosomal protein S9 | **1.90** | **0.05** | 98.15 | 56.46 | 186.72 | 67.76 | 78.59 | 43.36 | 95.15 | 193.24 | 80.41 | 190.53 | 269.17 | 97.56 | 122.16 | 190.89 | 250.04 |
| O60506 | Heterogeneous nuclear ribonucleoprotein Q | **1.73** | **0.05** | 95.16 | 61.33 | 164.64 | 36.81 | 60.16 | 49.68 | 147.28 | 175.04 | 43.63 | 177.00 | 171.05 | 91.05 | 174.58 | 192.62 | 181.52 |
| P35268 | 60S ribosomal protein L22 | **1.51** | **0.05** | 160.79 | 64.72 | 242.42 | 24.44 | 130.23 | 104.58 | 202.56 | 253.65 | 112.91 | 273.62 | 269.31 | 212.48 | 224.54 | 240.68 | 233.92 |
| Q7Z794 | Keratin, type II cytoskeletal 1b | **0.49** | **0.05** | 1525.37 | 745.99 | 744.74 | 330.96 | 2856.53 | 1166.78 | 1218.43 | 1262.25 | 1122.88 | 1242.13 | 747.32 | 775.86 | 270.95 | 907.34 | 524.82 |
| P21266 | Glutathione S-transferase Mu 3 | **0.17** | **0.05** | 749.86 | 489.04 | 125.13 | 102.50 | 413.83 | 55.75 | 1024.33 | 1150.85 | 1104.56 | 0.00 | 292.36 | 99.23 | 69.37 | 98.29 | 191.51 |
| P07858 | Cathepsin B | **2.06** | **0.05** | 95.48 | 69.58 | 196.27 | 74.09 | 123.80 | 57.39 | 113.02 | 183.18 | 0.00 | 200.37 | 334.07 | 190.95 | 135.83 | 127.72 | 188.67 |
| P51149 | Ras-related protein Rab-7a | **1.94** | **0.05** | 70.61 | 50.15 | 136.90 | 44.86 | 76.52 | 0.00 | 74.29 | 140.89 | 61.36 | 122.24 | 166.80 | 120.46 | 72.99 | 134.26 | 204.66 |
| Q9NR31 | GTP-binding protein SAR1a | **1.73** | **0.05** | 71.93 | 38.04 | 124.26 | 37.34 | 90.88 | 49.24 | 63.05 | 127.01 | 29.47 | 104.43 | 146.34 | 169.96 | 89.37 | 80.98 | 154.47 |
| P05787 | Keratin, type II cytoskeletal 8 | **0.45** | **0.05** | 2941.74 | 1067.84 | 1331.07 | 1223.81 | 4438.76 | 1525.15 | 3367.17 | 2735.59 | 2642.03 | 3630.39 | 1246.95 | 1114.43 | 179.73 | 1388.69 | 426.20 |
| Q13938 | Calcyphosin | **0.21** | **0.05** | 252.72 | 190.53 | 53.49 | 88.79 | 573.73 | 183.62 | 106.99 | 127.11 | 272.15 | 228.76 | 48.12 | 44.09 | 0.00 | 0.00 | 0.00 |
| Q5VTL8 | Pre-mRNA-splicing factor 38B | **3.95** | **0.05** | 9.37 | 20.94 | 36.97 | 19.18 | 0.00 | 0.00 | 0.00 | 46.83 | 0.00 | 39.68 | 55.53 | 43.46 | 0.00 | 37.39 | 45.77 |
| P51911 | Calponin-1 | **3.80** | **0.05** | 62.80 | 75.38 | 238.97 | 161.77 | 77.19 | 53.43 | 183.40 | 0.00 | 0.00 | 165.67 | 358.18 | 414.67 | 355.19 | 140.10 | 0.00 |
| Q09160 | HLA class I histocompatibility antigen, A-80 alpha chain | **1.94** | **0.05** | 61.93 | 41.87 | 120.07 | 42.60 | 51.40 | 115.62 | 73.36 | 69.28 | 0.00 | 79.99 | 155.21 | 177.95 | 137.37 | 92.63 | 77.29 |
| Q9NXH8 | Torsin-4A | **0.36** | **0.05** | 30563.65 | 20196.69 | 11067.82 | 5930.49 | 22665.80 | 32094.65 | 23685.75 | 10428.87 | 63943.15 | 11403.05 | 17028.46 | 17.71 | 12043.63 | 15194.99 | 10719.10 |
| O94921 | Cyclin-dependent kinase 14 | **6.21** | **0.05** | 6.40 | 14.31 | 39.74 | 31.13 | 0.00 | 0.00 | 31.99 | 0.00 | 0.00 | 53.83 | 55.22 | 0.00 | 0.00 | 65.94 | 63.46 |
| P09769 | Tyrosine-protein kinase Fgr | **6.21** | **0.05** | 6.40 | 14.31 | 39.74 | 31.13 | 0.00 | 0.00 | 31.99 | 0.00 | 0.00 | 53.83 | 55.22 | 0.00 | 0.00 | 65.94 | 63.46 |
| P11802 | Cyclin-dependent kinase 4 | **6.21** | **0.05** | 6.40 | 14.31 | 39.74 | 31.13 | 0.00 | 0.00 | 31.99 | 0.00 | 0.00 | 53.83 | 55.22 | 0.00 | 0.00 | 65.94 | 63.46 |
| P20794 | Serine/threonine-protein kinase MAK | **6.21** | **0.05** | 6.40 | 14.31 | 39.74 | 31.13 | 0.00 | 0.00 | 31.99 | 0.00 | 0.00 | 53.83 | 55.22 | 0.00 | 0.00 | 65.94 | 63.46 |
| P50750 | Cyclin-dependent kinase 9 | **6.21** | **0.05** | 6.40 | 14.31 | 39.74 | 31.13 | 0.00 | 0.00 | 31.99 | 0.00 | 0.00 | 53.83 | 55.22 | 0.00 | 0.00 | 65.94 | 63.46 |
| P51451 | Tyrosine-protein kinase Blk | **6.21** | **0.05** | 6.40 | 14.31 | 39.74 | 31.13 | 0.00 | 0.00 | 31.99 | 0.00 | 0.00 | 53.83 | 55.22 | 0.00 | 0.00 | 65.94 | 63.46 |
| Q00534 | Cyclin-dependent kinase 6 | **6.21** | **0.05** | 6.40 | 14.31 | 39.74 | 31.13 | 0.00 | 0.00 | 31.99 | 0.00 | 0.00 | 53.83 | 55.22 | 0.00 | 0.00 | 65.94 | 63.46 |
| Q00535 | Cyclin-dependent kinase 5 | **6.21** | **0.05** | 6.40 | 14.31 | 39.74 | 31.13 | 0.00 | 0.00 | 31.99 | 0.00 | 0.00 | 53.83 | 55.22 | 0.00 | 0.00 | 65.94 | 63.46 |
| Q00536 | Cyclin-dependent kinase 16 | **6.21** | **0.05** | 6.40 | 14.31 | 39.74 | 31.13 | 0.00 | 0.00 | 31.99 | 0.00 | 0.00 | 53.83 | 55.22 | 0.00 | 0.00 | 65.94 | 63.46 |
| Q00537 | Cyclin-dependent kinase 17 | **6.21** | **0.05** | 6.40 | 14.31 | 39.74 | 31.13 | 0.00 | 0.00 | 31.99 | 0.00 | 0.00 | 53.83 | 55.22 | 0.00 | 0.00 | 65.94 | 63.46 |
| Q07002 | Cyclin-dependent kinase 18 | **6.21** | **0.05** | 6.40 | 14.31 | 39.74 | 31.13 | 0.00 | 0.00 | 31.99 | 0.00 | 0.00 | 53.83 | 55.22 | 0.00 | 0.00 | 65.94 | 63.46 |
| Q14004 | Cyclin-dependent kinase 13 | **6.21** | **0.05** | 6.40 | 14.31 | 39.74 | 31.13 | 0.00 | 0.00 | 31.99 | 0.00 | 0.00 | 53.83 | 55.22 | 0.00 | 0.00 | 65.94 | 63.46 |
| Q8IZL9 | Cyclin-dependent kinase 20 | **6.21** | **0.05** | 6.40 | 14.31 | 39.74 | 31.13 | 0.00 | 0.00 | 31.99 | 0.00 | 0.00 | 53.83 | 55.22 | 0.00 | 0.00 | 65.94 | 63.46 |
| Q96Q40 | Cyclin-dependent kinase 15 | **6.21** | **0.05** | 6.40 | 14.31 | 39.74 | 31.13 | 0.00 | 0.00 | 31.99 | 0.00 | 0.00 | 53.83 | 55.22 | 0.00 | 0.00 | 65.94 | 63.46 |
| Q9NYV4 | Cyclin-dependent kinase 12 | **6.21** | **0.05** | 6.40 | 14.31 | 39.74 | 31.13 | 0.00 | 0.00 | 31.99 | 0.00 | 0.00 | 53.83 | 55.22 | 0.00 | 0.00 | 65.94 | 63.46 |
| Q9UPZ9 | Serine/threonine-protein kinase ICK | **6.21** | **0.05** | 6.40 | 14.31 | 39.74 | 31.13 | 0.00 | 0.00 | 31.99 | 0.00 | 0.00 | 53.83 | 55.22 | 0.00 | 0.00 | 65.94 | 63.46 |
| O95741 | Copine-6 | **2.18** | **0.05** | 25.38 | 23.46 | 55.27 | 20.72 | 46.29 | 0.00 | 0.00 | 36.38 | 44.21 | 44.22 | 81.50 | 39.29 | 29.71 | 62.13 | 74.78 |
| Q8IYJ1 | Copine-9 | **2.18** | **0.05** | 25.38 | 23.46 | 55.27 | 20.72 | 46.29 | 0.00 | 0.00 | 36.38 | 44.21 | 44.22 | 81.50 | 39.29 | 29.71 | 62.13 | 74.78 |
| Q96A23 | Copine-4 | **2.18** | **0.05** | 25.38 | 23.46 | 55.27 | 20.72 | 46.29 | 0.00 | 0.00 | 36.38 | 44.21 | 44.22 | 81.50 | 39.29 | 29.71 | 62.13 | 74.78 |
| Q96FN4 | Copine-2 | **2.18** | **0.05** | 25.38 | 23.46 | 55.27 | 20.72 | 46.29 | 0.00 | 0.00 | 36.38 | 44.21 | 44.22 | 81.50 | 39.29 | 29.71 | 62.13 | 74.78 |
| Q9HCH3 | Copine-5 | **2.18** | **0.05** | 25.38 | 23.46 | 55.27 | 20.72 | 46.29 | 0.00 | 0.00 | 36.38 | 44.21 | 44.22 | 81.50 | 39.29 | 29.71 | 62.13 | 74.78 |
| Q9UBL6 | Copine-7 | **2.18** | **0.05** | 25.38 | 23.46 | 55.27 | 20.72 | 46.29 | 0.00 | 0.00 | 36.38 | 44.21 | 44.22 | 81.50 | 39.29 | 29.71 | 62.13 | 74.78 |
| Q6S8J3 | POTE ankyrin domain family member E | **1.40** | **0.05** | 5740.32 | 2024.03 | 8018.84 | 1325.48 | 6642.39 | 3465.42 | 6803.27 | 8042.36 | 3748.16 | 6882.28 | 7210.71 | 9617.21 | 9675.92 | 7946.49 | 6780.40 |
| P14854 | Cytochrome c oxidase subunit 6B1 | **0.20** | **0.05** | 82.02 | 53.77 | 16.67 | 18.67 | 159.62 | 67.91 | 112.37 | 38.58 | 31.64 | 0.00 | 27.93 | 0.00 | 0.00 | 40.05 | 32.02 |
| P26639 | Threonine--tRNA ligase, cytoplasmic | **4.24** | **0.05** | 5.91 | 13.21 | 25.06 | 14.79 | 0.00 | 0.00 | 0.00 | 29.53 | 0.00 | 23.07 | 27.44 | 43.97 | 21.56 | 0.00 | 34.36 |
| P68363 | Tubulin alpha-1B chain | **1.61** | **0.05** | 1261.02 | 684.21 | 2029.04 | 448.82 | 1295.33 | 570.13 | 1496.96 | 2261.89 | 680.78 | 1606.82 | 2117.76 | 1706.84 | 1669.57 | 2727.33 | 2345.91 |
| Q71U36 | Tubulin alpha-1A chain | **1.61** | **0.05** | 1261.02 | 684.21 | 2029.04 | 448.82 | 1295.33 | 570.13 | 1496.96 | 2261.89 | 680.78 | 1606.82 | 2117.76 | 1706.84 | 1669.57 | 2727.33 | 2345.91 |
| Q9BQE3 | Tubulin alpha-1C chain | **1.61** | **0.05** | 1261.02 | 684.21 | 2029.04 | 448.82 | 1295.33 | 570.13 | 1496.96 | 2261.89 | 680.78 | 1606.82 | 2117.76 | 1706.84 | 1669.57 | 2727.33 | 2345.91 |
| P61604 | 10 kDa heat shock protein, mitochondrial | **0.52** | **0.05** | 374.01 | 148.37 | 193.48 | 120.01 | 618.62 | 405.25 | 312.89 | 251.45 | 281.86 | 358.77 | 211.16 | 170.90 | 0.00 | 155.77 | 264.29 |
| P67812 | Signal peptidase complex catalytic subunit SEC11A | **2.65** | **0.05** | 11.70 | 16.19 | 30.98 | 8.08 | 0.00 | 0.00 | 25.93 | 32.57 | 0.00 | 39.14 | 41.67 | 28.87 | 28.57 | 19.77 | 27.83 |
| O43390 | Heterogeneous nuclear ribonucleoprotein R | **1.66** | **0.05** | 102.59 | 61.65 | 170.04 | 39.16 | 74.38 | 49.68 | 152.25 | 184.07 | 52.56 | 184.55 | 164.72 | 96.27 | 174.58 | 191.05 | 209.08 |
| Q08J23 | tRNA (cytosine(34)-C(5))-methyltransferase | **6.52** | **0.06** | 2.24 | 5.01 | 14.60 | 11.93 | 0.00 | 0.00 | 11.20 | 0.00 | 0.00 | 25.10 | 22.78 | 0.00 | 0.00 | 14.71 | 25.01 |
| P07237 | Protein disulfide-isomerase | **1.58** | **0.06** | 331.82 | 112.94 | 523.96 | 164.83 | 452.26 | 198.97 | 260.31 | 445.31 | 302.25 | 508.43 | 721.11 | 402.62 | 401.04 | 375.08 | 735.46 |
| P49917 | DNA ligase 4 | **10.82** | **0.06** | 14.67 | 32.81 | 158.74 | 142.26 | 73.37 | 0.00 | 0.00 | 0.00 | 0.00 | 129.58 | 224.30 | 243.94 | 354.65 | 0.00 | 0.00 |
| Q16555 | Dihydropyrimidinase-related protein 2 | **5.90** | **0.06** | 10.12 | 18.16 | 59.72 | 48.51 | 8.70 | 0.00 | 41.90 | 0.00 | 0.00 | 0.00 | 28.70 | 83.28 | 127.65 | 91.52 | 27.19 |
| Q15063 | Periostin | **13.98** | **0.06** | 35.19 | 39.59 | 491.83 | 450.82 | 30.95 | 0.00 | 94.75 | 50.23 | 0.00 | 214.89 | 464.65 | 1149.45 | 917.94 | 204.04 | 0.00 |
| P12004 | Proliferating cell nuclear antigen | **3.20** | **0.06** | 22.66 | 23.46 | 72.46 | 45.96 | 22.67 | 0.00 | 36.65 | 53.96 | 0.00 | 57.64 | 120.48 | 46.88 | 0.00 | 101.00 | 108.78 |
| Q15181 | Inorganic pyrophosphatase | **0.60** | **0.06** | 78.37 | 21.42 | 46.67 | 25.91 | 100.10 | 49.94 | 99.37 | 68.81 | 73.61 | 45.06 | 32.94 | 95.08 | 25.47 | 53.03 | 28.45 |
| P21802 | Fibroblast growth factor receptor 2 | **5.73** | **0.06** | 6.40 | 14.31 | 36.68 | 29.18 | 0.00 | 0.00 | 31.99 | 0.00 | 0.00 | 53.83 | 55.22 | 0.00 | 0.00 | 65.94 | 45.11 |
| P22455 | Fibroblast growth factor receptor 4 | **5.73** | **0.06** | 6.40 | 14.31 | 36.68 | 29.18 | 0.00 | 0.00 | 31.99 | 0.00 | 0.00 | 53.83 | 55.22 | 0.00 | 0.00 | 65.94 | 45.11 |
| P35606 | Coatomer subunit beta' | **2.10** | **0.06** | 20.77 | 20.44 | 43.52 | 14.36 | 24.69 | 0.00 | 33.01 | 46.13 | 0.00 | 19.56 | 56.28 | 50.90 | 55.10 | 33.50 | 45.79 |
| P13798 | Acylamino-acid-releasing enzyme | **0.28** | **0.06** | 34.36 | 22.81 | 9.59 | 14.91 | 63.35 | 0.00 | 33.13 | 41.94 | 33.40 | 30.68 | 0.00 | 0.00 | 0.00 | 0.00 | 26.88 |
| A5A3E0 | POTE ankyrin domain family member F | **1.39** | **0.06** | 5690.14 | 2077.92 | 7921.78 | 1314.43 | 6642.39 | 3246.60 | 6803.27 | 8010.27 | 3748.16 | 6882.28 | 6915.25 | 9600.85 | 9477.63 | 7874.29 | 6780.40 |
| O95777 | N-alpha-acetyltransferase 38, NatC auxiliary subunit | **5.42** | **0.06** | 4.45 | 9.94 | 24.09 | 18.92 | 0.00 | 0.00 | 0.00 | 22.23 | 0.00 | 41.60 | 35.36 | 0.00 | 0.00 | 35.74 | 31.86 |
| Q04695 | Keratin, type I cytoskeletal 17 | **0.58** | **0.06** | 1415.60 | 312.58 | 826.69 | 534.05 | 1759.74 | 1117.41 | 1252.18 | 1200.61 | 1748.08 | 1441.67 | 677.81 | 1538.05 | 305.09 | 626.24 | 371.31 |
| O43681 | ATPase ASNA1 | **6.86** | **0.06** | 3.73 | 8.34 | 25.58 | 21.22 | 0.00 | 0.00 | 0.00 | 0.00 | 18.64 | 29.82 | 39.84 | 32.18 | 0.00 | 0.00 | 51.63 |
| Q9UHQ9 | NADH-cytochrome b5 reductase 1 | **0.27** | **0.06** | 56.30 | 41.05 | 15.42 | 20.56 | 48.97 | 0.00 | 115.45 | 60.37 | 56.70 | 52.18 | 21.58 | 0.00 | 0.00 | 0.00 | 18.77 |
| Q12931 | Heat shock protein 75 kDa, mitochondrial | **1.88** | **0.06** | 617.19 | 245.08 | 1163.38 | 517.65 | 1008.06 | 532.82 | 359.06 | 513.78 | 672.24 | 923.39 | 1705.15 | 1162.00 | 531.84 | 1846.18 | 811.75 |
| P08779 | Keratin, type I cytoskeletal 16 | **0.56** | **0.06** | 1341.38 | 342.70 | 756.07 | 518.28 | 1759.74 | 1117.41 | 1070.11 | 1089.94 | 1669.68 | 1487.61 | 598.47 | 1274.81 | 144.96 | 626.24 | 404.36 |
| Q15746 | Myosin light chain kinase, smooth muscle | **8.66** | **0.06** | 7.01 | 15.68 | 60.75 | 53.95 | 0.00 | 0.00 | 0.00 | 35.07 | 0.00 | 27.39 | 49.39 | 166.71 | 63.37 | 33.27 | 24.35 |
| P35908 | Keratin, type II cytoskeletal 2 epidermal | **0.57** | **0.06** | 1215.52 | 448.45 | 688.89 | 369.91 | 1923.83 | 849.14 | 834.18 | 1340.77 | 1129.65 | 1374.43 | 537.75 | 576.21 | 274.86 | 625.05 | 745.03 |
| Q13410 | Butyrophilin subfamily 1 member A1 | **3.52** | **0.06** | 32.41 | 48.29 | 114.07 | 73.09 | 0.00 | 0.00 | 107.96 | 54.11 | 0.00 | 79.70 | 127.11 | 175.43 | 205.13 | 97.03 | 0.00 |
| P04259 | Keratin, type II cytoskeletal 6B | **0.52** | **0.06** | 1561.97 | 608.04 | 810.32 | 563.63 | 2526.98 | 869.50 | 1346.97 | 1633.76 | 1432.65 | 1892.38 | 611.82 | 613.73 | 233.03 | 765.92 | 745.03 |
| Q9BS26 | Endoplasmic reticulum resident protein 44 | **2.64** | **0.06** | 13.11 | 18.21 | 34.67 | 15.52 | 28.41 | 0.00 | 0.00 | 37.13 | 0.00 | 28.56 | 61.37 | 45.56 | 25.72 | 24.42 | 22.38 |
| P24539 | ATP synthase F(0) complex subunit B1, mitochondrial | **1.71** | **0.06** | 59.42 | 43.26 | 101.40 | 20.45 | 55.38 | 0.00 | 108.39 | 93.47 | 39.85 | 122.37 | 118.96 | 103.47 | 67.21 | 89.56 | 106.83 |
| Q9Y2S6 | Translation machinery-associated protein 7 | **1.75** | **0.06** | 646.43 | 479.26 | 1128.97 | 263.53 | 725.54 | 319.63 | 327.14 | 1450.99 | 408.87 | 1291.14 | 1180.65 | 1513.82 | 1014.35 | 1028.10 | 745.75 |
| P02533 | Keratin, type I cytoskeletal 14 | **0.58** | **0.06** | 1413.40 | 314.06 | 818.15 | 555.12 | 1759.74 | 1117.41 | 1241.13 | 1200.61 | 1748.08 | 1487.61 | 598.47 | 1538.05 | 254.19 | 626.24 | 404.36 |
| O76015 | Keratin, type I cuticular Ha8 | **0.56** | **0.06** | 1216.72 | 457.37 | 686.09 | 373.83 | 1930.64 | 946.42 | 789.43 | 1397.35 | 1019.77 | 1101.23 | 745.19 | 972.73 | 125.12 | 816.45 | 355.83 |
| P68036 | Ubiquitin-conjugating enzyme E2 L3 | **0.53** | **0.06** | 94.86 | 43.49 | 49.92 | 26.44 | 158.65 | 106.81 | 41.07 | 94.38 | 73.37 | 75.83 | 49.42 | 0.00 | 49.06 | 64.76 | 60.48 |
| P25205 | DNA replication licensing factor MCM3 | **13.08** | **0.06** | 1.30 | 2.91 | 17.03 | 16.31 | 0.00 | 0.00 | 0.00 | 6.51 | 0.00 | 41.63 | 11.88 | 0.00 | 0.00 | 22.02 | 26.66 |
| P61254 | 60S ribosomal protein L26 | **3.34** | **0.06** | 40.42 | 45.65 | 134.95 | 90.38 | 0.00 | 0.00 | 70.27 | 103.84 | 28.01 | 121.61 | 284.30 | 25.49 | 77.16 | 186.27 | 114.86 |
| Q9HB71 | Calcyclin-binding protein | **4.64** | **0.07** | 9.73 | 16.68 | 45.17 | 34.23 | 0.00 | 0.00 | 10.11 | 38.53 | 0.00 | 40.14 | 58.23 | 32.09 | 0.00 | 37.22 | 103.36 |
| P10321 | HLA class I histocompatibility antigen, Cw-7 alpha chain | **2.17** | **0.07** | 65.90 | 46.37 | 143.24 | 70.34 | 39.76 | 140.85 | 74.42 | 54.32 | 20.13 | 54.56 | 211.01 | 192.84 | 200.18 | 138.57 | 62.27 |
| P48668 | Keratin, type II cytoskeletal 6C | **0.52** | **0.07** | 1557.43 | 614.56 | 809.58 | 563.70 | 2526.98 | 846.79 | 1346.97 | 1633.76 | 1432.65 | 1892.38 | 611.82 | 613.73 | 233.03 | 761.47 | 745.03 |
| Q92673 | Sortilin-related receptor | **0.48** | **0.07** | 1038.58 | 417.64 | 496.09 | 437.99 | 1601.63 | 708.00 | 709.43 | 1368.32 | 805.54 | 871.45 | 0.00 | 344.31 | 818.40 | 0.00 | 942.37 |
| Q01546 | Keratin, type II cytoskeletal 2 oral | **0.46** | **0.07** | 1687.91 | 813.83 | 782.53 | 363.84 | 2816.20 | 985.11 | 2284.08 | 1227.01 | 1127.16 | 1274.40 | 660.18 | 706.99 | 198.78 | 836.48 | 1018.37 |
| P06493 | Cyclin-dependent kinase 1 | **5.66** | **0.07** | 6.40 | 14.31 | 36.23 | 29.04 | 0.00 | 0.00 | 31.99 | 0.00 | 0.00 | 53.83 | 55.22 | 0.00 | 0.00 | 65.94 | 42.38 |
| P24941 | Cyclin-dependent kinase 2 | **5.66** | **0.07** | 6.40 | 14.31 | 36.23 | 29.04 | 0.00 | 0.00 | 31.99 | 0.00 | 0.00 | 53.83 | 55.22 | 0.00 | 0.00 | 65.94 | 42.38 |
| Q00526 | Cyclin-dependent kinase 3 | **5.66** | **0.07** | 6.40 | 14.31 | 36.23 | 29.04 | 0.00 | 0.00 | 31.99 | 0.00 | 0.00 | 53.83 | 55.22 | 0.00 | 0.00 | 65.94 | 42.38 |
| Q86YQ8 | Copine-8 | **2.03** | **0.07** | 25.34 | 23.44 | 51.49 | 18.38 | 46.29 | 0.00 | 0.00 | 36.20 | 44.21 | 44.22 | 81.50 | 39.29 | 29.71 | 52.14 | 62.09 |
| P27797 | Calreticulin | **1.42** | **0.07** | 354.25 | 102.27 | 502.73 | 129.08 | 315.99 | 210.77 | 378.67 | 491.28 | 374.54 | 515.40 | 739.48 | 378.36 | 405.00 | 518.25 | 459.89 |
| P11362 | Fibroblast growth factor receptor 1 | **5.63** | **0.07** | 6.40 | 14.31 | 36.04 | 28.99 | 0.00 | 0.00 | 31.99 | 0.00 | 0.00 | 53.83 | 55.22 | 0.00 | 0.00 | 65.94 | 41.26 |
| P22607 | Fibroblast growth factor receptor 3 | **5.63** | **0.07** | 6.40 | 14.31 | 36.04 | 28.99 | 0.00 | 0.00 | 31.99 | 0.00 | 0.00 | 53.83 | 55.22 | 0.00 | 0.00 | 65.94 | 41.26 |
| P02538 | Keratin, type II cytoskeletal 6A | **0.53** | **0.07** | 1566.03 | 616.19 | 831.61 | 557.06 | 2526.98 | 846.79 | 1346.97 | 1676.78 | 1432.65 | 1892.38 | 611.82 | 745.93 | 233.03 | 761.47 | 745.03 |
| P27105 | Erythrocyte band 7 integral membrane protein | **3.99** | **0.07** | 15.13 | 23.48 | 60.32 | 43.83 | 22.21 | 0.00 | 53.45 | 0.00 | 0.00 | 33.76 | 99.45 | 130.75 | 36.18 | 37.82 | 23.98 |
| P25054 | Adenomatous polyposis coli protein | **1.66** | **0.07** | 55.19 | 37.99 | 91.65 | 19.29 | 58.94 | 0.00 | 77.21 | 99.86 | 39.95 | 90.53 | 100.64 | 70.40 | 69.69 | 98.85 | 119.78 |
| P09960 | Leukotriene A-4 hydrolase | **3.74** | **0.07** | 18.36 | 17.35 | 68.71 | 52.10 | 30.44 | 0.00 | 24.30 | 37.05 | 0.00 | 28.09 | 84.43 | 84.93 | 29.17 | 26.89 | 158.71 |
| Q96CX2 | BTB/POZ domain-containing protein KCTD12 | **4.30** | **0.07** | 8.70 | 19.46 | 37.42 | 25.83 | 0.00 | 0.00 | 43.50 | 0.00 | 0.00 | 34.44 | 27.68 | 79.88 | 41.82 | 40.70 | 0.00 |
| Q92542 | Nicastrin | **10.32** | **0.07** | 13.88 | 20.67 | 143.23 | 138.71 | 46.19 | 0.00 | 0.00 | 23.21 | 0.00 | 129.58 | 131.21 | 243.94 | 354.65 | 0.00 | 0.00 |
| A8MTJ3 | Guanine nucleotide-binding protein G(t) subunit alpha-3 | **2.94** | **0.07** | 257.20 | 286.01 | 757.24 | 476.47 | 0.00 | 0.00 | 493.62 | 620.21 | 172.17 | 631.40 | 216.02 | 691.13 | 1627.62 | 854.50 | 522.79 |
| P11488 | Guanine nucleotide-binding protein G(t) subunit alpha-1 | **2.94** | **0.07** | 257.20 | 286.01 | 757.24 | 476.47 | 0.00 | 0.00 | 493.62 | 620.21 | 172.17 | 631.40 | 216.02 | 691.13 | 1627.62 | 854.50 | 522.79 |
| P19087 | Guanine nucleotide-binding protein G(t) subunit alpha-2 | **2.94** | **0.07** | 257.20 | 286.01 | 757.24 | 476.47 | 0.00 | 0.00 | 493.62 | 620.21 | 172.17 | 631.40 | 216.02 | 691.13 | 1627.62 | 854.50 | 522.79 |
| P38405 | Guanine nucleotide-binding protein G(olf) subunit alpha | **2.94** | **0.07** | 257.20 | 286.01 | 757.24 | 476.47 | 0.00 | 0.00 | 493.62 | 620.21 | 172.17 | 631.40 | 216.02 | 691.13 | 1627.62 | 854.50 | 522.79 |
| Q03113 | Guanine nucleotide-binding protein subunit alpha-12 | **2.94** | **0.07** | 257.20 | 286.01 | 757.24 | 476.47 | 0.00 | 0.00 | 493.62 | 620.21 | 172.17 | 631.40 | 216.02 | 691.13 | 1627.62 | 854.50 | 522.79 |
| Q14344 | Guanine nucleotide-binding protein subunit alpha-13 | **2.94** | **0.07** | 257.20 | 286.01 | 757.24 | 476.47 | 0.00 | 0.00 | 493.62 | 620.21 | 172.17 | 631.40 | 216.02 | 691.13 | 1627.62 | 854.50 | 522.79 |
| P01903 | HLA class II histocompatibility antigen, DR alpha chain | **8.68** | **0.07** | 9.88 | 22.09 | 85.81 | 79.97 | 49.40 | 0.00 | 0.00 | 0.00 | 0.00 | 0.00 | 218.01 | 134.94 | 84.76 | 49.91 | 27.22 |
| P05455 | Lupus La protein | **1.61** | **0.07** | 60.04 | 29.01 | 96.96 | 30.54 | 24.77 | 50.02 | 95.62 | 83.74 | 46.07 | 101.50 | 115.51 | 93.77 | 46.30 | 87.33 | 137.34 |
| Q9BXX0 | EMILIN-2 | **16.93** | **0.07** | 31.36 | 70.13 | 531.12 | 541.87 | 156.82 | 0.00 | 0.00 | 0.00 | 0.00 | 566.73 | 314.03 | 1561.29 | 486.24 | 258.40 | 0.00 |
| P82987 | ADAMTS-like protein 3 | **0.19** | **0.07** | 3616.26 | 3526.78 | 677.64 | 571.31 | 3431.10 | 3208.17 | 1504.58 | 417.09 | 9520.34 | 1595.27 | 0.00 | 333.78 | 943.77 | 850.01 | 343.00 |
| P01906 | HLA class II histocompatibility antigen, DQ alpha 2 chain | **6.80** | **0.08** | 11.72 | 26.21 | 79.67 | 71.10 | 58.60 | 0.00 | 0.00 | 0.00 | 0.00 | 0.00 | 203.04 | 104.52 | 81.74 | 61.48 | 27.22 |
| P07205 | Phosphoglycerate kinase 2 | **0.62** | **0.08** | 600.92 | 209.40 | 371.16 | 170.86 | 615.05 | 543.64 | 447.18 | 953.23 | 445.48 | 461.87 | 103.94 | 287.39 | 397.17 | 613.18 | 363.44 |
| O75683 | Surfeit locus protein 6 | **0.44** | **0.08** | 2397.46 | 981.39 | 1059.77 | 1187.12 | 3900.14 | 1339.80 | 2716.07 | 2219.05 | 1812.23 | 3246.97 | 995.08 | 980.98 | 0.00 | 1135.61 | 0.00 |
| P17658 | Potassium voltage-gated channel subfamily A member 6 | **0.44** | **0.08** | 2397.46 | 981.39 | 1059.77 | 1187.12 | 3900.14 | 1339.80 | 2716.07 | 2219.05 | 1812.23 | 3246.97 | 995.08 | 980.98 | 0.00 | 1135.61 | 0.00 |
| P09471 | Guanine nucleotide-binding protein G(o) subunit alpha | **3.53** | **0.08** | 199.47 | 214.64 | 704.07 | 525.74 | 0.00 | 0.00 | 493.62 | 331.55 | 172.17 | 631.40 | 130.59 | 691.13 | 1627.62 | 854.50 | 289.19 |
| P21333 | Filamin-A | **4.19** | **0.08** | 165.99 | 77.45 | 695.59 | 584.59 | 222.14 | 113.69 | 274.30 | 116.56 | 103.27 | 236.24 | 630.24 | 1614.87 | 1151.06 | 470.09 | 71.03 |
| Q9H0A6 | RING finger protein 32 | **0.45** | **0.08** | 2397.46 | 981.39 | 1084.08 | 1162.31 | 3900.14 | 1339.80 | 2716.07 | 2219.05 | 1812.23 | 3246.97 | 995.08 | 980.98 | 0.00 | 1135.61 | 145.84 |
| Q14974 | Importin subunit beta-1 | **1.57** | **0.08** | 68.09 | 42.99 | 107.18 | 20.36 | 64.65 | 0.00 | 72.55 | 117.02 | 86.24 | 100.77 | 103.34 | 91.63 | 84.68 | 124.24 | 138.46 |
| P53634 | Dipeptidyl peptidase 1 | **11.25** | **0.08** | 5.88 | 13.14 | 66.12 | 67.02 | 0.00 | 0.00 | 0.00 | 29.39 | 0.00 | 109.72 | 70.90 | 0.00 | 0.00 | 43.74 | 172.38 |
| O15264 | Mitogen-activated protein kinase 13 | **5.73** | **0.08** | 5.84 | 13.06 | 33.49 | 28.69 | 0.00 | 0.00 | 0.00 | 29.21 | 0.00 | 27.81 | 33.72 | 86.89 | 0.00 | 28.19 | 24.35 |
| P43490 | Nicotinamide phosphoribosyltransferase | **5.78** | **0.08** | 29.67 | 34.95 | 171.51 | 157.61 | 42.89 | 0.00 | 0.00 | 83.44 | 22.03 | 0.00 | 342.62 | 305.44 | 48.32 | 39.16 | 293.52 |
| Q8TAA3 | Proteasome subunit alpha type-7-like | **1.60** | **0.08** | 54.26 | 34.55 | 86.59 | 18.88 | 64.64 | 0.00 | 78.93 | 85.45 | 42.31 | 55.71 | 102.92 | 96.36 | 73.55 | 87.19 | 103.80 |
| Q9NX62 | Inositol monophosphatase 3 | **12.33** | **0.08** | 43.08 | 96.32 | 531.12 | 541.87 | 0.00 | 0.00 | 0.00 | 0.00 | 215.39 | 566.73 | 314.03 | 1561.29 | 486.24 | 258.40 | 0.00 |
| P36955 | Pigment epithelium-derived factor | **5.68** | **0.08** | 9.23 | 20.63 | 52.37 | 46.54 | 0.00 | 46.14 | 0.00 | 0.00 | 0.00 | 11.79 | 23.82 | 84.47 | 121.82 | 67.72 | 4.61 |
| Q15029 | 116 kDa U5 small nuclear ribonucleoprotein component | **2.50** | **0.08** | 59.23 | 74.74 | 148.22 | 74.19 | 164.45 | 0.00 | 20.20 | 111.49 | 0.00 | 122.32 | 34.68 | 241.57 | 215.99 | 122.92 | 151.84 |
| P23396 | 40S ribosomal protein S3 | **2.05** | **0.08** | 148.33 | 74.55 | 304.55 | 162.21 | 140.29 | 57.32 | 241.60 | 202.05 | 100.40 | 286.22 | 299.64 | 607.52 | 129.26 | 212.17 | 292.50 |
| Q14697 | Neutral alpha-glucosidase AB | **1.74** | **0.08** | 130.73 | 76.93 | 227.01 | 83.84 | 109.54 | 70.73 | 173.91 | 242.02 | 57.45 | 275.87 | 348.85 | 122.15 | 188.99 | 263.51 | 162.70 |
| P60709 | Actin, cytoplasmic 1 | **1.50** | **0.08** | 8477.81 | 3354.74 | 12704.19 | 3711.73 | 10174.57 | 5482.58 | 10344.30 | 12035.77 | 4351.81 | 8276.26 | 15286.01 | 18055.42 | 13228.35 | 12360.53 | 9018.55 |
| P63261 | Actin, cytoplasmic 2 | **1.50** | **0.08** | 8477.81 | 3354.74 | 12704.19 | 3711.73 | 10174.57 | 5482.58 | 10344.30 | 12035.77 | 4351.81 | 8276.26 | 15286.01 | 18055.42 | 13228.35 | 12360.53 | 9018.55 |
| Q13395 | Probable methyltransferase TARBP1 | **21.03** | **0.08** | 10.44 | 23.33 | 219.42 | 236.71 | 0.00 | 0.00 | 52.18 | 0.00 | 0.00 | 26.53 | 386.45 | 610.19 | 189.27 | 104.08 | 0.00 |
| P46977 | Dolichyl-diphosphooligosaccharide--protein glycosyltransferase subunit STT3A | **2.12** | **0.08** | 21.03 | 22.74 | 44.50 | 10.49 | 0.00 | 0.00 | 44.06 | 45.93 | 15.18 | 30.69 | 52.97 | 48.30 | 45.28 | 56.55 | 33.22 |
| P04844 | Dolichyl-diphosphooligosaccharide--protein glycosyltransferase subunit 2 | **1.74** | **0.08** | 47.90 | 37.41 | 83.24 | 22.25 | 35.66 | 0.00 | 75.90 | 94.40 | 33.55 | 64.36 | 121.93 | 70.66 | 80.42 | 66.09 | 95.97 |
| P62913 | 60S ribosomal protein L11 | **1.50** | **0.08** | 166.60 | 73.73 | 250.46 | 68.75 | 153.57 | 84.35 | 203.52 | 272.41 | 119.18 | 181.31 | 366.51 | 229.14 | 201.74 | 228.26 | 295.83 |
| P52895 | Aldo-keto reductase family 1 member C2 | **0.25** | **0.08** | 220.39 | 198.87 | 56.13 | 56.70 | 572.84 | 142.62 | 142.34 | 86.69 | 157.48 | 50.94 | 108.42 | 141.32 | 16.92 | 0.00 | 19.18 |
| P02792 | Ferritin light chain | **43.44** | **0.08** | 3.95 | 8.84 | 171.73 | 191.19 | 0.00 | 0.00 | 0.00 | 0.00 | 19.77 | 92.26 | 536.25 | 151.10 | 198.40 | 31.90 | 20.45 |
| P26373 | 60S ribosomal protein L13 | **1.57** | **0.08** | 130.65 | 72.20 | 205.20 | 38.42 | 96.34 | 58.99 | 195.38 | 220.20 | 82.35 | 221.54 | 241.68 | 207.02 | 130.52 | 216.39 | 214.03 |
| P68871 | Hemoglobin subunit beta | **0.41** | **0.08** | 39294.39 | 28450.37 | 16289.78 | 6472.90 | 34787.30 | 39697.07 | 24167.04 | 11470.65 | 86349.89 | 22273.51 | 15260.04 | 9636.17 | 25475.49 | 15371.45 | 9721.99 |
| P11678 | Eosinophil peroxidase | **4.47** | **0.09** | 24.21 | 30.49 | 108.24 | 94.77 | 31.38 | 73.52 | 0.00 | 16.17 | 0.00 | 33.69 | 152.52 | 140.22 | 258.92 | 64.12 | 0.00 |
| P14625 | Endoplasmin | **1.18** | **0.09** | 505.82 | 54.76 | 598.84 | 94.58 | 440.11 | 516.43 | 461.82 | 574.34 | 536.40 | 686.70 | 627.07 | 517.11 | 507.83 | 726.26 | 528.06 |
| P13611 | Versican core protein | **17.15** | **0.09** | 10.90 | 24.38 | 187.02 | 201.77 | 54.51 | 0.00 | 0.00 | 0.00 | 0.00 | 66.40 | 63.49 | 131.17 | 338.20 | 522.86 | 0.00 |
| Q7Z7H5 | Transmembrane emp24 domain-containing protein 4 | **2.67** | **0.09** | 36.23 | 41.06 | 96.85 | 59.33 | 0.00 | 0.00 | 31.71 | 98.54 | 50.90 | 133.84 | 159.58 | 137.24 | 0.00 | 88.44 | 62.02 |
| Q8IXK2 | Polypeptide N-acetylgalactosaminyltransferase 12 | **4.49** | **0.09** | 10.19 | 22.79 | 45.80 | 35.73 | 50.97 | 0.00 | 0.00 | 0.00 | 0.00 | 68.48 | 0.00 | 68.77 | 62.03 | 75.54 | 0.00 |
| Q9UNX3 | 60S ribosomal protein L26-like 1 | **2.89** | **0.09** | 46.65 | 52.37 | 134.95 | 90.38 | 0.00 | 0.00 | 101.43 | 103.84 | 28.01 | 121.61 | 284.30 | 25.49 | 77.16 | 186.27 | 114.86 |
| Q14315 | Filamin-C | **3.46** | **0.09** | 98.05 | 58.45 | 339.67 | 277.66 | 125.56 | 0.00 | 148.55 | 124.57 | 91.56 | 101.84 | 310.64 | 719.70 | 631.03 | 229.41 | 45.39 |
| O75608 | Acyl-protein thioesterase 1 | **0.44** | **0.09** | 121.56 | 71.50 | 53.93 | 44.49 | 171.53 | 0.00 | 167.45 | 153.64 | 115.17 | 96.34 | 53.35 | 0.00 | 0.00 | 94.06 | 79.85 |
| Q8TDT2 | Probable G-protein coupled receptor 152 | **0.30** | **0.09** | 3074.10 | 2516.04 | 909.67 | 1086.01 | 6531.58 | 2871.18 | 1594.60 | 4373.15 | 0.00 | 2488.49 | 1123.88 | 0.00 | 0.00 | 1845.66 | 0.00 |
| P10620 | Microsomal glutathione S-transferase 1 | **0.50** | **0.09** | 74.91 | 32.25 | 37.76 | 31.68 | 88.92 | 35.59 | 99.53 | 105.36 | 45.18 | 57.84 | 64.64 | 0.00 | 0.00 | 34.26 | 69.85 |
| Q31612 | HLA class I histocompatibility antigen, B-73 alpha chain | **1.95** | **0.09** | 73.13 | 43.77 | 142.78 | 70.72 | 51.40 | 140.85 | 81.24 | 69.28 | 22.89 | 50.25 | 191.64 | 219.33 | 200.18 | 117.97 | 77.29 |
| P35579 | Myosin-9 | **1.74** | **0.09** | 382.89 | 134.45 | 667.18 | 308.14 | 431.78 | 403.66 | 564.24 | 307.86 | 206.90 | 423.31 | 633.31 | 1181.44 | 841.03 | 594.74 | 329.24 |
| Q99497 | Protein DJ-1 | **0.51** | **0.09** | 107.77 | 40.33 | 55.16 | 49.48 | 149.39 | 58.52 | 149.08 | 97.04 | 84.83 | 40.18 | 9.00 | 68.74 | 80.27 | 132.80 | 0.00 |
| P21980 | Protein-glutamine gamma-glutamyltransferase 2 | **7.98** | **0.09** | 22.74 | 20.87 | 181.54 | 185.63 | 36.57 | 0.00 | 41.37 | 35.77 | 0.00 | 27.92 | 82.22 | 429.59 | 127.60 | 401.54 | 20.35 |
| P43304 | Glycerol-3-phosphate dehydrogenase, mitochondrial | **0.27** | **0.09** | 21.22 | 13.09 | 5.67 | 13.89 | 23.41 | 0.00 | 19.22 | 30.21 | 33.28 | 34.03 | 0.00 | 0.00 | 0.00 | 0.00 | 0.00 |
| Q96IX5 | Up-regulated during skeletal muscle growth protein 5 | **0.45** | **0.09** | 59.20 | 19.58 | 26.58 | 33.87 | 74.92 | 45.73 | 82.18 | 57.93 | 35.22 | 84.68 | 0.00 | 0.00 | 0.00 | 35.59 | 39.21 |
| P62249 | 40S ribosomal protein S16 | **1.64** | **0.09** | 83.64 | 52.92 | 136.85 | 40.97 | 80.13 | 0.00 | 109.42 | 143.00 | 85.64 | 119.17 | 140.43 | 179.38 | 66.70 | 142.47 | 172.97 |
| P30626 | Sorcin | **0.40** | **0.09** | 223.04 | 169.74 | 90.17 | 37.57 | 512.91 | 220.53 | 167.78 | 131.70 | 82.27 | 92.71 | 114.14 | 146.51 | 48.24 | 50.92 | 88.51 |
| Q9NZ08 | Endoplasmic reticulum aminopeptidase 1 | **5.03** | **0.09** | 4.09 | 9.15 | 20.58 | 17.65 | 0.00 | 0.00 | 0.00 | 20.47 | 0.00 | 42.42 | 35.58 | 0.00 | 22.99 | 22.47 | 0.00 |
| Q16851 | UTP--glucose-1-phosphate uridylyltransferase | **1.60** | **0.09** | 44.59 | 28.41 | 71.56 | 19.36 | 77.20 | 0.00 | 39.32 | 54.00 | 52.44 | 46.68 | 93.71 | 92.08 | 63.28 | 56.09 | 77.51 |
| P19823 | Inter-alpha-trypsin inhibitor heavy chain H2 | **8.63** | **0.10** | 9.15 | 20.47 | 78.98 | 80.88 | 0.00 | 0.00 | 45.76 | 0.00 | 0.00 | 83.96 | 0.00 | 217.66 | 65.25 | 106.98 | 0.00 |
| Q7Z3Y9 | Keratin, type I cytoskeletal 26 | **4.32** | **0.10** | 53.33 | 41.37 | 230.27 | 210.39 | 32.71 | 111.63 | 0.00 | 55.90 | 66.42 | 77.69 | 95.54 | 594.36 | 132.10 | 104.11 | 377.80 |
| P18085 | ADP-ribosylation factor 4 | **1.37** | **0.10** | 185.54 | 68.81 | 254.68 | 54.23 | 199.32 | 136.29 | 213.34 | 277.49 | 101.29 | 255.47 | 331.05 | 206.60 | 309.59 | 209.69 | 215.68 |
| Q9GIY3 | HLA class II histocompatibility antigen, DRB1-14 beta chain | **5.92** | **0.10** | 8.19 | 18.32 | 48.51 | 45.14 | 40.96 | 0.00 | 0.00 | 0.00 | 0.00 | 41.33 | 102.66 | 98.50 | 48.59 | 0.00 | 0.00 |
| P50851 | Lipopolysaccharide-responsive and beige-like anchor protein | **14.44** | **0.10** | 3.24 | 7.25 | 46.85 | 52.26 | 16.22 | 0.00 | 0.00 | 0.00 | 0.00 | 0.00 | 61.71 | 133.52 | 0.00 | 70.33 | 15.53 |
| A7E2Y1 | Myosin-7B | **3.94** | **0.10** | 76.47 | 108.98 | 301.26 | 251.22 | 0.00 | 0.00 | 233.91 | 148.42 | 0.00 | 0.00 | 335.91 | 741.09 | 324.54 | 274.79 | 131.25 |
| P11055 | Myosin-3 | **3.94** | **0.10** | 76.47 | 108.98 | 301.26 | 251.22 | 0.00 | 0.00 | 233.91 | 148.42 | 0.00 | 0.00 | 335.91 | 741.09 | 324.54 | 274.79 | 131.25 |
| P12883 | Myosin-7 | **3.94** | **0.10** | 76.47 | 108.98 | 301.26 | 251.22 | 0.00 | 0.00 | 233.91 | 148.42 | 0.00 | 0.00 | 335.91 | 741.09 | 324.54 | 274.79 | 131.25 |
| P13533 | Myosin-6 | **3.94** | **0.10** | 76.47 | 108.98 | 301.26 | 251.22 | 0.00 | 0.00 | 233.91 | 148.42 | 0.00 | 0.00 | 335.91 | 741.09 | 324.54 | 274.79 | 131.25 |
| C9JRZ8 | Aldo-keto reductase family 1 member B15 | **3.03** | **0.10** | 41.16 | 70.44 | 124.64 | 77.74 | 0.00 | 0.00 | 0.00 | 162.65 | 43.16 | 226.85 | 150.06 | 166.10 | 81.45 | 0.00 | 123.37 |
| Q9NVD7 | Alpha-parvin | **7.17** | **0.10** | 3.34 | 7.48 | 23.99 | 23.98 | 16.72 | 0.00 | 0.00 | 0.00 | 0.00 | 0.00 | 18.42 | 62.01 | 39.73 | 23.79 | 0.00 |
| P45983 | Mitogen-activated protein kinase 8 | **4.76** | **0.10** | 7.01 | 15.68 | 33.42 | 28.71 | 0.00 | 0.00 | 0.00 | 35.07 | 0.00 | 27.39 | 33.72 | 86.89 | 0.00 | 28.19 | 24.35 |
| P45984 | Mitogen-activated protein kinase 9 | **4.76** | **0.10** | 7.01 | 15.68 | 33.42 | 28.71 | 0.00 | 0.00 | 0.00 | 35.07 | 0.00 | 27.39 | 33.72 | 86.89 | 0.00 | 28.19 | 24.35 |
| P53778 | Mitogen-activated protein kinase 12 | **4.76** | **0.10** | 7.01 | 15.68 | 33.42 | 28.71 | 0.00 | 0.00 | 0.00 | 35.07 | 0.00 | 27.39 | 33.72 | 86.89 | 0.00 | 28.19 | 24.35 |
| P53779 | Mitogen-activated protein kinase 10 | **4.76** | **0.10** | 7.01 | 15.68 | 33.42 | 28.71 | 0.00 | 0.00 | 0.00 | 35.07 | 0.00 | 27.39 | 33.72 | 86.89 | 0.00 | 28.19 | 24.35 |
| Q15759 | Mitogen-activated protein kinase 11 | **4.76** | **0.10** | 7.01 | 15.68 | 33.42 | 28.71 | 0.00 | 0.00 | 0.00 | 35.07 | 0.00 | 27.39 | 33.72 | 86.89 | 0.00 | 28.19 | 24.35 |
| Q16539 | Mitogen-activated protein kinase 14 | **4.76** | **0.10** | 7.01 | 15.68 | 33.42 | 28.71 | 0.00 | 0.00 | 0.00 | 35.07 | 0.00 | 27.39 | 33.72 | 86.89 | 0.00 | 28.19 | 24.35 |
| Q32MK0 | Myosin light chain kinase 3 | **4.76** | **0.10** | 7.01 | 15.68 | 33.42 | 28.71 | 0.00 | 0.00 | 0.00 | 35.07 | 0.00 | 27.39 | 33.72 | 86.89 | 0.00 | 28.19 | 24.35 |
| Q86YV6 | Myosin light chain kinase family member 4 | **4.76** | **0.10** | 7.01 | 15.68 | 33.42 | 28.71 | 0.00 | 0.00 | 0.00 | 35.07 | 0.00 | 27.39 | 33.72 | 86.89 | 0.00 | 28.19 | 24.35 |
| Q9H1R3 | Myosin light chain kinase 2, skeletal/cardiac muscle | **4.76** | **0.10** | 7.01 | 15.68 | 33.42 | 28.71 | 0.00 | 0.00 | 0.00 | 35.07 | 0.00 | 27.39 | 33.72 | 86.89 | 0.00 | 28.19 | 24.35 |
| Q14194 | Dihydropyrimidinase-related protein 1 | **5.84** | **0.10** | 9.82 | 21.96 | 57.38 | 54.30 | 0.00 | 0.00 | 49.10 | 0.00 | 0.00 | 0.00 | 43.62 | 105.28 | 132.51 | 62.88 | 0.00 |
| Q96AX2 | Ras-related protein Rab-37 | **1.63** | **0.10** | 339.33 | 109.76 | 553.17 | 240.19 | 344.40 | 275.99 | 219.34 | 511.72 | 345.18 | 959.06 | 608.97 | 403.64 | 347.52 | 341.37 | 658.42 |
| Q04828 | Aldo-keto reductase family 1 member C1 | **0.27** | **0.10** | 214.50 | 201.43 | 58.28 | 59.26 | 572.84 | 142.62 | 142.34 | 90.71 | 123.98 | 50.94 | 121.31 | 141.32 | 16.92 | 0.00 | 19.18 |
| P49257 | Protein ERGIC-53 | **2.26** | **0.10** | 23.51 | 21.88 | 53.11 | 30.35 | 40.08 | 0.00 | 32.78 | 44.71 | 0.00 | 45.30 | 87.58 | 71.77 | 65.90 | 0.00 | 48.10 |
| P30050 | 60S ribosomal protein L12 | **1.48** | **0.10** | 155.31 | 82.56 | 229.45 | 52.40 | 93.41 | 101.62 | 240.26 | 250.82 | 90.43 | 218.40 | 255.64 | 225.02 | 158.52 | 204.52 | 314.59 |
| O43175 | D-3-phosphoglycerate dehydrogenase | **21.69** | **0.10** | 6.32 | 9.83 | 137.09 | 160.00 | 22.39 | 0.00 | 0.00 | 9.22 | 0.00 | 152.01 | 117.90 | 0.00 | 0.00 | 116.55 | 436.09 |
| Q86YJ6 | Threonine synthase-like 2 | **2.69** | **0.10** | 148.21 | 205.03 | 399.34 | 246.50 | 329.22 | 0.00 | 0.00 | 411.81 | 0.00 | 500.38 | 396.14 | 0.00 | 403.41 | 336.08 | 760.02 |
| P26641 | Elongation factor 1-gamma | **1.65** | **0.10** | 102.88 | 75.23 | 170.24 | 47.56 | 69.27 | 0.00 | 167.03 | 184.84 | 93.24 | 135.71 | 220.27 | 123.03 | 123.88 | 199.46 | 219.07 |
| P62316 | Small nuclear ribonucleoprotein Sm D2 | **1.57** | **0.10** | 77.40 | 40.04 | 121.52 | 40.44 | 71.97 | 51.02 | 80.35 | 143.12 | 40.51 | 148.87 | 131.56 | 99.03 | 52.09 | 132.73 | 164.83 |
| Q01082 | Spectrin beta chain, non-erythrocytic 1 | **1.76** | **0.11** | 55.30 | 22.16 | 97.41 | 47.74 | 73.45 | 42.60 | 41.05 | 84.52 | 34.90 | 156.25 | 25.43 | 67.30 | 83.39 | 128.04 | 124.06 |
| Q9Y678 | Coatomer subunit gamma-1 | **2.79** | **0.11** | 11.82 | 17.32 | 32.99 | 20.99 | 0.00 | 0.00 | 20.86 | 38.25 | 0.00 | 0.00 | 61.96 | 47.19 | 30.72 | 33.40 | 24.67 |
| P61619 | Protein transport protein Sec61 subunit alpha isoform 1 | **2.39** | **0.11** | 23.12 | 31.74 | 55.22 | 27.57 | 0.00 | 0.00 | 54.79 | 60.83 | 0.00 | 59.39 | 74.05 | 0.00 | 63.55 | 63.58 | 70.73 |
| P02794 | Ferritin heavy chain | **3.12** | **0.11** | 38.95 | 38.42 | 121.69 | 96.23 | 73.37 | 79.91 | 0.00 | 0.00 | 41.46 | 150.53 | 99.80 | 243.94 | 205.13 | 30.75 | 0.00 |
| Q9Y490 | Talin-1 | **2.68** | **0.11** | 69.63 | 55.39 | 186.58 | 135.43 | 77.75 | 0.00 | 153.38 | 65.47 | 51.55 | 81.61 | 192.26 | 402.30 | 279.22 | 124.92 | 39.14 |
| P12110 | Collagen alpha-2(VI) chain | **2.43** | **0.11** | 373.14 | 342.60 | 907.76 | 582.98 | 261.27 | 826.78 | 633.58 | 103.79 | 40.26 | 1009.27 | 1088.25 | 1695.46 | 454.25 | 1165.55 | 33.77 |
| P06899 | Histone H2B type 1-J | **1.52** | **0.11** | 3713.97 | 1982.80 | 5660.59 | 1624.11 | 2777.70 | 2672.56 | 5069.57 | 6447.17 | 1602.86 | 6170.72 | 6259.54 | 4186.15 | 3393.60 | 6030.25 | 7923.29 |
| P23527 | Histone H2B type 1-O | **1.52** | **0.11** | 3713.97 | 1982.80 | 5660.59 | 1624.11 | 2777.70 | 2672.56 | 5069.57 | 6447.17 | 1602.86 | 6170.72 | 6259.54 | 4186.15 | 3393.60 | 6030.25 | 7923.29 |
| P33778 | Histone H2B type 1-B | **1.52** | **0.11** | 3713.97 | 1982.80 | 5660.59 | 1624.11 | 2777.70 | 2672.56 | 5069.57 | 6447.17 | 1602.86 | 6170.72 | 6259.54 | 4186.15 | 3393.60 | 6030.25 | 7923.29 |
| Q16778 | Histone H2B type 2-E | **1.52** | **0.11** | 3713.97 | 1982.80 | 5660.59 | 1624.11 | 2777.70 | 2672.56 | 5069.57 | 6447.17 | 1602.86 | 6170.72 | 6259.54 | 4186.15 | 3393.60 | 6030.25 | 7923.29 |
| Q8N257 | Histone H2B type 3-B | **1.52** | **0.11** | 3713.97 | 1982.80 | 5660.59 | 1624.11 | 2777.70 | 2672.56 | 5069.57 | 6447.17 | 1602.86 | 6170.72 | 6259.54 | 4186.15 | 3393.60 | 6030.25 | 7923.29 |
| Q9UHC1 | DNA mismatch repair protein Mlh3 | **11.53** | **0.11** | 43.08 | 96.32 | 496.65 | 553.75 | 0.00 | 0.00 | 0.00 | 0.00 | 215.39 | 566.73 | 314.03 | 1561.29 | 343.32 | 194.51 | 0.00 |
| Q9H4A6 | Golgi phosphoprotein 3 | **6.29** | **0.11** | 51.50 | 109.89 | 323.83 | 333.44 | 9.57 | 0.00 | 0.00 | 247.93 | 0.00 | 198.44 | 21.36 | 847.96 | 597.73 | 262.15 | 15.36 |
| Q9BYE2 | Transmembrane protease serine 13 | **2.52** | **0.11** | 90.39 | 131.57 | 228.01 | 123.38 | 162.84 | 0.00 | 0.00 | 289.10 | 0.00 | 290.26 | 347.74 | 197.50 | 0.00 | 234.25 | 298.32 |
| P40121 | Macrophage-capping protein | **1.95** | **0.11** | 141.93 | 114.81 | 276.48 | 131.30 | 339.09 | 80.46 | 141.98 | 94.80 | 53.34 | 152.41 | 439.80 | 394.40 | 259.29 | 306.80 | 106.15 |
| P38159 | RNA-binding motif protein, X chromosome | **1.45** | **0.11** | 93.36 | 32.55 | 135.68 | 43.64 | 65.34 | 62.94 | 120.60 | 134.43 | 83.48 | 163.83 | 119.78 | 138.90 | 58.76 | 148.22 | 184.59 |
| Q9NUQ9 | Protein FAM49B | **4.34** | **0.11** | 6.26 | 14.00 | 27.15 | 22.67 | 0.00 | 0.00 | 0.00 | 31.30 | 0.00 | 0.00 | 42.76 | 55.31 | 31.31 | 33.52 | 0.00 |
| Q08945 | FACT complex subunit SSRP1 | **4.61** | **0.11** | 8.57 | 12.17 | 39.50 | 38.08 | 0.00 | 0.00 | 16.89 | 25.99 | 0.00 | 81.64 | 82.38 | 0.00 | 0.00 | 20.74 | 52.20 |
| P26006 | Integrin alpha-3 | **0.14** | **0.11** | 23.21 | 21.55 | 3.27 | 8.02 | 33.16 | 0.00 | 0.00 | 44.15 | 38.77 | 0.00 | 0.00 | 19.65 | 0.00 | 0.00 | 0.00 |
| Q9UMY4 | Sorting nexin-12 | **10.69** | **0.11** | 3.61 | 8.07 | 38.61 | 42.99 | 18.05 | 0.00 | 0.00 | 0.00 | 0.00 | 42.18 | 42.22 | 0.00 | 30.16 | 117.10 | 0.00 |
| P13761 | HLA class II histocompatibility antigen, DRB1-7 beta chain | **6.26** | **0.11** | 8.19 | 18.32 | 51.27 | 53.00 | 40.96 | 0.00 | 0.00 | 0.00 | 0.00 | 41.33 | 130.23 | 98.79 | 37.27 | 0.00 | 0.00 |
| Q17RS7 | Flap endonuclease GEN homolog 1 | **2.93** | **0.11** | 81.68 | 132.96 | 239.53 | 156.35 | 102.51 | 0.00 | 305.88 | 0.00 | 0.00 | 318.43 | 254.82 | 0.00 | 471.67 | 215.28 | 177.00 |
| P12111 | Collagen alpha-3(VI) chain | **2.18** | **0.11** | 464.42 | 385.13 | 1013.18 | 591.35 | 343.54 | 1008.62 | 708.64 | 137.23 | 124.07 | 1164.38 | 1105.13 | 1692.72 | 590.05 | 1453.17 | 73.63 |
| Q86VI3 | Ras GTPase-activating-like protein IQGAP3 | **2.82** | **0.11** | 33.81 | 31.07 | 95.37 | 71.61 | 0.00 | 0.00 | 59.95 | 50.62 | 58.48 | 165.11 | 192.42 | 84.22 | 52.61 | 0.00 | 77.86 |
| O43169 | Cytochrome b5 type B | **0.29** | **0.11** | 75.76 | 62.32 | 21.72 | 38.10 | 159.23 | 0.00 | 54.51 | 116.31 | 48.74 | 0.00 | 93.41 | 0.00 | 0.00 | 36.92 | 0.00 |
| P35580 | Myosin-10 | **1.86** | **0.11** | 314.99 | 90.05 | 586.09 | 329.23 | 393.81 | 330.01 | 398.72 | 266.33 | 186.07 | 348.44 | 584.70 | 1179.97 | 629.50 | 547.59 | 226.32 |
| P61204 | ADP-ribosylation factor 3 | **1.35** | **0.11** | 228.07 | 93.55 | 307.84 | 54.36 | 272.95 | 84.76 | 256.00 | 331.10 | 195.52 | 296.14 | 404.29 | 254.97 | 277.37 | 278.78 | 335.50 |
| P84077 | ADP-ribosylation factor 1 | **1.35** | **0.11** | 228.07 | 93.55 | 307.84 | 54.36 | 272.95 | 84.76 | 256.00 | 331.10 | 195.52 | 296.14 | 404.29 | 254.97 | 277.37 | 278.78 | 335.50 |
| O75306 | NADH dehydrogenase [ubiquinone] iron-sulfur protein 2, mitochondrial | **6.26** | **0.11** | 75.61 | 169.07 | 473.54 | 492.50 | 0.00 | 0.00 | 378.05 | 0.00 | 0.00 | 244.53 | 593.67 | 1266.01 | 0.00 | 737.04 | 0.00 |
| P25787 | Proteasome subunit alpha type-2 | **2.46** | **0.11** | 15.87 | 14.74 | 39.03 | 25.89 | 23.42 | 0.00 | 30.76 | 25.16 | 0.00 | 49.15 | 77.90 | 0.00 | 43.45 | 25.07 | 38.60 |
| Q96HY6 | DDRGK domain-containing protein 1 | **0.08** | **0.11** | 35.21 | 35.57 | 2.78 | 6.80 | 82.38 | 0.00 | 53.41 | 40.25 | 0.00 | 16.65 | 0.00 | 0.00 | 0.00 | 0.00 | 0.00 |
| Q9H4A5 | Golgi phosphoprotein 3-like | **4.96** | **0.11** | 1.91 | 4.28 | 9.50 | 9.16 | 9.57 | 0.00 | 0.00 | 0.00 | 0.00 | 4.52 | 21.36 | 0.00 | 0.00 | 15.75 | 15.36 |
| Q9Y2Q0 | Probable phospholipid-transporting ATPase IA | **3.38** | **0.11** | 64.07 | 87.75 | 216.35 | 174.55 | 0.00 | 0.00 | 158.01 | 162.34 | 0.00 | 368.02 | 330.78 | 0.00 | 233.49 | 0.00 | 365.78 |
| Q9Y6K5 | 2'-5'-oligoadenylate synthase 3 | **2.70** | **0.11** | 194.50 | 273.58 | 524.70 | 336.20 | 0.00 | 574.74 | 397.73 | 0.00 | 0.00 | 779.29 | 671.46 | 583.34 | 135.63 | 890.14 | 88.31 |
| P04439 | HLA class I histocompatibility antigen, A-3 alpha chain | **2.08** | **0.11** | 145.23 | 87.25 | 302.41 | 190.51 | 250.38 | 208.93 | 148.22 | 76.11 | 42.51 | 67.12 | 471.45 | 474.48 | 354.54 | 385.81 | 61.07 |
| P13746 | HLA class I histocompatibility antigen, A-11 alpha chain | **2.08** | **0.11** | 145.23 | 87.25 | 302.41 | 190.51 | 250.38 | 208.93 | 148.22 | 76.11 | 42.51 | 67.12 | 471.45 | 474.48 | 354.54 | 385.81 | 61.07 |
| P30443 | HLA class I histocompatibility antigen, A-1 alpha chain | **2.08** | **0.11** | 145.23 | 87.25 | 302.41 | 190.51 | 250.38 | 208.93 | 148.22 | 76.11 | 42.51 | 67.12 | 471.45 | 474.48 | 354.54 | 385.81 | 61.07 |
| P30455 | HLA class I histocompatibility antigen, A-36 alpha chain | **2.08** | **0.11** | 145.23 | 87.25 | 302.41 | 190.51 | 250.38 | 208.93 | 148.22 | 76.11 | 42.51 | 67.12 | 471.45 | 474.48 | 354.54 | 385.81 | 61.07 |
| Q07020 | 60S ribosomal protein L18 | **1.62** | **0.11** | 146.67 | 102.08 | 238.08 | 70.29 | 97.32 | 46.31 | 216.08 | 289.61 | 84.03 | 188.20 | 225.66 | 234.47 | 167.00 | 244.88 | 368.27 |
| Q562R1 | Beta-actin-like protein 2 | **1.42** | **0.11** | 6615.77 | 2817.01 | 9417.03 | 2481.26 | 7031.54 | 4692.02 | 8371.22 | 10010.27 | 2973.77 | 5598.94 | 10914.45 | 12585.48 | 10501.17 | 9115.38 | 7786.77 |
| P04843 | Dolichyl-diphosphooligosaccharide--protein glycosyltransferase subunit 1 | **1.50** | **0.11** | 68.82 | 43.01 | 103.37 | 20.84 | 71.71 | 0.00 | 93.34 | 113.98 | 65.06 | 92.80 | 121.83 | 72.45 | 91.37 | 119.08 | 122.68 |
| P42330 | Aldo-keto reductase family 1 member C3 | **0.29** | **0.11** | 201.20 | 191.56 | 58.09 | 59.03 | 542.15 | 142.62 | 92.23 | 104.99 | 123.98 | 50.94 | 120.21 | 141.32 | 16.92 | 0.00 | 19.18 |
| O60437 | Periplakin | **15.01** | **0.12** | 15.19 | 15.22 | 228.06 | 273.75 | 33.30 | 0.00 | 15.75 | 0.00 | 26.91 | 79.20 | 96.13 | 711.13 | 401.34 | 15.50 | 65.06 |
| Q9UPN9 | E3 ubiquitin-protein ligase TRIM33 | **3.54** | **0.12** | 115.69 | 140.97 | 409.77 | 366.97 | 87.15 | 340.85 | 0.00 | 150.48 | 0.00 | 589.20 | 0.00 | 891.78 | 707.71 | 158.31 | 111.64 |
| Q96LP6 | Uncharacterized protein C12orf42 | **1.66** | **0.12** | 242.70 | 136.14 | 404.07 | 164.66 | 313.41 | 0.00 | 284.35 | 307.02 | 308.69 | 279.46 | 662.15 | 198.78 | 371.45 | 407.44 | 505.13 |
| P02749 | Beta-2-glycoprotein 1 | **0.35** | **0.12** | 62.67 | 46.07 | 21.66 | 31.75 | 73.62 | 0.00 | 89.57 | 33.79 | 116.37 | 19.58 | 0.00 | 28.92 | 0.00 | 81.45 | 0.00 |
| Q9NRW1 | Ras-related protein Rab-6B | **1.47** | **0.12** | 307.30 | 129.31 | 451.86 | 143.46 | 344.40 | 275.99 | 220.21 | 511.72 | 184.18 | 351.21 | 608.97 | 403.64 | 347.52 | 341.37 | 658.42 |
| P63092 | Guanine nucleotide-binding protein G(s) subunit alpha isoforms short | **2.68** | **0.12** | 257.20 | 286.01 | 688.66 | 489.12 | 0.00 | 0.00 | 493.62 | 620.21 | 172.17 | 631.40 | 216.02 | 691.13 | 1627.62 | 443.02 | 522.79 |
| Q5JWF2 | Guanine nucleotide-binding protein G(s) subunit alpha isoforms XLas | **2.68** | **0.12** | 257.20 | 286.01 | 688.66 | 489.12 | 0.00 | 0.00 | 493.62 | 620.21 | 172.17 | 631.40 | 216.02 | 691.13 | 1627.62 | 443.02 | 522.79 |
| P21926 | CD9 antigen | **0.36** | **0.12** | 176.00 | 124.57 | 63.25 | 91.12 | 296.62 | 134.11 | 295.56 | 153.73 | 0.00 | 25.25 | 0.00 | 144.45 | 0.00 | 0.00 | 209.78 |
| Q58FF7 | Putative heat shock protein HSP 90-beta-3 | **1.68** | **0.12** | 421.45 | 139.49 | 710.03 | 347.58 | 455.24 | 191.31 | 426.21 | 568.94 | 465.55 | 604.97 | 775.48 | 495.69 | 300.30 | 1319.96 | 763.79 |
| P60866 | 40S ribosomal protein S20 | **1.42** | **0.12** | 165.89 | 79.48 | 235.87 | 54.14 | 137.93 | 81.00 | 260.44 | 238.97 | 111.10 | 254.94 | 305.17 | 194.26 | 167.15 | 209.44 | 284.24 |
| P59998 | Actin-related protein 2/3 complex subunit 4 | **1.27** | **0.12** | 108.85 | 33.55 | 138.29 | 22.94 | 139.71 | 78.99 | 124.17 | 134.54 | 66.84 | 118.53 | 168.78 | 152.46 | 132.01 | 150.16 | 107.80 |
| Q9NR45 | Sialic acid synthase | **4.12** | **0.12** | 5.98 | 13.37 | 24.62 | 21.88 | 0.00 | 0.00 | 29.89 | 0.00 | 0.00 | 0.00 | 45.26 | 0.00 | 16.20 | 42.62 | 43.63 |
| O43736 | Integral membrane protein 2A | **2.22** | **0.12** | 204.07 | 192.53 | 453.34 | 272.04 | 262.78 | 0.00 | 363.10 | 394.45 | 0.00 | 115.42 | 415.61 | 232.09 | 886.87 | 580.64 | 489.43 |
| Q96PD6 | 2-acylglycerol O-acyltransferase 1 | **2.22** | **0.12** | 204.07 | 192.53 | 453.34 | 272.04 | 262.78 | 0.00 | 363.10 | 394.45 | 0.00 | 115.42 | 415.61 | 232.09 | 886.87 | 580.64 | 489.43 |
| Q99715 | Collagen alpha-1(XII) chain | **3.36** | **0.12** | 76.17 | 111.69 | 255.72 | 209.20 | 37.97 | 273.14 | 49.57 | 20.19 | 0.00 | 58.13 | 198.89 | 358.21 | 365.77 | 553.30 | 0.00 |
| P15121 | Aldose reductase | **1.96** | **0.12** | 74.03 | 84.47 | 145.14 | 52.75 | 48.95 | 0.00 | 58.30 | 219.73 | 43.16 | 236.65 | 152.97 | 126.93 | 81.45 | 114.77 | 158.09 |
| P50454 | Serpin H1 | **2.71** | **0.12** | 104.14 | 72.07 | 282.02 | 229.33 | 56.93 | 62.33 | 194.80 | 168.81 | 37.82 | 82.35 | 109.57 | 445.05 | 578.49 | 432.07 | 44.61 |
| P02042 | Hemoglobin subunit delta | **0.51** | **0.12** | 16782.98 | 10172.96 | 8641.45 | 5285.08 | 14261.27 | 15100.33 | 13173.05 | 7251.32 | 34128.94 | 7735.36 | 7238.61 | 2787.85 | 18571.15 | 8863.77 | 6651.94 |
| Q5T013 | Putative hydroxypyruvate isomerase | **1.92** | **0.12** | 118.21 | 117.19 | 227.53 | 95.54 | 138.03 | 0.00 | 266.10 | 186.92 | 0.00 | 88.12 | 151.89 | 206.98 | 332.32 | 309.13 | 276.72 |
| P29966 | Myristoylated alanine-rich C-kinase substrate | **0.72** | **0.12** | 379.55 | 108.74 | 272.60 | 99.22 | 448.19 | 392.23 | 297.50 | 246.33 | 513.51 | 364.49 | 400.40 | 201.48 | 305.08 | 150.86 | 213.29 |
| P15374 | Ubiquitin carboxyl-terminal hydrolase isozyme L3 | **0.23** | **0.12** | 19.12 | 17.73 | 4.40 | 10.78 | 36.78 | 0.00 | 30.57 | 28.25 | 0.00 | 0.00 | 0.00 | 0.00 | 0.00 | 0.00 | 26.40 |
| P62277 | 40S ribosomal protein S13 | **1.73** | **0.12** | 85.18 | 61.62 | 147.29 | 59.01 | 57.25 | 0.00 | 144.61 | 145.03 | 78.99 | 139.11 | 206.19 | 110.72 | 68.91 | 132.82 | 225.95 |
| P05783 | Keratin, type I cytoskeletal 18 | **0.64** | **0.12** | 795.90 | 278.04 | 507.79 | 280.78 | 1073.78 | 796.20 | 391.36 | 1032.82 | 685.35 | 637.03 | 477.86 | 923.41 | 169.04 | 611.47 | 227.90 |
| P00918 | Carbonic anhydrase 2 | **0.26** | **0.12** | 143.59 | 121.52 | 37.62 | 20.57 | 153.44 | 59.51 | 91.72 | 63.02 | 350.27 | 49.51 | 39.75 | 0.00 | 47.88 | 57.70 | 30.87 |
| P63267 | Actin, gamma-enteric smooth muscle | **1.53** | **0.12** | 6423.86 | 2266.12 | 9834.24 | 3961.55 | 7984.41 | 4120.93 | 7286.26 | 8818.59 | 3909.11 | 6129.08 | 7661.17 | 16740.06 | 12231.47 | 8720.61 | 7523.04 |
| Q13228 | Selenium-binding protein 1 | **0.18** | **0.13** | 22.94 | 21.24 | 4.21 | 10.32 | 34.75 | 36.00 | 0.00 | 0.00 | 43.97 | 0.00 | 0.00 | 0.00 | 25.27 | 0.00 | 0.00 |
| Q9H853 | Putative tubulin-like protein alpha-4B | **1.52** | **0.13** | 410.54 | 274.77 | 622.02 | 127.23 | 376.19 | 0.00 | 764.79 | 419.37 | 492.38 | 422.44 | 689.86 | 766.80 | 519.07 | 688.21 | 645.76 |
| A2A3N6 | Putative PIP5K1A and PSMD4-like protein | **0.42** | **0.13** | 111.14 | 71.22 | 46.88 | 54.83 | 192.24 | 0.00 | 107.45 | 147.72 | 108.29 | 112.83 | 109.75 | 0.00 | 0.00 | 0.00 | 58.70 |
| P30505 | HLA class I histocompatibility antigen, Cw-8 alpha chain | **2.32** | **0.13** | 108.12 | 78.10 | 251.33 | 182.03 | 100.55 | 238.14 | 103.79 | 63.86 | 34.24 | 56.79 | 349.53 | 516.83 | 332.70 | 195.66 | 56.46 |
| P50395 | Rab GDP dissociation inhibitor beta | **1.40** | **0.13** | 134.45 | 60.95 | 187.65 | 43.41 | 148.70 | 50.69 | 158.10 | 212.29 | 102.47 | 152.74 | 199.44 | 146.24 | 151.67 | 236.34 | 239.45 |
| P84085 | ADP-ribosylation factor 5 | **1.34** | **0.13** | 224.23 | 88.39 | 300.32 | 60.75 | 265.56 | 112.81 | 256.00 | 331.10 | 155.67 | 284.45 | 404.29 | 226.31 | 277.37 | 278.78 | 330.69 |
| P30040 | Endoplasmic reticulum resident protein 29 | **0.54** | **0.13** | 122.96 | 54.84 | 65.79 | 56.47 | 167.97 | 124.67 | 64.17 | 72.12 | 185.89 | 70.79 | 49.62 | 37.24 | 0.00 | 68.81 | 168.28 |
| P09467 | Fructose-1,6-bisphosphatase 1 | **0.13** | **0.13** | 183.13 | 184.78 | 23.41 | 18.59 | 216.34 | 71.78 | 490.30 | 103.95 | 33.29 | 38.88 | 34.14 | 0.00 | 27.97 | 39.44 | 0.00 |
| P31937 | 3-hydroxyisobutyrate dehydrogenase, mitochondrial | **0.17** | **0.13** | 7.46 | 7.05 | 1.29 | 3.17 | 15.35 | 0.00 | 10.61 | 11.35 | 0.00 | 0.00 | 7.75 | 0.00 | 0.00 | 0.00 | 0.00 |
| Q7Z406 | Myosin-14 | **1.86** | **0.13** | 322.28 | 157.70 | 599.58 | 335.56 | 372.33 | 252.41 | 564.24 | 277.85 | 144.59 | 294.72 | 565.25 | 1181.44 | 706.53 | 594.74 | 254.83 |
| Q14195 | Dihydropyrimidinase-related protein 3 | **8.61** | **0.13** | 14.13 | 21.66 | 121.66 | 143.40 | 0.00 | 0.00 | 49.10 | 0.00 | 21.55 | 0.00 | 58.10 | 310.04 | 296.40 | 65.42 | 0.00 |
| Q8TDQ7 | Glucosamine-6-phosphate isomerase 2 | **0.20** | **0.13** | 24.26 | 25.28 | 4.87 | 11.92 | 39.10 | 0.00 | 23.89 | 58.29 | 0.00 | 0.00 | 0.00 | 0.00 | 0.00 | 29.21 | 0.00 |
| P62263 | 40S ribosomal protein S14 | **1.54** | **0.13** | 118.74 | 69.76 | 183.37 | 57.86 | 118.25 | 0.00 | 140.36 | 159.36 | 175.73 | 254.38 | 190.72 | 153.44 | 112.09 | 142.79 | 246.78 |
| Q01581 | Hydroxymethylglutaryl-CoA synthase, cytoplasmic | **0.18** | **0.13** | 167.11 | 184.92 | 29.89 | 73.22 | 478.06 | 100.08 | 0.00 | 177.27 | 80.15 | 179.36 | 0.00 | 0.00 | 0.00 | 0.00 | 0.00 |
| O95782 | AP-2 complex subunit alpha-1 | **1.81** | **0.13** | 14.67 | 9.22 | 26.59 | 13.44 | 11.32 | 0.00 | 20.23 | 22.51 | 19.30 | 4.79 | 27.38 | 26.85 | 46.05 | 32.11 | 22.34 |
| P00387 | NADH-cytochrome b5 reductase 3 | **1.62** | **0.13** | 33.58 | 25.56 | 54.24 | 14.99 | 43.08 | 0.00 | 54.98 | 56.51 | 13.31 | 54.43 | 38.14 | 48.30 | 59.52 | 80.75 | 44.31 |
| Q96N66 | Lysophospholipid acyltransferase 7 | **0.44** | **0.13** | 65.59 | 39.55 | 29.00 | 32.90 | 68.32 | 0.00 | 86.06 | 104.55 | 69.03 | 73.54 | 51.62 | 0.00 | 0.00 | 48.81 | 0.00 |
| Q16698 | 2,4-dienoyl-CoA reductase, mitochondrial | **0.63** | **0.13** | 128.77 | 57.10 | 81.09 | 36.99 | 152.60 | 33.54 | 130.09 | 185.56 | 142.06 | 94.89 | 147.06 | 81.36 | 64.56 | 47.30 | 51.39 |
| P54868 | Hydroxymethylglutaryl-CoA synthase, mitochondrial | **0.21** | **0.13** | 198.89 | 211.66 | 42.41 | 83.15 | 563.34 | 103.23 | 48.30 | 201.30 | 78.28 | 207.81 | 46.66 | 0.00 | 0.00 | 0.00 | 0.00 |
| Q96M42 | Putative uncharacterized protein encoded by LINC00479 | **2.48** | **0.13** | 3769.37 | 5492.32 | 9341.65 | 5516.88 | 0.00 | 6768.11 | 0.00 | 12078.73 | 0.00 | 13967.84 | 10099.52 | 10999.74 | 6158.72 | 0.00 | 14824.07 |
| Q01469 | Fatty acid-binding protein, epidermal | **0.22** | **0.13** | 435.50 | 395.66 | 97.71 | 33.72 | 1047.27 | 251.31 | 610.96 | 185.82 | 82.14 | 105.61 | 119.92 | 78.60 | 44.49 | 141.64 | 95.96 |
| Q15436 | Protein transport protein Sec23A | **3.24** | **0.13** | 5.42 | 9.13 | 17.57 | 13.92 | 6.02 | 0.00 | 0.00 | 21.08 | 0.00 | 4.73 | 34.34 | 0.00 | 31.46 | 14.23 | 20.65 |
| P19827 | Inter-alpha-trypsin inhibitor heavy chain H1 | **3.21** | **0.13** | 30.01 | 22.15 | 96.31 | 88.59 | 38.44 | 59.63 | 0.00 | 19.62 | 32.34 | 61.63 | 35.02 | 230.57 | 73.86 | 176.80 | 0.00 |
| P62857 | 40S ribosomal protein S28 | **0.53** | **0.13** | 109.84 | 25.93 | 58.38 | 67.26 | 121.20 | 106.32 | 121.52 | 133.39 | 66.74 | 78.74 | 0.00 | 0.00 | 0.00 | 134.54 | 137.01 |
| Q96AG4 | Leucine-rich repeat-containing protein 59 | **1.87** | **0.13** | 33.40 | 23.23 | 62.43 | 32.69 | 54.43 | 0.00 | 36.70 | 54.50 | 21.38 | 63.72 | 94.27 | 0.00 | 62.50 | 78.44 | 75.65 |
| Q9UHP9 | Small muscular protein | **2.62** | **0.13** | 355.63 | 628.50 | 932.19 | 526.65 | 0.00 | 0.00 | 327.14 | 1450.99 | 0.00 | 1291.14 | 0.00 | 1513.82 | 1014.35 | 1028.10 | 745.75 |
| Q14151 | Scaffold attachment factor B2 | **2.10** | **0.13** | 16.60 | 16.09 | 34.86 | 19.76 | 0.00 | 23.97 | 22.54 | 36.47 | 0.00 | 54.05 | 51.00 | 27.73 | 0.00 | 34.02 | 42.38 |
| Q00059 | Transcription factor A, mitochondrial | **0.36** | **0.13** | 31.00 | 22.31 | 11.22 | 17.39 | 61.44 | 0.00 | 23.76 | 38.14 | 31.64 | 32.59 | 0.00 | 0.00 | 0.00 | 0.00 | 34.72 |
| P05534 | HLA class I histocompatibility antigen, A-24 alpha chain | **2.06** | **0.13** | 145.23 | 87.25 | 298.45 | 189.45 | 250.38 | 208.93 | 148.22 | 76.11 | 42.51 | 67.12 | 471.45 | 474.48 | 330.77 | 385.81 | 61.07 |
| Q14847 | LIM and SH3 domain protein 1 | **0.41** | **0.13** | 112.92 | 73.43 | 46.63 | 59.44 | 224.11 | 136.41 | 53.18 | 41.71 | 109.21 | 66.69 | 0.00 | 0.00 | 37.33 | 156.53 | 19.22 |
| Q9UQ80 | Proliferation-associated protein 2G4 | **1.94** | **0.13** | 83.02 | 55.78 | 161.37 | 92.51 | 83.76 | 0.00 | 143.88 | 122.19 | 65.28 | 229.49 | 126.20 | 106.63 | 64.34 | 127.41 | 314.18 |
| P14406 | Cytochrome c oxidase subunit 7A2, mitochondrial | **0.61** | **0.13** | 97.80 | 40.27 | 59.90 | 35.98 | 119.11 | 60.12 | 137.20 | 123.65 | 48.90 | 75.47 | 100.46 | 63.81 | 37.68 | 81.98 | 0.00 |
| Q15424 | Scaffold attachment factor B1 | **2.09** | **0.13** | 16.67 | 16.12 | 34.86 | 19.76 | 0.00 | 23.97 | 22.90 | 36.47 | 0.00 | 54.05 | 51.00 | 27.73 | 0.00 | 34.02 | 42.38 |
| P62736 | Actin, aortic smooth muscle | **1.45** | **0.13** | 7426.53 | 3019.70 | 10793.92 | 3645.11 | 9363.88 | 4514.94 | 8727.94 | 10616.80 | 3909.11 | 6882.28 | 9116.25 | 16740.06 | 13228.35 | 10651.28 | 8145.30 |
| P68032 | Actin, alpha cardiac muscle 1 | **1.45** | **0.13** | 7426.53 | 3019.70 | 10793.92 | 3645.11 | 9363.88 | 4514.94 | 8727.94 | 10616.80 | 3909.11 | 6882.28 | 9116.25 | 16740.06 | 13228.35 | 10651.28 | 8145.30 |
| P68133 | Actin, alpha skeletal muscle | **1.45** | **0.13** | 7426.53 | 3019.70 | 10793.92 | 3645.11 | 9363.88 | 4514.94 | 8727.94 | 10616.80 | 3909.11 | 6882.28 | 9116.25 | 16740.06 | 13228.35 | 10651.28 | 8145.30 |
| Q63HK3 | Zinc finger protein with KRAB and SCAN domains 2 | **2.32** | **0.14** | 225.16 | 240.56 | 521.88 | 338.09 | 160.79 | 606.35 | 295.79 | 62.87 | 0.00 | 649.73 | 462.36 | 992.54 | 343.62 | 683.01 | 0.00 |
| P12882 | Myosin-1 | **3.67** | **0.14** | 76.47 | 108.98 | 280.76 | 258.72 | 0.00 | 0.00 | 233.91 | 148.42 | 0.00 | 0.00 | 335.91 | 741.09 | 324.54 | 151.75 | 131.25 |
| P13535 | Myosin-8 | **3.67** | **0.14** | 76.47 | 108.98 | 280.76 | 258.72 | 0.00 | 0.00 | 233.91 | 148.42 | 0.00 | 0.00 | 335.91 | 741.09 | 324.54 | 151.75 | 131.25 |
| Q9Y623 | Myosin-4 | **3.67** | **0.14** | 76.47 | 108.98 | 280.76 | 258.72 | 0.00 | 0.00 | 233.91 | 148.42 | 0.00 | 0.00 | 335.91 | 741.09 | 324.54 | 151.75 | 131.25 |
| P13797 | Plastin-3 | **1.88** | **0.14** | 104.62 | 47.30 | 196.21 | 116.52 | 138.09 | 148.33 | 70.17 | 126.96 | 39.56 | 111.91 | 311.36 | 355.43 | 185.87 | 160.71 | 51.97 |
| P05164 | Myeloperoxidase | **23.69** | **0.14** | 10.87 | 15.81 | 257.60 | 341.14 | 34.71 | 0.00 | 0.00 | 19.66 | 0.00 | 20.98 | 782.29 | 597.01 | 90.26 | 55.04 | 0.00 |
| Q07000 | HLA class I histocompatibility antigen, Cw-15 alpha chain | **2.34** | **0.14** | 107.36 | 79.01 | 251.33 | 182.03 | 100.55 | 238.14 | 103.79 | 63.86 | 30.46 | 56.79 | 349.53 | 516.83 | 332.70 | 195.66 | 56.46 |
| Q29963 | HLA class I histocompatibility antigen, Cw-6 alpha chain | **2.34** | **0.14** | 107.36 | 79.01 | 251.33 | 182.03 | 100.55 | 238.14 | 103.79 | 63.86 | 30.46 | 56.79 | 349.53 | 516.83 | 332.70 | 195.66 | 56.46 |
| P02788 | Lactotransferrin | **84.30** | **0.14** | 2.72 | 6.08 | 229.31 | 314.79 | 13.60 | 0.00 | 0.00 | 0.00 | 0.00 | 0.00 | 594.61 | 671.74 | 63.65 | 45.87 | 0.00 |
| P04229 | HLA class II histocompatibility antigen, DRB1-1 beta chain | **7.83** | **0.14** | 8.19 | 18.32 | 64.11 | 74.33 | 40.96 | 0.00 | 0.00 | 0.00 | 0.00 | 41.33 | 196.25 | 98.50 | 48.59 | 0.00 | 0.00 |
| Q29974 | HLA class II histocompatibility antigen, DRB1-16 beta chain | **7.83** | **0.14** | 8.19 | 18.32 | 64.11 | 74.33 | 40.96 | 0.00 | 0.00 | 0.00 | 0.00 | 41.33 | 196.25 | 98.50 | 48.59 | 0.00 | 0.00 |
| Q30154 | HLA class II histocompatibility antigen, DR beta 5 chain | **7.83** | **0.14** | 8.19 | 18.32 | 64.11 | 74.33 | 40.96 | 0.00 | 0.00 | 0.00 | 0.00 | 41.33 | 196.25 | 98.50 | 48.59 | 0.00 | 0.00 |
| Q9UBQ0 | Vacuolar protein sorting-associated protein 29 | **0.18** | **0.14** | 23.97 | 23.32 | 4.31 | 10.56 | 28.33 | 0.00 | 51.08 | 40.46 | 0.00 | 0.00 | 0.00 | 0.00 | 0.00 | 0.00 | 25.86 |
| P05109 | Protein S100-A8 | **12.20** | **0.14** | 80.66 | 75.35 | 983.83 | 1254.29 | 142.40 | 177.07 | 48.79 | 0.00 | 35.03 | 123.06 | 3219.39 | 609.57 | 160.54 | 90.83 | 1699.55 |
| Q15075 | Early endosome antigen 1 | **2.14** | **0.14** | 417.53 | 584.31 | 891.92 | 380.57 | 271.39 | 169.49 | 170.02 | 1450.99 | 25.75 | 444.44 | 605.05 | 1513.82 | 1014.35 | 1028.10 | 745.75 |
| P55263 | Adenosine kinase | **0.16** | **0.14** | 18.45 | 18.55 | 2.96 | 7.25 | 31.32 | 0.00 | 19.46 | 41.45 | 0.00 | 0.00 | 17.75 | 0.00 | 0.00 | 0.00 | 0.00 |
| Q9BXB1 | Leucine-rich repeat-containing G-protein coupled receptor 4 | **1.91** | **0.14** | 170.37 | 118.32 | 325.08 | 182.63 | 131.60 | 0.00 | 300.78 | 261.54 | 157.95 | 291.92 | 326.25 | 408.95 | 550.13 | 373.21 | 0.00 |
| Q9C005 | Protein dpy-30 homolog | **0.39** | **0.14** | 16.80 | 13.42 | 6.53 | 7.23 | 37.58 | 0.00 | 14.86 | 15.25 | 16.33 | 0.00 | 11.17 | 0.00 | 0.00 | 13.90 | 14.12 |
| Q9TNN7 | HLA class I histocompatibility antigen, Cw-5 alpha chain | **2.33** | **0.14** | 105.03 | 73.03 | 244.94 | 179.25 | 100.55 | 225.81 | 100.69 | 63.86 | 34.24 | 56.79 | 349.53 | 516.83 | 294.38 | 195.66 | 56.46 |
| P01893 | Putative HLA class I histocompatibility antigen, alpha chain H | **2.10** | **0.14** | 113.96 | 61.67 | 239.64 | 169.89 | 136.76 | 208.93 | 96.86 | 76.11 | 51.12 | 67.12 | 349.39 | 474.48 | 330.77 | 154.98 | 61.07 |
| P17931 | Galectin-3 | **0.36** | **0.14** | 273.26 | 244.10 | 99.39 | 92.55 | 628.21 | 380.00 | 257.66 | 74.74 | 25.66 | 146.68 | 162.52 | 0.00 | 0.00 | 63.12 | 224.02 |
| P02751 | Fibronectin | **7.32** | **0.14** | 114.27 | 119.66 | 836.96 | 1008.44 | 65.07 | 73.87 | 326.34 | 33.72 | 72.37 | 226.25 | 325.83 | 2734.19 | 1164.30 | 546.23 | 24.97 |
| Q7Z3Y8 | Keratin, type I cytoskeletal 27 | **3.93** | **0.14** | 51.44 | 28.55 | 202.08 | 209.88 | 32.71 | 94.22 | 25.07 | 38.80 | 66.42 | 77.69 | 62.05 | 594.36 | 84.06 | 104.11 | 290.21 |
| Q7Z3Z0 | Keratin, type I cytoskeletal 25 | **3.93** | **0.14** | 51.44 | 28.55 | 202.08 | 209.88 | 32.71 | 94.22 | 25.07 | 38.80 | 66.42 | 77.69 | 62.05 | 594.36 | 84.06 | 104.11 | 290.21 |
| Q9Y6B6 | GTP-binding protein SAR1b | **1.62** | **0.14** | 78.10 | 62.13 | 126.25 | 35.46 | 63.66 | 49.24 | 61.01 | 186.68 | 29.93 | 82.45 | 86.97 | 169.96 | 126.53 | 137.14 | 154.47 |
| Q03591 | Complement factor H-related protein 1 | **8.23** | **0.14** | 5.38 | 12.03 | 44.29 | 52.26 | 0.00 | 0.00 | 26.91 | 0.00 | 0.00 | 61.91 | 28.00 | 139.64 | 0.00 | 36.17 | 0.00 |
| O43143 | Putative pre-mRNA-splicing factor ATP-dependent RNA helicase DHX15 | **1.99** | **0.14** | 17.76 | 19.61 | 35.33 | 16.51 | 24.88 | 0.00 | 47.03 | 0.00 | 16.92 | 25.08 | 44.80 | 18.77 | 21.12 | 41.23 | 60.96 |
| P08603 | Complement factor H | **2.53** | **0.14** | 17.63 | 11.19 | 44.54 | 35.55 | 18.40 | 0.00 | 30.63 | 17.06 | 22.08 | 47.55 | 28.00 | 107.28 | 49.51 | 34.91 | 0.00 |
| P29401 | Transketolase | **1.44** | **0.14** | 186.90 | 57.41 | 269.57 | 101.34 | 164.99 | 129.91 | 238.43 | 257.28 | 143.90 | 142.41 | 360.09 | 397.87 | 207.10 | 199.30 | 310.67 |
| Q6ZRH9 | Uncharacterized protein FLJ46347 | **0.38** | **0.14** | 39421.18 | 29445.27 | 15125.30 | 11142.51 | 25977.60 | 67226.96 | 16042.72 | 12888.95 | 74969.65 | 28911.08 | 11493.04 | 11407.70 | 27752.17 | 0.00 | 11187.80 |
| Q93052 | Lipoma-preferred partner | **0.15** | **0.14** | 46.54 | 47.80 | 7.21 | 17.66 | 112.58 | 66.33 | 53.80 | 0.00 | 0.00 | 0.00 | 0.00 | 0.00 | 0.00 | 43.25 | 0.00 |
| P62266 | 40S ribosomal protein S23 | **1.70** | **0.14** | 49.00 | 30.48 | 83.27 | 38.89 | 64.57 | 0.00 | 66.63 | 74.76 | 39.05 | 83.34 | 58.15 | 111.12 | 20.70 | 98.10 | 128.21 |
| O14818 | Proteasome subunit alpha type-7 | **1.50** | **0.14** | 46.91 | 29.63 | 70.16 | 18.27 | 50.54 | 0.00 | 78.93 | 62.79 | 42.31 | 48.68 | 88.58 | 74.24 | 49.54 | 69.16 | 90.78 |
| Q13418 | Integrin-linked protein kinase | **4.09** | **0.15** | 5.73 | 12.81 | 23.41 | 21.68 | 0.00 | 0.00 | 28.65 | 0.00 | 0.00 | 0.00 | 21.87 | 56.58 | 35.20 | 26.81 | 0.00 |
| Q96CM8 | Acyl-CoA synthetase family member 2, mitochondrial | **0.16** | **0.15** | 28.64 | 29.57 | 4.51 | 11.04 | 58.02 | 0.00 | 59.99 | 25.20 | 0.00 | 0.00 | 0.00 | 0.00 | 0.00 | 27.04 | 0.00 |
| P14550 | Alcohol dehydrogenase [NADP(+)] | **2.12** | **0.15** | 108.58 | 92.19 | 230.66 | 148.62 | 132.14 | 0.00 | 162.62 | 219.60 | 28.53 | 115.42 | 235.89 | 40.65 | 474.31 | 264.92 | 252.76 |
| O75131 | Copine-3 | **1.86** | **0.15** | 31.36 | 18.01 | 58.28 | 33.85 | 46.29 | 0.00 | 36.86 | 36.38 | 37.27 | 40.06 | 123.10 | 39.29 | 29.71 | 55.85 | 61.66 |
| Q9HAW9 | UDP-glucuronosyltransferase 1-8 | **0.01** | **0.15** | 110.42 | 135.52 | 1.31 | 3.22 | 65.85 | 0.00 | 24.66 | 337.92 | 123.70 | 0.00 | 0.00 | 0.00 | 0.00 | 7.88 | 0.00 |
| P04179 | Superoxide dismutase [Mn], mitochondrial | **3.49** | **0.15** | 69.84 | 39.26 | 243.97 | 246.73 | 115.10 | 108.50 | 52.27 | 44.15 | 29.18 | 54.16 | 161.53 | 473.95 | 619.66 | 154.54 | 0.00 |
| Q05682 | Caldesmon | **2.03** | **0.15** | 86.34 | 83.48 | 175.45 | 99.62 | 186.61 | 134.57 | 110.55 | 0.00 | 0.00 | 118.71 | 126.69 | 227.39 | 297.87 | 250.49 | 31.54 |
| Q9BRX8 | Redox-regulatory protein FAM213A | **0.40** | **0.15** | 45.27 | 36.11 | 18.06 | 20.05 | 21.67 | 0.00 | 71.63 | 44.04 | 89.00 | 41.61 | 35.22 | 0.00 | 0.00 | 31.56 | 0.00 |
| P48444 | Coatomer subunit delta | **1.88** | **0.15** | 21.84 | 26.71 | 41.14 | 12.66 | 25.39 | 0.00 | 18.65 | 65.16 | 0.00 | 24.27 | 49.55 | 60.37 | 33.26 | 37.43 | 41.97 |
| O60656 | UDP-glucuronosyltransferase 1-9 | **0.01** | **0.15** | 110.08 | 135.80 | 1.31 | 3.22 | 65.85 | 0.00 | 22.94 | 337.92 | 123.70 | 0.00 | 0.00 | 0.00 | 0.00 | 7.88 | 0.00 |
| P19224 | UDP-glucuronosyltransferase 1-6 | **0.01** | **0.15** | 110.08 | 135.80 | 1.31 | 3.22 | 65.85 | 0.00 | 22.94 | 337.92 | 123.70 | 0.00 | 0.00 | 0.00 | 0.00 | 7.88 | 0.00 |
| P22309 | UDP-glucuronosyltransferase 1-1 | **0.01** | **0.15** | 110.08 | 135.80 | 1.31 | 3.22 | 65.85 | 0.00 | 22.94 | 337.92 | 123.70 | 0.00 | 0.00 | 0.00 | 0.00 | 7.88 | 0.00 |
| P22310 | UDP-glucuronosyltransferase 1-4 | **0.01** | **0.15** | 110.08 | 135.80 | 1.31 | 3.22 | 65.85 | 0.00 | 22.94 | 337.92 | 123.70 | 0.00 | 0.00 | 0.00 | 0.00 | 7.88 | 0.00 |
| P35503 | UDP-glucuronosyltransferase 1-3 | **0.01** | **0.15** | 110.08 | 135.80 | 1.31 | 3.22 | 65.85 | 0.00 | 22.94 | 337.92 | 123.70 | 0.00 | 0.00 | 0.00 | 0.00 | 7.88 | 0.00 |
| P35504 | UDP-glucuronosyltransferase 1-5 | **0.01** | **0.15** | 110.08 | 135.80 | 1.31 | 3.22 | 65.85 | 0.00 | 22.94 | 337.92 | 123.70 | 0.00 | 0.00 | 0.00 | 0.00 | 7.88 | 0.00 |
| Q9HAW7 | UDP-glucuronosyltransferase 1-7 | **0.01** | **0.15** | 110.08 | 135.80 | 1.31 | 3.22 | 65.85 | 0.00 | 22.94 | 337.92 | 123.70 | 0.00 | 0.00 | 0.00 | 0.00 | 7.88 | 0.00 |
| Q8N3X6 | Ligand-dependent nuclear receptor corepressor-like protein | **1.42** | **0.15** | 6456.66 | 3290.19 | 9155.16 | 2366.21 | 10502.31 | 2318.10 | 4042.17 | 7064.94 | 8355.80 | 8815.21 | 12900.06 | 5564.72 | 9975.74 | 9048.06 | 8627.15 |
| P53597 | Succinyl-CoA ligase [ADP/GDP-forming] subunit alpha, mitochondrial | **0.22** | **0.15** | 52.94 | 56.81 | 11.55 | 28.30 | 58.76 | 0.00 | 136.62 | 69.34 | 0.00 | 0.00 | 0.00 | 0.00 | 0.00 | 0.00 | 69.32 |
| P30447 | HLA class I histocompatibility antigen, A-23 alpha chain | **2.16** | **0.15** | 162.74 | 109.24 | 350.83 | 245.44 | 250.38 | 296.47 | 148.22 | 76.11 | 42.51 | 67.12 | 471.45 | 689.51 | 429.99 | 385.81 | 61.07 |
| Q8N5S9 | Calcium/calmodulin-dependent protein kinase kinase 1 | **1.86** | **0.15** | 206.65 | 217.46 | 384.23 | 156.81 | 291.83 | 0.00 | 0.00 | 516.75 | 224.65 | 276.11 | 395.20 | 296.52 | 211.73 | 492.74 | 633.07 |
| Q16891 | Mitochondrial inner membrane protein | **1.47** | **0.15** | 30.37 | 18.85 | 44.79 | 11.35 | 32.80 | 0.00 | 50.24 | 40.14 | 28.68 | 56.13 | 50.44 | 33.45 | 27.76 | 50.18 | 50.78 |
| P00403 | Cytochrome c oxidase subunit 2 | **0.51** | **0.15** | 128.41 | 73.80 | 64.88 | 60.39 | 167.18 | 0.00 | 169.17 | 174.52 | 131.19 | 149.14 | 0.00 | 48.76 | 0.00 | 112.37 | 79.00 |
| P55036 | 26S proteasome non-ATPase regulatory subunit 4 | **0.47** | **0.15** | 88.05 | 49.33 | 41.18 | 49.05 | 115.71 | 0.00 | 107.45 | 108.78 | 108.29 | 112.83 | 82.33 | 0.00 | 0.00 | 0.00 | 51.91 |
| P30492 | HLA class I histocompatibility antigen, B-54 alpha chain | **2.15** | **0.15** | 139.43 | 94.99 | 300.05 | 209.78 | 242.62 | 231.26 | 126.12 | 61.15 | 35.97 | 36.92 | 446.31 | 534.21 | 349.39 | 387.42 | 46.05 |
| P16188 | HLA class I histocompatibility antigen, A-30 alpha chain | **2.08** | **0.15** | 144.48 | 95.55 | 300.02 | 201.32 | 250.38 | 225.81 | 148.22 | 63.86 | 34.13 | 56.79 | 471.45 | 516.83 | 311.17 | 387.42 | 56.46 |
| Q6PI47 | BTB/POZ domain-containing protein KCTD18 | **5.41** | **0.15** | 55.92 | 125.03 | 302.34 | 329.59 | 279.58 | 0.00 | 0.00 | 0.00 | 0.00 | 0.00 | 221.78 | 854.78 | 509.88 | 227.59 | 0.00 |
| P30486 | HLA class I histocompatibility antigen, B-48 alpha chain | **2.93** | **0.15** | 42.96 | 49.35 | 125.73 | 113.69 | 0.00 | 91.73 | 100.15 | 0.00 | 22.89 | 21.80 | 283.83 | 219.57 | 159.15 | 70.03 | 0.00 |
| Q31610 | HLA class I histocompatibility antigen, B-81 alpha chain | **2.93** | **0.15** | 42.96 | 49.35 | 125.73 | 113.69 | 0.00 | 91.73 | 100.15 | 0.00 | 22.89 | 21.80 | 283.83 | 219.57 | 159.15 | 70.03 | 0.00 |
| P30493 | HLA class I histocompatibility antigen, B-55 alpha chain | **2.14** | **0.15** | 140.11 | 94.88 | 300.21 | 209.55 | 242.62 | 231.26 | 129.52 | 61.15 | 35.97 | 37.86 | 446.31 | 534.21 | 349.39 | 387.42 | 46.05 |
| Q29940 | HLA class I histocompatibility antigen, B-59 alpha chain | **2.14** | **0.15** | 140.11 | 94.88 | 300.21 | 209.55 | 242.62 | 231.26 | 129.52 | 61.15 | 35.97 | 37.86 | 446.31 | 534.21 | 349.39 | 387.42 | 46.05 |
| P10768 | S-formylglutathione hydrolase | **0.17** | **0.15** | 23.63 | 24.24 | 4.13 | 10.13 | 46.62 | 0.00 | 0.00 | 50.09 | 21.45 | 0.00 | 0.00 | 0.00 | 24.81 | 0.00 | 0.00 |
| P18463 | HLA class I histocompatibility antigen, B-37 alpha chain | **2.29** | **0.15** | 115.51 | 77.26 | 265.08 | 209.67 | 125.13 | 231.26 | 129.52 | 61.15 | 30.46 | 37.86 | 446.31 | 534.21 | 349.39 | 176.67 | 46.05 |
| P30462 | HLA class I histocompatibility antigen, B-14 alpha chain | **2.29** | **0.15** | 115.51 | 77.26 | 265.08 | 209.67 | 125.13 | 231.26 | 129.52 | 61.15 | 30.46 | 37.86 | 446.31 | 534.21 | 349.39 | 176.67 | 46.05 |
| P30466 | HLA class I histocompatibility antigen, B-18 alpha chain | **2.29** | **0.15** | 115.51 | 77.26 | 265.08 | 209.67 | 125.13 | 231.26 | 129.52 | 61.15 | 30.46 | 37.86 | 446.31 | 534.21 | 349.39 | 176.67 | 46.05 |
| P30508 | HLA class I histocompatibility antigen, Cw-12 alpha chain | **2.06** | **0.15** | 138.43 | 99.66 | 285.34 | 188.00 | 250.38 | 238.14 | 103.79 | 63.86 | 35.97 | 56.79 | 361.83 | 516.83 | 332.70 | 387.42 | 56.46 |
| A6NDJ8 | Putative Rab-43-like protein ENSP00000330714 | **1.48** | **0.15** | 374.77 | 87.73 | 553.17 | 240.19 | 344.40 | 275.99 | 396.56 | 511.72 | 345.18 | 959.06 | 608.97 | 403.64 | 347.52 | 341.37 | 658.42 |
| P20338 | Ras-related protein Rab-4A | **1.48** | **0.15** | 374.77 | 87.73 | 553.17 | 240.19 | 344.40 | 275.99 | 396.56 | 511.72 | 345.18 | 959.06 | 608.97 | 403.64 | 347.52 | 341.37 | 658.42 |
| P61018 | Ras-related protein Rab-4B | **1.48** | **0.15** | 374.77 | 87.73 | 553.17 | 240.19 | 344.40 | 275.99 | 396.56 | 511.72 | 345.18 | 959.06 | 608.97 | 403.64 | 347.52 | 341.37 | 658.42 |
| Q14964 | Ras-related protein Rab-39A | **1.48** | **0.15** | 374.77 | 87.73 | 553.17 | 240.19 | 344.40 | 275.99 | 396.56 | 511.72 | 345.18 | 959.06 | 608.97 | 403.64 | 347.52 | 341.37 | 658.42 |
| Q15771 | Ras-related protein Rab-30 | **1.48** | **0.15** | 374.77 | 87.73 | 553.17 | 240.19 | 344.40 | 275.99 | 396.56 | 511.72 | 345.18 | 959.06 | 608.97 | 403.64 | 347.52 | 341.37 | 658.42 |
| Q6IQ22 | Ras-related protein Rab-12 | **1.48** | **0.15** | 374.77 | 87.73 | 553.17 | 240.19 | 344.40 | 275.99 | 396.56 | 511.72 | 345.18 | 959.06 | 608.97 | 403.64 | 347.52 | 341.37 | 658.42 |
| Q86YS6 | Ras-related protein Rab-43 | **1.48** | **0.15** | 374.77 | 87.73 | 553.17 | 240.19 | 344.40 | 275.99 | 396.56 | 511.72 | 345.18 | 959.06 | 608.97 | 403.64 | 347.52 | 341.37 | 658.42 |
| Q96DA2 | Ras-related protein Rab-39B | **1.48** | **0.15** | 374.77 | 87.73 | 553.17 | 240.19 | 344.40 | 275.99 | 396.56 | 511.72 | 345.18 | 959.06 | 608.97 | 403.64 | 347.52 | 341.37 | 658.42 |
| Q9H082 | Ras-related protein Rab-33B | **1.48** | **0.15** | 374.77 | 87.73 | 553.17 | 240.19 | 344.40 | 275.99 | 396.56 | 511.72 | 345.18 | 959.06 | 608.97 | 403.64 | 347.52 | 341.37 | 658.42 |
| P31040 | Succinate dehydrogenase [ubiquinone] flavoprotein subunit, mitochondrial | **0.34** | **0.15** | 29.17 | 23.97 | 10.02 | 9.03 | 11.06 | 0.00 | 58.18 | 45.74 | 30.85 | 7.20 | 19.43 | 0.00 | 13.54 | 19.92 | 0.00 |
| Q6NZI2 | Polymerase I and transcript release factor | **1.79** | **0.15** | 46.63 | 35.24 | 83.42 | 41.85 | 59.97 | 61.37 | 89.27 | 22.55 | 0.00 | 67.34 | 61.22 | 147.92 | 104.92 | 93.10 | 26.04 |
| P23142 | Fibulin-1 | **4.19** | **0.16** | 43.81 | 39.26 | 183.43 | 203.03 | 62.28 | 100.44 | 38.34 | 17.97 | 0.00 | 36.64 | 72.11 | 121.23 | 497.65 | 372.94 | 0.00 |
| Q9BRA2 | Thioredoxin domain-containing protein 17 | **0.30** | **0.16** | 42.33 | 39.91 | 12.79 | 22.58 | 66.78 | 0.00 | 86.16 | 58.71 | 0.00 | 0.00 | 0.00 | 0.00 | 0.00 | 21.25 | 55.50 |
| Q29960 | HLA class I histocompatibility antigen, Cw-16 alpha chain | **2.06** | **0.16** | 135.34 | 96.96 | 278.95 | 186.72 | 250.38 | 225.81 | 100.69 | 63.86 | 35.97 | 56.79 | 361.83 | 516.83 | 294.38 | 387.42 | 56.46 |
| Q9BYX7 | Putative beta-actin-like protein 3 | **1.55** | **0.16** | 5672.00 | 2172.22 | 8781.63 | 4016.11 | 6610.28 | 4842.51 | 6803.27 | 7813.96 | 2290.00 | 3915.28 | 6915.25 | 13792.98 | 13595.33 | 7690.54 | 6780.40 |
| Q8IYW4 | ENTH domain-containing protein 1 | **3.41** | **0.16** | 32.53 | 72.74 | 111.06 | 92.50 | 0.00 | 0.00 | 0.00 | 162.65 | 0.00 | 226.85 | 150.06 | 166.10 | 0.00 | 0.00 | 123.37 |
| Q14141 | Septin-6 | **1.54** | **0.16** | 39.24 | 27.83 | 60.33 | 17.41 | 0.00 | 41.41 | 65.72 | 64.48 | 24.58 | 74.10 | 34.83 | 82.14 | 50.86 | 52.60 | 67.48 |
| O60701 | UDP-glucose 6-dehydrogenase | **1.99** | **0.16** | 19.10 | 18.15 | 38.05 | 21.98 | 27.40 | 0.00 | 28.05 | 40.06 | 0.00 | 37.06 | 52.33 | 61.63 | 48.78 | 28.46 | 0.00 |
| P30460 | HLA class I histocompatibility antigen, B-8 alpha chain | **2.25** | **0.16** | 117.97 | 81.93 | 265.23 | 209.59 | 125.13 | 243.59 | 129.52 | 61.15 | 30.46 | 37.86 | 446.31 | 534.21 | 349.39 | 177.56 | 46.05 |
| P30480 | HLA class I histocompatibility antigen, B-42 alpha chain | **2.25** | **0.16** | 117.97 | 81.93 | 265.23 | 209.59 | 125.13 | 243.59 | 129.52 | 61.15 | 30.46 | 37.86 | 446.31 | 534.21 | 349.39 | 177.56 | 46.05 |
| Q9NX63 | Coiled-coil-helix-coiled-coil-helix domain-containing protein 3, mitochondrial | **2.16** | **0.16** | 12.39 | 17.03 | 26.81 | 14.23 | 0.00 | 0.00 | 33.05 | 28.91 | 0.00 | 36.43 | 39.18 | 25.27 | 0.00 | 26.42 | 33.58 |
| Q5SZB4 | Uncharacterized protein C9orf50 | **1.39** | **0.16** | 6603.43 | 3169.72 | 9155.16 | 2366.21 | 10502.31 | 2318.10 | 4776.02 | 7064.94 | 8355.80 | 8815.21 | 12900.06 | 5564.72 | 9975.74 | 9048.06 | 8627.15 |
| Q5T011 | Protein SZT2 | **1.39** | **0.16** | 6603.43 | 3169.72 | 9155.16 | 2366.21 | 10502.31 | 2318.10 | 4776.02 | 7064.94 | 8355.80 | 8815.21 | 12900.06 | 5564.72 | 9975.74 | 9048.06 | 8627.15 |
| Q6UXS9 | Inactive caspase-12 | **1.39** | **0.16** | 6603.43 | 3169.72 | 9155.16 | 2366.21 | 10502.31 | 2318.10 | 4776.02 | 7064.94 | 8355.80 | 8815.21 | 12900.06 | 5564.72 | 9975.74 | 9048.06 | 8627.15 |
| O95678 | Keratin, type II cytoskeletal 75 | **0.58** | **0.16** | 1871.80 | 850.18 | 1078.38 | 861.09 | 3158.19 | 1113.54 | 1089.13 | 2089.96 | 1908.19 | 2586.58 | 907.91 | 798.41 | 237.43 | 1515.23 | 424.72 |
| P30479 | HLA class I histocompatibility antigen, B-41 alpha chain | **2.23** | **0.16** | 118.73 | 80.93 | 265.23 | 209.59 | 125.13 | 243.59 | 129.52 | 61.15 | 34.24 | 37.86 | 446.31 | 534.21 | 349.39 | 177.56 | 46.05 |
| Q15084 | Protein disulfide-isomerase A6 | **1.30** | **0.16** | 231.91 | 54.85 | 301.67 | 88.49 | 228.63 | 160.11 | 248.74 | 310.48 | 211.62 | 255.26 | 406.18 | 245.48 | 255.03 | 423.38 | 224.66 |
| Q08380 | Galectin-3-binding protein | **0.55** | **0.16** | 55.50 | 27.92 | 30.37 | 26.56 | 97.39 | 44.22 | 68.07 | 42.44 | 25.39 | 42.82 | 26.49 | 0.00 | 47.72 | 65.19 | 0.00 |
| Q96LA5 | Fc receptor-like protein 2 | **0.35** | **0.16** | 2046.72 | 1706.73 | 722.64 | 580.63 | 3791.39 | 394.26 | 362.57 | 3784.43 | 1900.93 | 619.88 | 300.61 | 1455.94 | 0.00 | 1370.15 | 589.28 |
| P20073 | Annexin A7 | **1.50** | **0.16** | 26.47 | 16.60 | 39.70 | 12.23 | 43.15 | 0.00 | 26.95 | 24.84 | 37.42 | 46.96 | 50.72 | 52.75 | 34.14 | 31.25 | 22.35 |
| P02786 | Transferrin receptor protein 1 | **10.74** | **0.16** | 11.05 | 15.95 | 118.72 | 160.94 | 20.52 | 0.00 | 0.00 | 34.74 | 0.00 | 56.42 | 179.60 | 24.39 | 0.00 | 31.10 | 420.81 |
| Q10567 | AP-1 complex subunit beta-1 | **1.53** | **0.16** | 35.70 | 27.39 | 54.57 | 12.48 | 38.75 | 0.00 | 30.80 | 76.70 | 32.26 | 43.18 | 67.26 | 36.05 | 61.07 | 55.13 | 64.70 |
| P21964 | Catechol O-methyltransferase | **0.43** | **0.16** | 80.10 | 56.75 | 34.54 | 42.80 | 86.95 | 0.00 | 159.58 | 83.44 | 70.50 | 52.22 | 0.00 | 0.00 | 0.00 | 49.47 | 105.56 |
| P35900 | Keratin, type I cytoskeletal 20 | **0.57** | **0.16** | 918.14 | 316.45 | 524.02 | 500.60 | 1279.63 | 539.69 | 966.62 | 653.57 | 1151.19 | 1504.18 | 229.81 | 527.12 | 133.73 | 440.85 | 308.41 |
| Q56NI9 | N-acetyltransferase ESCO2 | **0.49** | **0.16** | 195.05 | 81.09 | 95.87 | 125.03 | 87.33 | 298.73 | 232.07 | 146.67 | 210.45 | 199.30 | 0.00 | 0.00 | 0.00 | 80.89 | 295.04 |
| P00488 | Coagulation factor XIII A chain | **4.70** | **0.16** | 23.04 | 36.90 | 108.26 | 120.25 | 30.54 | 0.00 | 84.66 | 0.00 | 0.00 | 19.54 | 36.70 | 333.97 | 129.14 | 110.90 | 19.29 |
| Q13576 | Ras GTPase-activating-like protein IQGAP2 | **2.70** | **0.16** | 36.18 | 33.37 | 97.73 | 84.80 | 0.00 | 0.00 | 59.95 | 67.28 | 53.64 | 183.46 | 192.42 | 119.88 | 0.00 | 0.00 | 90.62 |
| Q9Y3I0 | tRNA-splicing ligase RtcB homolog | **2.61** | **0.17** | 11.92 | 18.43 | 31.14 | 22.91 | 0.00 | 0.00 | 41.90 | 17.68 | 0.00 | 26.14 | 22.65 | 21.91 | 0.00 | 54.91 | 61.26 |
| Q15437 | Protein transport protein Sec23B | **2.59** | **0.17** | 5.42 | 9.13 | 14.06 | 9.68 | 6.02 | 0.00 | 0.00 | 21.08 | 0.00 | 4.73 | 21.41 | 0.00 | 23.42 | 14.23 | 20.57 |
| P40145 | Adenylate cyclase type 8 | **0.12** | **0.17** | 836.05 | 966.84 | 98.91 | 242.28 | 460.41 | 0.00 | 2087.29 | 1632.57 | 0.00 | 0.00 | 593.47 | 0.00 | 0.00 | 0.00 | 0.00 |
| P02656 | Apolipoprotein C-III | **0.37** | **0.17** | 101.20 | 94.28 | 37.20 | 41.30 | 118.27 | 251.22 | 81.43 | 0.00 | 55.08 | 0.00 | 86.50 | 69.60 | 67.12 | 0.00 | 0.00 |
| Q96FQ6 | Protein S100-A16 | **0.22** | **0.17** | 82.51 | 83.79 | 18.39 | 29.77 | 187.90 | 0.00 | 0.00 | 84.21 | 140.46 | 0.00 | 41.52 | 0.00 | 0.00 | 0.00 | 68.82 |
| Q01995 | Transgelin | **5.22** | **0.17** | 394.92 | 340.62 | 2063.35 | 2515.01 | 797.77 | 694.89 | 333.67 | 107.69 | 40.57 | 269.08 | 1500.93 | 6742.10 | 2931.23 | 892.03 | 44.73 |
| O43829 | Zinc finger and BTB domain-containing protein 14 | **4.45** | **0.17** | 30.50 | 68.19 | 135.61 | 142.23 | 152.48 | 0.00 | 0.00 | 0.00 | 0.00 | 109.46 | 377.17 | 0.00 | 206.96 | 0.00 | 120.06 |
| Q05315 | Galectin-10 | **3.23** | **0.17** | 68.24 | 71.65 | 220.20 | 214.82 | 118.11 | 161.47 | 0.00 | 61.60 | 0.00 | 158.28 | 152.05 | 267.08 | 622.44 | 121.36 | 0.00 |
| P06702 | Protein S100-A9 | **11.72** | **0.17** | 51.87 | 71.12 | 608.19 | 847.11 | 134.61 | 124.77 | 0.00 | 0.00 | 0.00 | 24.63 | 2117.50 | 406.26 | 25.00 | 0.00 | 1075.75 |
| Q9UJ70 | N-acetyl-D-glucosamine kinase | **6.02** | **0.17** | 13.94 | 24.74 | 83.94 | 105.48 | 12.61 | 0.00 | 0.00 | 57.11 | 0.00 | 18.75 | 158.26 | 39.88 | 21.29 | 0.00 | 265.45 |
| P84098 | 60S ribosomal protein L19 | **1.88** | **0.17** | 69.30 | 66.97 | 130.49 | 68.23 | 81.94 | 0.00 | 143.33 | 121.23 | 0.00 | 198.60 | 141.44 | 56.26 | 62.98 | 104.86 | 218.78 |
| Q9HC38 | Glyoxalase domain-containing protein 4 | **0.41** | **0.17** | 42.25 | 28.55 | 17.31 | 26.85 | 48.59 | 0.00 | 68.15 | 66.15 | 28.34 | 53.79 | 0.00 | 0.00 | 0.00 | 0.00 | 50.09 |
| P15559 | NAD(P)H dehydrogenase [quinone] 1 | **0.34** | **0.17** | 43.89 | 26.50 | 14.79 | 36.22 | 39.32 | 0.00 | 63.64 | 52.82 | 63.69 | 0.00 | 88.72 | 0.00 | 0.00 | 0.00 | 0.00 |
| P61006 | Ras-related protein Rab-8A | **1.47** | **0.17** | 294.17 | 56.77 | 433.53 | 200.89 | 357.76 | 222.77 | 251.33 | 337.28 | 301.74 | 770.73 | 551.67 | 366.11 | 278.29 | 221.24 | 413.14 |
| P13796 | Plastin-2 | **1.97** | **0.17** | 131.74 | 66.08 | 258.98 | 188.63 | 163.16 | 204.59 | 41.49 | 163.00 | 86.47 | 110.29 | 426.24 | 544.77 | 245.21 | 156.94 | 70.44 |
| O60493 | Sorting nexin-3 | **19.62** | **0.17** | 4.39 | 9.81 | 86.07 | 121.52 | 21.93 | 0.00 | 0.00 | 0.00 | 0.00 | 42.18 | 32.97 | 328.05 | 30.16 | 83.08 | 0.00 |
| Q96HE7 | ERO1-like protein alpha | **0.30** | **0.17** | 87.84 | 100.67 | 26.63 | 15.79 | 70.05 | 0.00 | 50.25 | 261.53 | 57.39 | 28.88 | 46.49 | 19.75 | 0.00 | 35.94 | 28.74 |
| Q86U42 | Polyadenylate-binding protein 2 | **4.13** | **0.17** | 7.32 | 16.37 | 30.26 | 31.07 | 0.00 | 0.00 | 0.00 | 36.61 | 0.00 | 33.61 | 26.89 | 0.00 | 0.00 | 36.64 | 84.40 |
| Q9GZT3 | SRA stem-loop-interacting RNA-binding protein, mitochondrial | **3.36** | **0.17** | 5.65 | 12.63 | 18.97 | 16.42 | 0.00 | 0.00 | 0.00 | 28.25 | 0.00 | 39.76 | 17.57 | 0.00 | 0.00 | 31.50 | 24.98 |
| P35749 | Myosin-11 | **1.62** | **0.17** | 420.31 | 222.55 | 681.72 | 336.65 | 393.81 | 711.85 | 564.24 | 279.29 | 152.34 | 397.25 | 633.31 | 1188.58 | 729.85 | 886.48 | 254.83 |
| P80404 | 4-aminobutyrate aminotransferase, mitochondrial | **0.26** | **0.17** | 8.72 | 8.84 | 2.28 | 5.59 | 20.74 | 0.00 | 12.14 | 10.72 | 0.00 | 0.00 | 13.68 | 0.00 | 0.00 | 0.00 | 0.00 |
| O15382 | Branched-chain-amino-acid aminotransferase, mitochondrial | **0.16** | **0.17** | 41.21 | 45.99 | 6.73 | 16.48 | 71.68 | 0.00 | 104.52 | 29.85 | 0.00 | 0.00 | 40.37 | 0.00 | 0.00 | 0.00 | 0.00 |
| Q15025 | TNFAIP3-interacting protein 1 | **0.08** | **0.17** | 702.17 | 876.17 | 53.81 | 131.80 | 0.00 | 2138.27 | 790.59 | 581.97 | 0.00 | 0.00 | 0.00 | 0.00 | 322.84 | 0.00 | 0.00 |
| P0C0L4 | Complement C4-A | **3.09** | **0.17** | 36.58 | 12.87 | 112.88 | 117.56 | 24.61 | 38.39 | 50.69 | 22.17 | 47.02 | 138.88 | 49.87 | 325.78 | 128.32 | 34.44 | 0.00 |
| P0C0L5 | Complement C4-B | **3.09** | **0.17** | 36.58 | 12.87 | 112.88 | 117.56 | 24.61 | 38.39 | 50.69 | 22.17 | 47.02 | 138.88 | 49.87 | 325.78 | 128.32 | 34.44 | 0.00 |
| O43237 | Cytoplasmic dynein 1 light intermediate chain 2 | **0.48** | **0.18** | 18.44 | 11.99 | 8.77 | 9.85 | 27.14 | 0.00 | 12.94 | 28.56 | 23.57 | 0.00 | 20.32 | 0.00 | 0.00 | 13.62 | 18.65 |
| Q9NQR4 | Omega-amidase NIT2 | **0.46** | **0.18** | 27.35 | 18.82 | 12.51 | 14.65 | 47.54 | 0.00 | 41.28 | 28.60 | 19.31 | 33.85 | 0.00 | 0.00 | 0.00 | 17.65 | 23.57 |
| P10314 | HLA class I histocompatibility antigen, A-32 alpha chain | **1.87** | **0.18** | 103.79 | 64.65 | 194.17 | 123.08 | 185.71 | 140.95 | 112.32 | 54.32 | 25.64 | 58.06 | 303.48 | 241.46 | 151.34 | 348.43 | 62.27 |
| P30459 | HLA class I histocompatibility antigen, A-74 alpha chain | **1.87** | **0.18** | 103.79 | 64.65 | 194.17 | 123.08 | 185.71 | 140.95 | 112.32 | 54.32 | 25.64 | 58.06 | 303.48 | 241.46 | 151.34 | 348.43 | 62.27 |
| O75643 | U5 small nuclear ribonucleoprotein 200 kDa helicase | **4.74** | **0.18** | 9.50 | 13.26 | 45.08 | 54.46 | 0.00 | 0.00 | 20.17 | 27.35 | 0.00 | 11.57 | 23.13 | 0.00 | 141.09 | 16.01 | 78.70 |
| Q9NPI1 | Bromodomain-containing protein 7 | **3.75** | **0.18** | 109.61 | 151.38 | 411.19 | 434.88 | 246.12 | 0.00 | 301.93 | 0.00 | 0.00 | 0.00 | 553.32 | 1148.44 | 527.77 | 237.59 | 0.00 |
| Q16762 | Thiosulfate sulfurtransferase | **0.25** | **0.18** | 36.58 | 39.17 | 9.06 | 22.19 | 66.74 | 0.00 | 86.59 | 29.55 | 0.00 | 0.00 | 0.00 | 0.00 | 0.00 | 54.34 | 0.00 |
| O76021 | Ribosomal L1 domain-containing protein 1 | **3.54** | **0.18** | 5.61 | 12.54 | 19.85 | 18.40 | 0.00 | 0.00 | 0.00 | 28.05 | 0.00 | 12.16 | 29.44 | 0.00 | 0.00 | 43.23 | 34.26 |
| O75223 | Gamma-glutamylcyclotransferase | **4.18** | **0.18** | 22.52 | 32.82 | 94.05 | 108.83 | 72.18 | 0.00 | 40.41 | 0.00 | 0.00 | 167.56 | 57.55 | 0.00 | 0.00 | 61.47 | 277.72 |
| Q9NR16 | Scavenger receptor cysteine-rich type 1 protein M160 | **0.09** | **0.18** | 2205.62 | 2723.04 | 209.35 | 201.52 | 1615.74 | 2409.25 | 257.57 | 0.00 | 6745.53 | 345.74 | 240.49 | 0.00 | 514.21 | 155.69 | 0.00 |
| P18124 | 60S ribosomal protein L7 | **1.56** | **0.18** | 130.72 | 86.90 | 203.30 | 78.57 | 86.05 | 56.90 | 174.80 | 262.92 | 72.92 | 193.69 | 205.78 | 153.78 | 96.23 | 243.64 | 326.69 |
| O60664 | Perilipin-3 | **0.62** | **0.18** | 84.99 | 52.50 | 52.28 | 16.79 | 173.05 | 93.28 | 47.74 | 61.15 | 49.71 | 63.47 | 65.58 | 25.83 | 54.16 | 66.54 | 38.12 |
| P29400 | Collagen alpha-5(IV) chain | **3.95** | **0.18** | 82.16 | 112.39 | 324.38 | 356.29 | 109.71 | 0.00 | 266.56 | 34.54 | 0.00 | 30.44 | 188.42 | 331.17 | 37.84 | 366.26 | 992.16 |
| P53396 | ATP-citrate synthase | **1.78** | **0.18** | 22.64 | 17.72 | 40.23 | 21.70 | 7.20 | 0.00 | 39.20 | 33.55 | 33.24 | 15.93 | 55.55 | 26.53 | 19.90 | 60.38 | 63.07 |
| P37837 | Transaldolase | **1.48** | **0.18** | 140.21 | 48.20 | 207.13 | 92.72 | 142.19 | 102.48 | 193.95 | 180.27 | 82.15 | 98.51 | 323.19 | 301.25 | 133.07 | 232.12 | 154.65 |
| Q7Z2K6 | Endoplasmic reticulum metallopeptidase 1 | **3.54** | **0.18** | 41.42 | 44.91 | 146.44 | 155.91 | 65.45 | 0.00 | 0.00 | 104.96 | 36.70 | 428.09 | 27.38 | 105.88 | 0.00 | 206.84 | 110.43 |
| P16189 | HLA class I histocompatibility antigen, A-31 alpha chain | **1.83** | **0.18** | 103.79 | 64.65 | 189.82 | 125.34 | 185.71 | 140.95 | 112.32 | 54.32 | 25.64 | 58.06 | 303.48 | 241.46 | 125.19 | 348.43 | 62.27 |
| P62495 | Eukaryotic peptide chain release factor subunit 1 | **3.70** | **0.18** | 6.34 | 14.18 | 23.47 | 23.14 | 0.00 | 0.00 | 0.00 | 31.70 | 0.00 | 23.22 | 50.89 | 0.00 | 0.00 | 15.65 | 51.07 |
| P46063 | ATP-dependent DNA helicase Q1 | **3.03** | **0.18** | 7.87 | 17.60 | 23.89 | 18.96 | 0.00 | 0.00 | 39.36 | 0.00 | 0.00 | 0.00 | 31.93 | 40.19 | 30.55 | 40.66 | 0.00 |
| Q6XUX3 | Dual serine/threonine and tyrosine protein kinase | **3.51** | **0.18** | 256.78 | 358.38 | 900.57 | 937.39 | 740.01 | 543.89 | 0.00 | 0.00 | 0.00 | 189.47 | 799.63 | 2502.67 | 1452.73 | 458.94 | 0.00 |
| P20336 | Ras-related protein Rab-3A | **1.27** | **0.18** | 374.77 | 87.73 | 476.14 | 135.10 | 344.40 | 275.99 | 396.56 | 511.72 | 345.18 | 496.89 | 608.97 | 403.64 | 347.52 | 341.37 | 658.42 |
| P20337 | Ras-related protein Rab-3B | **1.27** | **0.18** | 374.77 | 87.73 | 476.14 | 135.10 | 344.40 | 275.99 | 396.56 | 511.72 | 345.18 | 496.89 | 608.97 | 403.64 | 347.52 | 341.37 | 658.42 |
| Q96E17 | Ras-related protein Rab-3C | **1.27** | **0.18** | 374.77 | 87.73 | 476.14 | 135.10 | 344.40 | 275.99 | 396.56 | 511.72 | 345.18 | 496.89 | 608.97 | 403.64 | 347.52 | 341.37 | 658.42 |
| Q8N8R7 | ARL14 effector protein | **3.18** | **0.19** | 32.49 | 72.65 | 103.36 | 88.34 | 0.00 | 0.00 | 0.00 | 162.44 | 0.00 | 218.87 | 159.58 | 137.24 | 0.00 | 0.00 | 104.49 |
| Q15691 | Microtubule-associated protein RP/EB family member 1 | **1.60** | **0.19** | 37.57 | 23.46 | 60.15 | 27.91 | 41.05 | 0.00 | 34.48 | 50.00 | 62.33 | 32.20 | 105.88 | 69.29 | 38.93 | 41.91 | 72.71 |
| P49721 | Proteasome subunit beta type-2 | **1.45** | **0.19** | 33.07 | 23.09 | 48.09 | 10.63 | 35.55 | 0.00 | 62.46 | 42.86 | 24.50 | 41.16 | 38.60 | 48.40 | 38.88 | 64.03 | 57.47 |
| Q9Y2K3 | Myosin-15 | **2.96** | **0.19** | 200.18 | 301.41 | 593.45 | 547.88 | 0.00 | 0.00 | 0.00 | 677.73 | 323.17 | 109.11 | 333.07 | 880.01 | 1444.24 | 0.00 | 794.25 |
| P39023 | 60S ribosomal protein L3 | **1.61** | **0.19** | 98.66 | 80.94 | 159.16 | 59.81 | 75.32 | 0.00 | 156.21 | 203.46 | 58.30 | 82.33 | 238.71 | 147.44 | 104.57 | 174.86 | 207.04 |
| Q9NWU1 | 3-oxoacyl-[acyl-carrier-protein] synthase, mitochondrial | **1.50** | **0.19** | 282.21 | 174.52 | 422.92 | 153.65 | 313.41 | 0.00 | 481.93 | 307.02 | 308.69 | 392.57 | 662.15 | 198.78 | 371.45 | 407.44 | 505.13 |
| Q8NB25 | Protein FAM184A | **0.28** | **0.19** | 109.45 | 110.76 | 30.18 | 73.92 | 108.31 | 0.00 | 240.71 | 198.25 | 0.00 | 181.08 | 0.00 | 0.00 | 0.00 | 0.00 | 0.00 |
| P07954 | Fumarate hydratase, mitochondrial | **0.45** | **0.19** | 45.87 | 34.92 | 20.83 | 23.38 | 49.70 | 0.00 | 64.39 | 90.34 | 24.92 | 50.94 | 0.00 | 37.56 | 0.00 | 0.00 | 36.49 |
| Q9Y262 | Eukaryotic translation initiation factor 3 subunit L | **2.97** | **0.19** | 6.39 | 14.28 | 18.96 | 14.93 | 0.00 | 0.00 | 0.00 | 31.93 | 0.00 | 33.49 | 27.89 | 0.00 | 0.00 | 26.30 | 26.08 |
| Q92882 | Osteoclast-stimulating factor 1 | **2.82** | **0.19** | 3.39 | 4.73 | 9.55 | 8.64 | 9.75 | 0.00 | 0.00 | 7.20 | 0.00 | 4.93 | 25.15 | 0.00 | 11.97 | 9.06 | 6.20 |
| P30504 | HLA class I histocompatibility antigen, Cw-4 alpha chain | **1.99** | **0.19** | 123.09 | 92.20 | 245.11 | 171.87 | 242.62 | 200.51 | 75.17 | 61.15 | 35.97 | 36.92 | 294.94 | 444.87 | 264.69 | 387.42 | 41.82 |
| P30510 | HLA class I histocompatibility antigen, Cw-14 alpha chain | **1.99** | **0.19** | 123.09 | 92.20 | 245.11 | 171.87 | 242.62 | 200.51 | 75.17 | 61.15 | 35.97 | 36.92 | 294.94 | 444.87 | 264.69 | 387.42 | 41.82 |
| P16401 | Histone H1.5 | **1.62** | **0.19** | 681.19 | 320.01 | 1100.38 | 588.65 | 324.06 | 688.71 | 1063.20 | 925.35 | 404.65 | 1667.97 | 1872.47 | 564.70 | 469.48 | 774.73 | 1252.90 |
| Q58FF3 | Putative endoplasmin-like protein | **1.23** | **0.19** | 189.04 | 53.60 | 231.75 | 46.36 | 146.07 | 160.56 | 144.28 | 240.82 | 253.46 | 255.42 | 222.45 | 198.65 | 314.94 | 201.41 | 197.64 |
| Q8WXG9 | G-protein coupled receptor 98 | **2.65** | **0.19** | 160.29 | 237.36 | 425.28 | 356.31 | 0.00 | 528.54 | 272.89 | 0.00 | 0.00 | 654.36 | 408.23 | 835.22 | 0.00 | 653.87 | 0.00 |
| Q29865 | HLA class I histocompatibility antigen, Cw-18 alpha chain | **2.08** | **0.19** | 98.48 | 66.49 | 204.45 | 154.82 | 125.13 | 200.51 | 75.17 | 61.15 | 30.46 | 36.92 | 260.85 | 444.87 | 264.69 | 177.56 | 41.82 |
| P01023 | Alpha-2-macroglobulin | **0.49** | **0.19** | 294.74 | 209.70 | 144.03 | 84.37 | 181.27 | 538.99 | 489.60 | 53.29 | 210.54 | 167.41 | 71.95 | 277.17 | 177.20 | 130.49 | 39.93 |
| P04632 | Calpain small subunit 1 | **0.53** | **0.19** | 235.75 | 188.09 | 124.49 | 46.72 | 569.04 | 143.69 | 118.04 | 158.63 | 189.34 | 111.10 | 175.52 | 137.88 | 97.50 | 53.58 | 171.34 |
| Q14764 | Major vault protein | **0.50** | **0.19** | 63.07 | 45.58 | 31.37 | 28.53 | 142.05 | 36.05 | 49.59 | 58.36 | 29.32 | 0.00 | 40.23 | 75.55 | 39.69 | 32.77 | 0.00 |
| Q14651 | Plastin-1 | **1.74** | **0.19** | 99.00 | 38.93 | 172.63 | 110.54 | 123.05 | 148.33 | 56.02 | 103.04 | 64.57 | 96.88 | 248.39 | 349.66 | 161.26 | 136.84 | 42.75 |
| Q6NXT2 | Histone H3.3C | **1.54** | **0.19** | 2419.39 | 1105.19 | 3736.58 | 1821.83 | 1420.01 | 1382.93 | 3395.95 | 3754.65 | 2143.40 | 2272.10 | 2968.68 | 3292.09 | 3118.61 | 3402.38 | 7365.60 |
| Q96BM9 | ADP-ribosylation factor-like protein 8A | **2.55** | **0.19** | 11.62 | 16.01 | 29.63 | 24.57 | 0.00 | 0.00 | 0.00 | 31.46 | 26.66 | 60.69 | 42.16 | 0.00 | 40.40 | 0.00 | 34.55 |
| P30501 | HLA class I histocompatibility antigen, Cw-2 alpha chain | **2.09** | **0.19** | 118.36 | 76.32 | 247.18 | 191.31 | 136.76 | 238.14 | 103.79 | 76.11 | 37.01 | 51.54 | 327.74 | 543.31 | 332.70 | 175.95 | 51.84 |
| Q99439 | Calponin-2 | **1.98** | **0.19** | 66.19 | 32.10 | 131.05 | 98.24 | 82.94 | 103.99 | 77.36 | 41.23 | 25.41 | 74.47 | 155.38 | 121.58 | 295.78 | 139.06 | 0.00 |
| P13473 | Lysosome-associated membrane glycoprotein 2 | **7.87** | **0.20** | 171.88 | 384.35 | 1353.08 | 1838.96 | 0.00 | 0.00 | 0.00 | 859.42 | 0.00 | 4687.04 | 1374.22 | 0.00 | 31.10 | 17.18 | 2008.92 |
| P46776 | 60S ribosomal protein L27a | **1.55** | **0.20** | 107.66 | 95.87 | 167.32 | 40.19 | 73.43 | 0.00 | 157.00 | 246.89 | 60.99 | 153.06 | 216.12 | 174.93 | 97.90 | 169.88 | 192.00 |
| O15143 | Actin-related protein 2/3 complex subunit 1B | **1.47** | **0.20** | 51.73 | 11.11 | 76.05 | 37.30 | 54.60 | 42.51 | 67.92 | 53.49 | 40.12 | 55.66 | 57.78 | 145.83 | 54.69 | 91.58 | 50.76 |
| P61626 | Lysozyme C | **5.61** | **0.20** | 28.57 | 31.49 | 160.27 | 216.40 | 41.66 | 75.02 | 0.00 | 0.00 | 26.15 | 0.00 | 310.75 | 525.29 | 125.58 | 0.00 | 0.00 |
| Q96IU4 | Alpha/beta hydrolase domain-containing protein 14B | **0.29** | **0.20** | 33.24 | 32.88 | 9.61 | 23.53 | 70.78 | 0.00 | 59.66 | 35.74 | 0.00 | 0.00 | 0.00 | 0.00 | 0.00 | 0.00 | 57.63 |
| P09382 | Galectin-1 | **2.51** | **0.20** | 197.01 | 90.57 | 495.38 | 489.15 | 291.59 | 231.23 | 247.17 | 153.19 | 61.87 | 180.20 | 137.99 | 638.79 | 1370.74 | 569.77 | 74.76 |
| P60174 | Triosephosphate isomerase | **0.69** | **0.20** | 1251.47 | 520.31 | 866.16 | 402.13 | 1662.58 | 491.50 | 1260.77 | 1794.49 | 1048.02 | 964.21 | 367.95 | 433.03 | 1066.40 | 935.05 | 1430.32 |
| P18669 | Phosphoglycerate mutase 1 | **0.64** | **0.20** | 375.75 | 238.27 | 240.98 | 32.82 | 332.10 | 101.46 | 363.42 | 759.13 | 322.64 | 248.20 | 195.58 | 215.37 | 240.13 | 257.07 | 289.52 |
| P62244 | 40S ribosomal protein S15a | **1.75** | **0.20** | 63.19 | 43.43 | 110.75 | 65.57 | 70.31 | 0.00 | 83.00 | 116.34 | 46.33 | 150.08 | 121.86 | 112.20 | 0.00 | 86.18 | 194.15 |
| P01892 | HLA class I histocompatibility antigen, A-2 alpha chain | **1.83** | **0.20** | 101.30 | 63.09 | 185.26 | 128.84 | 185.71 | 128.52 | 112.32 | 54.32 | 25.64 | 54.56 | 303.48 | 241.46 | 101.38 | 348.43 | 62.27 |
| Q86X40 | Leucine-rich repeat-containing protein 28 | **2.63** | **0.20** | 701.78 | 508.68 | 1844.86 | 1862.72 | 698.69 | 1294.48 | 453.77 | 1061.96 | 0.00 | 2172.80 | 4379.34 | 226.17 | 0.00 | 626.69 | 3664.16 |
| P14923 | Junction plakoglobin | **2.20** | **0.20** | 42.60 | 26.54 | 93.72 | 82.54 | 66.17 | 0.00 | 58.89 | 53.41 | 34.52 | 127.90 | 174.31 | 34.15 | 0.00 | 31.35 | 194.61 |
| Q9HAW8 | UDP-glucuronosyltransferase 1-10 | **0.01** | **0.20** | 112.05 | 161.90 | 1.31 | 3.22 | 65.85 | 0.00 | 24.66 | 396.66 | 73.05 | 0.00 | 0.00 | 0.00 | 0.00 | 7.88 | 0.00 |
| Q06210 | Glutamine--fructose-6-phosphate aminotransferase [isomerizing] 1 | **3.11** | **0.20** | 6.89 | 9.65 | 21.42 | 21.73 | 0.00 | 0.00 | 0.00 | 20.05 | 14.41 | 0.00 | 59.19 | 23.58 | 0.00 | 21.03 | 24.73 |
| P13987 | CD59 glycoprotein | **0.33** | **0.20** | 67.59 | 63.42 | 22.21 | 34.50 | 107.36 | 0.00 | 135.53 | 95.06 | 0.00 | 70.51 | 0.00 | 0.00 | 0.00 | 62.76 | 0.00 |
| P13928 | Annexin A8 | **3.92** | **0.20** | 31.95 | 20.86 | 125.22 | 154.88 | 33.97 | 0.00 | 28.88 | 57.47 | 39.42 | 193.90 | 50.07 | 69.85 | 0.00 | 27.28 | 410.25 |
| Q5VT79 | Annexin A8-like protein 2 | **3.92** | **0.20** | 31.95 | 20.86 | 125.22 | 154.88 | 33.97 | 0.00 | 28.88 | 57.47 | 39.42 | 193.90 | 50.07 | 69.85 | 0.00 | 27.28 | 410.25 |
| P25788 | Proteasome subunit alpha type-3 | **1.52** | **0.20** | 57.89 | 38.54 | 87.85 | 33.89 | 63.18 | 0.00 | 106.60 | 69.37 | 50.31 | 59.40 | 111.87 | 144.29 | 79.97 | 57.62 | 73.92 |
| O43598 | 2'-deoxynucleoside 5'-phosphate N-hydrolase 1 | **2.76** | **0.21** | 13.37 | 21.93 | 36.97 | 32.85 | 0.00 | 0.00 | 50.50 | 0.00 | 16.35 | 62.58 | 55.38 | 0.00 | 27.23 | 76.61 | 0.00 |
| Q04637 | Eukaryotic translation initiation factor 4 gamma 1 | **1.96** | **0.21** | 14.19 | 14.80 | 27.85 | 17.76 | 22.76 | 0.00 | 14.00 | 34.20 | 0.00 | 17.77 | 34.97 | 0.00 | 23.24 | 45.10 | 46.04 |
| Q9H0C2 | ADP/ATP translocase 4 | **1.62** | **0.21** | 238.84 | 143.37 | 385.96 | 201.09 | 268.51 | 0.00 | 282.16 | 387.59 | 255.93 | 275.53 | 738.57 | 318.20 | 143.91 | 429.90 | 409.62 |
| P46976 | Glycogenin-1 | **0.14** | **0.21** | 27.95 | 35.58 | 3.81 | 9.33 | 82.62 | 44.55 | 12.59 | 0.00 | 0.00 | 0.00 | 0.00 | 0.00 | 0.00 | 0.00 | 22.87 |
| P62753 | 40S ribosomal protein S6 | **1.62** | **0.21** | 94.00 | 73.17 | 152.44 | 69.31 | 84.11 | 25.94 | 168.02 | 170.80 | 21.12 | 154.23 | 141.15 | 109.17 | 63.38 | 178.45 | 268.24 |
| P16070 | CD44 antigen | **4.32** | **0.21** | 39.69 | 38.02 | 171.58 | 212.52 | 0.00 | 72.34 | 47.69 | 78.44 | 0.00 | 61.36 | 603.56 | 96.05 | 114.80 | 67.10 | 86.63 |
| O00151 | PDZ and LIM domain protein 1 | **0.45** | **0.21** | 107.09 | 81.12 | 48.14 | 63.44 | 173.79 | 91.27 | 200.66 | 0.00 | 69.72 | 32.07 | 0.00 | 0.00 | 132.78 | 123.95 | 0.00 |
| P17693 | HLA class I histocompatibility antigen, alpha chain G | **2.41** | **0.21** | 179.12 | 137.45 | 432.29 | 414.80 | 222.13 | 395.54 | 143.87 | 82.94 | 51.12 | 54.26 | 643.17 | 1067.56 | 632.76 | 151.12 | 44.85 |
| P61326 | Protein mago nashi homolog | **2.46** | **0.21** | 23.92 | 33.02 | 58.79 | 48.78 | 0.00 | 0.00 | 53.92 | 65.70 | 0.00 | 66.87 | 108.51 | 0.00 | 0.00 | 70.51 | 106.86 |
| Q96A72 | Protein mago nashi homolog 2 | **2.46** | **0.21** | 23.92 | 33.02 | 58.79 | 48.78 | 0.00 | 0.00 | 53.92 | 65.70 | 0.00 | 66.87 | 108.51 | 0.00 | 0.00 | 70.51 | 106.86 |
| Q5T2P8 | Annexin A8-like protein 1 | **3.56** | **0.21** | 31.26 | 19.85 | 111.23 | 135.15 | 33.97 | 0.00 | 28.88 | 54.05 | 39.42 | 154.94 | 50.07 | 69.85 | 0.00 | 27.28 | 365.28 |
| Q9BVK6 | Transmembrane emp24 domain-containing protein 9 | **1.76** | **0.21** | 53.70 | 42.98 | 94.65 | 54.99 | 65.10 | 0.00 | 31.80 | 116.10 | 55.50 | 147.19 | 125.19 | 137.24 | 0.00 | 88.44 | 69.86 |
| P58107 | Epiplakin | **0.60** | **0.21** | 92.86 | 58.95 | 55.50 | 31.07 | 128.96 | 0.00 | 125.77 | 68.70 | 140.89 | 104.78 | 26.96 | 43.85 | 50.25 | 27.00 | 80.19 |
| P30512 | HLA class I histocompatibility antigen, A-29 alpha chain | **1.79** | **0.21** | 103.79 | 64.65 | 185.78 | 128.19 | 185.71 | 140.95 | 112.32 | 54.32 | 25.64 | 58.06 | 303.48 | 241.46 | 100.98 | 348.43 | 62.27 |
| Q02878 | 60S ribosomal protein L6 | **1.42** | **0.21** | 114.37 | 44.06 | 162.42 | 68.06 | 81.62 | 100.29 | 100.64 | 191.94 | 97.37 | 193.25 | 159.89 | 66.23 | 102.65 | 201.37 | 251.10 |
| Q9UKX2 | Myosin-2 | **1.56** | **0.21** | 246.82 | 140.00 | 385.83 | 190.74 | 294.97 | 0.00 | 288.49 | 301.78 | 348.84 | 401.44 | 335.91 | 741.09 | 389.76 | 214.17 | 232.60 |
| P49189 | 4-trimethylaminobutyraldehyde dehydrogenase | **0.50** | **0.21** | 37.04 | 23.32 | 18.57 | 22.06 | 38.21 | 0.00 | 37.27 | 63.99 | 45.72 | 0.00 | 52.66 | 0.00 | 0.00 | 28.49 | 30.28 |
| P30044 | Peroxiredoxin-5, mitochondrial | **0.67** | **0.22** | 268.70 | 152.84 | 179.94 | 55.54 | 526.04 | 145.30 | 283.61 | 175.75 | 212.78 | 243.97 | 246.86 | 132.79 | 116.50 | 183.78 | 155.73 |
| P20340 | Ras-related protein Rab-6A | **1.30** | **0.22** | 307.30 | 129.31 | 400.93 | 104.56 | 344.40 | 275.99 | 220.21 | 511.72 | 184.18 | 361.91 | 608.97 | 403.64 | 347.52 | 341.37 | 342.14 |
| O00764 | Pyridoxal kinase | **2.28** | **0.22** | 13.22 | 12.44 | 30.11 | 25.91 | 25.26 | 0.00 | 23.65 | 17.17 | 0.00 | 0.00 | 28.03 | 59.95 | 10.51 | 62.64 | 19.52 |
| Q86SE5 | RNA-binding Raly-like protein | **1.84** | **0.22** | 41.34 | 38.79 | 76.05 | 46.37 | 55.92 | 0.00 | 69.40 | 81.36 | 0.00 | 90.92 | 81.12 | 47.92 | 0.00 | 131.98 | 104.38 |
| P50914 | 60S ribosomal protein L14 | **1.55** | **0.22** | 94.18 | 67.54 | 146.10 | 61.99 | 77.36 | 34.88 | 63.83 | 210.10 | 84.72 | 154.43 | 70.57 | 114.32 | 119.44 | 165.15 | 252.67 |
| P59665 | Neutrophil defensin 1 | **6.98** | **0.22** | 71.87 | 73.52 | 501.74 | 745.28 | 141.66 | 151.76 | 65.91 | 0.00 | 0.00 | 0.00 | 1449.72 | 1476.07 | 0.00 | 84.62 | 0.00 |
| P59666 | Neutrophil defensin 3 | **6.98** | **0.22** | 71.87 | 73.52 | 501.74 | 745.28 | 141.66 | 151.76 | 65.91 | 0.00 | 0.00 | 0.00 | 1449.72 | 1476.07 | 0.00 | 84.62 | 0.00 |
| P60891 | Ribose-phosphate pyrophosphokinase 1 | **0.28** | **0.22** | 14.42 | 16.01 | 4.02 | 9.85 | 16.71 | 0.00 | 0.00 | 38.92 | 16.48 | 0.00 | 24.14 | 0.00 | 0.00 | 0.00 | 0.00 |
| Q9UBF2 | Coatomer subunit gamma-2 | **2.42** | **0.22** | 11.18 | 16.84 | 27.06 | 21.97 | 0.00 | 0.00 | 18.01 | 37.88 | 0.00 | 0.00 | 39.73 | 46.38 | 47.37 | 0.00 | 28.89 |
| O75369 | Filamin-B | **1.73** | **0.22** | 126.41 | 55.28 | 219.20 | 147.38 | 173.83 | 49.82 | 153.10 | 86.47 | 168.85 | 72.27 | 225.90 | 406.14 | 375.54 | 176.72 | 58.65 |
| P14618 | Pyruvate kinase PKM | **0.72** | **0.22** | 1127.28 | 482.43 | 807.33 | 172.51 | 1677.06 | 603.48 | 697.21 | 1538.79 | 1119.87 | 788.83 | 643.52 | 831.08 | 706.02 | 741.02 | 1133.52 |
| P04080 | Cystatin-B | **0.60** | **0.22** | 443.39 | 259.78 | 266.93 | 183.61 | 889.58 | 239.58 | 333.18 | 442.27 | 312.34 | 242.52 | 559.54 | 116.99 | 49.63 | 254.28 | 378.65 |
| Q12905 | Interleukin enhancer-binding factor 2 | **1.57** | **0.22** | 84.78 | 69.44 | 133.22 | 52.76 | 67.87 | 0.00 | 135.63 | 173.19 | 47.22 | 144.65 | 163.63 | 82.97 | 57.00 | 153.76 | 197.28 |
| P10599 | Thioredoxin | **0.65** | **0.22** | 168.82 | 66.46 | 110.38 | 78.17 | 217.28 | 256.99 | 152.16 | 102.75 | 114.92 | 128.08 | 0.00 | 69.68 | 109.38 | 116.87 | 238.26 |
| O75955 | Flotillin-1 | **0.30** | **0.22** | 15.25 | 15.82 | 4.55 | 11.14 | 0.00 | 0.00 | 31.53 | 31.57 | 13.13 | 0.00 | 0.00 | 0.00 | 0.00 | 27.29 | 0.00 |
| Q71DI3 | Histone H3.2 | **1.40** | **0.22** | 2311.71 | 1230.42 | 3227.80 | 1079.68 | 1420.01 | 1382.93 | 3395.95 | 3894.61 | 1465.07 | 2305.75 | 3122.07 | 3292.09 | 2102.16 | 3402.38 | 5142.36 |
| Q99496 | E3 ubiquitin-protein ligase RING2 | **0.48** | **0.22** | 2035.01 | 1466.37 | 978.71 | 1208.82 | 3900.14 | 1339.80 | 2716.07 | 2219.05 | 0.00 | 3246.97 | 508.71 | 980.98 | 0.00 | 1135.61 | 0.00 |
| Q9H2U2 | Inorganic pyrophosphatase 2, mitochondrial | **0.44** | **0.22** | 61.11 | 42.02 | 26.73 | 44.39 | 84.64 | 0.00 | 51.10 | 57.10 | 112.68 | 0.00 | 27.43 | 114.17 | 0.00 | 0.00 | 18.80 |
| P62491 | Ras-related protein Rab-11A | **1.42** | **0.22** | 102.91 | 32.97 | 146.44 | 67.51 | 125.22 | 64.02 | 71.85 | 137.70 | 115.74 | 142.26 | 158.95 | 93.13 | 95.88 | 114.69 | 273.74 |
| Q15121 | Astrocytic phosphoprotein PEA-15 | **2.18** | **0.22** | 17.07 | 24.42 | 37.28 | 26.41 | 52.67 | 0.00 | 0.00 | 0.00 | 32.69 | 0.00 | 47.79 | 76.84 | 48.51 | 27.00 | 23.54 |
| P18465 | HLA class I histocompatibility antigen, B-57 alpha chain | **2.02** | **0.22** | 161.71 | 123.88 | 326.71 | 257.23 | 349.87 | 213.64 | 126.12 | 82.94 | 35.97 | 37.32 | 446.31 | 715.59 | 349.39 | 366.82 | 44.85 |
| Q969T7 | 7-methylguanosine phosphate-specific 5'-nucleotidase | **1.58** | **0.22** | 9402.00 | 5788.77 | 14818.69 | 7598.76 | 15065.69 | 319.10 | 13242.59 | 7670.79 | 10711.82 | 16927.01 | 7140.18 | 17877.48 | 26819.15 | 13742.41 | 6405.89 |
| Q15907 | Ras-related protein Rab-11B | **1.42** | **0.22** | 102.91 | 32.97 | 146.60 | 67.99 | 125.22 | 64.02 | 71.85 | 137.70 | 115.74 | 142.26 | 158.95 | 109.69 | 80.29 | 114.69 | 273.74 |
| P20810 | Calpastatin | **0.22** | **0.23** | 70.02 | 83.98 | 15.73 | 20.26 | 200.55 | 0.00 | 100.46 | 49.07 | 0.00 | 20.55 | 22.99 | 0.00 | 0.00 | 0.00 | 50.87 |
| P02730 | Band 3 anion transport protein | **0.25** | **0.23** | 94.51 | 110.61 | 23.34 | 20.95 | 55.26 | 0.00 | 123.04 | 20.40 | 273.85 | 40.10 | 19.56 | 0.00 | 51.22 | 29.15 | 0.00 |
| P58546 | Myotrophin | **0.65** | **0.23** | 102.26 | 50.54 | 66.84 | 39.91 | 168.38 | 109.04 | 63.13 | 42.38 | 128.39 | 79.67 | 59.62 | 0.00 | 57.20 | 83.88 | 120.66 |
| O00584 | Ribonuclease T2 | **0.14** | **0.23** | 84.72 | 113.72 | 11.98 | 10.78 | 143.47 | 22.17 | 0.00 | 257.95 | 0.00 | 22.57 | 25.31 | 0.00 | 0.00 | 10.52 | 13.51 |
| Q5TZA2 | Rootletin | **0.62** | **0.23** | 173.86 | 63.67 | 107.55 | 97.90 | 258.41 | 139.15 | 88.77 | 192.38 | 190.58 | 182.29 | 0.00 | 0.00 | 149.40 | 77.42 | 236.18 |
| Q9NVJ2 | ADP-ribosylation factor-like protein 8B | **2.39** | **0.23** | 11.62 | 16.01 | 27.82 | 23.85 | 0.00 | 0.00 | 0.00 | 31.46 | 26.66 | 60.69 | 31.27 | 0.00 | 40.40 | 0.00 | 34.55 |
| P31150 | Rab GDP dissociation inhibitor alpha | **1.41** | **0.23** | 101.96 | 61.28 | 144.22 | 47.50 | 112.25 | 29.96 | 137.26 | 178.94 | 51.38 | 96.14 | 154.01 | 108.75 | 106.92 | 194.29 | 205.20 |
| P39019 | 40S ribosomal protein S19 | **1.17** | **0.23** | 141.74 | 36.25 | 165.76 | 25.52 | 121.42 | 173.25 | 160.52 | 165.79 | 87.72 | 194.43 | 198.10 | 163.85 | 158.03 | 141.54 | 138.60 |
| P78417 | Glutathione S-transferase omega-1 | **0.76** | **0.23** | 121.60 | 47.74 | 92.40 | 26.57 | 139.39 | 51.55 | 166.23 | 155.99 | 94.83 | 71.88 | 110.16 | 122.94 | 108.44 | 88.48 | 52.53 |
| P08579 | U2 small nuclear ribonucleoprotein B'' | **3.08** | **0.23** | 8.92 | 19.95 | 27.49 | 26.60 | 0.00 | 0.00 | 0.00 | 44.60 | 0.00 | 25.69 | 70.97 | 29.37 | 0.00 | 0.00 | 38.91 |
| P08238 | Heat shock protein HSP 90-beta | **1.39** | **0.23** | 939.48 | 279.92 | 1310.40 | 588.36 | 1042.14 | 618.96 | 930.20 | 1348.24 | 757.86 | 1027.68 | 1311.08 | 983.45 | 551.68 | 1865.76 | 2122.77 |
| O94979 | Protein transport protein Sec31A | **9.19** | **0.23** | 6.03 | 9.63 | 55.41 | 85.07 | 0.00 | 0.00 | 8.07 | 22.09 | 0.00 | 228.97 | 20.65 | 21.13 | 20.80 | 16.01 | 24.92 |
| P63173 | 60S ribosomal protein L38 | **2.59** | **0.23** | 22.34 | 20.51 | 57.89 | 58.82 | 33.87 | 0.00 | 37.88 | 39.96 | 0.00 | 60.61 | 95.60 | 0.00 | 0.00 | 39.29 | 151.82 |
| P25786 | Proteasome subunit alpha type-1 | **1.73** | **0.23** | 31.02 | 29.53 | 53.57 | 28.75 | 41.37 | 0.00 | 64.59 | 49.16 | 0.00 | 44.85 | 68.14 | 60.75 | 0.00 | 66.73 | 80.97 |
| Q99584 | Protein S100-A13 | **0.29** | **0.23** | 35.47 | 40.03 | 10.24 | 25.07 | 92.46 | 0.00 | 0.00 | 59.02 | 25.87 | 0.00 | 0.00 | 61.42 | 0.00 | 0.00 | 0.00 |
| P13747 | HLA class I histocompatibility antigen, alpha chain E | **2.70** | **0.23** | 133.92 | 78.66 | 362.00 | 388.52 | 222.13 | 213.59 | 99.80 | 82.94 | 51.12 | 54.26 | 435.40 | 1067.56 | 433.97 | 135.99 | 44.85 |
| P59190 | Ras-related protein Rab-15 | **1.35** | **0.23** | 348.01 | 98.79 | 468.47 | 188.97 | 357.76 | 222.77 | 365.52 | 492.27 | 301.74 | 770.73 | 477.32 | 366.11 | 278.29 | 317.25 | 601.10 |
| Q92930 | Ras-related protein Rab-8B | **1.35** | **0.23** | 348.01 | 98.79 | 468.47 | 188.97 | 357.76 | 222.77 | 365.52 | 492.27 | 301.74 | 770.73 | 477.32 | 366.11 | 278.29 | 317.25 | 601.10 |
| Q07960 | Rho GTPase-activating protein 1 | **1.57** | **0.24** | 55.39 | 40.79 | 86.76 | 40.62 | 69.77 | 0.00 | 89.02 | 92.40 | 25.75 | 58.15 | 48.70 | 95.48 | 162.44 | 81.18 | 74.61 |
| P63027 | Vesicle-associated membrane protein 2 | **0.66** | **0.24** | 93.53 | 39.50 | 61.82 | 42.49 | 123.14 | 144.96 | 81.30 | 69.16 | 49.07 | 0.00 | 129.42 | 78.39 | 43.71 | 63.25 | 56.11 |
| P06239 | Tyrosine-protein kinase Lck | **2.09** | **0.24** | 17.36 | 18.32 | 36.32 | 28.73 | 20.24 | 0.00 | 43.87 | 0.00 | 22.69 | 53.83 | 45.01 | 0.00 | 0.00 | 55.59 | 63.46 |
| P11766 | Alcohol dehydrogenase class-3 | **2.04** | **0.24** | 44.74 | 41.76 | 91.28 | 72.06 | 82.21 | 0.00 | 60.35 | 81.11 | 0.00 | 48.77 | 75.22 | 108.66 | 0.00 | 214.48 | 100.51 |
| Q15650 | Activating signal cointegrator 1 | **1.77** | **0.24** | 865.70 | 942.49 | 1528.78 | 792.56 | 1090.83 | 2278.08 | 0.00 | 959.58 | 0.00 | 2932.57 | 1960.23 | 1159.84 | 851.94 | 936.42 | 1331.67 |
| P03989 | HLA class I histocompatibility antigen, B-27 alpha chain | **1.92** | **0.24** | 137.05 | 80.77 | 263.66 | 220.71 | 222.13 | 213.64 | 129.52 | 82.94 | 37.01 | 32.62 | 446.31 | 560.70 | 349.39 | 148.09 | 44.85 |
| Q9UNH7 | Sorting nexin-6 | **5.83** | **0.24** | 4.99 | 11.16 | 29.08 | 40.96 | 0.00 | 0.00 | 24.95 | 0.00 | 0.00 | 13.49 | 27.42 | 0.00 | 24.33 | 0.00 | 109.26 |
| P46926 | Glucosamine-6-phosphate isomerase 1 | **0.37** | **0.24** | 28.57 | 30.12 | 10.57 | 16.45 | 65.14 | 0.00 | 23.89 | 53.81 | 0.00 | 0.00 | 0.00 | 0.00 | 0.00 | 29.21 | 34.20 |
| P07738 | Bisphosphoglycerate mutase | **0.33** | **0.24** | 12.98 | 12.47 | 4.27 | 10.45 | 26.25 | 0.00 | 0.00 | 15.54 | 23.11 | 0.00 | 0.00 | 0.00 | 0.00 | 25.60 | 0.00 |
| Q96JB5 | CDK5 regulatory subunit-associated protein 3 | **0.31** | **0.24** | 7.44 | 7.97 | 2.28 | 5.58 | 7.96 | 0.00 | 10.18 | 19.08 | 0.00 | 0.00 | 0.00 | 0.00 | 0.00 | 0.00 | 13.66 |
| P05155 | Plasma protease C1 inhibitor | **2.17** | **0.24** | 43.14 | 42.29 | 93.58 | 79.94 | 87.09 | 89.52 | 12.54 | 26.55 | 0.00 | 51.57 | 30.81 | 152.36 | 229.54 | 60.04 | 37.16 |
| P30461 | HLA class I histocompatibility antigen, B-13 alpha chain | **1.89** | **0.24** | 139.87 | 76.54 | 264.68 | 219.44 | 222.13 | 213.64 | 129.52 | 82.94 | 51.12 | 38.73 | 446.31 | 560.70 | 349.39 | 148.09 | 44.85 |
| P30485 | HLA class I histocompatibility antigen, B-47 alpha chain | **1.89** | **0.24** | 139.87 | 76.54 | 264.68 | 219.44 | 222.13 | 213.64 | 129.52 | 82.94 | 51.12 | 38.73 | 446.31 | 560.70 | 349.39 | 148.09 | 44.85 |
| P21796 | Voltage-dependent anion-selective channel protein 1 | **0.66** | **0.24** | 342.34 | 175.71 | 226.50 | 128.40 | 458.97 | 182.01 | 570.50 | 337.07 | 163.17 | 432.78 | 159.73 | 106.15 | 105.47 | 254.03 | 300.85 |
| P02746 | Complement C1q subcomponent subunit B | **2.00** | **0.24** | 18.32 | 27.70 | 36.66 | 20.56 | 0.00 | 62.41 | 29.19 | 0.00 | 0.00 | 35.06 | 42.00 | 57.95 | 52.89 | 32.06 | 0.00 |
| P05997 | Collagen alpha-2(V) chain | **1.64** | **0.24** | 153.56 | 179.33 | 251.84 | 63.98 | 87.93 | 461.50 | 77.67 | 140.72 | 0.00 | 172.75 | 288.22 | 178.70 | 288.53 | 252.29 | 330.53 |
| P27816 | Microtubule-associated protein 4 | **0.48** | **0.24** | 45.31 | 37.14 | 21.94 | 24.13 | 94.78 | 64.57 | 47.23 | 19.96 | 0.00 | 40.80 | 0.00 | 0.00 | 47.43 | 43.43 | 0.00 |
| P00915 | Carbonic anhydrase 1 | **0.31** | **0.24** | 752.81 | 838.90 | 233.57 | 101.25 | 441.85 | 257.55 | 661.97 | 185.85 | 2216.83 | 420.77 | 172.30 | 226.32 | 252.95 | 199.88 | 129.20 |
| Q5VTH9 | WD repeat-containing protein 78 | **1.41** | **0.24** | 214.17 | 140.12 | 302.53 | 92.15 | 377.64 | 263.70 | 0.00 | 256.51 | 173.00 | 437.02 | 371.47 | 276.22 | 174.90 | 251.19 | 304.35 |
| Q9H4A4 | Aminopeptidase B | **14.17** | **0.24** | 24.04 | 24.24 | 340.52 | 558.15 | 31.14 | 0.00 | 32.17 | 56.87 | 0.00 | 0.00 | 185.99 | 1453.21 | 320.42 | 21.53 | 61.97 |
| P16615 | Sarcoplasmic/endoplasmic reticulum calcium ATPase 2 | **1.75** | **0.24** | 45.71 | 45.69 | 80.11 | 45.08 | 0.00 | 0.00 | 74.22 | 103.43 | 50.88 | 85.42 | 81.47 | 48.14 | 33.02 | 70.12 | 162.51 |
| P14324 | Farnesyl pyrophosphate synthase | **2.62** | **0.24** | 12.39 | 27.71 | 32.45 | 25.53 | 0.00 | 0.00 | 0.00 | 61.95 | 0.00 | 40.94 | 52.64 | 0.00 | 0.00 | 47.69 | 53.45 |
| P84243 | Histone H3.3 | **1.38** | **0.24** | 2380.95 | 1260.65 | 3286.74 | 1144.44 | 1446.47 | 1458.21 | 3524.61 | 3976.61 | 1498.85 | 2333.14 | 3067.08 | 3292.09 | 2124.63 | 3578.26 | 5325.27 |
| P02647 | Apolipoprotein A-I | **0.49** | **0.24** | 1343.67 | 1295.41 | 660.25 | 355.48 | 1486.48 | 3533.92 | 683.73 | 334.46 | 679.76 | 541.08 | 669.09 | 988.47 | 1125.86 | 490.91 | 146.09 |
| Q9Y5Z4 | Heme-binding protein 2 | **0.55** | **0.24** | 50.85 | 32.87 | 28.04 | 27.94 | 82.71 | 0.00 | 53.87 | 41.72 | 75.93 | 47.79 | 24.77 | 0.00 | 23.88 | 0.00 | 71.78 |
| O60814 | Histone H2B type 1-K | **1.38** | **0.24** | 4532.19 | 2447.06 | 6236.82 | 2094.56 | 3156.79 | 2907.04 | 7218.89 | 7154.07 | 2224.16 | 6942.72 | 6978.44 | 4186.15 | 3393.60 | 6806.87 | 9113.18 |
| P57053 | Histone H2B type F-S | **1.38** | **0.24** | 4532.19 | 2447.06 | 6236.82 | 2094.56 | 3156.79 | 2907.04 | 7218.89 | 7154.07 | 2224.16 | 6942.72 | 6978.44 | 4186.15 | 3393.60 | 6806.87 | 9113.18 |
| P58876 | Histone H2B type 1-D | **1.38** | **0.24** | 4532.19 | 2447.06 | 6236.82 | 2094.56 | 3156.79 | 2907.04 | 7218.89 | 7154.07 | 2224.16 | 6942.72 | 6978.44 | 4186.15 | 3393.60 | 6806.87 | 9113.18 |
| P62807 | Histone H2B type 1-C/E/F/G/I | **1.38** | **0.24** | 4532.19 | 2447.06 | 6236.82 | 2094.56 | 3156.79 | 2907.04 | 7218.89 | 7154.07 | 2224.16 | 6942.72 | 6978.44 | 4186.15 | 3393.60 | 6806.87 | 9113.18 |
| Q5QNW6 | Histone H2B type 2-F | **1.38** | **0.24** | 4532.19 | 2447.06 | 6236.82 | 2094.56 | 3156.79 | 2907.04 | 7218.89 | 7154.07 | 2224.16 | 6942.72 | 6978.44 | 4186.15 | 3393.60 | 6806.87 | 9113.18 |
| Q93079 | Histone H2B type 1-H | **1.38** | **0.24** | 4532.19 | 2447.06 | 6236.82 | 2094.56 | 3156.79 | 2907.04 | 7218.89 | 7154.07 | 2224.16 | 6942.72 | 6978.44 | 4186.15 | 3393.60 | 6806.87 | 9113.18 |
| Q99877 | Histone H2B type 1-N | **1.38** | **0.24** | 4532.19 | 2447.06 | 6236.82 | 2094.56 | 3156.79 | 2907.04 | 7218.89 | 7154.07 | 2224.16 | 6942.72 | 6978.44 | 4186.15 | 3393.60 | 6806.87 | 9113.18 |
| Q99879 | Histone H2B type 1-M | **1.38** | **0.24** | 4532.19 | 2447.06 | 6236.82 | 2094.56 | 3156.79 | 2907.04 | 7218.89 | 7154.07 | 2224.16 | 6942.72 | 6978.44 | 4186.15 | 3393.60 | 6806.87 | 9113.18 |
| Q99880 | Histone H2B type 1-L | **1.38** | **0.24** | 4532.19 | 2447.06 | 6236.82 | 2094.56 | 3156.79 | 2907.04 | 7218.89 | 7154.07 | 2224.16 | 6942.72 | 6978.44 | 4186.15 | 3393.60 | 6806.87 | 9113.18 |
| P62820 | Ras-related protein Rab-1A | **1.32** | **0.24** | 291.49 | 73.91 | 383.84 | 150.36 | 277.88 | 222.77 | 297.34 | 413.35 | 246.13 | 616.17 | 336.35 | 366.11 | 210.07 | 272.55 | 501.80 |
| Q92928 | Putative Ras-related protein Rab-1C | **1.32** | **0.24** | 291.49 | 73.91 | 383.84 | 150.36 | 277.88 | 222.77 | 297.34 | 413.35 | 246.13 | 616.17 | 336.35 | 366.11 | 210.07 | 272.55 | 501.80 |
| Q9H0U4 | Ras-related protein Rab-1B | **1.32** | **0.24** | 291.49 | 73.91 | 383.84 | 150.36 | 277.88 | 222.77 | 297.34 | 413.35 | 246.13 | 616.17 | 336.35 | 366.11 | 210.07 | 272.55 | 501.80 |
| P30740 | Leukocyte elastase inhibitor | **3.51** | **0.25** | 6.65 | 14.86 | 23.36 | 26.63 | 33.23 | 0.00 | 0.00 | 0.00 | 0.00 | 19.72 | 70.80 | 0.00 | 0.00 | 34.43 | 15.19 |
| Q8TE77 | Protein phosphatase Slingshot homolog 3 | **0.45** | **0.25** | 49.45 | 37.42 | 22.14 | 35.30 | 81.60 | 0.00 | 88.40 | 52.25 | 25.00 | 53.25 | 0.00 | 0.00 | 0.00 | 0.00 | 79.60 |
| P30475 | HLA class I histocompatibility antigen, B-39 alpha chain | **1.84** | **0.25** | 137.05 | 80.77 | 252.17 | 203.26 | 222.13 | 213.64 | 129.52 | 82.94 | 37.01 | 32.62 | 446.31 | 491.78 | 349.39 | 148.09 | 44.85 |
| Q29718 | HLA class I histocompatibility antigen, B-82 alpha chain | **1.84** | **0.25** | 137.05 | 80.77 | 252.17 | 203.26 | 222.13 | 213.64 | 129.52 | 82.94 | 37.01 | 32.62 | 446.31 | 491.78 | 349.39 | 148.09 | 44.85 |
| Q29836 | HLA class I histocompatibility antigen, B-67 alpha chain | **1.84** | **0.25** | 137.05 | 80.77 | 252.17 | 203.26 | 222.13 | 213.64 | 129.52 | 82.94 | 37.01 | 32.62 | 446.31 | 491.78 | 349.39 | 148.09 | 44.85 |
| Q95365 | HLA class I histocompatibility antigen, B-38 alpha chain | **1.84** | **0.25** | 137.05 | 80.77 | 252.17 | 203.26 | 222.13 | 213.64 | 129.52 | 82.94 | 37.01 | 32.62 | 446.31 | 491.78 | 349.39 | 148.09 | 44.85 |
| P61247 | 40S ribosomal protein S3a | **1.41** | **0.25** | 157.64 | 93.44 | 222.81 | 80.58 | 138.01 | 63.58 | 234.08 | 274.13 | 78.40 | 191.11 | 238.90 | 195.31 | 119.08 | 229.51 | 362.96 |
| P04040 | Catalase | **0.67** | **0.25** | 154.69 | 84.13 | 103.02 | 20.10 | 223.85 | 97.00 | 92.79 | 93.01 | 266.78 | 80.75 | 128.68 | 124.51 | 101.87 | 98.98 | 83.34 |
| P31483 | Nucleolysin TIA-1 isoform p40 | **0.31** | **0.25** | 14.65 | 16.08 | 4.50 | 11.03 | 14.49 | 0.00 | 20.13 | 38.63 | 0.00 | 0.00 | 0.00 | 0.00 | 0.00 | 27.02 | 0.00 |
| P25398 | 40S ribosomal protein S12 | **0.63** | **0.25** | 82.29 | 46.47 | 51.81 | 35.09 | 102.00 | 101.29 | 113.16 | 95.03 | 0.00 | 86.67 | 35.51 | 0.00 | 49.68 | 43.41 | 95.60 |
| P63104 | 14-3-3 protein zeta/delta | **1.23** | **0.25** | 1318.98 | 327.22 | 1620.64 | 451.96 | 1533.88 | 781.90 | 1505.27 | 1544.57 | 1229.27 | 1677.48 | 1513.22 | 1401.00 | 1002.22 | 1764.06 | 2365.84 |
| Q8TD47 | 40S ribosomal protein S4, Y isoform 2 | **1.73** | **0.25** | 74.61 | 73.37 | 129.03 | 72.04 | 79.91 | 0.00 | 144.19 | 148.97 | 0.00 | 176.48 | 210.86 | 116.68 | 0.00 | 127.06 | 143.09 |
| P04792 | Heat shock protein beta-1 | **2.16** | **0.25** | 1649.53 | 672.74 | 3566.90 | 3544.07 | 2756.18 | 1191.81 | 1397.83 | 1108.95 | 1792.89 | 3433.21 | 5426.45 | 970.04 | 482.69 | 1346.39 | 9742.63 |
| P30483 | HLA class I histocompatibility antigen, B-45 alpha chain | **1.81** | **0.25** | 139.87 | 76.54 | 253.19 | 201.95 | 222.13 | 213.64 | 129.52 | 82.94 | 51.12 | 38.73 | 446.31 | 491.78 | 349.39 | 148.09 | 44.85 |
| P30487 | HLA class I histocompatibility antigen, B-49 alpha chain | **1.81** | **0.25** | 139.87 | 76.54 | 253.19 | 201.95 | 222.13 | 213.64 | 129.52 | 82.94 | 51.12 | 38.73 | 446.31 | 491.78 | 349.39 | 148.09 | 44.85 |
| P30488 | HLA class I histocompatibility antigen, B-50 alpha chain | **1.81** | **0.25** | 139.87 | 76.54 | 253.19 | 201.95 | 222.13 | 213.64 | 129.52 | 82.94 | 51.12 | 38.73 | 446.31 | 491.78 | 349.39 | 148.09 | 44.85 |
| P61160 | Actin-related protein 2 | **1.45** | **0.25** | 85.60 | 53.80 | 124.12 | 49.41 | 106.28 | 24.16 | 134.82 | 130.95 | 31.79 | 86.42 | 217.03 | 114.77 | 83.40 | 135.58 | 107.55 |
| P12235 | ADP/ATP translocase 1 | **1.53** | **0.25** | 202.08 | 119.95 | 308.52 | 157.74 | 211.27 | 0.00 | 226.55 | 316.66 | 255.93 | 224.13 | 591.47 | 273.80 | 121.06 | 337.69 | 302.96 |
| P07437 | Tubulin beta chain | **1.36** | **0.25** | 802.20 | 405.16 | 1094.81 | 378.92 | 814.96 | 305.82 | 915.56 | 1392.70 | 581.96 | 812.99 | 1281.08 | 744.06 | 744.72 | 1636.35 | 1349.65 |
| Q86YW9 | Mediator of RNA polymerase II transcription subunit 12-like protein | **0.34** | **0.25** | 142.80 | 132.80 | 48.97 | 119.94 | 208.37 | 0.00 | 277.86 | 227.78 | 0.00 | 0.00 | 0.00 | 0.00 | 0.00 | 0.00 | 293.80 |
| Q93074 | Mediator of RNA polymerase II transcription subunit 12 | **0.34** | **0.25** | 142.80 | 132.80 | 48.97 | 119.94 | 208.37 | 0.00 | 277.86 | 227.78 | 0.00 | 0.00 | 0.00 | 0.00 | 0.00 | 0.00 | 293.80 |
| Q9Y4L1 | Hypoxia up-regulated protein 1 | **1.62** | **0.25** | 34.97 | 23.11 | 56.70 | 33.13 | 49.96 | 0.00 | 32.79 | 60.86 | 31.25 | 75.51 | 87.83 | 34.92 | 76.85 | 65.07 | 0.00 |
| P61019 | Ras-related protein Rab-2A | **1.41** | **0.25** | 59.97 | 38.19 | 84.77 | 28.87 | 84.21 | 0.00 | 74.45 | 95.23 | 45.95 | 99.81 | 114.11 | 59.75 | 42.81 | 81.28 | 110.87 |
| Q92945 | Far upstream element-binding protein 2 | **0.77** | **0.25** | 93.95 | 32.87 | 71.97 | 26.57 | 147.05 | 96.97 | 87.84 | 79.12 | 58.80 | 105.97 | 40.71 | 76.40 | 45.29 | 97.15 | 66.29 |
| Q13813 | Spectrin alpha chain, non-erythrocytic 1 | **0.59** | **0.25** | 100.30 | 66.55 | 59.59 | 21.49 | 201.94 | 126.69 | 50.91 | 85.03 | 36.95 | 54.43 | 72.76 | 68.10 | 18.11 | 70.59 | 73.56 |
| Q6KB66 | Keratin, type II cytoskeletal 80 | **0.05** | **0.25** | 45.85 | 72.51 | 2.37 | 5.80 | 172.88 | 0.00 | 22.45 | 33.90 | 0.00 | 0.00 | 0.00 | 0.00 | 0.00 | 14.21 | 0.00 |
| P78386 | Keratin, type II cuticular Hb5 | **0.58** | **0.25** | 388.17 | 236.53 | 226.85 | 199.87 | 381.22 | 501.40 | 0.00 | 630.40 | 427.82 | 222.23 | 180.47 | 594.48 | 0.00 | 240.80 | 123.11 |
| P00167 | Cytochrome b5 | **0.36** | **0.25** | 248.97 | 300.51 | 90.62 | 100.01 | 745.35 | 126.43 | 309.09 | 63.98 | 0.00 | 187.10 | 159.76 | 0.00 | 0.00 | 0.00 | 196.84 |
| Q9UNM6 | 26S proteasome non-ATPase regulatory subunit 13 | **2.64** | **0.25** | 15.70 | 17.51 | 41.40 | 43.88 | 20.04 | 0.00 | 0.00 | 42.43 | 16.04 | 0.00 | 56.32 | 114.62 | 0.00 | 21.39 | 56.10 |
| P30464 | HLA class I histocompatibility antigen, B-15 alpha chain | **1.78** | **0.25** | 162.39 | 123.64 | 288.63 | 200.46 | 349.87 | 213.64 | 129.52 | 82.94 | 35.97 | 32.62 | 446.31 | 491.78 | 349.39 | 366.82 | 44.85 |
| P30484 | HLA class I histocompatibility antigen, B-46 alpha chain | **1.78** | **0.25** | 162.39 | 123.64 | 288.63 | 200.46 | 349.87 | 213.64 | 129.52 | 82.94 | 35.97 | 32.62 | 446.31 | 491.78 | 349.39 | 366.82 | 44.85 |
| P30495 | HLA class I histocompatibility antigen, B-56 alpha chain | **1.78** | **0.25** | 162.39 | 123.64 | 288.63 | 200.46 | 349.87 | 213.64 | 129.52 | 82.94 | 35.97 | 32.62 | 446.31 | 491.78 | 349.39 | 366.82 | 44.85 |
| P61106 | Ras-related protein Rab-14 | **1.31** | **0.25** | 201.77 | 49.11 | 264.50 | 111.20 | 159.24 | 275.99 | 188.51 | 224.06 | 161.07 | 406.74 | 316.62 | 224.07 | 134.59 | 152.29 | 352.71 |
| P61026 | Ras-related protein Rab-10 | **1.30** | **0.25** | 308.13 | 70.53 | 399.30 | 161.87 | 259.42 | 222.77 | 365.52 | 391.22 | 301.74 | 585.94 | 551.67 | 249.45 | 207.85 | 317.25 | 483.67 |
| P01889 | HLA class I histocompatibility antigen, B-7 alpha chain | **1.85** | **0.25** | 143.04 | 88.61 | 265.14 | 219.81 | 222.13 | 243.59 | 129.52 | 82.94 | 37.01 | 32.62 | 446.31 | 560.70 | 349.39 | 156.96 | 44.85 |
| P25815 | Protein S100-P | **0.42** | **0.25** | 552.61 | 523.92 | 232.14 | 346.01 | 588.32 | 368.08 | 176.31 | 189.38 | 1440.93 | 914.47 | 56.81 | 72.49 | 0.00 | 84.30 | 264.78 |
| Q9Y295 | Developmentally-regulated GTP-binding protein 1 | **2.71** | **0.26** | 3.68 | 8.22 | 9.98 | 8.82 | 0.00 | 0.00 | 0.00 | 18.39 | 0.00 | 0.00 | 18.36 | 0.00 | 8.14 | 13.04 | 20.32 |
| Q9BW60 | Elongation of very long chain fatty acids protein 1 | **2.99** | **0.26** | 7.36 | 16.46 | 21.99 | 22.31 | 0.00 | 0.00 | 0.00 | 0.00 | 36.81 | 37.49 | 20.87 | 0.00 | 0.00 | 16.28 | 57.31 |
| Q04826 | HLA class I histocompatibility antigen, B-40 alpha chain | **1.82** | **0.26** | 145.86 | 84.51 | 266.16 | 218.52 | 222.13 | 243.59 | 129.52 | 82.94 | 51.12 | 38.73 | 446.31 | 560.70 | 349.39 | 156.96 | 44.85 |
| P62424 | 60S ribosomal protein L7a | **1.42** | **0.26** | 142.24 | 102.79 | 202.65 | 61.07 | 92.29 | 43.25 | 207.54 | 289.44 | 78.69 | 168.81 | 269.42 | 177.96 | 119.13 | 204.04 | 276.54 |
| P35659 | Protein DEK | **2.41** | **0.26** | 15.05 | 21.29 | 36.21 | 33.71 | 0.00 | 0.00 | 30.07 | 45.17 | 0.00 | 38.58 | 39.45 | 0.00 | 0.00 | 49.47 | 89.75 |
| O15400 | Syntaxin-7 | **0.44** | **0.26** | 27.79 | 23.75 | 12.24 | 19.18 | 64.51 | 0.00 | 29.36 | 16.33 | 28.75 | 41.19 | 32.27 | 0.00 | 0.00 | 0.00 | 0.00 |
| Q8TB22 | Spermatogenesis-associated protein 20 | **0.35** | **0.26** | 73.71 | 84.85 | 26.08 | 44.28 | 77.63 | 0.00 | 0.00 | 207.18 | 83.77 | 0.00 | 0.00 | 106.92 | 0.00 | 0.00 | 49.54 |
| P51571 | Translocon-associated protein subunit delta | **8.44** | **0.26** | 39.52 | 33.72 | 333.51 | 542.52 | 22.94 | 0.00 | 85.46 | 61.15 | 28.02 | 206.23 | 82.21 | 1436.20 | 124.73 | 76.21 | 75.47 |
| P82979 | SAP domain-containing ribonucleoprotein | **0.58** | **0.26** | 39.00 | 16.48 | 22.78 | 26.05 | 64.91 | 30.77 | 37.35 | 41.34 | 20.61 | 57.15 | 33.54 | 0.00 | 0.00 | 0.00 | 45.99 |
| O43432 | Eukaryotic translation initiation factor 4 gamma 3 | **5.77** | **0.26** | 7.57 | 11.06 | 43.70 | 66.12 | 0.00 | 0.00 | 13.49 | 24.37 | 0.00 | 16.39 | 49.08 | 0.00 | 23.24 | 173.50 | 0.00 |
| Q6UXL0 | Interleukin-20 receptor subunit beta | **0.22** | **0.26** | 1807.49 | 2818.96 | 402.70 | 632.36 | 1603.14 | 6710.21 | 0.00 | 724.12 | 0.00 | 0.00 | 116.65 | 753.03 | 0.00 | 1546.53 | 0.00 |
| Q14683 | Structural maintenance of chromosomes protein 1A | **1.50** | **0.26** | 82.09 | 55.94 | 123.27 | 57.60 | 84.53 | 0.00 | 117.45 | 146.31 | 62.16 | 179.91 | 58.43 | 94.71 | 64.88 | 156.29 | 185.39 |
| P01891 | HLA class I histocompatibility antigen, A-68 alpha chain | **1.76** | **0.26** | 98.36 | 62.17 | 173.40 | 127.15 | 185.71 | 103.90 | 122.25 | 54.32 | 25.64 | 46.44 | 303.48 | 175.70 | 104.09 | 348.43 | 62.27 |
| P16190 | HLA class I histocompatibility antigen, A-33 alpha chain | **1.76** | **0.26** | 98.36 | 62.17 | 173.40 | 127.15 | 185.71 | 103.90 | 122.25 | 54.32 | 25.64 | 46.44 | 303.48 | 175.70 | 104.09 | 348.43 | 62.27 |
| P30453 | HLA class I histocompatibility antigen, A-34 alpha chain | **1.76** | **0.26** | 98.36 | 62.17 | 173.40 | 127.15 | 185.71 | 103.90 | 122.25 | 54.32 | 25.64 | 46.44 | 303.48 | 175.70 | 104.09 | 348.43 | 62.27 |
| P30481 | HLA class I histocompatibility antigen, B-44 alpha chain | **1.74** | **0.26** | 139.75 | 76.40 | 243.74 | 190.94 | 222.13 | 213.04 | 129.52 | 82.94 | 51.12 | 38.73 | 422.35 | 491.78 | 291.63 | 173.09 | 44.85 |
| P30456 | HLA class I histocompatibility antigen, A-43 alpha chain | **1.53** | **0.26** | 143.10 | 93.69 | 219.54 | 113.94 | 264.50 | 197.39 | 150.40 | 69.28 | 33.91 | 79.99 | 303.48 | 311.46 | 227.22 | 317.78 | 77.29 |
| P62241 | 40S ribosomal protein S8 | **1.50** | **0.26** | 119.93 | 72.99 | 180.31 | 91.40 | 69.96 | 52.57 | 179.61 | 216.05 | 81.49 | 192.04 | 236.46 | 93.83 | 85.69 | 148.49 | 325.35 |
| P55072 | Transitional endoplasmic reticulum ATPase | **1.36** | **0.26** | 121.97 | 49.18 | 165.56 | 68.14 | 99.47 | 63.65 | 172.44 | 174.30 | 99.97 | 113.10 | 224.04 | 139.99 | 80.41 | 175.18 | 260.66 |
| P22392 | Nucleoside diphosphate kinase B | **1.30** | **0.26** | 434.72 | 181.90 | 563.32 | 175.00 | 434.73 | 158.87 | 572.18 | 621.77 | 386.06 | 763.18 | 746.77 | 418.36 | 325.94 | 595.77 | 529.94 |
| O95626 | Acidic leucine-rich nuclear phosphoprotein 32 family member D | **0.30** | **0.27** | 24.13 | 29.33 | 7.19 | 17.61 | 70.70 | 0.00 | 0.00 | 32.24 | 17.73 | 0.00 | 0.00 | 0.00 | 0.00 | 0.00 | 43.12 |
| P12931 | Proto-oncogene tyrosine-protein kinase Src | **0.47** | **0.27** | 26.67 | 20.45 | 12.44 | 19.33 | 20.24 | 0.00 | 38.42 | 54.00 | 20.71 | 0.00 | 34.80 | 0.00 | 0.00 | 39.82 | 0.00 |
| Q6UXV0 | GDNF family receptor alpha-like | **0.40** | **0.27** | 444.67 | 416.91 | 175.85 | 338.44 | 0.00 | 0.00 | 829.26 | 807.67 | 586.42 | 0.00 | 845.05 | 0.00 | 0.00 | 210.07 | 0.00 |
| P17661 | Desmin | **1.74** | **0.27** | 680.85 | 503.91 | 1186.80 | 834.09 | 552.80 | 1451.01 | 491.74 | 88.45 | 820.26 | 738.73 | 859.70 | 2423.96 | 1971.13 | 916.08 | 211.23 |
| P07951 | Tropomyosin beta chain | **2.21** | **0.27** | 601.85 | 498.81 | 1332.12 | 1296.89 | 1267.04 | 1000.30 | 272.32 | 324.17 | 145.43 | 377.94 | 1098.43 | 3640.65 | 1969.60 | 738.80 | 167.30 |
| P04908 | Histone H2A type 1-B/E | **1.14** | **0.27** | 7219.06 | 1894.98 | 8236.12 | 881.55 | 6769.32 | 6376.64 | 9565.59 | 8614.09 | 4769.65 | 8549.35 | 9177.85 | 8690.34 | 6676.32 | 7807.67 | 8515.17 |
| P0C0S8 | Histone H2A type 1 | **1.14** | **0.27** | 7219.06 | 1894.98 | 8236.12 | 881.55 | 6769.32 | 6376.64 | 9565.59 | 8614.09 | 4769.65 | 8549.35 | 9177.85 | 8690.34 | 6676.32 | 7807.67 | 8515.17 |
| P20671 | Histone H2A type 1-D | **1.14** | **0.27** | 7219.06 | 1894.98 | 8236.12 | 881.55 | 6769.32 | 6376.64 | 9565.59 | 8614.09 | 4769.65 | 8549.35 | 9177.85 | 8690.34 | 6676.32 | 7807.67 | 8515.17 |
| Q16777 | Histone H2A type 2-C | **1.14** | **0.27** | 7219.06 | 1894.98 | 8236.12 | 881.55 | 6769.32 | 6376.64 | 9565.59 | 8614.09 | 4769.65 | 8549.35 | 9177.85 | 8690.34 | 6676.32 | 7807.67 | 8515.17 |
| Q6FI13 | Histone H2A type 2-A | **1.14** | **0.27** | 7219.06 | 1894.98 | 8236.12 | 881.55 | 6769.32 | 6376.64 | 9565.59 | 8614.09 | 4769.65 | 8549.35 | 9177.85 | 8690.34 | 6676.32 | 7807.67 | 8515.17 |
| Q7L7L0 | Histone H2A type 3 | **1.14** | **0.27** | 7219.06 | 1894.98 | 8236.12 | 881.55 | 6769.32 | 6376.64 | 9565.59 | 8614.09 | 4769.65 | 8549.35 | 9177.85 | 8690.34 | 6676.32 | 7807.67 | 8515.17 |
| Q93077 | Histone H2A type 1-C | **1.14** | **0.27** | 7219.06 | 1894.98 | 8236.12 | 881.55 | 6769.32 | 6376.64 | 9565.59 | 8614.09 | 4769.65 | 8549.35 | 9177.85 | 8690.34 | 6676.32 | 7807.67 | 8515.17 |
| Q96KK5 | Histone H2A type 1-H | **1.14** | **0.27** | 7219.06 | 1894.98 | 8236.12 | 881.55 | 6769.32 | 6376.64 | 9565.59 | 8614.09 | 4769.65 | 8549.35 | 9177.85 | 8690.34 | 6676.32 | 7807.67 | 8515.17 |
| Q99878 | Histone H2A type 1-J | **1.14** | **0.27** | 7219.06 | 1894.98 | 8236.12 | 881.55 | 6769.32 | 6376.64 | 9565.59 | 8614.09 | 4769.65 | 8549.35 | 9177.85 | 8690.34 | 6676.32 | 7807.67 | 8515.17 |
| Q9BTM1 | Histone H2A.J | **1.14** | **0.27** | 7219.06 | 1894.98 | 8236.12 | 881.55 | 6769.32 | 6376.64 | 9565.59 | 8614.09 | 4769.65 | 8549.35 | 9177.85 | 8690.34 | 6676.32 | 7807.67 | 8515.17 |
| Q9UJS0 | Calcium-binding mitochondrial carrier protein Aralar2 | **2.36** | **0.27** | 6.99 | 10.23 | 16.47 | 15.29 | 0.00 | 0.00 | 22.61 | 12.32 | 0.00 | 28.22 | 33.77 | 0.00 | 0.00 | 8.87 | 27.93 |
| Q9H361 | Polyadenylate-binding protein 3 | **1.70** | **0.27** | 47.45 | 44.17 | 80.50 | 48.01 | 73.65 | 0.00 | 70.51 | 93.10 | 0.00 | 95.47 | 95.96 | 0.00 | 48.05 | 129.68 | 113.84 |
| Q8IWJ2 | GRIP and coiled-coil domain-containing protein 2 | **0.26** | **0.27** | 564.59 | 840.19 | 145.83 | 234.94 | 2011.57 | 0.00 | 550.44 | 260.92 | 0.00 | 539.44 | 0.00 | 0.00 | 0.00 | 335.56 | 0.00 |
| P31949 | Protein S100-A11 | **0.67** | **0.27** | 2529.98 | 1329.95 | 1707.71 | 988.63 | 4610.23 | 1963.80 | 1135.89 | 2958.04 | 1981.95 | 2062.25 | 3296.57 | 766.80 | 554.44 | 1854.05 | 1712.12 |
| P02747 | Complement C1q subcomponent subunit C | **2.41** | **0.27** | 33.72 | 47.17 | 81.29 | 79.04 | 0.00 | 97.99 | 70.60 | 0.00 | 0.00 | 49.06 | 53.33 | 228.42 | 102.39 | 54.57 | 0.00 |
| Q15046 | Lysine--tRNA ligase | **2.07** | **0.27** | 11.35 | 15.59 | 23.50 | 18.18 | 0.00 | 0.00 | 26.72 | 30.03 | 0.00 | 14.38 | 51.70 | 0.00 | 14.80 | 35.53 | 24.57 |
| P45880 | Voltage-dependent anion-selective channel protein 2 | **0.72** | **0.27** | 146.85 | 71.73 | 105.10 | 45.41 | 136.90 | 65.23 | 255.13 | 169.77 | 107.20 | 178.36 | 69.84 | 63.34 | 83.40 | 141.91 | 93.76 |
| Q5CZ79 | Ankyrin repeat domain-containing protein 20B | **2.18** | **0.27** | 33.59 | 46.85 | 73.12 | 61.83 | 71.41 | 0.00 | 0.00 | 96.54 | 0.00 | 110.36 | 0.00 | 0.00 | 65.46 | 122.18 | 140.73 |
| O75694 | Nuclear pore complex protein Nup155 | **1.59** | **0.27** | 1979.24 | 1374.54 | 3150.23 | 1839.73 | 2945.95 | 0.00 | 3267.26 | 2551.60 | 1131.37 | 4687.04 | 4026.88 | 0.00 | 3460.45 | 4720.27 | 2006.72 |
| Q05193 | Dynamin-1 | **0.59** | **0.27** | 33.01 | 19.24 | 19.52 | 18.80 | 38.74 | 0.00 | 43.52 | 34.10 | 48.69 | 0.00 | 49.07 | 22.80 | 15.51 | 0.00 | 29.77 |
| P68431 | Histone H3.1 | **1.37** | **0.27** | 2432.15 | 1364.19 | 3324.44 | 1164.00 | 1420.01 | 1411.64 | 3835.05 | 4014.25 | 1479.79 | 2339.94 | 3222.15 | 3292.09 | 2116.02 | 3586.89 | 5389.52 |
| Q6P2Q9 | Pre-mRNA-processing-splicing factor 8 | **2.14** | **0.27** | 7.18 | 10.39 | 15.37 | 12.39 | 0.00 | 0.00 | 0.00 | 22.71 | 13.18 | 20.33 | 24.27 | 0.00 | 0.00 | 18.83 | 28.76 |
| P12109 | Collagen alpha-1(VI) chain | **1.78** | **0.27** | 495.81 | 422.06 | 882.77 | 628.03 | 389.18 | 808.23 | 1052.85 | 148.22 | 80.54 | 1316.48 | 624.61 | 1136.33 | 386.04 | 1754.43 | 78.71 |
| Q9Y5U9 | Immediate early response 3-interacting protein 1 | **2.19** | **0.27** | 11.50 | 15.88 | 25.13 | 21.62 | 25.85 | 0.00 | 0.00 | 31.64 | 0.00 | 36.69 | 52.63 | 22.99 | 0.00 | 0.00 | 38.49 |
| O96000 | NADH dehydrogenase [ubiquinone] 1 beta subcomplex subunit 10 | **1.82** | **0.27** | 21.50 | 20.51 | 39.05 | 27.83 | 40.45 | 0.00 | 40.93 | 26.12 | 0.00 | 20.45 | 90.50 | 17.56 | 27.26 | 51.08 | 27.45 |
| P30499 | HLA class I histocompatibility antigen, Cw-1 alpha chain | **1.56** | **0.27** | 46.29 | 32.82 | 72.35 | 39.85 | 28.12 | 102.99 | 40.83 | 39.36 | 20.13 | 28.26 | 51.85 | 133.53 | 80.65 | 99.50 | 40.31 |
| O75874 | Isocitrate dehydrogenase [NADP] cytoplasmic | **0.54** | **0.27** | 381.93 | 303.66 | 205.40 | 114.69 | 841.60 | 63.35 | 249.88 | 238.53 | 516.30 | 374.47 | 227.25 | 67.10 | 90.11 | 270.86 | 202.61 |
| P17655 | Calpain-2 catalytic subunit | **1.79** | **0.28** | 17.13 | 15.77 | 30.64 | 21.55 | 26.93 | 0.00 | 26.91 | 31.83 | 0.00 | 9.03 | 21.72 | 59.54 | 55.30 | 24.90 | 13.35 |
| P06737 | Glycogen phosphorylase, liver form | **1.77** | **0.28** | 29.01 | 33.68 | 51.33 | 30.05 | 16.24 | 0.00 | 74.09 | 54.70 | 0.00 | 36.26 | 69.48 | 55.20 | 16.43 | 31.17 | 99.47 |
| Q86TI4 | WD repeat-containing protein 86 | **1.85** | **0.28** | 1020.58 | 991.50 | 1885.85 | 1395.30 | 1093.84 | 2533.05 | 1206.05 | 269.97 | 0.00 | 1370.90 | 1658.24 | 1505.31 | 4597.23 | 1666.01 | 517.42 |
| Q16695 | Histone H3.1t | **1.31** | **0.28** | 2419.39 | 1105.19 | 3159.98 | 1012.56 | 1420.01 | 1382.93 | 3395.95 | 3754.65 | 2143.40 | 2272.10 | 2968.68 | 3292.09 | 2102.16 | 3402.38 | 4922.45 |
| P27348 | 14-3-3 protein theta | **1.23** | **0.28** | 1087.05 | 322.52 | 1335.28 | 378.15 | 1257.66 | 521.60 | 1222.87 | 1303.88 | 1129.25 | 1455.80 | 1092.87 | 1075.40 | 902.19 | 1577.44 | 1907.95 |
| P46779 | 60S ribosomal protein L28 | **1.84** | **0.28** | 49.54 | 61.83 | 91.34 | 58.07 | 0.00 | 0.00 | 33.00 | 148.73 | 65.97 | 146.29 | 77.32 | 58.35 | 0.00 | 113.92 | 152.18 |
| P62899 | 60S ribosomal protein L31 | **1.79** | **0.28** | 91.95 | 119.74 | 164.96 | 90.44 | 112.59 | 0.00 | 0.00 | 289.10 | 58.09 | 162.44 | 186.34 | 197.50 | 0.00 | 168.25 | 275.22 |
| O00483 | NADH dehydrogenase [ubiquinone] 1 alpha subcomplex subunit 4 | **0.41** | **0.28** | 78.99 | 80.52 | 32.05 | 54.28 | 148.41 | 0.00 | 171.81 | 74.75 | 0.00 | 130.82 | 0.00 | 0.00 | 0.00 | 0.00 | 61.47 |
| P05141 | ADP/ATP translocase 2 | **1.48** | **0.28** | 266.91 | 158.27 | 395.10 | 201.05 | 271.79 | 0.00 | 374.49 | 396.23 | 292.03 | 284.49 | 753.11 | 313.52 | 164.93 | 427.88 | 426.68 |
| O14907 | Tax1-binding protein 3 | **0.44** | **0.28** | 27.59 | 26.24 | 12.00 | 18.65 | 34.79 | 0.00 | 55.48 | 47.66 | 0.00 | 38.37 | 0.00 | 0.00 | 0.00 | 33.63 | 0.00 |
| P13861 | cAMP-dependent protein kinase type II-alpha regulatory subunit | **0.51** | **0.28** | 19.56 | 15.10 | 9.92 | 12.72 | 16.18 | 41.41 | 24.53 | 15.66 | 0.00 | 0.00 | 26.66 | 0.00 | 7.78 | 0.00 | 25.07 |
| P07900 | Heat shock protein HSP 90-alpha | **1.41** | **0.28** | 887.27 | 348.45 | 1250.60 | 625.29 | 1042.14 | 434.66 | 930.20 | 1348.24 | 681.10 | 909.61 | 1287.22 | 939.52 | 434.14 | 1810.33 | 2122.77 |
| P13647 | Keratin, type II cytoskeletal 5 | **0.68** | **0.28** | 1227.24 | 424.52 | 836.22 | 648.53 | 1905.54 | 990.49 | 793.10 | 1313.56 | 1133.53 | 1700.85 | 461.24 | 498.52 | 260.43 | 1630.95 | 465.31 |
| Q12904 | Aminoacyl tRNA synthase complex-interacting multifunctional protein 1 | **0.37** | **0.28** | 14.93 | 13.78 | 5.48 | 13.41 | 22.37 | 0.00 | 28.08 | 24.19 | 0.00 | 0.00 | 0.00 | 0.00 | 0.00 | 0.00 | 32.86 |
| P83731 | 60S ribosomal protein L24 | **1.52** | **0.28** | 84.54 | 67.26 | 128.33 | 59.19 | 78.10 | 0.00 | 129.32 | 170.22 | 45.04 | 101.02 | 190.02 | 79.80 | 62.88 | 128.39 | 207.87 |
| P10316 | HLA class I histocompatibility antigen, A-69 alpha chain | **1.69** | **0.28** | 103.29 | 63.67 | 174.75 | 125.56 | 185.71 | 128.52 | 122.25 | 54.32 | 25.64 | 54.56 | 303.48 | 175.70 | 104.09 | 348.43 | 62.27 |
| Q9ULC5 | Long-chain-fatty-acid--CoA ligase 5 | **0.36** | **0.28** | 36.66 | 43.77 | 13.35 | 22.28 | 53.44 | 0.00 | 104.27 | 25.60 | 0.00 | 0.00 | 53.16 | 0.00 | 0.00 | 26.91 | 0.00 |
| P22492 | Histone H1t | **1.25** | **0.28** | 952.10 | 320.95 | 1189.86 | 359.11 | 1118.99 | 1408.14 | 873.01 | 785.27 | 575.10 | 1743.28 | 867.35 | 1293.49 | 1353.64 | 749.93 | 1131.49 |
| Q02539 | Histone H1.1 | **1.25** | **0.28** | 952.10 | 320.95 | 1189.86 | 359.11 | 1118.99 | 1408.14 | 873.01 | 785.27 | 575.10 | 1743.28 | 867.35 | 1293.49 | 1353.64 | 749.93 | 1131.49 |
| Q9BTT0 | Acidic leucine-rich nuclear phosphoprotein 32 family member E | **2.44** | **0.28** | 11.74 | 18.97 | 28.70 | 28.22 | 0.00 | 0.00 | 15.09 | 43.61 | 0.00 | 0.00 | 73.88 | 27.64 | 0.00 | 25.25 | 45.41 |
| Q8WZ60 | Kelch-like protein 6 | **0.42** | **0.28** | 878.45 | 815.64 | 366.83 | 673.93 | 1509.88 | 0.00 | 1234.14 | 1648.21 | 0.00 | 527.70 | 0.00 | 0.00 | 0.00 | 0.00 | 1673.26 |
| Q01085 | Nucleolysin TIAR | **0.33** | **0.28** | 13.75 | 15.82 | 4.50 | 11.03 | 14.49 | 0.00 | 15.65 | 38.63 | 0.00 | 0.00 | 0.00 | 0.00 | 0.00 | 27.02 | 0.00 |
| P08670 | Vimentin | **1.60** | **0.28** | 1180.18 | 926.03 | 1886.97 | 1091.67 | 1051.67 | 2716.85 | 1172.67 | 291.01 | 668.70 | 1484.04 | 1530.18 | 2043.46 | 3870.97 | 1811.04 | 582.14 |
| Q4KMP7 | TBC1 domain family member 10B | **0.09** | **0.28** | 113.23 | 186.38 | 9.98 | 24.45 | 56.92 | 0.00 | 441.91 | 67.32 | 0.00 | 0.00 | 0.00 | 0.00 | 0.00 | 0.00 | 59.90 |
| P10412 | Histone H1.4 | **1.36** | **0.28** | 1577.57 | 757.03 | 2148.90 | 881.28 | 1410.38 | 2825.19 | 1473.07 | 1421.80 | 757.40 | 3697.10 | 2567.88 | 1702.58 | 1549.63 | 1283.23 | 2092.96 |
| P16402 | Histone H1.3 | **1.36** | **0.28** | 1577.57 | 757.03 | 2148.90 | 881.28 | 1410.38 | 2825.19 | 1473.07 | 1421.80 | 757.40 | 3697.10 | 2567.88 | 1702.58 | 1549.63 | 1283.23 | 2092.96 |
| P16403 | Histone H1.2 | **1.36** | **0.28** | 1577.57 | 757.03 | 2148.90 | 881.28 | 1410.38 | 2825.19 | 1473.07 | 1421.80 | 757.40 | 3697.10 | 2567.88 | 1702.58 | 1549.63 | 1283.23 | 2092.96 |
| P17081 | Rho-related GTP-binding protein RhoQ | **0.55** | **0.28** | 107.87 | 70.90 | 59.78 | 68.62 | 145.24 | 116.21 | 40.99 | 201.95 | 34.95 | 0.00 | 114.59 | 0.00 | 89.94 | 154.17 | 0.00 |
| Q9H4E5 | Rho-related GTP-binding protein RhoJ | **0.55** | **0.28** | 107.87 | 70.90 | 59.78 | 68.62 | 145.24 | 116.21 | 40.99 | 201.95 | 34.95 | 0.00 | 114.59 | 0.00 | 89.94 | 154.17 | 0.00 |
| O00217 | NADH dehydrogenase [ubiquinone] iron-sulfur protein 8, mitochondrial | **2.50** | **0.29** | 4.35 | 9.74 | 10.90 | 9.30 | 21.77 | 0.00 | 0.00 | 0.00 | 0.00 | 10.36 | 19.74 | 0.00 | 14.05 | 21.23 | 0.00 |
| P12236 | ADP/ATP translocase 3 | **1.47** | **0.29** | 266.91 | 158.27 | 392.96 | 201.49 | 271.79 | 0.00 | 374.49 | 396.23 | 292.03 | 284.49 | 753.11 | 313.52 | 161.82 | 427.88 | 416.92 |
| Q08257 | Quinone oxidoreductase | **0.30** | **0.29** | 40.20 | 52.30 | 12.00 | 29.40 | 125.84 | 0.00 | 24.11 | 51.05 | 0.00 | 0.00 | 0.00 | 0.00 | 0.00 | 0.00 | 72.00 |
| P09493 | Tropomyosin alpha-1 chain | **2.00** | **0.29** | 571.98 | 450.76 | 1145.36 | 1049.00 | 1185.16 | 913.55 | 262.31 | 339.25 | 159.64 | 358.77 | 1005.49 | 3007.58 | 1647.06 | 662.23 | 191.01 |
| Q8IUE6 | Histone H2A type 2-B | **1.22** | **0.29** | 3716.41 | 822.99 | 4550.51 | 1461.74 | 3627.83 | 5004.80 | 3471.92 | 3751.28 | 2726.24 | 5569.19 | 5072.18 | 6711.63 | 3020.34 | 3511.86 | 3417.86 |
| P36543 | V-type proton ATPase subunit E 1 | **3.22** | **0.29** | 4.44 | 9.94 | 14.31 | 17.25 | 0.00 | 0.00 | 0.00 | 22.22 | 0.00 | 11.78 | 14.47 | 0.00 | 12.60 | 0.00 | 47.00 |
| A6NHL2 | Tubulin alpha chain-like 3 | **1.45** | **0.29** | 122.29 | 86.37 | 176.74 | 74.09 | 177.06 | 62.10 | 178.99 | 193.32 | 0.00 | 127.94 | 135.43 | 182.24 | 90.58 | 236.38 | 287.85 |
| Q14204 | Cytoplasmic dynein 1 heavy chain 1 | **1.59** | **0.29** | 38.28 | 29.61 | 60.85 | 35.70 | 37.10 | 0.00 | 45.07 | 81.63 | 27.61 | 33.46 | 121.84 | 44.29 | 30.40 | 50.04 | 85.05 |
| Q9UIF9 | Bromodomain adjacent to zinc finger domain protein 2A | **0.54** | **0.29** | 177.59 | 113.16 | 95.87 | 125.03 | 0.00 | 298.73 | 232.07 | 146.67 | 210.45 | 199.30 | 0.00 | 0.00 | 0.00 | 80.89 | 295.04 |
| O75347 | Tubulin-specific chaperone A | **0.41** | **0.29** | 28.04 | 29.76 | 11.63 | 18.50 | 70.53 | 0.00 | 28.80 | 40.89 | 0.00 | 0.00 | 28.28 | 0.00 | 0.00 | 0.00 | 41.51 |
| O75083 | WD repeat-containing protein 1 | **0.73** | **0.29** | 140.54 | 80.35 | 102.42 | 22.71 | 146.78 | 46.09 | 256.33 | 165.95 | 87.53 | 84.20 | 106.63 | 128.13 | 82.07 | 130.36 | 83.12 |
| P29692 | Elongation factor 1-delta | **1.55** | **0.29** | 65.04 | 68.13 | 100.93 | 36.54 | 60.84 | 0.00 | 109.02 | 155.34 | 0.00 | 74.01 | 105.16 | 83.71 | 56.12 | 138.84 | 147.75 |
| Q496Y0 | LON peptidase N-terminal domain and RING finger protein 3 | **1.54** | **0.29** | 149.25 | 145.66 | 229.15 | 90.65 | 387.66 | 109.72 | 162.88 | 85.99 | 0.00 | 155.80 | 331.94 | 318.24 | 211.04 | 255.85 | 102.00 |
| O00507 | Probable ubiquitin carboxyl-terminal hydrolase FAF-Y | **2.60** | **0.30** | 81.39 | 181.99 | 211.41 | 201.46 | 0.00 | 0.00 | 406.95 | 0.00 | 0.00 | 457.87 | 0.00 | 0.00 | 117.08 | 286.52 | 407.01 |
| P30084 | Enoyl-CoA hydratase, mitochondrial | **0.58** | **0.30** | 74.91 | 51.41 | 43.54 | 42.35 | 75.27 | 0.00 | 113.03 | 130.82 | 55.44 | 50.04 | 29.06 | 0.00 | 0.00 | 76.80 | 105.32 |
| P18464 | HLA class I histocompatibility antigen, B-51 alpha chain | **1.70** | **0.30** | 162.27 | 123.58 | 275.06 | 195.48 | 349.87 | 213.04 | 129.52 | 82.94 | 35.97 | 32.62 | 422.35 | 491.78 | 305.69 | 358.84 | 39.11 |
| P30490 | HLA class I histocompatibility antigen, B-52 alpha chain | **1.70** | **0.30** | 162.27 | 123.58 | 275.06 | 195.48 | 349.87 | 213.04 | 129.52 | 82.94 | 35.97 | 32.62 | 422.35 | 491.78 | 305.69 | 358.84 | 39.11 |
| P30491 | HLA class I histocompatibility antigen, B-53 alpha chain | **1.70** | **0.30** | 162.27 | 123.58 | 275.06 | 195.48 | 349.87 | 213.04 | 129.52 | 82.94 | 35.97 | 32.62 | 422.35 | 491.78 | 305.69 | 358.84 | 39.11 |
| P30498 | HLA class I histocompatibility antigen, B-78 alpha chain | **1.70** | **0.30** | 162.27 | 123.58 | 275.06 | 195.48 | 349.87 | 213.04 | 129.52 | 82.94 | 35.97 | 32.62 | 422.35 | 491.78 | 305.69 | 358.84 | 39.11 |
| P30685 | HLA class I histocompatibility antigen, B-35 alpha chain | **1.70** | **0.30** | 162.27 | 123.58 | 275.06 | 195.48 | 349.87 | 213.04 | 129.52 | 82.94 | 35.97 | 32.62 | 422.35 | 491.78 | 305.69 | 358.84 | 39.11 |
| P09661 | U2 small nuclear ribonucleoprotein A' | **2.25** | **0.30** | 12.65 | 17.57 | 28.47 | 27.45 | 0.00 | 0.00 | 0.00 | 35.83 | 27.40 | 71.13 | 21.16 | 0.00 | 0.00 | 34.79 | 43.77 |
| Q9Y2Q3 | Glutathione S-transferase kappa 1 | **1.42** | **0.30** | 59.28 | 36.42 | 83.94 | 37.02 | 87.94 | 0.00 | 77.18 | 82.50 | 48.75 | 77.72 | 107.87 | 91.94 | 32.29 | 57.10 | 136.74 |
| Q7L576 | Cytoplasmic FMR1-interacting protein 1 | **2.94** | **0.30** | 5.73 | 8.39 | 16.88 | 21.06 | 0.00 | 0.00 | 10.15 | 18.52 | 0.00 | 22.02 | 17.32 | 0.00 | 6.23 | 0.00 | 55.70 |
| P55209 | Nucleosome assembly protein 1-like 1 | **0.57** | **0.30** | 59.59 | 37.85 | 34.05 | 38.63 | 82.44 | 0.00 | 70.13 | 97.24 | 48.16 | 59.77 | 0.00 | 0.00 | 0.00 | 86.44 | 58.06 |
| Q8N1G4 | Leucine-rich repeat-containing protein 47 | **0.70** | **0.30** | 26.74 | 7.55 | 18.65 | 15.83 | 33.29 | 16.25 | 28.92 | 21.75 | 33.49 | 30.93 | 32.18 | 0.00 | 0.00 | 33.29 | 15.52 |
| P29590 | Protein PML | **1.63** | **0.30** | 26.71 | 22.33 | 43.48 | 27.49 | 60.34 | 0.00 | 30.46 | 15.07 | 27.70 | 36.15 | 18.43 | 63.75 | 88.10 | 36.43 | 18.05 |
| P31946 | 14-3-3 protein beta/alpha | **1.21** | **0.30** | 1081.92 | 310.51 | 1308.96 | 368.31 | 1260.90 | 534.40 | 1238.33 | 1246.71 | 1129.25 | 1480.43 | 1114.87 | 1119.41 | 831.70 | 1419.36 | 1888.01 |
| P99999 | Cytochrome c | **1.44** | **0.31** | 97.55 | 55.05 | 140.01 | 71.27 | 131.33 | 0.00 | 127.14 | 115.91 | 113.36 | 182.32 | 143.65 | 78.08 | 46.57 | 144.51 | 244.93 |
| Q9H6S3 | Epidermal growth factor receptor kinase substrate 8-like protein 2 | **1.42** | **0.31** | 70.05 | 47.22 | 99.46 | 42.71 | 0.00 | 76.19 | 83.86 | 59.53 | 130.66 | 94.62 | 112.62 | 162.36 | 115.13 | 77.75 | 34.27 |
| Q92973 | Transportin-1 | **1.94** | **0.31** | 11.16 | 10.33 | 21.67 | 19.45 | 21.35 | 0.00 | 0.00 | 17.72 | 16.72 | 33.76 | 24.71 | 0.00 | 21.63 | 0.00 | 49.89 |
| O75448 | Mediator of RNA polymerase II transcription subunit 24 | **1.36** | **0.31** | 1231.93 | 748.15 | 1673.51 | 608.71 | 1304.99 | 0.00 | 1990.12 | 1606.66 | 1257.91 | 1386.10 | 1321.54 | 1410.47 | 1135.71 | 2039.66 | 2747.59 |
| Q7Z591 | AT-hook-containing transcription factor | **1.36** | **0.31** | 1231.93 | 748.15 | 1673.51 | 608.71 | 1304.99 | 0.00 | 1990.12 | 1606.66 | 1257.91 | 1386.10 | 1321.54 | 1410.47 | 1135.71 | 2039.66 | 2747.59 |
| Q86VV8 | Rotatin | **1.36** | **0.31** | 1231.93 | 748.15 | 1673.51 | 608.71 | 1304.99 | 0.00 | 1990.12 | 1606.66 | 1257.91 | 1386.10 | 1321.54 | 1410.47 | 1135.71 | 2039.66 | 2747.59 |
| Q9UMN6 | Histone-lysine N-methyltransferase 2B | **1.36** | **0.31** | 1231.93 | 748.15 | 1673.51 | 608.71 | 1304.99 | 0.00 | 1990.12 | 1606.66 | 1257.91 | 1386.10 | 1321.54 | 1410.47 | 1135.71 | 2039.66 | 2747.59 |
| O95758 | Polypyrimidine tract-binding protein 3 | **2.26** | **0.31** | 31.17 | 47.48 | 70.42 | 68.64 | 0.00 | 0.00 | 48.50 | 107.34 | 0.00 | 134.31 | 31.03 | 0.00 | 0.00 | 103.75 | 153.44 |
| P62745 | Rho-related GTP-binding protein RhoB | **1.89** | **0.31** | 38.60 | 37.72 | 72.77 | 61.51 | 60.70 | 0.00 | 47.40 | 84.91 | 0.00 | 0.00 | 87.33 | 76.77 | 0.00 | 132.07 | 140.48 |
| O15144 | Actin-related protein 2/3 complex subunit 2 | **1.51** | **0.31** | 61.15 | 55.95 | 92.12 | 29.83 | 95.96 | 0.00 | 103.98 | 105.82 | 0.00 | 66.53 | 101.95 | 86.59 | 54.36 | 137.43 | 105.83 |
| Q9HAV0 | Guanine nucleotide-binding protein subunit beta-4 | **1.42** | **0.31** | 73.07 | 60.26 | 103.41 | 31.28 | 28.80 | 0.00 | 71.65 | 141.67 | 123.23 | 114.32 | 67.93 | 100.44 | 68.78 | 120.79 | 148.19 |
| Q9P0M6 | Core histone macro-H2A.2 | **1.62** | **0.31** | 84.42 | 93.21 | 136.99 | 69.03 | 94.28 | 0.00 | 226.21 | 101.63 | 0.00 | 228.45 | 141.18 | 187.21 | 29.35 | 100.45 | 135.29 |
| Q15942 | Zyxin | **0.41** | **0.31** | 30.32 | 34.84 | 12.42 | 19.69 | 83.85 | 0.00 | 41.78 | 0.00 | 25.98 | 30.60 | 0.00 | 0.00 | 43.89 | 0.00 | 0.00 |
| P06865 | Beta-hexosaminidase subunit alpha | **0.38** | **0.31** | 12.10 | 11.68 | 4.64 | 11.36 | 21.15 | 0.00 | 14.38 | 24.97 | 0.00 | 0.00 | 27.82 | 0.00 | 0.00 | 0.00 | 0.00 |
| Q8WUY1 | Protein THEM6 | **0.40** | **0.31** | 20.16 | 21.88 | 8.10 | 15.54 | 45.47 | 0.00 | 40.86 | 14.45 | 0.00 | 0.00 | 0.00 | 0.00 | 0.00 | 9.82 | 38.81 |
| P52565 | Rho GDP-dissociation inhibitor 1 | **0.84** | **0.31** | 168.80 | 49.18 | 141.52 | 35.44 | 158.62 | 162.01 | 175.34 | 242.66 | 105.35 | 142.64 | 100.08 | 129.55 | 119.64 | 203.00 | 154.20 |
| Q03169 | Tumor necrosis factor alpha-induced protein 2 | **0.39** | **0.31** | 9.78 | 8.97 | 3.84 | 9.41 | 0.00 | 0.00 | 17.59 | 15.77 | 15.56 | 0.00 | 0.00 | 0.00 | 0.00 | 0.00 | 23.04 |
| P31146 | Coronin-1A | **1.58** | **0.31** | 60.49 | 54.56 | 95.31 | 53.35 | 61.32 | 148.64 | 40.24 | 52.27 | 0.00 | 52.77 | 113.25 | 187.33 | 106.61 | 70.23 | 41.69 |
| P62995 | Transformer-2 protein homolog beta | **0.09** | **0.31** | 200.61 | 355.20 | 17.38 | 30.33 | 30.77 | 835.32 | 63.87 | 51.01 | 22.07 | 30.07 | 0.00 | 0.00 | 0.00 | 0.00 | 74.20 |
| P50213 | Isocitrate dehydrogenase [NAD] subunit alpha, mitochondrial | **0.50** | **0.32** | 21.76 | 15.03 | 10.94 | 18.07 | 30.53 | 0.00 | 39.83 | 21.80 | 16.64 | 22.85 | 0.00 | 0.00 | 0.00 | 0.00 | 42.77 |
| Q8TF74 | WAS/WASL-interacting protein family member 2 | **2.65** | **0.32** | 40.54 | 90.66 | 107.25 | 112.95 | 0.00 | 0.00 | 0.00 | 202.72 | 0.00 | 36.53 | 178.55 | 0.00 | 0.00 | 151.63 | 276.76 |
| Q16181 | Septin-7 | **1.19** | **0.32** | 60.78 | 22.28 | 72.27 | 13.34 | 48.99 | 41.41 | 89.23 | 80.26 | 43.98 | 57.49 | 80.66 | 70.16 | 58.27 | 92.12 | 74.95 |
| P62633 | Cellular nucleic acid-binding protein | **2.22** | **0.32** | 8.69 | 8.88 | 19.31 | 22.03 | 15.15 | 0.00 | 8.55 | 19.77 | 0.00 | 48.97 | 0.00 | 0.00 | 0.00 | 29.70 | 37.21 |
| Q9C0C4 | Semaphorin-4C | **1.94** | **0.32** | 158.36 | 189.90 | 307.47 | 262.71 | 160.68 | 480.71 | 101.96 | 48.47 | 0.00 | 128.89 | 172.70 | 745.00 | 321.06 | 455.31 | 21.88 |
| P00558 | Phosphoglycerate kinase 1 | **0.69** | **0.32** | 827.07 | 500.26 | 570.07 | 98.54 | 1174.97 | 297.78 | 563.39 | 1513.98 | 585.25 | 622.27 | 636.89 | 398.10 | 578.11 | 665.73 | 519.35 |
| Q5VXU9 | Uncharacterized protein C9orf84 | **2.01** | **0.32** | 178.38 | 280.19 | 359.15 | 285.83 | 669.05 | 0.00 | 93.32 | 129.50 | 0.00 | 131.89 | 474.33 | 387.74 | 821.09 | 339.87 | 0.00 |
| P06241 | Tyrosine-protein kinase Fyn | **1.72** | **0.32** | 21.16 | 15.58 | 36.32 | 28.73 | 20.24 | 0.00 | 43.87 | 19.00 | 22.69 | 53.83 | 45.01 | 0.00 | 0.00 | 55.59 | 63.46 |
| P09497 | Clathrin light chain B | **0.71** | **0.32** | 50.41 | 18.90 | 35.97 | 25.29 | 79.78 | 53.05 | 42.92 | 48.24 | 28.07 | 0.00 | 45.60 | 71.42 | 51.62 | 22.13 | 25.05 |
| P11279 | Lysosome-associated membrane glycoprotein 1 | **0.34** | **0.32** | 481.98 | 625.12 | 165.29 | 86.69 | 122.40 | 173.90 | 1575.98 | 428.75 | 108.86 | 178.12 | 317.36 | 195.45 | 119.13 | 99.68 | 81.99 |
| Q56UQ5 | TPT1-like protein | **1.66** | **0.32** | 58.15 | 58.20 | 96.37 | 61.98 | 71.86 | 0.00 | 0.00 | 83.58 | 135.29 | 100.64 | 209.29 | 88.33 | 100.03 | 40.51 | 39.44 |
| P05198 | Eukaryotic translation initiation factor 2 subunit 1 | **1.56** | **0.32** | 30.74 | 28.89 | 47.99 | 25.82 | 40.98 | 0.00 | 60.25 | 52.50 | 0.00 | 57.99 | 73.37 | 0.00 | 40.58 | 61.85 | 54.16 |
| P00441 | Superoxide dismutase [Cu-Zn] | **0.51** | **0.32** | 126.89 | 122.34 | 64.71 | 27.00 | 241.03 | 0.00 | 0.00 | 147.27 | 246.15 | 86.13 | 89.16 | 42.28 | 32.67 | 46.41 | 91.62 |
| O60763 | General vesicular transport factor p115 | **1.27** | **0.33** | 30.56 | 17.83 | 38.75 | 7.03 | 36.70 | 0.00 | 45.11 | 39.97 | 31.00 | 41.11 | 52.12 | 35.25 | 34.55 | 34.04 | 35.43 |
| P02748 | Complement component C9 | **2.52** | **0.33** | 13.95 | 15.08 | 35.12 | 43.24 | 12.36 | 0.00 | 35.17 | 22.19 | 0.00 | 0.00 | 22.89 | 115.76 | 46.70 | 25.36 | 0.00 |
| Q07065 | Cytoskeleton-associated protein 4 | **1.60** | **0.33** | 79.74 | 30.25 | 127.76 | 99.06 | 102.84 | 93.33 | 27.34 | 93.63 | 81.53 | 105.31 | 49.07 | 111.13 | 322.76 | 65.86 | 112.46 |
| Q86XR8 | Centrosomal protein of 57 kDa | **0.35** | **0.33** | 837.94 | 1081.57 | 294.59 | 183.63 | 313.88 | 360.13 | 550.86 | 205.07 | 2759.74 | 557.51 | 306.64 | 306.70 | 377.01 | 219.70 | 0.00 |
| P09211 | Glutathione S-transferase P | **0.67** | **0.33** | 1133.21 | 709.14 | 755.49 | 502.37 | 1128.05 | 262.16 | 1352.72 | 2164.75 | 758.37 | 314.30 | 714.47 | 375.74 | 447.83 | 1607.38 | 1073.22 |
| Q8NBX0 | Saccharopine dehydrogenase-like oxidoreductase | **0.56** | **0.33** | 61.92 | 45.12 | 34.71 | 42.28 | 77.90 | 0.00 | 107.20 | 93.90 | 30.59 | 72.24 | 0.00 | 38.89 | 0.00 | 0.00 | 97.14 |
| Q96DU9 | Polyadenylate-binding protein 5 | **1.94** | **0.33** | 19.89 | 28.02 | 38.59 | 31.44 | 0.00 | 0.00 | 40.42 | 59.04 | 0.00 | 57.82 | 63.47 | 0.00 | 0.00 | 69.75 | 40.52 |
| P62701 | 40S ribosomal protein S4, X isoform | **1.35** | **0.33** | 92.45 | 60.63 | 124.74 | 43.35 | 71.42 | 0.00 | 132.49 | 155.78 | 102.58 | 118.47 | 172.27 | 107.13 | 63.52 | 109.34 | 177.69 |
| P02790 | Hemopexin | **1.52** | **0.33** | 207.53 | 120.76 | 316.48 | 208.26 | 143.26 | 418.61 | 189.43 | 165.91 | 120.46 | 163.01 | 101.98 | 375.02 | 684.56 | 344.89 | 229.39 |
| P02100 | Hemoglobin subunit epsilon | **0.53** | **0.33** | 19032.90 | 20032.40 | 10069.02 | 7179.62 | 7876.35 | 9397.75 | 13382.26 | 9822.42 | 54685.69 | 5353.02 | 5979.14 | 4208.00 | 23297.30 | 12836.93 | 8739.71 |
| P07196 | Neurofilament light polypeptide | **1.68** | **0.33** | 710.83 | 736.39 | 1190.73 | 798.29 | 777.83 | 1937.06 | 542.04 | 160.39 | 136.84 | 940.94 | 829.76 | 1492.54 | 2640.86 | 892.65 | 347.61 |
| P07197 | Neurofilament medium polypeptide | **1.68** | **0.33** | 710.83 | 736.39 | 1190.73 | 798.29 | 777.83 | 1937.06 | 542.04 | 160.39 | 136.84 | 940.94 | 829.76 | 1492.54 | 2640.86 | 892.65 | 347.61 |
| Q16352 | Alpha-internexin | **1.68** | **0.33** | 710.83 | 736.39 | 1190.73 | 798.29 | 777.83 | 1937.06 | 542.04 | 160.39 | 136.84 | 940.94 | 829.76 | 1492.54 | 2640.86 | 892.65 | 347.61 |
| Q04917 | 14-3-3 protein eta | **1.18** | **0.33** | 1040.13 | 218.70 | 1224.41 | 346.29 | 1112.54 | 650.20 | 1157.38 | 1151.26 | 1129.25 | 1303.58 | 950.85 | 1255.23 | 760.88 | 1307.65 | 1768.25 |
| P31947 | 14-3-3 protein sigma | **1.23** | **0.33** | 1035.29 | 325.19 | 1272.36 | 422.03 | 1186.23 | 456.08 | 1201.39 | 1203.49 | 1129.25 | 1476.78 | 1091.61 | 1028.91 | 755.12 | 1310.95 | 1970.78 |
| P13489 | Ribonuclease inhibitor | **0.86** | **0.33** | 80.50 | 22.39 | 69.03 | 14.69 | 105.10 | 61.69 | 92.09 | 91.33 | 52.31 | 59.74 | 45.15 | 76.52 | 74.54 | 70.97 | 87.27 |
| Q9P1Z0 | Zinc finger and BTB domain-containing protein 4 | **0.48** | **0.33** | 106.11 | 119.45 | 50.55 | 56.10 | 0.00 | 0.00 | 172.51 | 80.27 | 277.79 | 116.92 | 0.00 | 0.00 | 0.00 | 96.89 | 89.52 |
| P01008 | Antithrombin-III | **0.78** | **0.33** | 110.29 | 28.58 | 86.05 | 45.93 | 130.81 | 102.76 | 112.41 | 139.29 | 66.20 | 54.71 | 64.46 | 163.23 | 83.41 | 113.34 | 37.15 |
| B5ME19 | Eukaryotic translation initiation factor 3 subunit C-like protein | **1.59** | **0.34** | 19.34 | 19.21 | 30.79 | 18.00 | 33.09 | 0.00 | 21.15 | 42.48 | 0.00 | 18.34 | 38.17 | 42.44 | 0.00 | 46.96 | 38.84 |
| Q99613 | Eukaryotic translation initiation factor 3 subunit C | **1.59** | **0.34** | 19.34 | 19.21 | 30.79 | 18.00 | 33.09 | 0.00 | 21.15 | 42.48 | 0.00 | 18.34 | 38.17 | 42.44 | 0.00 | 46.96 | 38.84 |
| P60981 | Destrin | **0.74** | **0.34** | 78.87 | 30.48 | 58.66 | 34.47 | 80.87 | 53.41 | 130.47 | 63.26 | 66.36 | 64.59 | 50.20 | 97.03 | 51.90 | 0.00 | 88.24 |
| O95372 | Acyl-protein thioesterase 2 | **0.47** | **0.34** | 33.48 | 30.79 | 15.57 | 27.73 | 53.41 | 0.00 | 0.00 | 52.19 | 61.82 | 68.33 | 0.00 | 0.00 | 0.00 | 0.00 | 25.12 |
| P31930 | Cytochrome b-c1 complex subunit 1, mitochondrial | **0.65** | **0.34** | 62.71 | 38.99 | 40.59 | 33.29 | 92.73 | 0.00 | 87.85 | 83.61 | 49.36 | 72.89 | 72.76 | 0.00 | 0.00 | 45.17 | 52.74 |
| P62851 | 40S ribosomal protein S25 | **1.15** | **0.34** | 255.42 | 63.71 | 294.82 | 64.56 | 338.00 | 305.60 | 220.38 | 227.74 | 185.37 | 366.40 | 352.66 | 333.79 | 210.30 | 243.04 | 262.72 |
| P39656 | Dolichyl-diphosphooligosaccharide--protein glycosyltransferase 48 kDa subunit | **1.32** | **0.34** | 44.55 | 32.97 | 58.86 | 10.51 | 40.53 | 0.00 | 43.13 | 92.94 | 46.13 | 68.57 | 49.11 | 45.98 | 60.69 | 72.55 | 56.23 |
| P67936 | Tropomyosin alpha-4 chain | **1.65** | **0.34** | 600.60 | 417.46 | 988.30 | 764.15 | 1183.88 | 894.67 | 326.04 | 380.87 | 217.54 | 384.09 | 935.34 | 2261.47 | 1459.30 | 687.89 | 201.71 |
| O95931 | Chromobox protein homolog 7 | **1.45** | **0.34** | 127.36 | 100.16 | 184.70 | 88.54 | 134.59 | 0.00 | 122.34 | 279.38 | 100.48 | 154.43 | 125.67 | 114.32 | 119.44 | 285.11 | 309.20 |
| Q9Y281 | Cofilin-2 | **0.79** | **0.34** | 637.01 | 202.04 | 503.43 | 232.25 | 824.91 | 522.00 | 588.93 | 861.37 | 387.82 | 500.64 | 287.84 | 237.65 | 599.09 | 513.38 | 882.00 |
| O00468 | Agrin | **0.43** | **0.34** | 39.72 | 47.65 | 17.00 | 26.59 | 105.50 | 0.00 | 74.00 | 19.08 | 0.00 | 0.00 | 56.74 | 0.00 | 0.00 | 45.26 | 0.00 |
| Q9Y3Y2 | Chromatin target of PRMT1 protein | **1.81** | **0.34** | 17.54 | 17.76 | 31.66 | 26.99 | 25.51 | 0.00 | 0.00 | 41.47 | 20.71 | 57.97 | 27.00 | 0.00 | 0.00 | 57.72 | 47.26 |
| P68402 | Platelet-activating factor acetylhydrolase IB subunit beta | **0.63** | **0.34** | 52.96 | 37.22 | 33.45 | 27.60 | 86.85 | 0.00 | 29.68 | 65.50 | 82.77 | 0.00 | 59.17 | 0.00 | 61.28 | 44.95 | 35.29 |
| P35637 | RNA-binding protein FUS | **0.77** | **0.34** | 126.99 | 23.69 | 97.71 | 65.26 | 142.95 | 103.40 | 157.82 | 125.76 | 105.04 | 162.89 | 83.22 | 0.00 | 51.83 | 123.36 | 164.98 |
| O60812 | Heterogeneous nuclear ribonucleoprotein C-like 1 | **1.31** | **0.35** | 172.61 | 95.82 | 226.29 | 83.22 | 163.92 | 56.55 | 234.98 | 296.92 | 110.69 | 212.44 | 259.28 | 134.66 | 131.32 | 279.13 | 340.92 |
| P28074 | Proteasome subunit beta type-5 | **2.09** | **0.35** | 10.85 | 15.14 | 22.73 | 22.71 | 0.00 | 0.00 | 23.02 | 31.25 | 0.00 | 36.69 | 5.60 | 0.00 | 6.32 | 29.55 | 58.20 |
| P16520 | Guanine nucleotide-binding protein G(I)/G(S)/G(T) subunit beta-3 | **1.49** | **0.35** | 72.69 | 72.71 | 108.29 | 24.67 | 28.80 | 0.00 | 34.48 | 134.31 | 165.86 | 114.32 | 67.93 | 100.44 | 104.15 | 120.79 | 142.11 |
| P43243 | Matrin-3 | **1.37** | **0.35** | 60.10 | 45.60 | 82.44 | 15.88 | 68.57 | 0.00 | 105.14 | 99.34 | 27.47 | 86.29 | 68.12 | 59.49 | 85.40 | 102.30 | 93.01 |
| O75947 | ATP synthase subunit d, mitochondrial | **0.59** | **0.35** | 96.77 | 89.62 | 57.53 | 34.85 | 230.92 | 0.00 | 129.08 | 87.33 | 36.50 | 97.47 | 80.87 | 42.18 | 0.00 | 49.26 | 75.42 |
| P49327 | Fatty acid synthase | **2.64** | **0.35** | 87.53 | 68.57 | 231.43 | 315.58 | 52.81 | 0.00 | 79.13 | 178.71 | 127.00 | 187.78 | 246.45 | 42.92 | 0.00 | 64.49 | 846.93 |
| P07947 | Tyrosine-protein kinase Yes | **1.66** | **0.35** | 21.16 | 15.58 | 35.14 | 27.92 | 20.24 | 0.00 | 43.87 | 19.00 | 22.69 | 53.83 | 45.01 | 0.00 | 0.00 | 48.52 | 63.46 |
| Q86UP2 | Kinectin | **0.47** | **0.35** | 35.69 | 40.82 | 16.64 | 21.80 | 105.65 | 0.00 | 21.35 | 32.46 | 18.98 | 55.10 | 22.72 | 0.00 | 0.00 | 0.00 | 22.04 |
| P14927 | Cytochrome b-c1 complex subunit 7 | **0.46** | **0.35** | 43.68 | 55.51 | 20.19 | 17.56 | 135.18 | 0.00 | 49.58 | 33.63 | 0.00 | 27.37 | 24.14 | 0.00 | 0.00 | 24.02 | 45.58 |
| P01011 | Alpha-1-antichymotrypsin | **0.62** | **0.35** | 110.26 | 91.78 | 68.10 | 46.71 | 55.77 | 272.14 | 53.91 | 79.96 | 89.52 | 114.73 | 0.00 | 78.60 | 26.49 | 73.46 | 115.31 |
| O14672 | Disintegrin and metalloproteinase domain-containing protein 10 | **0.56** | **0.35** | 25.57 | 20.77 | 14.40 | 16.91 | 22.75 | 0.00 | 57.89 | 26.08 | 21.12 | 20.83 | 0.00 | 0.00 | 0.00 | 26.08 | 39.48 |
| P52566 | Rho GDP-dissociation inhibitor 2 | **1.17** | **0.35** | 132.75 | 33.01 | 155.04 | 40.45 | 139.29 | 169.21 | 86.62 | 154.98 | 113.64 | 126.84 | 189.35 | 179.02 | 155.09 | 190.85 | 89.08 |
| Q02880 | DNA topoisomerase 2-beta | **2.19** | **0.35** | 14.75 | 20.99 | 32.34 | 34.84 | 0.00 | 0.00 | 28.79 | 44.95 | 0.00 | 16.07 | 34.61 | 0.00 | 0.00 | 89.18 | 54.15 |
| Q8NBS9 | Thioredoxin domain-containing protein 5 | **1.34** | **0.35** | 80.52 | 29.30 | 107.89 | 59.35 | 55.70 | 111.02 | 97.24 | 95.38 | 43.28 | 63.23 | 77.67 | 71.52 | 196.33 | 170.77 | 67.81 |
| Q07507 | Dermatopontin | **1.77** | **0.35** | 39.11 | 57.74 | 69.38 | 44.93 | 0.00 | 128.29 | 67.28 | 0.00 | 0.00 | 69.81 | 60.32 | 133.27 | 53.88 | 98.97 | 0.00 |
| O43653 | Prostate stem cell antigen | **0.15** | **0.35** | 243.11 | 439.03 | 37.03 | 47.57 | 1012.57 | 202.98 | 0.00 | 0.00 | 0.00 | 9.28 | 124.49 | 49.20 | 0.00 | 0.00 | 39.21 |
| Q9Y230 | RuvB-like 2 | **1.47** | **0.35** | 24.55 | 23.27 | 36.20 | 12.45 | 30.93 | 0.00 | 47.70 | 44.12 | 0.00 | 35.86 | 42.48 | 35.20 | 12.83 | 42.34 | 48.48 |
| P10319 | HLA class I histocompatibility antigen, B-58 alpha chain | **1.67** | **0.36** | 186.95 | 136.62 | 311.57 | 255.50 | 349.87 | 304.64 | 161.31 | 82.94 | 35.97 | 37.32 | 422.35 | 715.59 | 305.69 | 349.33 | 39.11 |
| P54709 | Sodium/potassium-transporting ATPase subunit beta-3 | **1.93** | **0.36** | 15.65 | 23.16 | 30.28 | 26.21 | 0.00 | 0.00 | 51.54 | 26.69 | 0.00 | 62.17 | 48.07 | 0.00 | 25.48 | 0.00 | 45.94 |
| P67809 | Nuclease-sensitive element-binding protein 1 | **1.34** | **0.36** | 116.43 | 44.21 | 156.31 | 82.28 | 98.24 | 89.61 | 127.74 | 188.39 | 78.19 | 254.77 | 146.89 | 67.30 | 60.58 | 169.51 | 238.83 |
| Q13011 | Delta(3,5)-Delta(2,4)-dienoyl-CoA isomerase, mitochondrial | **1.62** | **0.36** | 37.09 | 31.44 | 59.92 | 44.23 | 34.59 | 20.31 | 46.52 | 84.01 | 0.00 | 38.65 | 46.09 | 18.80 | 27.33 | 99.04 | 129.60 |
| Q15286 | Ras-related protein Rab-35 | **1.27** | **0.36** | 348.01 | 98.79 | 442.50 | 198.00 | 357.76 | 222.77 | 365.52 | 492.27 | 301.74 | 770.73 | 321.50 | 366.11 | 278.29 | 317.25 | 601.10 |
| O43852 | Calumenin | **2.92** | **0.36** | 25.04 | 34.30 | 73.14 | 106.30 | 61.36 | 63.84 | 0.00 | 0.00 | 0.00 | 14.63 | 288.00 | 29.31 | 15.97 | 35.76 | 55.17 |
| P20700 | Lamin-B1 | **1.32** | **0.36** | 87.32 | 34.43 | 114.87 | 55.52 | 46.64 | 58.43 | 122.90 | 118.59 | 90.06 | 192.25 | 97.06 | 46.54 | 66.75 | 159.23 | 127.40 |
| P30086 | Phosphatidylethanolamine-binding protein 1 | **0.74** | **0.36** | 416.81 | 126.52 | 308.28 | 223.10 | 562.87 | 244.53 | 420.92 | 347.99 | 507.74 | 225.13 | 188.09 | 187.58 | 123.85 | 720.08 | 404.92 |
| P54578 | Ubiquitin carboxyl-terminal hydrolase 14 | **6.75** | **0.36** | 18.13 | 24.89 | 122.32 | 239.82 | 0.00 | 0.00 | 42.89 | 47.77 | 0.00 | 18.64 | 609.29 | 0.00 | 0.00 | 61.21 | 44.80 |
| Q8NCW5 | NAD(P)H-hydrate epimerase | **0.51** | **0.36** | 27.81 | 28.19 | 14.08 | 19.30 | 49.54 | 0.00 | 0.00 | 61.87 | 27.63 | 0.00 | 18.70 | 0.00 | 16.47 | 0.00 | 49.30 |
| P07602 | Proactivator polypeptide | **0.51** | **0.36** | 241.44 | 299.13 | 122.28 | 66.80 | 772.63 | 169.73 | 76.70 | 98.27 | 89.84 | 43.57 | 240.73 | 131.06 | 83.13 | 102.87 | 132.30 |
| P55735 | Protein SEC13 homolog | **1.49** | **0.36** | 24.54 | 23.09 | 36.60 | 18.78 | 37.04 | 0.00 | 35.69 | 49.99 | 0.00 | 0.00 | 43.75 | 39.10 | 41.29 | 40.77 | 54.70 |
| Q8TER0 | Sushi, nidogen and EGF-like domain-containing protein 1 | **0.43** | **0.36** | 79.15 | 73.48 | 33.65 | 82.43 | 116.36 | 0.00 | 152.96 | 126.45 | 0.00 | 0.00 | 0.00 | 0.00 | 0.00 | 0.00 | 201.91 |
| P36578 | 60S ribosomal protein L4 | **1.57** | **0.36** | 86.48 | 87.49 | 135.49 | 82.27 | 14.34 | 0.00 | 146.46 | 204.62 | 66.95 | 115.05 | 164.57 | 61.28 | 38.99 | 168.39 | 264.64 |
| Q9BYZ2 | L-lactate dehydrogenase A-like 6B | **0.41** | **0.37** | 83.16 | 113.97 | 33.72 | 52.74 | 92.82 | 0.00 | 47.98 | 274.98 | 0.00 | 0.00 | 0.00 | 0.00 | 0.00 | 89.62 | 112.68 |
| Q9NVA2 | Septin-11 | **1.34** | **0.37** | 41.56 | 30.83 | 55.63 | 17.50 | 0.00 | 41.41 | 82.02 | 56.98 | 27.41 | 52.38 | 28.57 | 82.14 | 55.46 | 63.85 | 51.40 |
| P07203 | Glutathione peroxidase 1 | **0.57** | **0.37** | 30.70 | 22.76 | 17.56 | 22.83 | 32.24 | 0.00 | 59.08 | 43.76 | 18.42 | 0.00 | 26.03 | 57.43 | 21.87 | 0.00 | 0.00 |
| P30041 | Peroxiredoxin-6 | **1.25** | **0.37** | 206.14 | 71.45 | 257.59 | 101.25 | 234.45 | 108.93 | 189.41 | 304.73 | 193.17 | 239.52 | 175.24 | 328.73 | 194.82 | 179.30 | 427.95 |
| P22105 | Tenascin-X | **2.63** | **0.37** | 423.17 | 831.85 | 1111.54 | 1427.11 | 213.87 | 0.00 | 0.00 | 1901.97 | 0.00 | 821.83 | 0.00 | 30.19 | 2912.01 | 0.00 | 2905.20 |
| O43776 | Asparagine--tRNA ligase, cytoplasmic | **1.62** | **0.37** | 36.70 | 35.68 | 59.28 | 42.11 | 42.43 | 0.00 | 64.34 | 76.71 | 0.00 | 35.86 | 113.27 | 41.34 | 0.00 | 67.11 | 98.09 |
| O15069 | NAC-alpha domain-containing protein 1 | **1.53** | **0.37** | 45.05 | 42.06 | 68.88 | 41.13 | 62.15 | 0.00 | 76.03 | 87.08 | 0.00 | 96.49 | 101.52 | 43.00 | 0.00 | 68.56 | 103.74 |
| P02458 | Collagen alpha-1(II) chain | **0.25** | **0.37** | 1725.28 | 2854.88 | 427.77 | 223.91 | 397.45 | 6709.52 | 1519.44 | 0.00 | 0.00 | 512.09 | 475.66 | 422.71 | 658.92 | 497.24 | 0.00 |
| P07737 | Profilin-1 | **1.23** | **0.37** | 746.77 | 354.64 | 920.13 | 253.33 | 674.06 | 583.24 | 982.84 | 1202.55 | 291.16 | 890.27 | 676.45 | 1323.66 | 961.01 | 1036.57 | 632.83 |
| Q16363 | Laminin subunit alpha-4 | **2.62** | **0.37** | 15.39 | 34.41 | 40.37 | 49.99 | 0.00 | 0.00 | 76.94 | 0.00 | 0.00 | 18.40 | 0.00 | 98.75 | 15.83 | 109.25 | 0.00 |
| Q9NSD9 | Phenylalanine--tRNA ligase beta subunit | **2.01** | **0.37** | 7.72 | 12.55 | 15.50 | 14.40 | 0.00 | 0.00 | 9.74 | 28.87 | 0.00 | 26.18 | 13.13 | 0.00 | 0.00 | 17.42 | 36.29 |
| P01765 | Ig heavy chain V-III region TIL | **0.48** | **0.37** | 138.85 | 143.49 | 66.53 | 111.28 | 0.00 | 266.07 | 123.86 | 0.00 | 304.34 | 0.00 | 37.64 | 0.00 | 284.93 | 76.62 | 0.00 |
| Q9NYL9 | Tropomodulin-3 | **0.64** | **0.37** | 33.87 | 28.13 | 21.52 | 14.35 | 77.41 | 0.00 | 37.31 | 30.89 | 23.75 | 39.94 | 0.00 | 10.53 | 23.49 | 23.79 | 31.39 |
| P18462 | HLA class I histocompatibility antigen, A-25 alpha chain | **1.48** | **0.37** | 120.71 | 87.90 | 179.10 | 112.34 | 264.50 | 113.60 | 122.25 | 69.28 | 33.91 | 51.60 | 303.48 | 177.81 | 144.55 | 319.85 | 77.29 |
| P30450 | HLA class I histocompatibility antigen, A-26 alpha chain | **1.48** | **0.37** | 120.71 | 87.90 | 179.10 | 112.34 | 264.50 | 113.60 | 122.25 | 69.28 | 33.91 | 51.60 | 303.48 | 177.81 | 144.55 | 319.85 | 77.29 |
| P30457 | HLA class I histocompatibility antigen, A-66 alpha chain | **1.48** | **0.37** | 120.71 | 87.90 | 179.10 | 112.34 | 264.50 | 113.60 | 122.25 | 69.28 | 33.91 | 51.60 | 303.48 | 177.81 | 144.55 | 319.85 | 77.29 |
| P22061 | Protein-L-isoaspartate(D-aspartate) O-methyltransferase | **2.12** | **0.37** | 23.35 | 21.37 | 49.61 | 62.62 | 36.79 | 0.00 | 0.00 | 40.90 | 39.08 | 103.52 | 0.00 | 146.13 | 0.00 | 0.00 | 47.98 |
| Q58FF8 | Putative heat shock protein HSP 90-beta 2 | **1.33** | **0.37** | 702.55 | 258.70 | 935.71 | 497.33 | 818.05 | 321.82 | 723.45 | 1023.63 | 625.80 | 792.11 | 706.74 | 699.86 | 336.00 | 1443.36 | 1636.19 |
| Q5SSJ5 | Heterochromatin protein 1-binding protein 3 | **1.44** | **0.37** | 43.66 | 40.44 | 62.71 | 26.62 | 0.00 | 0.00 | 82.52 | 72.57 | 63.22 | 90.90 | 65.45 | 29.20 | 30.81 | 78.48 | 81.43 |
| P84095 | Rho-related GTP-binding protein RhoG | **0.65** | **0.37** | 107.87 | 70.90 | 70.20 | 62.17 | 145.24 | 116.21 | 40.99 | 201.95 | 34.95 | 0.00 | 114.59 | 62.52 | 89.94 | 154.17 | 0.00 |
| O14980 | Exportin-1 | **2.05** | **0.37** | 11.17 | 15.30 | 22.90 | 24.13 | 0.00 | 0.00 | 27.93 | 27.93 | 0.00 | 0.00 | 35.61 | 0.00 | 9.50 | 31.34 | 60.98 |
| P11940 | Polyadenylate-binding protein 1 | **1.48** | **0.37** | 60.07 | 40.81 | 89.18 | 58.31 | 80.38 | 0.00 | 79.56 | 102.28 | 38.15 | 96.64 | 99.57 | 0.00 | 44.30 | 139.75 | 154.82 |
| O00231 | 26S proteasome non-ATPase regulatory subunit 11 | **1.68** | **0.37** | 20.73 | 27.01 | 34.90 | 23.32 | 25.40 | 0.00 | 13.08 | 65.20 | 0.00 | 13.94 | 56.77 | 52.15 | 0.00 | 34.75 | 51.78 |
| Q15293 | Reticulocalbin-1 | **0.50** | **0.37** | 148.35 | 195.37 | 73.60 | 29.09 | 488.44 | 125.87 | 54.13 | 73.30 | 0.00 | 107.52 | 69.57 | 57.38 | 54.83 | 111.05 | 41.27 |
| P11047 | Laminin subunit gamma-1 | **1.63** | **0.38** | 67.11 | 59.59 | 109.07 | 84.16 | 64.14 | 49.32 | 169.39 | 33.33 | 19.37 | 65.84 | 103.32 | 246.16 | 49.65 | 168.14 | 21.33 |
| Q92522 | Histone H1x | **1.61** | **0.38** | 73.20 | 39.77 | 117.78 | 99.77 | 28.94 | 124.95 | 94.17 | 79.49 | 38.43 | 79.40 | 176.92 | 0.00 | 45.22 | 127.42 | 277.69 |
| Q13268 | Dehydrogenase/reductase SDR family member 2, mitochondrial | **0.45** | **0.38** | 672.55 | 759.93 | 301.36 | 559.78 | 916.63 | 182.85 | 1906.34 | 186.20 | 170.74 | 1435.37 | 79.50 | 178.98 | 0.00 | 114.32 | 0.00 |
| P51884 | Lumican | **1.85** | **0.38** | 484.90 | 833.44 | 898.16 | 642.54 | 267.35 | 1966.53 | 104.33 | 63.11 | 23.18 | 398.85 | 900.60 | 1772.25 | 1494.73 | 743.63 | 78.92 |
| P38646 | Stress-70 protein, mitochondrial | **1.42** | **0.38** | 108.83 | 51.71 | 154.69 | 98.95 | 133.27 | 38.46 | 165.93 | 133.42 | 73.04 | 260.46 | 107.04 | 76.32 | 40.79 | 160.99 | 282.52 |
| O95876 | WD repeat-containing and planar cell polarity effector protein fritz homolog | **1.46** | **0.38** | 270.90 | 248.62 | 395.02 | 195.70 | 486.84 | 0.00 | 453.28 | 414.38 | 0.00 | 476.38 | 0.00 | 460.80 | 487.36 | 428.69 | 516.89 |
| Q8WV92 | MIT domain-containing protein 1 | **2.47** | **0.38** | 31.06 | 29.00 | 76.58 | 112.06 | 15.00 | 0.00 | 27.58 | 76.98 | 35.73 | 42.99 | 139.73 | 0.00 | 0.00 | 0.00 | 276.77 |
| Q2TB90 | Putative hexokinase HKDC1 | **1.43** | **0.38** | 43.75 | 27.28 | 62.74 | 38.17 | 59.73 | 0.00 | 68.60 | 54.85 | 35.57 | 85.82 | 127.38 | 62.55 | 30.50 | 33.17 | 37.05 |
| P05976 | Myosin light chain 1/3, skeletal muscle isoform | **1.36** | **0.38** | 150.12 | 123.90 | 204.87 | 68.86 | 367.16 | 114.10 | 121.01 | 91.80 | 56.55 | 183.93 | 226.59 | 269.44 | 281.86 | 169.54 | 97.87 |
| P08590 | Myosin light chain 3 | **1.36** | **0.38** | 150.12 | 123.90 | 204.87 | 68.86 | 367.16 | 114.10 | 121.01 | 91.80 | 56.55 | 183.93 | 226.59 | 269.44 | 281.86 | 169.54 | 97.87 |
| P24666 | Low molecular weight phosphotyrosine protein phosphatase | **0.09** | **0.38** | 352.45 | 723.04 | 31.68 | 49.78 | 1644.62 | 0.00 | 75.37 | 0.00 | 42.27 | 39.20 | 23.01 | 0.00 | 0.00 | 127.85 | 0.00 |
| Q02543 | 60S ribosomal protein L18a | **1.55** | **0.38** | 50.17 | 47.06 | 77.69 | 50.62 | 34.02 | 0.00 | 93.51 | 106.03 | 17.31 | 56.39 | 85.51 | 0.00 | 59.85 | 133.88 | 130.49 |
| O75340 | Programmed cell death protein 6 | **0.67** | **0.38** | 42.45 | 31.44 | 28.55 | 17.76 | 88.17 | 0.00 | 39.99 | 47.28 | 36.82 | 22.67 | 44.67 | 0.00 | 23.40 | 31.19 | 49.39 |
| O60361 | Putative nucleoside diphosphate kinase | **1.23** | **0.38** | 410.97 | 161.94 | 504.77 | 172.01 | 424.03 | 158.87 | 563.17 | 538.74 | 370.02 | 529.45 | 746.77 | 370.68 | 256.03 | 595.77 | 529.94 |
| P42766 | 60S ribosomal protein L35 | **1.60** | **0.38** | 150.91 | 181.37 | 241.12 | 143.93 | 0.00 | 59.12 | 339.70 | 355.75 | 0.00 | 322.88 | 286.61 | 352.31 | 132.17 | 0.00 | 352.75 |
| Q9C0C2 | 182 kDa tankyrase-1-binding protein | **3.48** | **0.38** | 12.36 | 11.53 | 42.95 | 73.04 | 24.22 | 0.00 | 0.00 | 17.49 | 20.08 | 20.72 | 186.44 | 0.00 | 0.00 | 0.00 | 50.51 |
| P43487 | Ran-specific GTPase-activating protein | **1.50** | **0.38** | 42.97 | 39.52 | 64.38 | 37.57 | 63.85 | 0.00 | 75.87 | 75.14 | 0.00 | 113.70 | 34.95 | 34.60 | 24.78 | 82.93 | 95.34 |
| Q96JP2 | Unconventional myosin-XVB | **0.53** | **0.38** | 798.81 | 929.62 | 423.40 | 362.07 | 2270.99 | 0.00 | 913.56 | 0.00 | 809.49 | 0.00 | 561.44 | 779.24 | 388.93 | 810.80 | 0.00 |
| Q92804 | TATA-binding protein-associated factor 2N | **0.75** | **0.38** | 100.18 | 20.81 | 75.24 | 61.09 | 128.36 | 95.91 | 108.65 | 96.74 | 71.26 | 92.28 | 94.78 | 0.00 | 0.00 | 122.60 | 141.77 |
| P26232 | Catenin alpha-2 | **0.67** | **0.38** | 52.70 | 31.02 | 35.08 | 32.46 | 52.99 | 0.00 | 70.11 | 61.35 | 79.06 | 75.13 | 43.34 | 0.00 | 0.00 | 24.83 | 67.17 |
| Q96A26 | Protein FAM162A | **0.65** | **0.38** | 126.05 | 42.99 | 81.78 | 106.71 | 185.17 | 139.11 | 94.74 | 136.55 | 74.69 | 246.26 | 179.44 | 0.00 | 0.00 | 0.00 | 64.96 |
| P27635 | 60S ribosomal protein L10 | **1.77** | **0.39** | 17.15 | 15.91 | 30.30 | 30.36 | 29.94 | 0.00 | 31.75 | 24.06 | 0.00 | 12.69 | 40.39 | 0.00 | 0.00 | 59.90 | 68.83 |
| P18510 | Interleukin-1 receptor antagonist protein | **0.50** | **0.39** | 45.58 | 47.29 | 22.79 | 35.70 | 108.15 | 0.00 | 75.15 | 44.58 | 0.00 | 0.00 | 59.95 | 0.00 | 0.00 | 0.00 | 76.78 |
| P63244 | Guanine nucleotide-binding protein subunit beta-2-like 1 | **1.32** | **0.39** | 119.99 | 81.17 | 158.88 | 60.30 | 85.89 | 42.89 | 199.13 | 214.99 | 57.05 | 169.68 | 174.65 | 115.92 | 95.27 | 132.84 | 264.94 |
| P61981 | 14-3-3 protein gamma | **1.19** | **0.39** | 1025.02 | 315.08 | 1216.02 | 367.51 | 1157.52 | 463.21 | 1177.24 | 1197.87 | 1129.25 | 1347.69 | 983.24 | 1011.22 | 789.06 | 1346.91 | 1818.02 |
| Q99426 | Tubulin-folding cofactor B | **0.52** | **0.39** | 17.54 | 16.42 | 9.13 | 14.22 | 35.17 | 0.00 | 25.85 | 26.66 | 0.00 | 29.77 | 0.00 | 0.00 | 0.00 | 0.00 | 24.99 |
| P09622 | Dihydrolipoyl dehydrogenase, mitochondrial | **0.69** | **0.39** | 67.13 | 41.94 | 46.44 | 33.66 | 70.83 | 0.00 | 116.23 | 73.06 | 75.51 | 49.85 | 52.91 | 0.00 | 30.11 | 42.97 | 102.79 |
| P07305 | Histone H1.0 | **1.86** | **0.39** | 83.37 | 54.07 | 155.19 | 168.82 | 65.72 | 0.00 | 111.05 | 142.15 | 97.94 | 0.00 | 129.22 | 142.49 | 202.10 | 0.00 | 457.33 |
| P40925 | Malate dehydrogenase, cytoplasmic | **1.27** | **0.39** | 204.18 | 139.14 | 258.66 | 47.12 | 225.54 | 0.00 | 375.69 | 263.76 | 155.90 | 217.49 | 265.65 | 271.78 | 188.51 | 311.47 | 297.08 |
| P02671 | Fibrinogen alpha chain | **1.87** | **0.39** | 329.30 | 176.00 | 614.83 | 681.77 | 217.15 | 499.48 | 537.88 | 155.20 | 236.80 | 566.21 | 203.80 | 1916.21 | 718.79 | 166.56 | 117.41 |
| Q99575 | Ribonucleases P/MRP protein subunit POP1 | **1.82** | **0.39** | 616.24 | 859.45 | 1123.14 | 978.80 | 0.00 | 0.00 | 1309.91 | 1771.30 | 0.00 | 2420.07 | 1485.48 | 0.00 | 1041.33 | 1791.97 | 0.00 |
| O94826 | Mitochondrial import receptor subunit TOM70 | **0.67** | **0.39** | 22.38 | 14.66 | 14.93 | 12.79 | 38.60 | 0.00 | 20.10 | 21.36 | 31.83 | 27.44 | 15.25 | 0.00 | 0.00 | 29.37 | 17.55 |
| Q15393 | Splicing factor 3B subunit 3 | **1.71** | **0.39** | 19.27 | 19.73 | 32.92 | 28.59 | 0.00 | 0.00 | 33.70 | 43.87 | 18.80 | 33.25 | 47.29 | 0.00 | 0.00 | 44.24 | 72.71 |
| Q9HDC9 | Adipocyte plasma membrane-associated protein | **0.46** | **0.39** | 140.28 | 204.70 | 64.32 | 40.57 | 41.24 | 0.00 | 67.93 | 90.77 | 501.44 | 51.99 | 138.79 | 82.63 | 39.30 | 32.39 | 40.83 |
| Q8IYT4 | Katanin p60 ATPase-containing subunit A-like 2 | **1.39** | **0.39** | 106.79 | 35.05 | 148.54 | 98.14 | 98.24 | 59.08 | 145.31 | 137.15 | 94.15 | 101.73 | 294.66 | 184.74 | 0.00 | 130.98 | 179.15 |
| P31942 | Heterogeneous nuclear ribonucleoprotein H3 | **0.52** | **0.39** | 31.83 | 26.90 | 16.61 | 29.02 | 21.03 | 0.00 | 73.78 | 33.53 | 30.82 | 28.65 | 0.00 | 0.00 | 0.00 | 71.04 | 0.00 |
| Q06323 | Proteasome activator complex subunit 1 | **0.70** | **0.39** | 227.87 | 158.33 | 159.12 | 44.02 | 440.37 | 347.89 | 97.50 | 167.28 | 86.29 | 148.19 | 175.42 | 222.75 | 185.39 | 115.29 | 107.71 |
| Q9Y2X3 | Nucleolar protein 58 | **1.92** | **0.40** | 14.89 | 20.90 | 28.55 | 28.23 | 0.00 | 0.00 | 43.75 | 30.69 | 0.00 | 36.98 | 34.82 | 0.00 | 0.00 | 23.81 | 75.69 |
| Q93084 | Sarcoplasmic/endoplasmic reticulum calcium ATPase 3 | **1.56** | **0.40** | 41.47 | 39.18 | 64.68 | 45.68 | 0.00 | 0.00 | 70.97 | 82.39 | 53.97 | 59.56 | 69.52 | 57.15 | 0.00 | 58.96 | 142.86 |
| Q9BPW8 | Protein NipSnap homolog 1 | **0.52** | **0.40** | 24.21 | 22.81 | 12.68 | 20.05 | 31.19 | 0.00 | 45.58 | 44.28 | 0.00 | 0.00 | 0.00 | 0.00 | 0.00 | 44.32 | 31.78 |
| P62829 | 60S ribosomal protein L23 | **1.39** | **0.40** | 96.96 | 69.86 | 135.22 | 72.30 | 92.36 | 0.00 | 138.50 | 184.29 | 69.64 | 91.24 | 106.30 | 86.67 | 90.20 | 167.84 | 269.07 |
| Q96E39 | RNA binding motif protein, X-linked-like-1 | **1.25** | **0.40** | 100.13 | 50.08 | 125.30 | 44.19 | 65.34 | 62.94 | 156.68 | 153.25 | 62.44 | 94.52 | 119.78 | 138.90 | 58.76 | 179.68 | 160.17 |
| Q9UMS4 | Pre-mRNA-processing factor 19 | **1.78** | **0.40** | 24.09 | 33.35 | 42.84 | 36.38 | 0.00 | 0.00 | 67.20 | 53.24 | 0.00 | 36.21 | 75.83 | 0.00 | 0.00 | 77.83 | 67.14 |
| P24534 | Elongation factor 1-beta | **0.75** | **0.40** | 108.31 | 41.51 | 81.77 | 55.59 | 169.88 | 80.11 | 97.86 | 127.96 | 65.74 | 78.79 | 0.00 | 76.63 | 62.56 | 101.57 | 171.04 |
| O75828 | Carbonyl reductase [NADPH] 3 | **1.25** | **0.40** | 50.87 | 33.68 | 63.84 | 12.83 | 75.07 | 0.00 | 84.66 | 57.25 | 37.38 | 45.54 | 64.14 | 75.26 | 57.00 | 60.01 | 81.07 |
| P34932 | Heat shock 70 kDa protein 4 | **0.65** | **0.40** | 59.22 | 42.89 | 38.50 | 35.50 | 100.99 | 0.00 | 74.70 | 90.75 | 29.65 | 54.94 | 27.00 | 0.00 | 0.00 | 61.51 | 87.53 |
| Q9Y6C2 | EMILIN-1 | **0.49** | **0.41** | 228.58 | 314.90 | 112.36 | 90.18 | 72.46 | 286.06 | 29.41 | 754.95 | 0.00 | 250.41 | 62.22 | 184.45 | 80.26 | 96.79 | 0.00 |
| P62258 | 14-3-3 protein epsilon | **1.18** | **0.41** | 1086.60 | 368.45 | 1286.96 | 389.63 | 1253.95 | 498.83 | 1347.43 | 1377.09 | 955.72 | 1452.31 | 1080.45 | 1052.25 | 808.88 | 1417.74 | 1910.11 |
| P57721 | Poly(rC)-binding protein 3 | **1.18** | **0.41** | 221.96 | 90.47 | 262.76 | 65.08 | 258.83 | 95.75 | 317.42 | 275.11 | 162.70 | 277.50 | 270.57 | 270.19 | 148.80 | 258.26 | 351.27 |
| P33241 | Lymphocyte-specific protein 1 | **0.41** | **0.41** | 58.68 | 74.13 | 23.98 | 58.74 | 75.22 | 0.00 | 0.00 | 178.87 | 39.30 | 0.00 | 143.89 | 0.00 | 0.00 | 0.00 | 0.00 |
| Q15019 | Septin-2 | **0.73** | **0.41** | 70.23 | 47.50 | 51.31 | 23.12 | 63.94 | 0.00 | 133.07 | 74.89 | 79.25 | 33.92 | 54.67 | 30.17 | 36.68 | 91.35 | 61.04 |
| P06753 | Tropomyosin alpha-3 chain | **1.55** | **0.41** | 610.88 | 417.56 | 946.54 | 771.81 | 1194.95 | 900.35 | 297.06 | 424.21 | 237.84 | 390.47 | 828.18 | 2348.56 | 1261.48 | 578.42 | 272.13 |
| B0FP48 | Uroplakin-3b-like protein | **0.30** | **0.41** | 122.33 | 226.00 | 36.13 | 88.51 | 0.00 | 0.00 | 524.38 | 56.79 | 30.49 | 216.79 | 0.00 | 0.00 | 0.00 | 0.00 | 0.00 |
| Q10570 | Cleavage and polyadenylation specificity factor subunit 1 | **0.48** | **0.41** | 899.87 | 1243.83 | 430.36 | 452.79 | 111.26 | 0.00 | 866.66 | 481.35 | 3040.09 | 126.54 | 97.91 | 39.27 | 1232.54 | 558.54 | 527.39 |
| P20339 | Ras-related protein Rab-5A | **0.90** | **0.41** | 62.57 | 9.19 | 56.28 | 13.86 | 64.53 | 48.71 | 62.71 | 74.48 | 62.44 | 65.44 | 45.28 | 47.38 | 45.90 | 79.98 | 53.71 |
| Q13242 | Serine/arginine-rich splicing factor 9 | **0.45** | **0.41** | 12.59 | 11.97 | 5.71 | 13.99 | 20.79 | 0.00 | 25.81 | 16.33 | 0.00 | 0.00 | 0.00 | 0.00 | 0.00 | 0.00 | 34.27 |
| Q8WUM4 | Programmed cell death 6-interacting protein | **0.77** | **0.41** | 51.71 | 31.58 | 39.56 | 13.08 | 83.49 | 0.00 | 67.01 | 59.56 | 48.48 | 43.99 | 46.71 | 33.06 | 23.98 | 29.90 | 59.72 |
| A6NIZ1 | Ras-related protein Rap-1b-like protein | **1.44** | **0.41** | 60.17 | 56.97 | 86.94 | 46.44 | 107.60 | 0.00 | 76.21 | 117.05 | 0.00 | 28.02 | 85.56 | 158.36 | 118.12 | 78.87 | 52.69 |
| P18206 | Vinculin | **1.43** | **0.41** | 153.32 | 69.14 | 218.84 | 157.70 | 231.84 | 108.75 | 224.15 | 116.12 | 85.73 | 145.36 | 155.63 | 477.34 | 328.26 | 171.91 | 34.57 |
| Q14112 | Nidogen-2 | **2.23** | **0.42** | 29.49 | 65.95 | 65.78 | 73.24 | 0.00 | 0.00 | 147.46 | 0.00 | 0.00 | 58.46 | 34.94 | 55.54 | 36.67 | 209.08 | 0.00 |
| Q96M86 | Dynein heavy chain domain-containing protein 1 | **1.51** | **0.42** | 643.21 | 774.98 | 969.00 | 310.28 | 387.00 | 0.00 | 1014.86 | 1814.19 | 0.00 | 731.75 | 578.40 | 1219.76 | 1207.32 | 1305.99 | 770.81 |
| P02652 | Apolipoprotein A-II | **0.50** | **0.42** | 274.97 | 374.11 | 137.72 | 126.14 | 126.77 | 940.85 | 124.94 | 44.44 | 137.83 | 120.41 | 60.98 | 364.42 | 178.95 | 101.54 | 0.00 |
| P17096 | High mobility group protein HMG-I/HMG-Y | **0.59** | **0.42** | 122.27 | 115.35 | 72.15 | 80.58 | 0.00 | 0.00 | 156.29 | 227.63 | 227.45 | 172.85 | 128.16 | 0.00 | 0.00 | 0.00 | 131.89 |
| P48637 | Glutathione synthetase | **0.58** | **0.42** | 23.58 | 22.85 | 13.71 | 15.72 | 45.89 | 0.00 | 26.80 | 45.23 | 0.00 | 35.81 | 0.00 | 0.00 | 24.20 | 0.00 | 22.24 |
| P51572 | B-cell receptor-associated protein 31 | **1.34** | **0.42** | 162.40 | 46.91 | 218.36 | 140.41 | 234.25 | 120.11 | 120.67 | 164.40 | 172.55 | 288.27 | 475.07 | 141.52 | 112.08 | 134.68 | 158.55 |
| Q13404 | Ubiquitin-conjugating enzyme E2 variant 1 | **1.19** | **0.42** | 61.03 | 22.19 | 72.37 | 22.08 | 85.73 | 43.13 | 47.53 | 84.81 | 43.95 | 78.87 | 76.98 | 46.75 | 50.41 | 73.71 | 107.49 |
| O15020 | Spectrin beta chain, non-erythrocytic 2 | **6.79** | **0.42** | 49.98 | 111.75 | 339.47 | 751.98 | 0.00 | 0.00 | 0.00 | 249.88 | 0.00 | 1871.92 | 116.48 | 0.00 | 0.00 | 27.30 | 21.13 |
| Q14160 | Protein scribble homolog | **1.57** | **0.42** | 96.12 | 91.84 | 151.36 | 119.70 | 119.91 | 0.00 | 164.44 | 196.27 | 0.00 | 258.22 | 217.31 | 0.00 | 0.00 | 187.89 | 244.74 |
| Q01518 | Adenylyl cyclase-associated protein 1 | **1.89** | **0.42** | 261.68 | 84.76 | 493.65 | 606.67 | 290.40 | 127.45 | 322.74 | 334.76 | 233.02 | 169.01 | 173.01 | 335.64 | 286.69 | 272.86 | 1724.70 |
| O15230 | Laminin subunit alpha-5 | **2.35** | **0.42** | 8.67 | 8.87 | 20.40 | 29.97 | 13.91 | 0.00 | 20.37 | 0.00 | 9.06 | 0.00 | 24.35 | 77.20 | 0.00 | 20.88 | 0.00 |
| Q16543 | Hsp90 co-chaperone Cdc37 | **1.24** | **0.42** | 48.95 | 19.23 | 60.94 | 26.55 | 47.29 | 20.28 | 49.20 | 74.05 | 53.93 | 55.50 | 73.02 | 60.60 | 21.92 | 51.88 | 102.71 |
| Q07866 | Kinesin light chain 1 | **0.14** | **0.42** | 106.59 | 229.72 | 15.16 | 14.80 | 0.00 | 0.00 | 15.61 | 517.35 | 0.00 | 11.69 | 22.36 | 0.00 | 0.00 | 18.00 | 38.91 |
| Q9H0B6 | Kinesin light chain 2 | **0.14** | **0.42** | 106.59 | 229.72 | 15.16 | 14.80 | 0.00 | 0.00 | 15.61 | 517.35 | 0.00 | 11.69 | 22.36 | 0.00 | 0.00 | 18.00 | 38.91 |
| Q15459 | Splicing factor 3A subunit 1 | **1.57** | **0.43** | 20.77 | 19.58 | 32.57 | 26.02 | 26.65 | 0.00 | 38.01 | 39.20 | 0.00 | 54.80 | 46.21 | 0.00 | 0.00 | 38.45 | 55.97 |
| P41252 | Isoleucine--tRNA ligase, cytoplasmic | **1.77** | **0.43** | 9.05 | 12.51 | 16.02 | 14.81 | 0.00 | 0.00 | 25.06 | 20.18 | 0.00 | 20.52 | 17.59 | 0.00 | 18.43 | 0.00 | 39.58 |
| O15061 | Synemin | **0.74** | **0.43** | 453.56 | 299.47 | 333.93 | 172.01 | 751.87 | 620.30 | 582.28 | 313.36 | 0.00 | 476.38 | 37.61 | 248.93 | 487.36 | 428.69 | 324.62 |
| Q9BZK3 | Putative nascent polypeptide-associated complex subunit alpha-like protein | **1.67** | **0.43** | 24.09 | 33.72 | 40.33 | 31.20 | 0.00 | 0.00 | 50.36 | 70.10 | 0.00 | 22.05 | 59.64 | 0.00 | 20.03 | 57.00 | 83.28 |
| P57735 | Ras-related protein Rab-25 | **1.57** | **0.43** | 32.30 | 32.24 | 50.65 | 39.62 | 61.52 | 0.00 | 0.00 | 67.19 | 32.78 | 71.54 | 77.93 | 0.00 | 0.00 | 69.19 | 85.22 |
| P12036 | Neurofilament heavy polypeptide | **1.56** | **0.43** | 710.83 | 736.39 | 1112.15 | 849.36 | 777.83 | 1937.06 | 542.04 | 160.39 | 136.84 | 469.50 | 829.76 | 1492.54 | 2640.86 | 892.65 | 347.61 |
| O14983 | Sarcoplasmic/endoplasmic reticulum calcium ATPase 1 | **1.42** | **0.43** | 41.50 | 41.37 | 59.07 | 29.21 | 0.00 | 0.00 | 69.67 | 92.41 | 45.43 | 51.18 | 39.27 | 43.64 | 33.02 | 77.78 | 109.53 |
| P07384 | Calpain-1 catalytic subunit | **0.75** | **0.43** | 191.14 | 72.56 | 144.00 | 108.96 | 238.19 | 71.99 | 253.34 | 215.25 | 176.95 | 175.72 | 187.42 | 61.57 | 48.84 | 62.25 | 328.21 |
| P08134 | Rho-related GTP-binding protein RhoC | **1.28** | **0.43** | 104.12 | 67.77 | 132.78 | 47.86 | 147.38 | 76.63 | 125.98 | 170.62 | 0.00 | 89.07 | 217.10 | 86.55 | 122.08 | 136.06 | 145.85 |
| P61020 | Ras-related protein Rab-5B | **0.90** | **0.43** | 62.92 | 9.50 | 56.84 | 14.05 | 65.50 | 48.71 | 62.71 | 75.25 | 62.44 | 65.44 | 43.97 | 47.38 | 45.90 | 79.98 | 58.35 |
| P21810 | Biglycan | **1.62** | **0.43** | 181.36 | 222.19 | 294.03 | 231.60 | 166.19 | 560.85 | 132.33 | 47.43 | 0.00 | 132.99 | 160.30 | 651.21 | 379.75 | 418.05 | 21.88 |
| Q02790 | Peptidyl-prolyl cis-trans isomerase FKBP4 | **1.86** | **0.44** | 38.12 | 22.85 | 70.76 | 86.34 | 33.48 | 0.00 | 50.48 | 52.83 | 53.79 | 87.47 | 26.20 | 32.93 | 0.00 | 40.81 | 237.12 |
| P78527 | DNA-dependent protein kinase catalytic subunit | **1.44** | **0.44** | 30.68 | 34.19 | 44.03 | 19.67 | 19.67 | 0.00 | 61.79 | 71.95 | 0.00 | 53.92 | 48.35 | 27.46 | 13.37 | 55.18 | 65.91 |
| P04222 | HLA class I histocompatibility antigen, Cw-3 alpha chain | **1.58** | **0.44** | 139.05 | 95.79 | 220.21 | 204.25 | 222.13 | 258.51 | 94.67 | 82.94 | 37.01 | 37.32 | 232.12 | 581.58 | 291.66 | 139.47 | 39.11 |
| Q8N0Y7 | Probable phosphoglycerate mutase 4 | **0.83** | **0.44** | 434.81 | 211.74 | 360.89 | 69.23 | 450.09 | 90.71 | 624.45 | 591.13 | 417.68 | 312.85 | 295.97 | 285.79 | 422.92 | 425.88 | 421.95 |
| P51659 | Peroxisomal multifunctional enzyme type 2 | **0.61** | **0.44** | 34.23 | 35.16 | 20.92 | 18.34 | 74.14 | 0.00 | 31.15 | 65.86 | 0.00 | 25.55 | 32.07 | 0.00 | 21.30 | 0.00 | 46.61 |
| P02679 | Fibrinogen gamma chain | **1.51** | **0.44** | 531.50 | 323.01 | 804.90 | 691.91 | 380.20 | 695.36 | 1022.33 | 280.73 | 278.89 | 719.59 | 353.32 | 2060.53 | 1099.69 | 349.85 | 246.40 |
| Q9Y4G6 | Talin-2 | **1.58** | **0.44** | 62.72 | 65.21 | 99.22 | 81.74 | 58.17 | 0.00 | 146.06 | 109.36 | 0.00 | 51.92 | 72.15 | 224.79 | 166.07 | 80.42 | 0.00 |
| P20962 | Parathymosin | **1.62** | **0.44** | 60.21 | 85.88 | 97.39 | 67.92 | 207.59 | 0.00 | 55.85 | 37.61 | 0.00 | 26.95 | 54.58 | 140.45 | 198.78 | 124.49 | 39.07 |
| Q13838 | Spliceosome RNA helicase DDX39B | **1.30** | **0.44** | 100.38 | 56.77 | 130.47 | 65.50 | 68.40 | 45.45 | 160.22 | 163.43 | 64.40 | 107.20 | 96.81 | 81.66 | 70.63 | 220.40 | 206.15 |
| P52907 | F-actin-capping protein subunit alpha-1 | **0.88** | **0.44** | 124.45 | 37.96 | 109.22 | 24.69 | 138.07 | 114.40 | 101.38 | 182.95 | 85.43 | 120.07 | 89.50 | 105.63 | 83.04 | 105.15 | 151.95 |
| Q01844 | RNA-binding protein EWS | **0.78** | **0.44** | 105.22 | 45.79 | 81.58 | 50.69 | 168.37 | 106.20 | 98.40 | 113.38 | 39.76 | 113.48 | 103.29 | 0.00 | 36.98 | 111.62 | 124.14 |
| Q6UW68 | Transmembrane protein 205 | **0.48** | **0.44** | 34.76 | 32.48 | 16.64 | 40.76 | 64.81 | 0.00 | 62.24 | 46.77 | 0.00 | 0.00 | 0.00 | 0.00 | 0.00 | 0.00 | 99.85 |
| Q96KP4 | Cytosolic non-specific dipeptidase | **0.65** | **0.44** | 147.91 | 142.72 | 96.77 | 60.47 | 381.70 | 124.85 | 151.37 | 81.63 | 0.00 | 113.42 | 85.74 | 21.82 | 61.23 | 96.77 | 201.66 |
| P08962 | CD63 antigen | **0.58** | **0.44** | 124.25 | 132.09 | 71.98 | 83.68 | 0.00 | 209.75 | 0.00 | 110.06 | 301.45 | 95.69 | 182.81 | 153.39 | 0.00 | 0.00 | 0.00 |
| Q9Y6C9 | Mitochondrial carrier homolog 2 | **0.45** | **0.44** | 21.77 | 26.30 | 9.70 | 23.75 | 10.00 | 0.00 | 58.07 | 40.80 | 0.00 | 0.00 | 0.00 | 0.00 | 0.00 | 0.00 | 58.17 |
| P01774 | Ig heavy chain V-III region POM | **0.51** | **0.45** | 128.71 | 135.58 | 66.03 | 124.50 | 0.00 | 189.29 | 133.96 | 0.00 | 320.27 | 0.00 | 0.00 | 0.00 | 310.29 | 85.90 | 0.00 |
| P01776 | Ig heavy chain V-III region WAS | **0.51** | **0.45** | 128.71 | 135.58 | 66.03 | 124.50 | 0.00 | 189.29 | 133.96 | 0.00 | 320.27 | 0.00 | 0.00 | 0.00 | 310.29 | 85.90 | 0.00 |
| P01779 | Ig heavy chain V-III region TUR | **0.51** | **0.45** | 128.71 | 135.58 | 66.03 | 124.50 | 0.00 | 189.29 | 133.96 | 0.00 | 320.27 | 0.00 | 0.00 | 0.00 | 310.29 | 85.90 | 0.00 |
| Q9UI47 | Catenin alpha-3 | **0.71** | **0.45** | 63.42 | 36.16 | 45.26 | 38.70 | 87.88 | 0.00 | 81.81 | 79.23 | 68.15 | 73.81 | 87.85 | 0.00 | 0.00 | 38.02 | 71.87 |
| P62195 | 26S protease regulatory subunit 8 | **0.65** | **0.45** | 20.85 | 14.34 | 13.51 | 15.92 | 33.50 | 0.00 | 12.02 | 27.84 | 30.90 | 21.80 | 21.50 | 0.00 | 0.00 | 0.00 | 37.73 |
| P36952 | Serpin B5 | **0.54** | **0.45** | 58.19 | 48.87 | 31.68 | 59.29 | 50.43 | 0.00 | 92.85 | 120.81 | 26.83 | 0.00 | 42.43 | 0.00 | 0.00 | 0.00 | 147.65 |
| P62330 | ADP-ribosylation factor 6 | **0.62** | **0.45** | 33.00 | 23.72 | 20.36 | 28.12 | 52.01 | 0.00 | 38.89 | 56.19 | 17.91 | 62.28 | 10.32 | 0.00 | 0.00 | 0.00 | 49.56 |
| Q00325 | Phosphate carrier protein, mitochondrial | **1.25** | **0.45** | 153.28 | 98.19 | 192.07 | 63.57 | 162.78 | 0.00 | 236.03 | 239.77 | 127.82 | 264.05 | 198.14 | 135.43 | 97.07 | 233.49 | 224.25 |
| P60660 | Myosin light polypeptide 6 | **1.30** | **0.45** | 711.64 | 545.66 | 921.95 | 327.75 | 1654.17 | 661.30 | 528.43 | 444.24 | 270.06 | 731.61 | 1064.80 | 1329.70 | 1195.82 | 742.98 | 466.78 |
| P00751 | Complement factor B | **1.49** | **0.45** | 30.93 | 23.06 | 46.10 | 37.53 | 0.00 | 61.42 | 38.66 | 36.10 | 18.48 | 41.28 | 0.00 | 103.17 | 75.25 | 37.55 | 19.37 |
| O75477 | Erlin-1 | **1.56** | **0.46** | 24.09 | 34.76 | 37.53 | 22.19 | 0.00 | 0.00 | 44.71 | 75.74 | 0.00 | 42.94 | 59.49 | 28.25 | 58.81 | 0.00 | 35.71 |
| Q9UNZ2 | NSFL1 cofactor p47 | **0.61** | **0.46** | 25.11 | 23.10 | 15.44 | 18.16 | 43.25 | 45.00 | 37.29 | 0.00 | 0.00 | 19.36 | 39.80 | 0.00 | 0.00 | 0.00 | 33.49 |
| P48552 | Nuclear receptor-interacting protein 1 | **0.55** | **0.46** | 98.42 | 104.53 | 54.25 | 84.12 | 208.68 | 206.62 | 76.80 | 0.00 | 0.00 | 157.00 | 0.00 | 0.00 | 0.00 | 0.00 | 168.49 |
| Q99733 | Nucleosome assembly protein 1-like 4 | **0.66** | **0.46** | 46.49 | 27.45 | 30.59 | 38.14 | 71.66 | 0.00 | 52.89 | 59.76 | 48.16 | 46.47 | 0.00 | 0.00 | 0.00 | 94.37 | 42.69 |
| P56537 | Eukaryotic translation initiation factor 6 | **1.72** | **0.46** | 36.06 | 36.48 | 61.96 | 71.14 | 34.93 | 0.00 | 77.12 | 68.23 | 0.00 | 99.33 | 110.06 | 0.00 | 0.00 | 0.00 | 162.35 |
| Q13596 | Sorting nexin-1 | **1.24** | **0.46** | 24.92 | 14.09 | 30.80 | 11.22 | 30.70 | 0.00 | 32.40 | 27.92 | 33.58 | 52.27 | 22.77 | 32.80 | 27.78 | 22.01 | 27.18 |
| O14979 | Heterogeneous nuclear ribonucleoprotein D-like | **0.84** | **0.46** | 132.05 | 43.70 | 110.65 | 47.38 | 100.56 | 173.59 | 160.17 | 154.23 | 71.72 | 143.91 | 54.70 | 69.07 | 81.32 | 160.68 | 154.22 |
| P18621 | 60S ribosomal protein L17 | **1.65** | **0.46** | 50.72 | 70.40 | 83.45 | 70.05 | 0.00 | 0.00 | 143.01 | 110.61 | 0.00 | 129.56 | 82.00 | 0.00 | 0.00 | 167.06 | 122.07 |
| Q9BXT5 | Testis-expressed sequence 15 protein | **1.63** | **0.46** | 32.98 | 45.20 | 53.80 | 44.15 | 79.51 | 0.00 | 0.00 | 85.37 | 0.00 | 75.51 | 107.35 | 0.00 | 76.85 | 63.11 | 0.00 |
| Q9UBU9 | Nuclear RNA export factor 1 | **1.63** | **0.46** | 32.98 | 45.20 | 53.80 | 44.15 | 79.51 | 0.00 | 0.00 | 85.37 | 0.00 | 75.51 | 107.35 | 0.00 | 76.85 | 63.11 | 0.00 |
| O15347 | High mobility group protein B3 | **0.68** | **0.46** | 112.68 | 73.04 | 76.94 | 79.47 | 139.86 | 0.00 | 201.28 | 115.97 | 106.28 | 82.27 | 59.50 | 0.00 | 0.00 | 106.88 | 213.00 |
| P05090 | Apolipoprotein D | **1.75** | **0.46** | 20.41 | 28.03 | 35.71 | 36.35 | 53.98 | 0.00 | 0.00 | 0.00 | 48.08 | 43.71 | 39.30 | 98.71 | 32.55 | 0.00 | 0.00 |
| P51148 | Ras-related protein Rab-5C | **1.13** | **0.46** | 89.64 | 22.65 | 100.87 | 25.28 | 106.15 | 58.48 | 93.41 | 114.25 | 75.92 | 97.38 | 134.61 | 106.13 | 61.37 | 117.85 | 87.86 |
| Q9NZD2 | Glycolipid transfer protein | **1.69** | **0.46** | 48.97 | 35.03 | 82.55 | 91.79 | 81.56 | 0.00 | 29.56 | 52.15 | 81.56 | 130.50 | 31.97 | 0.00 | 0.00 | 235.61 | 97.25 |
| P20648 | Potassium-transporting ATPase alpha chain 1 | **1.30** | **0.46** | 65.30 | 47.51 | 84.58 | 36.09 | 72.14 | 0.00 | 129.60 | 79.21 | 45.57 | 135.73 | 112.75 | 78.30 | 51.25 | 40.62 | 88.86 |
| O94905 | Erlin-2 | **0.71** | **0.46** | 35.46 | 24.29 | 25.08 | 20.75 | 36.40 | 0.00 | 44.71 | 66.75 | 29.45 | 38.42 | 50.32 | 28.25 | 0.00 | 0.00 | 33.46 |
| P02675 | Fibrinogen beta chain | **1.60** | **0.46** | 520.79 | 282.01 | 833.57 | 867.81 | 318.42 | 837.71 | 796.38 | 214.68 | 436.75 | 750.64 | 266.38 | 2443.13 | 1109.18 | 277.61 | 154.49 |
| Q12906 | Interleukin enhancer-binding factor 3 | **1.36** | **0.46** | 75.41 | 72.96 | 102.42 | 43.16 | 41.85 | 0.00 | 141.69 | 164.48 | 29.02 | 131.98 | 126.56 | 67.16 | 31.29 | 119.65 | 137.91 |
| P62873 | Guanine nucleotide-binding protein G(I)/G(S)/G(T) subunit beta-1 | **1.27** | **0.46** | 70.92 | 51.35 | 90.27 | 20.36 | 37.23 | 0.00 | 96.28 | 127.85 | 93.24 | 114.32 | 67.93 | 100.44 | 67.33 | 83.46 | 108.15 |
| Q9Y277 | Voltage-dependent anion-selective channel protein 3 | **0.79** | **0.47** | 139.00 | 69.17 | 110.01 | 57.12 | 229.04 | 61.02 | 181.95 | 139.70 | 83.28 | 149.78 | 54.35 | 69.51 | 53.03 | 179.42 | 153.96 |
| Q00839 | Heterogeneous nuclear ribonucleoprotein U | **1.27** | **0.47** | 285.46 | 158.47 | 362.21 | 172.85 | 208.61 | 138.36 | 463.47 | 450.13 | 166.74 | 373.25 | 474.16 | 174.70 | 142.29 | 424.57 | 584.30 |
| P02545 | Prelamin-A/C | **0.89** | **0.47** | 560.97 | 128.27 | 499.72 | 136.54 | 569.64 | 761.27 | 576.78 | 456.04 | 441.14 | 485.69 | 340.72 | 373.74 | 688.30 | 483.50 | 626.37 |
| O60749 | Sorting nexin-2 | **1.35** | **0.47** | 25.28 | 23.92 | 34.20 | 9.00 | 50.88 | 0.00 | 33.12 | 42.42 | 0.00 | 47.80 | 30.06 | 32.80 | 27.78 | 24.48 | 42.28 |
| Q9UIJ7 | GTP:AMP phosphotransferase AK3, mitochondrial | **0.63** | **0.47** | 21.14 | 19.60 | 13.27 | 14.79 | 30.30 | 0.00 | 35.44 | 39.96 | 0.00 | 0.00 | 22.30 | 30.87 | 0.00 | 0.00 | 26.48 |
| Q9UHD9 | Ubiquilin-2 | **0.77** | **0.47** | 18.69 | 10.69 | 14.31 | 8.47 | 23.01 | 19.80 | 0.00 | 24.90 | 25.72 | 25.92 | 17.51 | 13.91 | 0.00 | 16.31 | 12.21 |
| P37802 | Transgelin-2 | **0.86** | **0.47** | 528.62 | 92.56 | 456.18 | 194.19 | 651.19 | 507.87 | 562.51 | 525.83 | 395.72 | 484.34 | 328.18 | 457.31 | 742.92 | 551.21 | 173.12 |
| Q7Z6G3 | N-terminal EF-hand calcium-binding protein 2 | **0.65** | **0.47** | 121.53 | 71.53 | 78.61 | 108.05 | 120.63 | 183.94 | 150.86 | 152.21 | 0.00 | 70.10 | 0.00 | 0.00 | 0.00 | 271.19 | 130.39 |
| Q9NY33 | Dipeptidyl peptidase 3 | **0.67** | **0.47** | 38.60 | 23.97 | 25.94 | 30.23 | 51.03 | 0.00 | 41.49 | 63.92 | 36.56 | 70.05 | 0.00 | 0.00 | 0.00 | 47.12 | 38.48 |
| Q02818 | Nucleobindin-1 | **0.60** | **0.47** | 37.68 | 41.54 | 22.42 | 24.81 | 106.43 | 0.00 | 12.48 | 41.92 | 27.56 | 50.64 | 44.28 | 0.00 | 0.00 | 0.00 | 39.60 |
| P49773 | Histidine triad nucleotide-binding protein 1 | **0.47** | **0.47** | 77.01 | 129.90 | 36.01 | 31.45 | 0.00 | 0.00 | 299.80 | 85.24 | 0.00 | 40.76 | 38.32 | 58.16 | 0.00 | 0.00 | 78.84 |
| P54136 | Arginine--tRNA ligase, cytoplasmic | **1.48** | **0.47** | 18.75 | 19.14 | 27.71 | 20.09 | 19.63 | 0.00 | 30.35 | 43.79 | 0.00 | 18.40 | 48.57 | 13.93 | 0.00 | 35.98 | 49.39 |
| Q07812 | Apoptosis regulator BAX | **0.61** | **0.47** | 28.05 | 28.70 | 17.06 | 19.71 | 65.54 | 0.00 | 28.92 | 45.78 | 0.00 | 0.00 | 40.74 | 0.00 | 22.75 | 0.00 | 38.86 |
| Q9Y265 | RuvB-like 1 | **1.58** | **0.47** | 20.39 | 22.87 | 32.12 | 27.92 | 12.76 | 0.00 | 40.58 | 48.60 | 0.00 | 67.39 | 30.87 | 0.00 | 0.00 | 39.08 | 55.36 |
| Q5IJ48 | Protein crumbs homolog 2 | **1.72** | **0.47** | 63.22 | 88.28 | 108.43 | 108.02 | 0.00 | 0.00 | 182.52 | 133.58 | 0.00 | 142.49 | 0.00 | 0.00 | 53.19 | 193.62 | 261.28 |
| Q99456 | Keratin, type I cytoskeletal 12 | **0.47** | **0.47** | 311.88 | 489.42 | 146.96 | 217.53 | 138.03 | 0.00 | 93.82 | 146.34 | 1181.18 | 561.01 | 0.00 | 0.00 | 0.00 | 162.64 | 158.12 |
| P52272 | Heterogeneous nuclear ribonucleoprotein M | **1.23** | **0.47** | 148.04 | 60.01 | 181.80 | 84.35 | 116.16 | 79.92 | 195.58 | 224.82 | 123.74 | 212.56 | 217.51 | 93.49 | 59.73 | 241.40 | 266.09 |
| P35555 | Fibrillin-1 | **1.56** | **0.48** | 73.58 | 96.20 | 114.41 | 85.59 | 90.07 | 232.14 | 45.67 | 0.00 | 0.00 | 73.43 | 115.68 | 260.04 | 101.09 | 136.24 | 0.00 |
| P04217 | Alpha-1B-glycoprotein | **1.64** | **0.48** | 27.98 | 31.21 | 45.84 | 45.27 | 0.00 | 75.88 | 0.00 | 30.93 | 33.07 | 37.68 | 0.00 | 108.52 | 90.49 | 38.37 | 0.00 |
| Q9UBV4 | Protein Wnt-16 | **0.58** | **0.48** | 43.11 | 41.74 | 24.87 | 39.51 | 91.33 | 0.00 | 51.93 | 72.27 | 0.00 | 0.00 | 60.74 | 0.00 | 0.00 | 0.00 | 88.45 |
| P01763 | Ig heavy chain V-III region WEA | **0.56** | **0.48** | 203.12 | 279.83 | 114.09 | 94.26 | 36.08 | 675.11 | 241.92 | 62.49 | 0.00 | 82.02 | 101.83 | 0.00 | 287.40 | 113.04 | 100.28 |
| P01767 | Ig heavy chain V-III region BUT | **0.56** | **0.48** | 203.12 | 279.83 | 114.09 | 94.26 | 36.08 | 675.11 | 241.92 | 62.49 | 0.00 | 82.02 | 101.83 | 0.00 | 287.40 | 113.04 | 100.28 |
| O75367 | Core histone macro-H2A.1 | **1.23** | **0.48** | 154.82 | 101.30 | 190.10 | 55.09 | 153.19 | 73.23 | 290.65 | 213.80 | 43.22 | 211.72 | 246.09 | 181.96 | 94.10 | 172.24 | 234.50 |
| P01781 | Ig heavy chain V-III region GAL | **1.53** | **0.48** | 43.09 | 64.85 | 65.83 | 36.32 | 0.00 | 145.78 | 69.69 | 0.00 | 0.00 | 53.10 | 60.55 | 44.88 | 139.03 | 45.59 | 51.80 |
| O75489 | NADH dehydrogenase [ubiquinone] iron-sulfur protein 3, mitochondrial | **0.57** | **0.48** | 25.04 | 24.50 | 14.32 | 23.78 | 30.99 | 0.00 | 55.39 | 38.79 | 0.00 | 0.00 | 29.41 | 0.00 | 0.00 | 56.50 | 0.00 |
| P30046 | D-dopachrome decarboxylase | **0.63** | **0.48** | 55.99 | 56.01 | 35.03 | 38.82 | 0.00 | 0.00 | 128.70 | 86.17 | 65.10 | 65.57 | 0.00 | 0.00 | 0.00 | 80.79 | 63.82 |
| P25705 | ATP synthase subunit alpha, mitochondrial | **1.24** | **0.48** | 304.71 | 171.63 | 379.03 | 163.61 | 334.18 | 82.70 | 433.67 | 493.04 | 179.94 | 613.76 | 361.37 | 320.32 | 171.35 | 279.94 | 527.45 |
| P32455 | Interferon-induced guanylate-binding protein 1 | **2.06** | **0.48** | 47.57 | 96.00 | 97.77 | 125.50 | 0.00 | 0.00 | 0.00 | 19.17 | 218.66 | 341.13 | 52.72 | 111.72 | 64.89 | 0.00 | 16.14 |
| Q9NRP0 | Oligosaccharyltransferase complex subunit | **0.60** | **0.49** | 28.17 | 28.05 | 16.95 | 23.17 | 33.91 | 0.00 | 42.39 | 64.55 | 0.00 | 0.00 | 0.00 | 15.36 | 28.29 | 58.08 | 0.00 |
| P31939 | Bifunctional purine biosynthesis protein PURH | **0.71** | **0.49** | 39.66 | 25.40 | 28.06 | 26.99 | 52.91 | 0.00 | 52.16 | 63.62 | 29.60 | 44.44 | 17.16 | 0.00 | 0.00 | 39.26 | 67.48 |
| Q92599 | Septin-8 | **0.65** | **0.49** | 50.04 | 41.34 | 32.73 | 37.57 | 0.00 | 41.41 | 90.34 | 94.33 | 24.12 | 46.77 | 0.00 | 82.14 | 0.00 | 0.00 | 67.48 |
| Q15365 | Poly(rC)-binding protein 1 | **1.19** | **0.49** | 269.38 | 133.15 | 321.89 | 107.90 | 324.85 | 95.75 | 424.56 | 331.25 | 170.50 | 293.38 | 348.71 | 259.75 | 189.88 | 329.77 | 509.85 |
| Q4VXU2 | Polyadenylate-binding protein 1-like | **1.49** | **0.49** | 35.28 | 33.58 | 52.64 | 43.97 | 46.25 | 0.00 | 57.14 | 73.00 | 0.00 | 67.02 | 55.94 | 0.00 | 0.00 | 102.37 | 90.52 |
| P09758 | Tumor-associated calcium signal transducer 2 | **0.72** | **0.49** | 71.59 | 43.14 | 51.75 | 46.98 | 82.73 | 0.00 | 106.61 | 102.29 | 66.35 | 51.70 | 100.96 | 0.00 | 0.00 | 49.10 | 108.76 |
| P14314 | Glucosidase 2 subunit beta | **0.88** | **0.49** | 216.32 | 51.30 | 190.66 | 63.77 | 255.35 | 239.68 | 185.77 | 259.64 | 141.16 | 254.63 | 268.44 | 120.67 | 124.62 | 209.16 | 166.47 |
| P26440 | Isovaleryl-CoA dehydrogenase, mitochondrial | **0.58** | **0.49** | 16.63 | 16.95 | 9.70 | 15.05 | 19.91 | 0.00 | 39.86 | 23.40 | 0.00 | 27.94 | 0.00 | 0.00 | 0.00 | 0.00 | 30.28 |
| P09668 | Pro-cathepsin H | **0.64** | **0.49** | 79.20 | 68.82 | 50.42 | 64.05 | 178.02 | 0.00 | 114.84 | 59.05 | 44.10 | 39.81 | 173.12 | 0.00 | 0.00 | 54.70 | 34.88 |
| O94973 | AP-2 complex subunit alpha-2 | **1.62** | **0.49** | 9.67 | 9.32 | 15.63 | 16.42 | 11.32 | 0.00 | 0.00 | 17.75 | 19.30 | 4.79 | 27.38 | 0.00 | 0.00 | 39.27 | 22.34 |
| P51858 | Hepatoma-derived growth factor | **1.39** | **0.49** | 47.03 | 29.30 | 65.31 | 50.23 | 68.53 | 0.00 | 67.89 | 61.89 | 36.83 | 48.08 | 129.24 | 54.29 | 0.00 | 38.87 | 121.39 |
| P47755 | F-actin-capping protein subunit alpha-2 | **1.19** | **0.49** | 135.71 | 56.13 | 161.18 | 61.00 | 99.05 | 114.40 | 91.08 | 229.12 | 144.90 | 169.67 | 124.88 | 115.67 | 94.47 | 210.13 | 252.28 |
| P25789 | Proteasome subunit alpha type-4 | **1.39** | **0.50** | 33.73 | 32.02 | 46.84 | 29.20 | 49.49 | 0.00 | 48.58 | 70.57 | 0.00 | 45.40 | 0.00 | 53.94 | 29.85 | 71.68 | 80.19 |
| Q86VP6 | Cullin-associated NEDD8-dissociated protein 1 | **1.20** | **0.50** | 61.69 | 28.06 | 73.89 | 28.67 | 30.82 | 47.36 | 97.87 | 84.12 | 48.29 | 59.37 | 127.31 | 54.63 | 55.84 | 59.92 | 86.24 |
| O75368 | SH3 domain-binding glutamic acid-rich-like protein | **0.69** | **0.50** | 32.87 | 21.58 | 22.71 | 25.43 | 35.33 | 0.00 | 45.67 | 26.58 | 56.79 | 0.00 | 0.00 | 53.71 | 45.48 | 37.06 | 0.00 |
| Q8NFI4 | Putative protein FAM10A5 | **0.82** | **0.50** | 127.97 | 48.18 | 104.66 | 59.57 | 208.05 | 115.32 | 77.53 | 115.97 | 123.00 | 182.09 | 125.73 | 113.55 | 0.00 | 114.79 | 91.81 |
| P16949 | Stathmin | **0.55** | **0.50** | 92.94 | 127.76 | 51.47 | 64.11 | 293.22 | 0.00 | 0.00 | 148.16 | 23.29 | 64.14 | 87.68 | 0.00 | 0.00 | 156.98 | 0.00 |
| P40429 | 60S ribosomal protein L13a | **1.29** | **0.50** | 91.67 | 72.44 | 118.58 | 55.06 | 94.31 | 0.00 | 192.03 | 120.96 | 51.08 | 163.37 | 123.58 | 39.80 | 72.46 | 124.70 | 187.57 |
| P63220 | 40S ribosomal protein S21 | **0.71** | **0.50** | 52.71 | 41.57 | 37.53 | 30.25 | 41.06 | 47.49 | 115.34 | 59.68 | 0.00 | 59.37 | 64.12 | 0.00 | 40.40 | 61.30 | 0.00 |
| P63010 | AP-2 complex subunit beta | **1.30** | **0.50** | 37.41 | 27.27 | 48.57 | 25.63 | 38.71 | 0.00 | 39.36 | 76.70 | 32.26 | 40.67 | 69.57 | 0.00 | 61.07 | 58.72 | 61.36 |
| Q9UBI6 | Guanine nucleotide-binding protein G(I)/G(S)/G(O) subunit gamma-12 | **0.64** | **0.50** | 36.12 | 34.57 | 23.26 | 26.72 | 59.23 | 0.00 | 75.36 | 46.02 | 0.00 | 0.00 | 0.00 | 0.00 | 55.81 | 51.74 | 32.00 |
| Q9P2E9 | Ribosome-binding protein 1 | **1.38** | **0.50** | 51.93 | 48.42 | 71.51 | 44.70 | 98.10 | 108.59 | 21.62 | 31.37 | 0.00 | 55.32 | 72.38 | 65.42 | 157.23 | 51.10 | 27.64 |
| Q99536 | Synaptic vesicle membrane protein VAT-1 homolog | **1.31** | **0.50** | 43.13 | 33.70 | 56.57 | 30.28 | 88.48 | 0.00 | 41.60 | 60.44 | 25.12 | 0.00 | 55.36 | 89.19 | 58.48 | 63.41 | 72.97 |
| O95336 | 6-phosphogluconolactonase | **0.72** | **0.50** | 58.28 | 34.04 | 41.99 | 41.91 | 71.93 | 0.00 | 57.71 | 77.13 | 84.65 | 0.00 | 76.97 | 103.86 | 43.37 | 27.75 | 0.00 |
| Q16836 | Hydroxyacyl-coenzyme A dehydrogenase, mitochondrial | **0.64** | **0.51** | 45.74 | 43.96 | 29.35 | 34.87 | 59.80 | 0.00 | 97.71 | 71.17 | 0.00 | 46.55 | 83.34 | 0.00 | 0.00 | 0.00 | 46.23 |
| P01877 | Ig alpha-2 chain C region | **1.48** | **0.51** | 516.13 | 503.83 | 762.88 | 649.55 | 86.53 | 1340.13 | 522.58 | 126.69 | 504.70 | 236.46 | 792.04 | 397.22 | 2027.27 | 465.61 | 658.68 |
| P57723 | Poly(rC)-binding protein 4 | **0.58** | **0.51** | 196.29 | 185.00 | 114.81 | 203.01 | 293.14 | 0.00 | 402.14 | 286.20 | 0.00 | 189.66 | 0.00 | 0.00 | 0.00 | 0.00 | 499.18 |
| Q96FW1 | Ubiquitin thioesterase OTUB1 | **1.31** | **0.51** | 53.27 | 32.07 | 69.66 | 44.18 | 52.78 | 0.00 | 69.32 | 84.53 | 59.72 | 71.15 | 82.82 | 55.87 | 0.00 | 71.11 | 137.03 |
| Q15149 | Plectin | **1.42** | **0.51** | 154.56 | 84.33 | 218.82 | 192.41 | 136.01 | 59.16 | 166.50 | 122.97 | 288.19 | 183.24 | 125.13 | 187.47 | 603.70 | 126.70 | 86.67 |
| P17858 | 6-phosphofructokinase, liver type | **0.80** | **0.51** | 66.64 | 35.52 | 53.56 | 27.49 | 108.96 | 21.45 | 78.11 | 85.20 | 39.49 | 39.63 | 54.64 | 35.19 | 32.34 | 53.17 | 106.42 |
| P13693 | Translationally-controlled tumor protein | **1.29** | **0.51** | 66.36 | 44.73 | 85.76 | 48.12 | 62.05 | 0.00 | 54.66 | 112.52 | 102.59 | 77.70 | 180.86 | 71.52 | 77.27 | 45.61 | 61.59 |
| Q6DN03 | Putative histone H2B type 2-C | **1.43** | **0.51** | 481.23 | 227.08 | 688.07 | 635.71 | 276.74 | 326.54 | 851.37 | 517.28 | 434.24 | 657.33 | 1184.48 | 0.00 | 27.08 | 643.58 | 1615.94 |
| Q6DRA6 | Putative histone H2B type 2-D | **1.43** | **0.51** | 481.23 | 227.08 | 688.07 | 635.71 | 276.74 | 326.54 | 851.37 | 517.28 | 434.24 | 657.33 | 1184.48 | 0.00 | 27.08 | 643.58 | 1615.94 |
| P14678 | Small nuclear ribonucleoprotein-associated proteins B and B' | **1.61** | **0.51** | 11.57 | 17.36 | 18.61 | 16.72 | 0.00 | 0.00 | 18.87 | 38.98 | 0.00 | 15.64 | 21.97 | 0.00 | 0.00 | 35.24 | 38.84 |
| P63162 | Small nuclear ribonucleoprotein-associated protein N | **1.61** | **0.51** | 11.57 | 17.36 | 18.61 | 16.72 | 0.00 | 0.00 | 18.87 | 38.98 | 0.00 | 15.64 | 21.97 | 0.00 | 0.00 | 35.24 | 38.84 |
| Q9BUP0 | EF-hand domain-containing protein D1 | **1.45** | **0.51** | 12.55 | 11.82 | 18.25 | 15.16 | 24.50 | 0.00 | 21.83 | 0.00 | 16.41 | 18.89 | 23.91 | 0.00 | 12.46 | 44.35 | 9.91 |
| Q9UKX3 | Myosin-13 | **1.52** | **0.51** | 197.70 | 249.38 | 301.26 | 251.22 | 606.17 | 0.00 | 233.91 | 148.42 | 0.00 | 0.00 | 335.91 | 741.09 | 324.54 | 274.79 | 131.25 |
| P50993 | Sodium/potassium-transporting ATPase subunit alpha-2 | **1.23** | **0.51** | 54.20 | 36.61 | 66.43 | 22.46 | 62.43 | 0.00 | 101.40 | 61.61 | 45.57 | 100.16 | 77.37 | 62.68 | 43.55 | 40.62 | 74.21 |
| P36542 | ATP synthase subunit gamma, mitochondrial | **0.75** | **0.51** | 62.00 | 42.29 | 46.23 | 34.54 | 71.98 | 0.00 | 94.44 | 103.17 | 40.44 | 51.48 | 67.63 | 0.00 | 13.76 | 50.61 | 93.88 |
| Q5JNZ5 | Putative 40S ribosomal protein S26-like 1 | **1.25** | **0.51** | 103.08 | 44.35 | 129.27 | 75.18 | 88.17 | 56.91 | 152.36 | 147.81 | 70.15 | 212.84 | 131.32 | 146.13 | 0.00 | 98.37 | 186.94 |
| P18827 | Syndecan-1 | **0.74** | **0.51** | 196.31 | 121.85 | 145.08 | 125.37 | 181.03 | 406.81 | 130.04 | 99.11 | 164.55 | 142.04 | 169.52 | 0.00 | 69.90 | 118.98 | 370.04 |
| P28070 | Proteasome subunit beta type-4 | **1.46** | **0.51** | 26.29 | 25.74 | 38.49 | 32.32 | 28.98 | 0.00 | 48.35 | 54.11 | 0.00 | 80.53 | 57.19 | 0.00 | 0.00 | 48.83 | 44.38 |
| P04406 | Glyceraldehyde-3-phosphate dehydrogenase | **0.89** | **0.51** | 2819.15 | 999.70 | 2519.45 | 386.09 | 3344.40 | 1706.25 | 2687.59 | 4229.28 | 2128.23 | 2337.96 | 2116.36 | 3132.35 | 2290.89 | 2393.78 | 2845.37 |
| Q14134 | Tripartite motif-containing protein 29 | **0.61** | **0.52** | 59.96 | 66.28 | 36.86 | 47.39 | 39.43 | 0.00 | 123.31 | 0.00 | 137.08 | 0.00 | 62.42 | 0.00 | 0.00 | 41.41 | 117.34 |
| P22307 | Non-specific lipid-transfer protein | **0.61** | **0.52** | 22.21 | 20.92 | 13.58 | 21.27 | 43.75 | 0.00 | 0.00 | 29.28 | 38.00 | 0.00 | 45.62 | 0.00 | 0.00 | 35.88 | 0.00 |
| P49207 | 60S ribosomal protein L34 | **1.47** | **0.52** | 78.46 | 84.04 | 115.13 | 94.65 | 58.98 | 0.00 | 165.61 | 167.71 | 0.00 | 154.80 | 230.44 | 135.80 | 0.00 | 0.00 | 169.73 |
| P11216 | Glycogen phosphorylase, brain form | **1.41** | **0.52** | 38.35 | 45.44 | 54.22 | 32.94 | 24.69 | 0.00 | 106.45 | 60.60 | 0.00 | 50.09 | 60.99 | 29.28 | 26.54 | 42.28 | 116.12 |
| P01876 | Ig alpha-1 chain C region | **1.51** | **0.52** | 601.72 | 595.56 | 905.91 | 850.88 | 97.72 | 1579.80 | 542.45 | 150.81 | 637.81 | 389.05 | 792.04 | 463.54 | 2615.48 | 490.38 | 684.93 |
| Q6NVY1 | 3-hydroxyisobutyryl-CoA hydrolase, mitochondrial | **1.72** | **0.52** | 54.24 | 61.89 | 93.36 | 124.73 | 147.33 | 0.00 | 79.99 | 43.90 | 0.00 | 219.54 | 10.55 | 0.00 | 281.86 | 0.00 | 48.19 |
| O00303 | Eukaryotic translation initiation factor 3 subunit F | **0.64** | **0.52** | 21.53 | 21.00 | 13.76 | 17.60 | 45.32 | 0.00 | 24.62 | 37.71 | 0.00 | 43.55 | 15.84 | 0.00 | 0.00 | 0.00 | 23.15 |
| Q13561 | Dynactin subunit 2 | **1.48** | **0.52** | 25.66 | 43.36 | 37.90 | 12.14 | 100.07 | 0.00 | 0.00 | 28.23 | 0.00 | 29.10 | 49.93 | 54.32 | 29.21 | 25.03 | 39.79 |
| P51888 | Prolargin | **0.52** | **0.52** | 255.70 | 432.41 | 132.31 | 135.00 | 253.01 | 1005.19 | 20.31 | 0.00 | 0.00 | 66.53 | 169.14 | 389.28 | 62.85 | 84.68 | 21.37 |
| P00367 | Glutamate dehydrogenase 1, mitochondrial | **0.75** | **0.52** | 67.26 | 53.62 | 50.78 | 26.30 | 44.77 | 0.00 | 104.62 | 136.47 | 50.42 | 64.47 | 24.59 | 29.98 | 27.72 | 74.86 | 83.04 |
| O00159 | Unconventional myosin-Ic | **1.32** | **0.52** | 22.62 | 21.91 | 29.77 | 8.43 | 33.81 | 0.00 | 49.43 | 29.88 | 0.00 | 26.67 | 18.88 | 42.89 | 34.31 | 31.63 | 24.21 |
| Q06830 | Peroxiredoxin-1 | **1.17** | **0.52** | 764.60 | 190.36 | 892.03 | 388.65 | 926.42 | 464.08 | 802.87 | 710.05 | 919.57 | 537.70 | 1012.09 | 837.53 | 368.81 | 1364.83 | 1231.19 |
| Q15417 | Calponin-3 | **1.45** | **0.52** | 66.38 | 78.07 | 96.19 | 70.81 | 20.68 | 109.62 | 183.40 | 18.21 | 0.00 | 95.95 | 134.74 | 39.63 | 201.27 | 105.55 | 0.00 |
| P35222 | Catenin beta-1 | **1.35** | **0.53** | 44.17 | 26.62 | 59.62 | 46.00 | 53.12 | 0.00 | 60.37 | 67.10 | 40.23 | 116.99 | 91.70 | 34.15 | 0.00 | 25.46 | 89.43 |
| P50570 | Dynamin-2 | **0.81** | **0.53** | 41.36 | 24.91 | 33.45 | 14.31 | 38.02 | 0.00 | 63.67 | 50.25 | 54.88 | 34.10 | 45.60 | 22.80 | 15.51 | 53.93 | 28.80 |
| P12956 | X-ray repair cross-complementing protein 6 | **1.22** | **0.53** | 122.16 | 69.02 | 148.51 | 63.45 | 163.27 | 10.77 | 179.37 | 156.97 | 100.41 | 207.19 | 135.53 | 83.12 | 69.10 | 175.44 | 220.69 |
| Q00610 | Clathrin heavy chain 1 | **1.19** | **0.53** | 133.87 | 66.49 | 159.26 | 61.22 | 130.90 | 52.86 | 180.06 | 217.05 | 88.47 | 97.59 | 238.62 | 90.35 | 132.58 | 206.78 | 189.65 |
| Q15819 | Ubiquitin-conjugating enzyme E2 variant 2 | **1.16** | **0.53** | 63.27 | 21.02 | 73.11 | 27.27 | 85.73 | 43.13 | 58.74 | 84.81 | 43.95 | 78.87 | 54.37 | 46.75 | 50.41 | 94.15 | 114.11 |
| O15173 | Membrane-associated progesterone receptor component 2 | **0.80** | **0.53** | 78.70 | 40.40 | 62.64 | 40.30 | 135.17 | 102.77 | 64.50 | 58.95 | 32.13 | 62.09 | 142.20 | 55.73 | 41.03 | 38.66 | 36.15 |
| O43809 | Cleavage and polyadenylation specificity factor subunit 5 | **1.43** | **0.53** | 23.91 | 25.15 | 34.29 | 26.92 | 19.63 | 0.00 | 52.35 | 47.55 | 0.00 | 51.72 | 46.23 | 0.00 | 0.00 | 48.66 | 59.15 |
| P07910 | Heterogeneous nuclear ribonucleoproteins C1/C2 | **1.24** | **0.53** | 204.18 | 128.15 | 254.16 | 123.85 | 176.59 | 61.06 | 311.97 | 359.83 | 111.43 | 212.44 | 277.17 | 134.66 | 120.90 | 337.25 | 442.52 |
| P62942 | Peptidyl-prolyl cis-trans isomerase FKBP1A | **1.37** | **0.53** | 44.29 | 42.56 | 60.53 | 39.57 | 82.26 | 0.00 | 86.89 | 0.00 | 52.31 | 91.00 | 77.47 | 108.62 | 39.43 | 0.00 | 46.64 |
| O95716 | Ras-related protein Rab-3D | **0.93** | **0.53** | 374.77 | 87.73 | 349.93 | 29.88 | 344.40 | 275.99 | 396.56 | 511.72 | 345.18 | 341.24 | 312.34 | 403.64 | 347.52 | 341.37 | 353.48 |
| Q15836 | Vesicle-associated membrane protein 3 | **0.79** | **0.53** | 78.13 | 39.84 | 61.82 | 42.49 | 84.28 | 144.96 | 60.31 | 52.01 | 49.07 | 0.00 | 129.42 | 78.39 | 43.71 | 63.25 | 56.11 |
| P30048 | Thioredoxin-dependent peroxide reductase, mitochondrial | **0.79** | **0.53** | 137.94 | 99.82 | 109.36 | 39.10 | 161.57 | 0.00 | 273.34 | 156.67 | 98.12 | 104.87 | 122.09 | 179.49 | 69.91 | 100.60 | 79.20 |
| P35221 | Catenin alpha-1 | **0.78** | **0.53** | 96.94 | 58.88 | 75.17 | 52.35 | 150.48 | 0.00 | 136.55 | 95.15 | 102.50 | 141.78 | 109.90 | 28.35 | 0.00 | 87.21 | 83.76 |
| P11217 | Glycogen phosphorylase, muscle form | **1.51** | **0.53** | 30.19 | 41.42 | 45.74 | 38.10 | 0.00 | 0.00 | 78.91 | 72.06 | 0.00 | 50.09 | 79.88 | 0.00 | 0.00 | 56.62 | 87.84 |
| Q15366 | Poly(rC)-binding protein 2 | **1.15** | **0.53** | 226.84 | 103.75 | 260.47 | 67.99 | 258.83 | 95.75 | 332.24 | 306.51 | 140.88 | 277.50 | 270.57 | 203.03 | 167.89 | 281.32 | 362.53 |
| P53675 | Clathrin heavy chain 2 | **1.34** | **0.54** | 59.46 | 58.97 | 79.86 | 46.23 | 65.33 | 0.00 | 101.62 | 130.38 | 0.00 | 19.76 | 153.03 | 44.20 | 75.33 | 98.85 | 87.99 |
| P26038 | Moesin | **1.15** | **0.54** | 302.24 | 131.54 | 347.65 | 103.22 | 396.00 | 162.59 | 183.45 | 465.98 | 303.19 | 199.33 | 291.80 | 468.18 | 292.43 | 395.45 | 438.72 |
| P08123 | Collagen alpha-2(I) chain | **0.43** | **0.54** | 5379.55 | 10142.47 | 2299.64 | 1158.73 | 2474.10 | 23440.15 | 758.33 | 29.52 | 195.64 | 3194.86 | 2260.38 | 2662.11 | 3086.70 | 2551.57 | 42.20 |
| Q14568 | Putative heat shock protein HSP 90-alpha A2 | **1.23** | **0.54** | 732.11 | 275.47 | 897.31 | 513.96 | 904.34 | 347.43 | 773.07 | 1051.86 | 583.84 | 785.05 | 614.11 | 635.08 | 331.06 | 1297.61 | 1720.92 |
| P02765 | Alpha-2-HS-glycoprotein | **1.26** | **0.54** | 105.62 | 42.61 | 133.41 | 88.30 | 58.03 | 157.08 | 67.99 | 136.00 | 109.01 | 104.25 | 79.46 | 300.14 | 107.17 | 155.20 | 54.27 |
| P62917 | 60S ribosomal protein L8 | **1.24** | **0.54** | 136.06 | 104.69 | 168.25 | 61.07 | 92.77 | 0.00 | 190.16 | 278.33 | 119.06 | 188.90 | 78.07 | 159.43 | 144.09 | 173.85 | 265.18 |
| P02461 | Collagen alpha-1(III) chain | **0.39** | **0.54** | 2315.87 | 4652.33 | 913.20 | 602.27 | 684.48 | 10624.28 | 218.64 | 20.39 | 31.57 | 1836.71 | 877.67 | 890.69 | 1223.23 | 618.68 | 32.19 |
| Q92901 | 60S ribosomal protein L3-like | **0.62** | **0.54** | 52.46 | 55.23 | 32.27 | 50.41 | 46.69 | 0.00 | 124.24 | 91.36 | 0.00 | 0.00 | 0.00 | 0.00 | 0.00 | 86.58 | 107.03 |
| P84090 | Enhancer of rudimentary homolog | **0.63** | **0.54** | 58.04 | 63.24 | 36.56 | 50.03 | 144.33 | 0.00 | 0.00 | 46.80 | 99.09 | 126.18 | 0.00 | 0.00 | 0.00 | 34.95 | 58.25 |
| P04075 | Fructose-bisphosphate aldolase A | **1.16** | **0.54** | 617.75 | 324.09 | 715.80 | 186.33 | 574.14 | 250.47 | 453.23 | 1118.45 | 692.46 | 1000.76 | 592.27 | 643.27 | 506.63 | 673.75 | 878.11 |
| Q9C0K3 | Actin-related protein 3C | **1.45** | **0.55** | 15.59 | 25.03 | 22.66 | 10.86 | 20.46 | 0.00 | 0.00 | 57.47 | 0.00 | 10.92 | 32.48 | 9.02 | 21.71 | 34.91 | 26.90 |
| P01764 | Ig heavy chain V-III region VH26 | **0.67** | **0.55** | 128.64 | 132.58 | 86.54 | 88.86 | 0.00 | 286.09 | 117.56 | 0.00 | 239.56 | 56.15 | 49.58 | 0.00 | 258.11 | 70.27 | 85.14 |
| P06703 | Protein S100-A6 | **0.76** | **0.55** | 1128.44 | 640.37 | 858.46 | 760.06 | 1568.94 | 441.99 | 831.25 | 792.03 | 2007.97 | 2204.98 | 770.30 | 1125.41 | 0.00 | 389.10 | 660.96 |
| P02452 | Collagen alpha-1(I) chain | **0.42** | **0.55** | 9245.54 | 18026.75 | 3905.83 | 1974.77 | 3160.16 | 41420.04 | 1242.84 | 98.44 | 306.23 | 5637.99 | 3971.01 | 3947.73 | 5031.18 | 4750.69 | 96.36 |
| P50995 | Annexin A11 | **0.82** | **0.55** | 69.99 | 45.06 | 57.58 | 17.26 | 106.68 | 0.00 | 111.82 | 58.96 | 72.47 | 34.37 | 69.62 | 75.32 | 38.04 | 67.61 | 60.50 |
| Q96I99 | Succinyl-CoA ligase [GDP-forming] subunit beta, mitochondrial | **2.16** | **0.55** | 30.64 | 24.64 | 66.13 | 123.58 | 28.35 | 0.00 | 68.09 | 34.36 | 22.39 | 34.88 | 316.77 | 0.00 | 0.00 | 19.34 | 25.77 |
| P22695 | Cytochrome b-c1 complex subunit 2, mitochondrial | **0.77** | **0.55** | 62.66 | 45.17 | 47.97 | 32.81 | 79.65 | 0.00 | 119.18 | 75.87 | 38.61 | 51.56 | 101.03 | 34.33 | 0.00 | 55.73 | 45.15 |
| P13804 | Electron transfer flavoprotein subunit alpha, mitochondrial | **0.70** | **0.55** | 40.25 | 37.85 | 28.11 | 26.64 | 55.50 | 0.00 | 80.91 | 64.85 | 0.00 | 27.97 | 54.32 | 0.00 | 22.85 | 0.00 | 63.53 |
| P62191 | 26S protease regulatory subunit 4 | **0.76** | **0.55** | 22.83 | 14.30 | 17.38 | 14.60 | 18.87 | 0.00 | 26.76 | 35.77 | 32.75 | 6.17 | 39.52 | 0.00 | 12.00 | 18.36 | 28.24 |
| O00148 | ATP-dependent RNA helicase DDX39A | **1.30** | **0.55** | 84.82 | 41.62 | 110.09 | 81.76 | 57.03 | 45.45 | 115.33 | 141.90 | 64.40 | 104.02 | 73.92 | 0.00 | 70.63 | 205.85 | 206.15 |
| P20774 | Mimecan | **0.45** | **0.55** | 178.41 | 378.27 | 79.51 | 104.84 | 37.59 | 854.46 | 0.00 | 0.00 | 0.00 | 6.81 | 133.56 | 269.65 | 38.22 | 28.81 | 0.00 |
| Q9H254 | Spectrin beta chain, non-erythrocytic 4 | **1.29** | **0.55** | 78.78 | 54.95 | 101.54 | 64.95 | 107.55 | 0.00 | 100.31 | 138.71 | 47.33 | 0.00 | 142.92 | 103.77 | 52.40 | 133.62 | 176.52 |
| P42704 | Leucine-rich PPR motif-containing protein, mitochondrial | **1.26** | **0.55** | 42.59 | 31.20 | 53.83 | 29.00 | 48.54 | 0.00 | 52.66 | 84.07 | 27.66 | 61.26 | 93.04 | 28.44 | 21.58 | 38.49 | 80.14 |
| Q95604 | HLA class I histocompatibility antigen, Cw-17 alpha chain | **1.30** | **0.55** | 126.95 | 90.19 | 164.75 | 108.75 | 264.50 | 150.53 | 122.04 | 69.28 | 28.40 | 49.31 | 152.83 | 281.19 | 144.62 | 305.20 | 55.33 |
| P05386 | 60S acidic ribosomal protein P1 | **0.78** | **0.55** | 286.33 | 172.80 | 222.41 | 169.57 | 378.55 | 27.21 | 396.87 | 436.96 | 192.08 | 106.98 | 0.00 | 119.09 | 351.23 | 328.94 | 428.24 |
| P52943 | Cysteine-rich protein 2 | **0.69** | **0.55** | 74.70 | 51.12 | 51.91 | 68.02 | 135.16 | 0.00 | 100.72 | 84.18 | 53.46 | 55.05 | 0.00 | 0.00 | 0.00 | 86.97 | 169.44 |
| P62826 | GTP-binding nuclear protein Ran | **1.27** | **0.55** | 242.59 | 108.13 | 307.03 | 210.20 | 193.78 | 121.91 | 305.89 | 396.13 | 195.25 | 377.44 | 199.84 | 143.42 | 133.07 | 297.50 | 690.89 |
| P46782 | 40S ribosomal protein S5 | **0.84** | **0.55** | 166.88 | 73.73 | 139.39 | 73.67 | 209.12 | 75.91 | 208.24 | 241.08 | 100.04 | 108.88 | 147.31 | 61.55 | 79.34 | 177.26 | 262.03 |
| Q9UQ16 | Dynamin-3 | **0.70** | **0.55** | 29.64 | 27.85 | 20.61 | 20.91 | 38.74 | 0.00 | 55.98 | 0.00 | 53.47 | 0.00 | 55.57 | 22.80 | 15.51 | 0.00 | 29.77 |
| P48735 | Isocitrate dehydrogenase [NADP], mitochondrial | **1.27** | **0.55** | 149.92 | 116.67 | 189.73 | 98.75 | 321.57 | 0.00 | 108.57 | 142.28 | 177.19 | 237.71 | 218.81 | 86.17 | 63.62 | 205.52 | 326.59 |
| Q9UKM9 | RNA-binding protein Raly | **1.24** | **0.55** | 51.17 | 30.97 | 63.45 | 34.56 | 55.92 | 0.00 | 68.04 | 81.36 | 50.55 | 80.00 | 84.29 | 47.92 | 0.00 | 92.41 | 76.07 |
| Q09028 | Histone-binding protein RBBP4 | **0.63** | **0.55** | 73.95 | 103.36 | 46.24 | 37.63 | 33.88 | 0.00 | 54.60 | 255.50 | 25.75 | 36.04 | 39.59 | 0.00 | 20.22 | 82.12 | 99.44 |
| P22626 | Heterogeneous nuclear ribonucleoproteins A2/B1 | **0.82** | **0.55** | 439.48 | 170.20 | 358.24 | 249.94 | 376.08 | 378.40 | 721.98 | 449.45 | 271.51 | 441.35 | 159.56 | 142.77 | 154.35 | 757.80 | 493.61 |
| P00846 | ATP synthase subunit a | **1.29** | **0.56** | 46.57 | 45.61 | 60.20 | 17.38 | 52.66 | 0.00 | 81.16 | 99.01 | 0.00 | 70.53 | 70.66 | 61.36 | 30.86 | 49.64 | 78.13 |
| Q9Y266 | Nuclear migration protein nudC | **1.28** | **0.56** | 32.85 | 23.59 | 42.16 | 26.32 | 54.73 | 0.00 | 47.35 | 46.13 | 16.06 | 39.43 | 81.06 | 36.67 | 0.00 | 53.83 | 41.97 |
| Q9H4B7 | Tubulin beta-1 chain | **1.28** | **0.56** | 316.17 | 181.83 | 404.55 | 275.85 | 30.55 | 334.75 | 379.18 | 531.20 | 305.16 | 286.24 | 291.91 | 115.56 | 380.85 | 923.29 | 429.47 |
| P05556 | Integrin beta-1 | **0.83** | **0.56** | 108.13 | 61.41 | 89.68 | 38.54 | 172.70 | 50.80 | 89.53 | 173.89 | 53.75 | 69.41 | 96.85 | 148.17 | 97.41 | 95.30 | 30.93 |
| P55769 | NHP2-like protein 1 | **0.80** | **0.56** | 79.13 | 22.10 | 63.65 | 52.62 | 65.18 | 93.78 | 108.34 | 75.23 | 53.13 | 130.84 | 36.02 | 27.35 | 0.00 | 67.66 | 120.05 |
| Q9H115 | Beta-soluble NSF attachment protein | **1.64** | **0.56** | 7.32 | 10.02 | 12.00 | 14.57 | 18.48 | 0.00 | 0.00 | 18.12 | 0.00 | 4.32 | 24.86 | 0.00 | 7.78 | 0.00 | 35.05 |
| P07476 | Involucrin | **0.77** | **0.56** | 257.38 | 133.00 | 198.96 | 177.99 | 179.39 | 101.64 | 420.95 | 367.30 | 217.62 | 434.56 | 159.47 | 0.00 | 0.00 | 269.83 | 329.92 |
| P33176 | Kinesin-1 heavy chain | **1.39** | **0.56** | 19.13 | 18.95 | 26.68 | 21.92 | 21.90 | 0.00 | 31.08 | 42.66 | 0.00 | 37.22 | 39.73 | 0.00 | 0.00 | 30.30 | 52.84 |
| Q16659 | Mitogen-activated protein kinase 6 | **0.51** | **0.56** | 43.39 | 63.14 | 22.07 | 54.07 | 152.05 | 0.00 | 24.21 | 40.67 | 0.00 | 0.00 | 0.00 | 0.00 | 132.44 | 0.00 | 0.00 |
| P23526 | Adenosylhomocysteinase | **1.24** | **0.56** | 96.32 | 46.40 | 119.35 | 73.70 | 75.88 | 42.97 | 157.41 | 130.39 | 74.96 | 147.03 | 70.28 | 56.81 | 43.86 | 170.22 | 227.92 |
| O43423 | Acidic leucine-rich nuclear phosphoprotein 32 family member C | **0.73** | **0.56** | 170.06 | 117.24 | 123.40 | 135.33 | 332.18 | 225.90 | 147.76 | 126.73 | 17.73 | 359.38 | 67.31 | 0.00 | 0.00 | 152.22 | 161.50 |
| Q6PIW4 | Fidgetin-like protein 1 | **1.77** | **0.56** | 47.81 | 59.39 | 84.40 | 124.29 | 50.80 | 0.00 | 145.31 | 42.94 | 0.00 | 32.60 | 294.66 | 0.00 | 0.00 | 0.00 | 179.15 |
| Q9UN37 | Vacuolar protein sorting-associated protein 4A | **1.77** | **0.56** | 47.81 | 59.39 | 84.40 | 124.29 | 50.80 | 0.00 | 145.31 | 42.94 | 0.00 | 32.60 | 294.66 | 0.00 | 0.00 | 0.00 | 179.15 |
| P28066 | Proteasome subunit alpha type-5 | **1.32** | **0.56** | 56.39 | 34.91 | 74.52 | 59.09 | 70.75 | 0.00 | 91.78 | 70.06 | 49.39 | 40.93 | 179.09 | 28.05 | 26.90 | 67.32 | 104.82 |
| P22314 | Ubiquitin-like modifier-activating enzyme 1 | **1.44** | **0.56** | 140.64 | 53.83 | 202.70 | 223.78 | 163.25 | 82.53 | 189.23 | 185.36 | 82.83 | 79.37 | 128.38 | 113.02 | 111.87 | 658.11 | 125.43 |
| Q92597 | Protein NDRG1 | **0.72** | **0.56** | 108.66 | 81.93 | 78.03 | 86.27 | 105.38 | 0.00 | 71.23 | 218.53 | 148.19 | 156.87 | 137.28 | 0.00 | 0.00 | 0.00 | 174.04 |
| P20742 | Pregnancy zone protein | **0.74** | **0.57** | 136.98 | 104.19 | 101.06 | 95.10 | 90.66 | 272.45 | 199.75 | 0.00 | 122.04 | 103.82 | 52.99 | 285.61 | 77.83 | 71.13 | 14.99 |
| Q8TEX9 | Importin-4 | **0.37** | **0.57** | 378.87 | 847.18 | 140.64 | 123.57 | 0.00 | 1894.35 | 0.00 | 0.00 | 0.00 | 231.29 | 99.59 | 0.00 | 261.26 | 251.71 | 0.00 |
| Q9P035 | Very-long-chain (3R)-3-hydroxyacyl-[acyl-carrier protein] dehydratase 3 | **1.46** | **0.57** | 33.89 | 31.50 | 49.53 | 50.94 | 51.02 | 0.00 | 66.13 | 52.31 | 0.00 | 133.51 | 73.38 | 0.00 | 0.00 | 30.87 | 59.41 |
| Q9NTK5 | Obg-like ATPase 1 | **1.18** | **0.57** | 34.62 | 21.85 | 40.92 | 12.94 | 37.80 | 0.00 | 35.94 | 60.77 | 38.58 | 34.52 | 50.25 | 42.57 | 17.80 | 50.36 | 50.05 |
| P62854 | 40S ribosomal protein S26 | **1.22** | **0.57** | 86.92 | 42.50 | 106.05 | 60.11 | 68.06 | 56.91 | 134.19 | 130.92 | 44.53 | 120.74 | 131.32 | 146.13 | 0.00 | 74.24 | 163.88 |
| P30043 | Flavin reductase (NADPH) | **0.78** | **0.57** | 145.63 | 112.70 | 112.97 | 40.41 | 88.16 | 68.02 | 212.67 | 47.97 | 311.31 | 88.90 | 95.05 | 153.03 | 130.35 | 156.44 | 54.07 |
| Q99798 | Aconitate hydratase, mitochondrial | **0.80** | **0.57** | 60.72 | 45.24 | 48.57 | 20.48 | 60.54 | 0.00 | 118.79 | 85.83 | 38.45 | 42.38 | 28.72 | 67.52 | 55.10 | 73.97 | 23.73 |
| A6NL28 | Putative tropomyosin alpha-3 chain-like protein | **1.24** | **0.57** | 175.66 | 118.33 | 218.28 | 118.31 | 367.09 | 141.87 | 72.77 | 203.67 | 92.92 | 124.03 | 246.79 | 415.79 | 275.00 | 136.20 | 111.85 |
| P48643 | T-complex protein 1 subunit epsilon | **1.29** | **0.57** | 61.33 | 45.14 | 79.27 | 53.51 | 50.81 | 0.00 | 57.07 | 125.48 | 73.30 | 111.95 | 55.20 | 32.78 | 40.22 | 62.86 | 172.62 |
| P33121 | Long-chain-fatty-acid--CoA ligase 1 | **0.60** | **0.57** | 34.76 | 49.00 | 21.00 | 27.42 | 53.44 | 0.00 | 112.84 | 7.53 | 0.00 | 0.00 | 66.60 | 39.81 | 0.00 | 0.00 | 19.57 |
| P62158 | Calmodulin | **1.18** | **0.57** | 440.41 | 253.21 | 518.77 | 188.82 | 826.55 | 549.21 | 287.73 | 351.91 | 186.66 | 752.72 | 515.15 | 301.20 | 371.37 | 435.47 | 736.74 |
| P09525 | Annexin A4 | **0.73** | **0.57** | 187.11 | 194.30 | 136.89 | 74.63 | 529.77 | 97.81 | 95.80 | 151.68 | 60.48 | 98.78 | 286.66 | 107.99 | 85.70 | 118.61 | 123.57 |
| P49448 | Glutamate dehydrogenase 2, mitochondrial | **0.80** | **0.57** | 58.81 | 43.19 | 47.11 | 21.80 | 44.77 | 0.00 | 84.69 | 114.18 | 50.42 | 64.47 | 24.59 | 29.98 | 27.72 | 64.19 | 71.75 |
| P01009 | Alpha-1-antitrypsin | **0.74** | **0.57** | 1222.87 | 1225.32 | 904.43 | 501.44 | 910.18 | 3401.39 | 630.69 | 590.93 | 581.14 | 750.12 | 335.65 | 1473.78 | 1566.58 | 745.14 | 555.31 |
| P11277 | Spectrin beta chain, erythrocytic | **0.54** | **0.58** | 11.22 | 16.82 | 6.05 | 12.68 | 4.07 | 0.00 | 12.01 | 0.00 | 39.99 | 4.63 | 0.00 | 0.00 | 0.00 | 31.66 | 0.00 |
| Q9BR76 | Coronin-1B | **0.78** | **0.58** | 50.91 | 22.65 | 39.60 | 37.95 | 58.17 | 63.82 | 36.46 | 76.39 | 19.69 | 29.11 | 44.07 | 0.00 | 0.00 | 69.99 | 94.46 |
| P09496 | Clathrin light chain A | **1.41** | **0.58** | 26.50 | 38.25 | 37.37 | 23.81 | 83.35 | 0.00 | 0.00 | 49.16 | 0.00 | 66.98 | 29.45 | 0.00 | 28.75 | 41.45 | 57.59 |
| P21291 | Cysteine and glycine-rich protein 1 | **1.27** | **0.58** | 76.01 | 49.78 | 96.65 | 65.60 | 144.59 | 94.77 | 84.94 | 19.31 | 36.46 | 40.24 | 35.52 | 173.90 | 170.31 | 116.35 | 43.61 |
| P41219 | Peripherin | **0.77** | **0.58** | 669.20 | 521.31 | 517.61 | 357.33 | 479.98 | 1481.12 | 512.63 | 76.37 | 795.88 | 326.55 | 50.42 | 589.63 | 986.94 | 851.17 | 300.98 |
| O43488 | Aflatoxin B1 aldehyde reductase member 2 | **1.50** | **0.58** | 20.88 | 28.87 | 31.41 | 31.94 | 0.00 | 0.00 | 57.87 | 46.55 | 0.00 | 0.00 | 52.70 | 0.00 | 23.80 | 29.43 | 82.52 |
| P10635 | Cytochrome P450 2D6 | **0.64** | **0.58** | 26.41 | 25.90 | 16.82 | 29.44 | 58.41 | 0.00 | 31.96 | 41.70 | 0.00 | 0.00 | 72.11 | 28.83 | 0.00 | 0.00 | 0.00 |
| P14649 | Myosin light chain 6B | **1.22** | **0.58** | 721.88 | 615.89 | 882.41 | 297.63 | 1771.01 | 638.34 | 604.59 | 432.03 | 163.44 | 740.11 | 1038.30 | 1213.53 | 1146.53 | 707.73 | 448.29 |
| Q9BV40 | Vesicle-associated membrane protein 8 | **0.76** | **0.59** | 60.43 | 43.69 | 46.07 | 40.90 | 123.33 | 0.00 | 63.07 | 55.90 | 59.84 | 59.53 | 99.97 | 0.00 | 0.00 | 39.41 | 77.52 |
| P12532 | Creatine kinase U-type, mitochondrial | **2.03** | **0.59** | 22.37 | 14.28 | 45.52 | 90.93 | 18.82 | 0.00 | 30.67 | 24.94 | 37.44 | 45.81 | 0.00 | 0.00 | 0.00 | 0.00 | 227.33 |
| P68371 | Tubulin beta-4B chain | **1.16** | **0.59** | 772.46 | 391.56 | 894.41 | 334.46 | 766.43 | 305.82 | 915.56 | 1337.67 | 536.82 | 683.62 | 919.34 | 524.79 | 679.98 | 1412.15 | 1146.60 |
| P35527 | Keratin, type I cytoskeletal 9 | **0.51** | **0.59** | 536.82 | 1024.91 | 271.66 | 521.58 | 48.22 | 112.10 | 0.00 | 156.66 | 2367.10 | 1330.89 | 56.50 | 41.08 | 43.38 | 0.00 | 158.12 |
| Q16822 | Phosphoenolpyruvate carboxykinase [GTP], mitochondrial | **0.65** | **0.59** | 17.64 | 16.18 | 11.54 | 19.54 | 28.53 | 0.00 | 31.90 | 27.78 | 0.00 | 0.00 | 0.00 | 0.00 | 0.00 | 22.16 | 47.08 |
| P69892 | Hemoglobin subunit gamma-2 | **0.80** | **0.59** | 11282.10 | 4854.36 | 8975.58 | 8122.32 | 5446.73 | 9397.75 | 13382.26 | 9822.42 | 18361.33 | 2704.06 | 2067.49 | 4208.00 | 23297.30 | 12836.93 | 8739.71 |
| Q8NC51 | Plasminogen activator inhibitor 1 RNA-binding protein | **0.65** | **0.59** | 15.86 | 20.30 | 10.25 | 13.20 | 5.69 | 0.00 | 45.87 | 27.74 | 0.00 | 16.81 | 0.00 | 0.00 | 0.00 | 32.83 | 11.84 |
| Q9H9B4 | Sideroflexin-1 | **0.66** | **0.60** | 29.48 | 27.79 | 19.61 | 31.11 | 51.63 | 0.00 | 38.36 | 57.43 | 0.00 | 48.21 | 0.00 | 0.00 | 0.00 | 0.00 | 69.43 |
| P42356 | Phosphatidylinositol 4-kinase alpha | **1.27** | **0.60** | 158.10 | 154.16 | 200.68 | 102.84 | 351.38 | 0.00 | 0.00 | 210.91 | 228.23 | 0.00 | 261.20 | 232.81 | 190.76 | 238.93 | 280.41 |
| P02462 | Collagen alpha-1(IV) chain | **1.38** | **0.60** | 193.22 | 189.39 | 266.78 | 244.66 | 167.77 | 163.72 | 516.78 | 34.54 | 83.29 | 184.74 | 202.81 | 589.23 | 78.23 | 545.66 | 0.00 |
| P09012 | U1 small nuclear ribonucleoprotein A | **1.46** | **0.60** | 18.37 | 25.17 | 26.90 | 26.34 | 0.00 | 0.00 | 0.00 | 44.60 | 47.24 | 25.69 | 70.97 | 29.37 | 0.00 | 0.00 | 35.39 |
| P20674 | Cytochrome c oxidase subunit 5A, mitochondrial | **0.71** | **0.60** | 70.15 | 85.78 | 49.79 | 30.36 | 208.05 | 0.00 | 88.27 | 54.44 | 0.00 | 66.82 | 0.00 | 91.55 | 42.95 | 53.52 | 43.89 |
| Q9UHV9 | Prefoldin subunit 2 | **0.71** | **0.60** | 24.41 | 23.50 | 17.24 | 20.23 | 52.21 | 38.29 | 0.00 | 31.56 | 0.00 | 21.41 | 39.17 | 0.00 | 0.00 | 42.87 | 0.00 |
| Q58FF6 | Putative heat shock protein HSP 90-beta 4 | **0.82** | **0.60** | 2115.41 | 1602.74 | 1728.16 | 650.89 | 1722.63 | 4870.38 | 1969.70 | 1170.51 | 843.82 | 1395.18 | 1027.18 | 2104.55 | 2660.27 | 2074.56 | 1107.22 |
| Q9BSJ8 | Extended synaptotagmin-1 | **1.67** | **0.60** | 10.05 | 22.47 | 16.83 | 19.31 | 0.00 | 0.00 | 0.00 | 50.24 | 0.00 | 0.00 | 8.84 | 51.43 | 22.82 | 17.89 | 0.00 |
| O75531 | Barrier-to-autointegration factor | **1.46** | **0.60** | 38.90 | 61.86 | 56.64 | 47.65 | 141.72 | 0.00 | 0.00 | 52.79 | 0.00 | 85.60 | 0.00 | 0.00 | 62.47 | 118.08 | 73.69 |
| Q04837 | Single-stranded DNA-binding protein, mitochondrial | **1.38** | **0.60** | 32.25 | 31.59 | 44.48 | 41.51 | 36.82 | 0.00 | 69.10 | 55.33 | 0.00 | 95.39 | 45.58 | 0.00 | 0.00 | 36.63 | 89.25 |
| Q9Y3C8 | Ubiquitin-fold modifier-conjugating enzyme 1 | **0.67** | **0.60** | 32.39 | 18.94 | 21.70 | 40.52 | 42.86 | 0.00 | 30.90 | 43.83 | 44.38 | 0.00 | 0.00 | 0.00 | 0.00 | 100.86 | 29.34 |
| P19338 | Nucleolin | **1.26** | **0.60** | 146.20 | 66.81 | 183.74 | 142.84 | 108.40 | 85.65 | 207.58 | 229.13 | 100.25 | 284.81 | 85.60 | 57.45 | 55.10 | 215.11 | 404.38 |
| Q1KMD3 | Heterogeneous nuclear ribonucleoprotein U-like protein 2 | **1.14** | **0.61** | 61.54 | 26.67 | 70.42 | 27.92 | 46.16 | 45.00 | 93.74 | 86.87 | 35.94 | 67.94 | 97.34 | 30.14 | 45.43 | 97.40 | 84.25 |
| P37108 | Signal recognition particle 14 kDa protein | **0.76** | **0.61** | 34.08 | 21.56 | 25.75 | 28.61 | 40.33 | 0.00 | 52.98 | 49.97 | 27.13 | 57.85 | 0.00 | 0.00 | 0.00 | 43.23 | 53.44 |
| P49748 | Very long-chain specific acyl-CoA dehydrogenase, mitochondrial | **1.28** | **0.61** | 43.36 | 45.79 | 55.71 | 31.25 | 34.85 | 0.00 | 87.93 | 94.03 | 0.00 | 31.92 | 67.13 | 68.66 | 34.46 | 25.11 | 107.00 |
| Q13310 | Polyadenylate-binding protein 4 | **1.31** | **0.61** | 49.04 | 33.58 | 64.08 | 55.05 | 48.55 | 0.00 | 70.28 | 88.24 | 38.15 | 47.90 | 56.03 | 0.00 | 30.47 | 92.17 | 157.90 |
| Q9UNL2 | Translocon-associated protein subunit gamma | **1.44** | **0.61** | 16.53 | 17.66 | 23.75 | 25.82 | 13.96 | 0.00 | 28.93 | 39.78 | 0.00 | 0.00 | 16.12 | 0.00 | 15.13 | 60.03 | 51.24 |
| Q14152 | Eukaryotic translation initiation factor 3 subunit A | **0.68** | **0.61** | 18.27 | 21.65 | 12.34 | 15.66 | 12.24 | 0.00 | 28.05 | 51.05 | 0.00 | 0.00 | 21.42 | 0.00 | 0.00 | 14.11 | 38.48 |
| Q9BVC6 | Transmembrane protein 109 | **0.64** | **0.61** | 64.60 | 78.63 | 41.27 | 68.07 | 133.24 | 165.01 | 0.00 | 0.00 | 24.73 | 160.75 | 0.00 | 86.85 | 0.00 | 0.00 | 0.00 |
| Q14247 | Src substrate cortactin | **0.79** | **0.61** | 55.07 | 35.83 | 43.57 | 36.04 | 99.08 | 0.00 | 62.07 | 49.76 | 64.47 | 63.06 | 60.46 | 0.00 | 0.00 | 49.65 | 88.23 |
| P05023 | Sodium/potassium-transporting ATPase subunit alpha-1 | **1.20** | **0.61** | 63.24 | 41.80 | 75.68 | 36.79 | 74.07 | 0.00 | 112.84 | 78.92 | 50.35 | 134.99 | 91.81 | 62.68 | 43.55 | 34.74 | 86.29 |
| P46940 | Ras GTPase-activating-like protein IQGAP1 | **0.71** | **0.61** | 287.21 | 383.37 | 204.10 | 72.93 | 116.15 | 67.89 | 216.76 | 964.49 | 70.74 | 249.19 | 206.78 | 131.27 | 107.85 | 301.00 | 228.53 |
| Q58FG1 | Putative heat shock protein HSP 90-alpha A4 | **0.87** | **0.61** | 286.81 | 73.10 | 248.30 | 149.42 | 291.31 | 187.86 | 393.23 | 271.82 | 289.86 | 203.64 | 158.95 | 131.29 | 145.58 | 511.33 | 338.98 |
| P36871 | Phosphoglucomutase-1 | **1.25** | **0.62** | 33.36 | 28.26 | 41.67 | 24.78 | 60.97 | 0.00 | 7.82 | 58.42 | 39.58 | 0.00 | 62.91 | 70.30 | 44.22 | 36.73 | 35.87 |
| P16152 | Carbonyl reductase [NADPH] 1 | **1.17** | **0.62** | 50.83 | 34.22 | 59.29 | 18.91 | 85.23 | 0.00 | 71.34 | 64.38 | 33.18 | 35.58 | 84.99 | 41.61 | 55.44 | 64.22 | 73.89 |
| Q15233 | Non-POU domain-containing octamer-binding protein | **1.19** | **0.62** | 94.82 | 39.10 | 112.74 | 67.66 | 84.89 | 51.69 | 109.60 | 154.00 | 73.91 | 154.68 | 78.47 | 0.00 | 100.28 | 181.14 | 161.84 |
| Q13435 | Splicing factor 3B subunit 2 | **0.80** | **0.62** | 32.41 | 20.25 | 26.02 | 20.39 | 43.54 | 53.11 | 36.99 | 28.40 | 0.00 | 36.13 | 44.96 | 0.00 | 0.00 | 37.72 | 37.32 |
| P02787 | Serotransferrin | **0.80** | **0.62** | 800.81 | 688.90 | 641.09 | 289.19 | 591.69 | 2012.58 | 651.13 | 376.89 | 371.76 | 496.53 | 298.85 | 809.92 | 907.78 | 966.06 | 367.42 |
| P17987 | T-complex protein 1 subunit alpha | **0.83** | **0.62** | 67.58 | 43.92 | 56.11 | 29.30 | 76.30 | 0.00 | 99.05 | 110.50 | 52.04 | 91.22 | 55.95 | 10.87 | 43.33 | 50.55 | 84.74 |
| O43396 | Thioredoxin-like protein 1 | **1.34** | **0.62** | 22.11 | 21.70 | 29.54 | 25.26 | 24.02 | 0.00 | 41.54 | 45.01 | 0.00 | 0.00 | 49.74 | 33.07 | 0.00 | 33.10 | 61.34 |
| P55010 | Eukaryotic translation initiation factor 5 | **1.32** | **0.62** | 15.37 | 14.10 | 20.24 | 16.67 | 26.68 | 0.00 | 23.39 | 26.76 | 0.00 | 22.09 | 34.21 | 0.00 | 0.00 | 26.79 | 38.37 |
| P50990 | T-complex protein 1 subunit theta | **1.16** | **0.62** | 88.36 | 53.61 | 102.67 | 38.31 | 123.87 | 0.00 | 111.46 | 130.18 | 76.27 | 168.16 | 96.82 | 95.62 | 50.52 | 91.41 | 113.51 |
| Q9GZM7 | Tubulointerstitial nephritis antigen-like | **0.56** | **0.62** | 240.41 | 391.40 | 134.79 | 287.48 | 45.73 | 56.31 | 938.39 | 118.75 | 42.89 | 12.51 | 38.50 | 37.16 | 0.00 | 720.56 | 0.00 |
| Q96P56 | Cation channel sperm-associated protein 2 | **1.35** | **0.62** | 79.95 | 89.49 | 108.14 | 91.66 | 202.00 | 0.00 | 0.00 | 141.53 | 56.22 | 127.38 | 0.00 | 224.67 | 175.13 | 121.66 | 0.00 |
| P11586 | C-1-tetrahydrofolate synthase, cytoplasmic | **1.37** | **0.62** | 20.26 | 18.71 | 27.83 | 28.40 | 33.05 | 0.00 | 38.13 | 30.12 | 0.00 | 37.85 | 31.58 | 0.00 | 0.00 | 21.48 | 76.08 |
| P34897 | Serine hydroxymethyltransferase, mitochondrial | **1.36** | **0.62** | 27.78 | 36.71 | 37.76 | 28.48 | 0.00 | 0.00 | 22.63 | 89.59 | 26.68 | 29.78 | 30.34 | 35.26 | 0.00 | 43.72 | 87.44 |
| Q16658 | Fascin | **1.36** | **0.62** | 95.46 | 104.92 | 129.37 | 113.95 | 0.00 | 0.00 | 167.47 | 237.24 | 72.61 | 62.42 | 0.00 | 116.49 | 172.00 | 330.65 | 94.67 |
| P14868 | Aspartate--tRNA ligase, cytoplasmic | **0.84** | **0.62** | 52.53 | 34.57 | 44.31 | 18.05 | 23.52 | 14.59 | 49.34 | 91.42 | 83.77 | 40.77 | 51.42 | 39.91 | 18.01 | 73.47 | 42.27 |
| P60900 | Proteasome subunit alpha type-6 | **1.20** | **0.62** | 43.32 | 26.02 | 51.99 | 29.83 | 49.07 | 0.00 | 64.49 | 61.93 | 41.13 | 57.54 | 40.09 | 88.28 | 0.00 | 60.24 | 65.77 |
| Q96HC4 | PDZ and LIM domain protein 5 | **1.39** | **0.63** | 13.98 | 13.90 | 19.37 | 21.28 | 16.82 | 0.00 | 31.96 | 21.13 | 0.00 | 0.00 | 0.00 | 37.16 | 37.24 | 41.81 | 0.00 |
| P55786 | Puromycin-sensitive aminopeptidase | **1.15** | **0.63** | 46.82 | 18.51 | 54.08 | 27.32 | 63.30 | 26.22 | 30.92 | 67.36 | 46.33 | 35.96 | 102.23 | 34.49 | 30.75 | 66.66 | 54.36 |
| Q96AE4 | Far upstream element-binding protein 1 | **0.86** | **0.63** | 75.43 | 37.62 | 64.85 | 32.30 | 128.48 | 96.32 | 68.13 | 50.45 | 33.80 | 87.07 | 112.39 | 42.85 | 36.69 | 77.34 | 32.77 |
| Q99832 | T-complex protein 1 subunit eta | **1.20** | **0.63** | 49.01 | 36.03 | 58.74 | 28.46 | 59.66 | 0.00 | 81.51 | 80.26 | 23.61 | 107.37 | 50.41 | 55.44 | 22.95 | 45.40 | 70.88 |
| Q6NXR0 | Interferon-inducible GTPase 5 | **0.88** | **0.63** | 446.60 | 130.74 | 395.02 | 195.70 | 486.84 | 620.30 | 453.28 | 414.38 | 258.19 | 476.38 | 0.00 | 460.80 | 487.36 | 428.69 | 516.89 |
| Q9BSC4 | Nucleolar protein 10 | **0.88** | **0.63** | 446.60 | 130.74 | 395.02 | 195.70 | 486.84 | 620.30 | 453.28 | 414.38 | 258.19 | 476.38 | 0.00 | 460.80 | 487.36 | 428.69 | 516.89 |
| Q9UL01 | Dermatan-sulfate epimerase | **0.88** | **0.63** | 446.60 | 130.74 | 395.02 | 195.70 | 486.84 | 620.30 | 453.28 | 414.38 | 258.19 | 476.38 | 0.00 | 460.80 | 487.36 | 428.69 | 516.89 |
| P13637 | Sodium/potassium-transporting ATPase subunit alpha-3 | **1.18** | **0.63** | 62.56 | 41.51 | 73.53 | 31.45 | 74.07 | 0.00 | 112.84 | 75.54 | 50.37 | 124.16 | 87.81 | 62.68 | 43.55 | 40.62 | 82.38 |
| A2BFH1 | Peptidyl-prolyl cis-trans isomerase A-like 4G | **1.34** | **0.63** | 308.13 | 339.03 | 412.98 | 360.96 | 289.25 | 0.00 | 0.00 | 809.86 | 441.53 | 836.63 | 400.11 | 471.42 | 0.00 | 769.74 | 0.00 |
| F5H284 | Peptidyl-prolyl cis-trans isomerase A-like 4D | **1.34** | **0.63** | 308.13 | 339.03 | 412.98 | 360.96 | 289.25 | 0.00 | 0.00 | 809.86 | 441.53 | 836.63 | 400.11 | 471.42 | 0.00 | 769.74 | 0.00 |
| Q8TF09 | Dynein light chain roadblock-type 2 | **0.72** | **0.63** | 27.47 | 28.42 | 19.87 | 22.90 | 37.48 | 67.43 | 32.43 | 0.00 | 0.00 | 0.00 | 39.66 | 51.04 | 0.00 | 28.51 | 0.00 |
| Q9NP97 | Dynein light chain roadblock-type 1 | **0.72** | **0.63** | 27.47 | 28.42 | 19.87 | 22.90 | 37.48 | 67.43 | 32.43 | 0.00 | 0.00 | 0.00 | 39.66 | 51.04 | 0.00 | 28.51 | 0.00 |
| Q13885 | Tubulin beta-2A chain | **1.15** | **0.63** | 736.22 | 405.88 | 849.67 | 357.89 | 692.89 | 264.18 | 878.65 | 1337.67 | 507.69 | 547.26 | 919.34 | 451.98 | 679.98 | 1385.86 | 1113.60 |
| Q9BVA1 | Tubulin beta-2B chain | **1.15** | **0.63** | 736.22 | 405.88 | 849.67 | 357.89 | 692.89 | 264.18 | 878.65 | 1337.67 | 507.69 | 547.26 | 919.34 | 451.98 | 679.98 | 1385.86 | 1113.60 |
| Q13509 | Tubulin beta-3 chain | **1.14** | **0.64** | 741.76 | 354.32 | 848.94 | 367.64 | 766.43 | 281.68 | 915.56 | 1208.32 | 536.82 | 616.21 | 858.78 | 401.93 | 679.98 | 1412.15 | 1124.56 |
| P18505 | Gamma-aminobutyric acid receptor subunit beta-1 | **0.69** | **0.64** | 119.26 | 113.33 | 82.82 | 130.67 | 147.43 | 0.00 | 227.10 | 221.77 | 0.00 | 0.00 | 0.00 | 0.00 | 0.00 | 209.23 | 287.67 |
| P48147 | Prolyl endopeptidase | **1.59** | **0.64** | 19.79 | 18.55 | 31.46 | 50.44 | 26.67 | 0.00 | 33.80 | 38.47 | 0.00 | 0.00 | 22.80 | 0.00 | 0.00 | 129.72 | 36.21 |
| Q96C90 | Protein phosphatase 1 regulatory subunit 14B | **0.70** | **0.64** | 10.79 | 7.31 | 7.52 | 13.40 | 20.57 | 0.00 | 11.08 | 11.96 | 10.37 | 3.71 | 7.15 | 0.00 | 0.00 | 0.00 | 34.23 |
| P62888 | 60S ribosomal protein L30 | **1.18** | **0.64** | 107.25 | 77.39 | 126.16 | 41.14 | 71.06 | 35.83 | 185.29 | 196.26 | 47.83 | 80.85 | 164.96 | 104.69 | 83.08 | 155.94 | 167.41 |
| Q9Y624 | Junctional adhesion molecule A | **1.32** | **0.64** | 29.64 | 30.23 | 39.06 | 34.05 | 0.00 | 0.00 | 28.91 | 66.58 | 52.70 | 38.56 | 44.05 | 0.00 | 0.00 | 73.37 | 78.36 |
| O95757 | Heat shock 70 kDa protein 4L | **1.72** | **0.64** | 38.38 | 36.57 | 66.07 | 124.24 | 58.71 | 0.00 | 80.70 | 52.47 | 0.00 | 12.59 | 0.00 | 0.00 | 313.47 | 0.00 | 70.35 |
| P61353 | 60S ribosomal protein L27 | **1.27** | **0.64** | 61.57 | 47.82 | 78.48 | 65.84 | 33.13 | 0.00 | 118.59 | 97.45 | 58.66 | 98.92 | 98.01 | 0.00 | 0.00 | 107.73 | 166.25 |
| O15145 | Actin-related protein 2/3 complex subunit 3 | **1.12** | **0.64** | 85.40 | 42.24 | 95.32 | 26.19 | 151.86 | 57.86 | 57.50 | 103.58 | 56.22 | 75.65 | 128.39 | 116.82 | 109.30 | 77.84 | 63.91 |
| Q03135 | Caveolin-1 | **0.77** | **0.65** | 63.10 | 60.26 | 48.60 | 31.57 | 126.76 | 110.97 | 77.77 | 0.00 | 0.00 | 61.86 | 54.05 | 0.00 | 71.07 | 83.00 | 21.64 |
| Q96HE9 | Proline-rich protein 11 | **0.73** | **0.65** | 1415.34 | 1761.75 | 1036.60 | 804.14 | 0.00 | 4452.32 | 1285.45 | 497.48 | 841.45 | 1073.06 | 518.80 | 0.00 | 2130.78 | 1797.18 | 699.75 |
| O00410 | Importin-5 | **1.15** | **0.65** | 37.93 | 9.95 | 43.60 | 25.01 | 41.09 | 47.46 | 36.70 | 42.86 | 21.51 | 0.00 | 62.20 | 45.32 | 46.63 | 71.95 | 35.48 |
| P15880 | 40S ribosomal protein S2 | **1.24** | **0.65** | 108.60 | 83.24 | 134.41 | 95.96 | 87.20 | 0.00 | 195.45 | 189.30 | 71.03 | 105.63 | 165.40 | 55.34 | 21.37 | 169.38 | 289.34 |
| P62910 | 60S ribosomal protein L32 | **0.73** | **0.65** | 66.43 | 78.97 | 48.72 | 44.87 | 50.81 | 0.00 | 189.92 | 91.41 | 0.00 | 0.00 | 89.66 | 30.57 | 0.00 | 102.29 | 69.81 |
| P38919 | Eukaryotic initiation factor 4A-III | **0.80** | **0.65** | 114.88 | 72.93 | 92.34 | 85.43 | 95.29 | 0.00 | 150.21 | 136.80 | 192.11 | 86.55 | 92.32 | 0.00 | 0.00 | 162.43 | 212.73 |
| Q14019 | Coactosin-like protein | **1.40** | **0.65** | 19.94 | 27.31 | 27.86 | 29.01 | 0.00 | 0.00 | 0.00 | 50.56 | 49.15 | 78.30 | 0.00 | 36.84 | 22.55 | 0.00 | 29.50 |
| Q96CW1 | AP-2 complex subunit mu | **1.34** | **0.65** | 10.92 | 15.24 | 14.66 | 11.58 | 0.00 | 0.00 | 23.07 | 31.52 | 0.00 | 0.00 | 19.04 | 23.43 | 20.15 | 25.35 | 0.00 |
| P53618 | Coatomer subunit beta | **1.29** | **0.65** | 19.18 | 19.59 | 24.76 | 20.12 | 19.12 | 0.00 | 32.80 | 43.97 | 0.00 | 0.00 | 43.56 | 0.00 | 31.47 | 29.30 | 44.24 |
| P12830 | Cadherin-1 | **1.44** | **0.65** | 51.42 | 41.36 | 73.84 | 100.44 | 114.76 | 0.00 | 39.47 | 46.70 | 56.17 | 104.34 | 82.96 | 0.00 | 0.00 | 0.00 | 255.71 |
| Q9Y224 | UPF0568 protein C14orf166 | **1.22** | **0.66** | 26.51 | 26.61 | 32.45 | 9.69 | 62.18 | 0.00 | 33.74 | 36.64 | 0.00 | 21.31 | 28.65 | 39.06 | 21.96 | 42.54 | 41.16 |
| Q8IVV2 | Lipoxygenase homology domain-containing protein 1 | **0.71** | **0.66** | 286.15 | 273.26 | 201.77 | 324.34 | 603.03 | 0.00 | 383.13 | 444.59 | 0.00 | 0.00 | 0.00 | 0.00 | 742.12 | 468.49 | 0.00 |
| Q9NR28 | Diablo homolog, mitochondrial | **1.23** | **0.66** | 24.31 | 22.69 | 29.79 | 17.10 | 33.77 | 0.00 | 0.00 | 47.19 | 40.60 | 44.66 | 39.15 | 0.00 | 19.08 | 34.89 | 40.96 |
| Q8N163 | DBIRD complex subunit KIAA1967 | **0.74** | **0.66** | 26.33 | 25.94 | 19.59 | 22.96 | 28.12 | 0.00 | 53.81 | 49.71 | 0.00 | 31.93 | 31.50 | 0.00 | 0.00 | 0.00 | 54.11 |
| P14543 | Nidogen-1 | **0.69** | **0.66** | 46.09 | 55.18 | 31.70 | 49.44 | 85.45 | 0.00 | 122.44 | 22.54 | 0.00 | 24.07 | 0.00 | 39.05 | 0.00 | 127.08 | 0.00 |
| Q96QK1 | Vacuolar protein sorting-associated protein 35 | **1.17** | **0.66** | 50.07 | 33.58 | 58.66 | 29.16 | 55.75 | 0.00 | 59.26 | 92.85 | 42.51 | 62.60 | 114.80 | 35.41 | 39.21 | 52.51 | 47.41 |
| P60842 | Eukaryotic initiation factor 4A-I | **1.13** | **0.66** | 158.29 | 71.93 | 179.26 | 79.29 | 160.29 | 44.26 | 197.04 | 236.78 | 153.10 | 150.41 | 154.11 | 100.57 | 116.22 | 259.22 | 295.05 |
| Q01130 | Serine/arginine-rich splicing factor 2 | **1.21** | **0.66** | 60.77 | 45.77 | 73.34 | 45.92 | 56.71 | 37.50 | 115.94 | 93.69 | 0.00 | 113.41 | 0.00 | 89.67 | 55.06 | 125.14 | 56.77 |
| P01766 | Ig heavy chain V-III region BRO | **0.73** | **0.66** | 206.58 | 291.82 | 150.43 | 85.57 | 36.08 | 705.46 | 225.02 | 66.33 | 0.00 | 103.73 | 118.86 | 157.10 | 319.62 | 99.72 | 103.58 |
| P04920 | Anion exchange protein 2 | **0.61** | **0.66** | 398.81 | 525.52 | 243.72 | 596.98 | 0.00 | 389.93 | 1282.32 | 0.00 | 321.80 | 0.00 | 1462.30 | 0.00 | 0.00 | 0.00 | 0.00 |
| Q96QV6 | Histone H2A type 1-A | **1.09** | **0.66** | 6084.24 | 1461.34 | 6613.03 | 2244.97 | 5374.78 | 8640.06 | 5801.96 | 5621.27 | 4983.12 | 7405.49 | 7035.92 | 10439.44 | 4056.62 | 5622.76 | 5117.94 |
| P11166 | Solute carrier family 2, facilitated glucose transporter member 1 | **1.46** | **0.66** | 97.18 | 89.64 | 141.61 | 204.36 | 21.24 | 52.63 | 41.05 | 132.36 | 238.61 | 112.62 | 158.06 | 0.00 | 16.87 | 22.96 | 539.13 |
| P48741 | Putative heat shock 70 kDa protein 7 | **0.88** | **0.66** | 789.74 | 228.43 | 692.32 | 464.61 | 907.68 | 650.28 | 888.15 | 1036.53 | 466.04 | 809.17 | 216.89 | 344.90 | 328.75 | 1313.08 | 1141.11 |
| P34931 | Heat shock 70 kDa protein 1-like | **0.87** | **0.66** | 801.18 | 245.25 | 698.53 | 487.39 | 971.36 | 531.10 | 925.96 | 1036.53 | 540.94 | 832.53 | 255.26 | 311.72 | 268.85 | 1313.08 | 1209.77 |
| Q9BXP5 | Serrate RNA effector molecule homolog | **0.75** | **0.67** | 16.37 | 15.21 | 12.26 | 15.18 | 22.93 | 0.00 | 30.63 | 28.31 | 0.00 | 21.73 | 0.00 | 0.00 | 0.00 | 15.02 | 36.83 |
| P31153 | S-adenosylmethionine synthase isoform type-2 | **0.74** | **0.67** | 34.69 | 35.61 | 25.77 | 30.81 | 34.72 | 0.00 | 80.82 | 57.89 | 0.00 | 57.89 | 0.00 | 0.00 | 0.00 | 67.08 | 29.64 |
| O75533 | Splicing factor 3B subunit 1 | **1.32** | **0.67** | 31.30 | 36.78 | 41.38 | 37.85 | 0.00 | 0.00 | 69.10 | 72.99 | 14.44 | 89.24 | 39.65 | 0.00 | 0.00 | 40.01 | 79.38 |
| O60282 | Kinesin heavy chain isoform 5C | **1.27** | **0.67** | 18.34 | 17.59 | 23.38 | 19.52 | 21.90 | 0.00 | 33.53 | 36.28 | 0.00 | 24.16 | 34.64 | 0.00 | 0.00 | 34.33 | 47.14 |
| Q12840 | Kinesin heavy chain isoform 5A | **1.27** | **0.67** | 18.34 | 17.59 | 23.38 | 19.52 | 21.90 | 0.00 | 33.53 | 36.28 | 0.00 | 24.16 | 34.64 | 0.00 | 0.00 | 34.33 | 47.14 |
| Q9P0L0 | Vesicle-associated membrane protein-associated protein A | **0.81** | **0.67** | 61.68 | 44.40 | 49.82 | 43.74 | 105.97 | 0.00 | 93.68 | 76.60 | 32.14 | 45.22 | 97.35 | 0.00 | 0.00 | 59.33 | 97.06 |
| P23246 | Splicing factor, proline- and glutamine-rich | **1.13** | **0.67** | 148.07 | 62.19 | 166.79 | 74.89 | 126.26 | 86.21 | 204.29 | 223.56 | 100.05 | 233.04 | 168.41 | 58.31 | 94.03 | 214.49 | 232.47 |
| P15311 | Ezrin | **0.91** | **0.67** | 349.31 | 149.02 | 317.76 | 83.47 | 501.17 | 188.72 | 195.86 | 470.85 | 389.97 | 267.83 | 251.25 | 266.01 | 278.90 | 387.38 | 455.17 |
| P18135 | Ig kappa chain V-III region HAH | **1.47** | **0.67** | 107.70 | 163.98 | 158.23 | 205.95 | 0.00 | 393.81 | 71.53 | 0.00 | 73.16 | 59.25 | 100.45 | 72.44 | 566.27 | 150.98 | 0.00 |
| P18136 | Ig kappa chain V-III region HIC | **1.47** | **0.67** | 107.70 | 163.98 | 158.23 | 205.95 | 0.00 | 393.81 | 71.53 | 0.00 | 73.16 | 59.25 | 100.45 | 72.44 | 566.27 | 150.98 | 0.00 |
| P16104 | Histone H2AX | **1.09** | **0.67** | 6102.26 | 1457.54 | 6621.55 | 2248.67 | 5374.78 | 8640.06 | 5892.06 | 5621.27 | 4983.12 | 7456.62 | 7035.92 | 10439.44 | 4056.62 | 5622.76 | 5117.94 |
| Q99623 | Prohibitin-2 | **1.21** | **0.67** | 118.22 | 74.51 | 143.35 | 106.99 | 125.70 | 28.59 | 212.37 | 163.11 | 61.32 | 212.44 | 125.20 | 79.91 | 44.95 | 71.77 | 325.83 |
| Q01105 | Protein SET | **0.88** | **0.67** | 145.61 | 83.75 | 127.57 | 51.60 | 198.23 | 176.30 | 201.28 | 152.26 | 0.00 | 103.31 | 182.64 | 87.58 | 68.59 | 127.83 | 195.50 |
| Q15056 | Eukaryotic translation initiation factor 4H | **0.73** | **0.67** | 21.79 | 19.97 | 15.80 | 24.56 | 33.43 | 0.00 | 38.28 | 37.23 | 0.00 | 0.00 | 0.00 | 0.00 | 0.00 | 44.31 | 50.49 |
| Q13283 | Ras GTPase-activating protein-binding protein 1 | **0.79** | **0.67** | 26.16 | 17.29 | 20.70 | 22.92 | 26.49 | 0.00 | 44.74 | 38.12 | 21.45 | 40.87 | 0.00 | 0.00 | 0.00 | 36.43 | 46.92 |
| P80723 | Brain acid soluble protein 1 | **1.15** | **0.67** | 168.21 | 123.34 | 193.96 | 69.94 | 161.43 | 264.90 | 0.00 | 107.12 | 307.61 | 134.52 | 181.54 | 179.32 | 234.40 | 311.47 | 122.49 |
| Q13765 | Nascent polypeptide-associated complex subunit alpha | **0.91** | **0.67** | 95.42 | 23.79 | 86.66 | 39.08 | 89.71 | 86.59 | 87.57 | 136.96 | 76.27 | 78.99 | 140.20 | 50.60 | 40.17 | 88.03 | 121.96 |
| O00571 | ATP-dependent RNA helicase DDX3X | **1.21** | **0.67** | 37.16 | 32.65 | 45.05 | 27.59 | 19.75 | 0.00 | 69.21 | 73.90 | 22.94 | 21.95 | 44.13 | 31.49 | 17.03 | 69.17 | 86.54 |
| P49755 | Transmembrane emp24 domain-containing protein 10 | **1.15** | **0.68** | 57.10 | 46.01 | 65.84 | 18.47 | 82.82 | 0.00 | 44.92 | 120.40 | 37.38 | 56.39 | 55.69 | 48.05 | 58.50 | 79.79 | 96.63 |
| Q6IBS0 | Twinfilin-2 | **0.77** | **0.68** | 27.52 | 26.24 | 21.10 | 23.65 | 35.55 | 0.00 | 45.13 | 56.92 | 0.00 | 0.00 | 0.00 | 0.00 | 39.37 | 51.13 | 36.08 |
| Q99460 | 26S proteasome non-ATPase regulatory subunit 1 | **0.74** | **0.68** | 19.22 | 20.01 | 14.23 | 18.64 | 26.33 | 0.00 | 22.19 | 47.56 | 0.00 | 0.00 | 15.26 | 0.00 | 0.00 | 23.67 | 46.47 |
| P0CG04 | Ig lambda-1 chain C regions | **1.27** | **0.68** | 845.71 | 1006.55 | 1071.08 | 742.47 | 273.95 | 2632.86 | 571.01 | 458.56 | 292.18 | 678.02 | 484.22 | 569.68 | 2296.47 | 1684.68 | 713.42 |
| P62280 | 40S ribosomal protein S11 | **1.20** | **0.68** | 79.04 | 61.46 | 94.46 | 58.41 | 59.16 | 0.00 | 120.46 | 158.28 | 57.27 | 112.87 | 90.21 | 0.00 | 64.39 | 129.84 | 169.43 |
| Q01813 | 6-phosphofructokinase type C | **1.23** | **0.68** | 24.46 | 25.41 | 30.16 | 19.21 | 46.74 | 21.45 | 0.00 | 54.12 | 0.00 | 31.54 | 32.90 | 35.12 | 0.00 | 22.29 | 59.14 |
| P00450 | Ceruloplasmin | **1.29** | **0.68** | 63.99 | 55.43 | 82.23 | 81.41 | 63.11 | 157.13 | 37.68 | 50.35 | 11.69 | 41.54 | 0.00 | 201.21 | 167.44 | 40.72 | 42.50 |
| Q7Z3Y7 | Keratin, type I cytoskeletal 28 | **1.19** | **0.68** | 361.00 | 142.84 | 429.25 | 332.95 | 558.56 | 396.50 | 159.74 | 332.55 | 357.62 | 483.42 | 263.73 | 1063.41 | 112.39 | 335.41 | 317.14 |
| Q8NHW5 | 60S acidic ribosomal protein P0-like | **0.85** | **0.68** | 135.79 | 63.30 | 115.98 | 86.55 | 94.32 | 64.78 | 188.16 | 214.40 | 117.28 | 121.08 | 63.48 | 0.00 | 95.28 | 164.09 | 251.92 |
| B9A064 | Immunoglobulin lambda-like polypeptide 5 | **1.27** | **0.68** | 834.11 | 1014.82 | 1058.40 | 751.13 | 273.95 | 2632.86 | 571.01 | 458.56 | 234.19 | 601.93 | 484.22 | 569.68 | 2296.47 | 1684.68 | 713.42 |
| Q9Y2B0 | Protein canopy homolog 2 | **1.27** | **0.69** | 22.24 | 20.75 | 28.18 | 25.36 | 30.18 | 0.00 | 41.51 | 39.50 | 0.00 | 41.25 | 66.17 | 0.00 | 0.00 | 30.71 | 30.95 |
| P15531 | Nucleoside diphosphate kinase A | **0.84** | **0.69** | 647.07 | 563.86 | 546.68 | 163.41 | 434.73 | 214.47 | 570.45 | 1629.68 | 386.06 | 763.18 | 691.16 | 418.36 | 325.94 | 556.57 | 524.87 |
| A6NNZ2 | Tubulin beta-8 chain-like protein LOC260334 | **1.16** | **0.69** | 468.22 | 307.24 | 543.32 | 287.89 | 338.04 | 197.03 | 571.41 | 957.89 | 276.75 | 403.55 | 300.70 | 316.26 | 442.87 | 1004.05 | 792.51 |
| Q15717 | ELAV-like protein 1 | **1.19** | **0.69** | 43.62 | 30.47 | 51.72 | 33.31 | 28.07 | 0.00 | 54.19 | 79.69 | 56.17 | 90.41 | 65.17 | 0.00 | 24.97 | 56.67 | 73.08 |
| Q92526 | T-complex protein 1 subunit zeta-2 | **1.25** | **0.69** | 36.67 | 33.65 | 45.67 | 37.51 | 65.83 | 0.00 | 56.24 | 61.27 | 0.00 | 78.92 | 45.41 | 0.00 | 0.00 | 69.68 | 80.04 |
| P38117 | Electron transfer flavoprotein subunit beta | **0.85** | **0.69** | 61.32 | 44.18 | 52.08 | 30.21 | 87.07 | 0.00 | 99.12 | 91.46 | 28.95 | 70.96 | 57.88 | 49.92 | 0.00 | 44.28 | 89.44 |
| P06576 | ATP synthase subunit beta, mitochondrial | **1.13** | **0.69** | 332.30 | 209.23 | 375.26 | 135.54 | 439.20 | 46.88 | 490.53 | 511.23 | 173.67 | 486.37 | 366.57 | 303.91 | 205.20 | 311.04 | 578.47 |
| P61224 | Ras-related protein Rap-1b | **1.19** | **0.69** | 76.46 | 71.95 | 90.92 | 44.18 | 137.74 | 0.00 | 99.23 | 145.32 | 0.00 | 28.02 | 90.46 | 158.36 | 118.12 | 78.87 | 71.68 |
| Q92764 | Keratin, type I cuticular Ha5 | **0.86** | **0.69** | 744.22 | 360.32 | 638.58 | 471.15 | 1084.40 | 1001.08 | 294.41 | 920.06 | 421.14 | 525.17 | 667.08 | 1532.46 | 169.04 | 566.70 | 371.00 |
| Q92817 | Envoplakin | **1.38** | **0.69** | 16.45 | 18.81 | 22.75 | 29.64 | 45.69 | 0.00 | 15.90 | 0.00 | 20.69 | 13.93 | 25.49 | 0.00 | 0.00 | 17.36 | 79.69 |
| P83916 | Chromobox protein homolog 1 | **1.25** | **0.69** | 20.72 | 19.20 | 26.00 | 22.90 | 38.53 | 0.00 | 29.39 | 35.67 | 0.00 | 22.06 | 56.11 | 0.00 | 0.00 | 36.25 | 41.56 |
| P10153 | Non-secretory ribonuclease | **1.37** | **0.69** | 69.78 | 127.33 | 95.62 | 82.18 | 55.42 | 293.46 | 0.00 | 0.00 | 0.00 | 55.42 | 64.58 | 125.62 | 240.80 | 87.33 | 0.00 |
| P78347 | General transcription factor II-I | **0.79** | **0.69** | 37.84 | 39.08 | 29.78 | 26.55 | 36.78 | 0.00 | 88.47 | 63.97 | 0.00 | 61.02 | 27.89 | 0.00 | 0.00 | 32.32 | 57.43 |
| P63208 | S-phase kinase-associated protein 1 | **0.81** | **0.69** | 41.82 | 36.16 | 34.05 | 27.13 | 98.26 | 46.48 | 0.00 | 25.50 | 38.85 | 54.44 | 52.95 | 57.80 | 0.00 | 0.00 | 39.12 |
| Q9BUF5 | Tubulin beta-6 chain | **1.15** | **0.69** | 586.78 | 358.22 | 674.61 | 356.60 | 449.35 | 185.50 | 710.28 | 1134.92 | 453.84 | 450.41 | 581.38 | 280.66 | 564.00 | 1274.78 | 896.42 |
| P42677 | 40S ribosomal protein S27 | **0.77** | **0.70** | 60.98 | 60.89 | 47.07 | 53.53 | 0.00 | 0.00 | 91.05 | 140.59 | 73.23 | 98.26 | 0.00 | 0.00 | 0.00 | 69.64 | 114.55 |
| P19367 | Hexokinase-1 | **1.17** | **0.70** | 48.13 | 35.60 | 56.07 | 29.86 | 52.47 | 0.00 | 93.79 | 65.18 | 29.19 | 81.03 | 102.59 | 46.60 | 25.34 | 31.88 | 48.99 |
| Q92626 | Peroxidasin homolog | **0.65** | **0.70** | 25.92 | 51.96 | 16.78 | 19.43 | 0.00 | 0.00 | 118.47 | 11.11 | 0.00 | 10.55 | 6.93 | 17.89 | 10.72 | 54.62 | 0.00 |
| P06727 | Apolipoprotein A-IV | **0.68** | **0.70** | 44.42 | 77.16 | 30.13 | 38.49 | 43.91 | 178.20 | 0.00 | 0.00 | 0.00 | 0.00 | 8.40 | 33.52 | 101.22 | 37.63 | 0.00 |
| P0C0S5 | Histone H2A.Z | **1.06** | **0.70** | 5760.96 | 1659.60 | 6122.52 | 1344.13 | 8041.24 | 5817.18 | 5916.51 | 5673.58 | 3356.28 | 7501.34 | 7171.24 | 7061.66 | 4095.48 | 5721.25 | 5184.16 |
| Q71UI9 | Histone H2A.V | **1.06** | **0.70** | 5760.96 | 1659.60 | 6122.52 | 1344.13 | 8041.24 | 5817.18 | 5916.51 | 5673.58 | 3356.28 | 7501.34 | 7171.24 | 7061.66 | 4095.48 | 5721.25 | 5184.16 |
| P46777 | 60S ribosomal protein L5 | **1.25** | **0.70** | 70.13 | 72.76 | 87.56 | 71.65 | 61.13 | 0.00 | 137.12 | 152.42 | 0.00 | 165.33 | 101.96 | 0.00 | 0.00 | 111.10 | 146.96 |
| P10909 | Clusterin | **0.74** | **0.70** | 163.45 | 198.70 | 121.13 | 154.41 | 97.08 | 516.81 | 92.55 | 67.48 | 43.34 | 30.23 | 56.61 | 423.88 | 115.44 | 100.64 | 0.00 |
| Q9NQ39 | Putative 40S ribosomal protein S10-like | **1.25** | **0.70** | 48.83 | 48.34 | 61.22 | 54.22 | 51.32 | 0.00 | 91.81 | 101.01 | 0.00 | 109.27 | 49.77 | 0.00 | 0.00 | 80.88 | 127.38 |
| P12268 | Inosine-5'-monophosphate dehydrogenase 2 | **1.27** | **0.70** | 35.89 | 20.60 | 45.45 | 50.09 | 42.87 | 0.00 | 52.76 | 43.37 | 40.46 | 65.36 | 34.10 | 39.17 | 0.00 | 0.00 | 134.05 |
| P55854 | Small ubiquitin-related modifier 3 | **0.93** | **0.70** | 362.92 | 117.02 | 338.53 | 87.68 | 499.62 | 387.47 | 422.20 | 190.78 | 314.53 | 335.98 | 494.52 | 325.20 | 308.18 | 342.70 | 224.59 |
| P61956 | Small ubiquitin-related modifier 2 | **0.93** | **0.70** | 362.92 | 117.02 | 338.53 | 87.68 | 499.62 | 387.47 | 422.20 | 190.78 | 314.53 | 335.98 | 494.52 | 325.20 | 308.18 | 342.70 | 224.59 |
| Q6EEV6 | Small ubiquitin-related modifier 4 | **0.93** | **0.70** | 362.92 | 117.02 | 338.53 | 87.68 | 499.62 | 387.47 | 422.20 | 190.78 | 314.53 | 335.98 | 494.52 | 325.20 | 308.18 | 342.70 | 224.59 |
| Q6NUK1 | Calcium-binding mitochondrial carrier protein SCaMC-1 | **0.78** | **0.70** | 43.04 | 30.25 | 33.65 | 45.32 | 51.44 | 0.00 | 24.76 | 70.40 | 68.61 | 19.58 | 96.76 | 0.00 | 0.00 | 0.00 | 85.55 |
| P01622 | Ig kappa chain V-III region Ti | **0.71** | **0.70** | 308.45 | 538.87 | 219.00 | 145.35 | 0.00 | 1267.57 | 147.98 | 46.83 | 79.85 | 96.53 | 142.55 | 222.41 | 477.87 | 276.05 | 98.57 |
| P04206 | Ig kappa chain V-III region GOL | **0.71** | **0.70** | 308.45 | 538.87 | 219.00 | 145.35 | 0.00 | 1267.57 | 147.98 | 46.83 | 79.85 | 96.53 | 142.55 | 222.41 | 477.87 | 276.05 | 98.57 |
| Q07021 | Complement component 1 Q subcomponent-binding protein, mitochondrial | **0.76** | **0.70** | 77.32 | 86.36 | 59.12 | 67.68 | 48.18 | 0.00 | 177.60 | 160.80 | 0.00 | 74.43 | 40.84 | 0.00 | 0.00 | 56.51 | 182.96 |
| Q86UE4 | Protein LYRIC | **1.16** | **0.70** | 26.87 | 17.11 | 31.28 | 19.64 | 30.67 | 46.51 | 23.93 | 33.26 | 0.00 | 34.22 | 61.75 | 32.46 | 27.82 | 0.00 | 31.46 |
| Q71UM5 | 40S ribosomal protein S27-like | **0.78** | **0.71** | 69.34 | 68.45 | 54.00 | 61.60 | 0.00 | 0.00 | 132.85 | 140.59 | 73.23 | 98.26 | 0.00 | 0.00 | 0.00 | 87.01 | 138.70 |
| P46459 | Vesicle-fusing ATPase | **1.33** | **0.71** | 17.69 | 16.60 | 23.58 | 30.04 | 34.12 | 0.00 | 23.47 | 30.86 | 0.00 | 0.00 | 73.46 | 0.00 | 0.00 | 25.66 | 42.38 |
| P14866 | Heterogeneous nuclear ribonucleoprotein L | **1.24** | **0.71** | 70.22 | 66.65 | 87.02 | 74.73 | 35.94 | 0.00 | 147.63 | 135.24 | 32.28 | 120.51 | 46.40 | 0.00 | 19.94 | 161.44 | 173.84 |
| Q16576 | Histone-binding protein RBBP7 | **0.78** | **0.71** | 58.94 | 70.69 | 46.10 | 37.66 | 33.88 | 0.00 | 54.60 | 180.45 | 25.75 | 36.04 | 38.77 | 0.00 | 20.22 | 82.12 | 99.44 |
| P51991 | Heterogeneous nuclear ribonucleoprotein A3 | **0.86** | **0.71** | 147.31 | 53.43 | 126.53 | 116.96 | 126.34 | 85.16 | 205.78 | 200.82 | 118.45 | 252.18 | 30.12 | 0.00 | 46.87 | 163.49 | 266.52 |
| Q9UL25 | Ras-related protein Rab-21 | **1.34** | **0.71** | 8.47 | 7.94 | 11.37 | 15.16 | 11.22 | 0.00 | 15.81 | 15.33 | 0.00 | 7.30 | 33.20 | 0.00 | 0.00 | 0.00 | 27.73 |
| P53007 | Tricarboxylate transport protein, mitochondrial | **0.84** | **0.71** | 31.33 | 20.20 | 26.23 | 23.26 | 28.24 | 0.00 | 32.45 | 54.52 | 41.42 | 55.28 | 19.89 | 0.00 | 0.00 | 40.45 | 41.78 |
| P13929 | Beta-enolase | **1.12** | **0.71** | 620.95 | 377.59 | 693.68 | 248.23 | 909.69 | 214.20 | 534.07 | 1106.11 | 340.69 | 578.46 | 552.50 | 591.98 | 562.13 | 1190.85 | 686.19 |
| P02768 | Serum albumin | **0.88** | **0.71** | 16898.05 | 10303.68 | 14945.92 | 6511.22 | 14703.05 | 34734.67 | 13486.57 | 8019.50 | 13546.45 | 13417.45 | 11364.48 | 16259.08 | 26956.52 | 13763.41 | 7914.59 |
| P47985 | Cytochrome b-c1 complex subunit Rieske, mitochondrial | **1.43** | **0.71** | 13.94 | 13.42 | 19.94 | 32.99 | 16.42 | 0.00 | 27.81 | 25.47 | 0.00 | 78.12 | 41.55 | 0.00 | 0.00 | 0.00 | 0.00 |
| P15153 | Ras-related C3 botulinum toxin substrate 2 | **1.08** | **0.71** | 133.38 | 28.82 | 144.01 | 56.38 | 154.68 | 124.43 | 120.35 | 169.92 | 97.55 | 132.62 | 205.43 | 65.58 | 92.67 | 180.43 | 187.36 |
| P62805 | Histone H4 | **1.11** | **0.72** | 6565.18 | 2794.90 | 7293.35 | 3491.71 | 4485.87 | 4787.75 | 10481.76 | 8587.30 | 4483.22 | 9823.86 | 8960.57 | 2783.99 | 2871.73 | 9072.56 | 10247.42 |
| Q96A08 | Histone H2B type 1-A | **1.09** | **0.72** | 4348.69 | 2007.98 | 4754.78 | 1600.40 | 3209.13 | 6768.11 | 3704.03 | 6075.40 | 1986.78 | 4838.09 | 3447.96 | 5543.38 | 3168.25 | 4046.57 | 7484.46 |
| Q5SZL2 | Centrosomal protein of 85 kDa-like | **1.24** | **0.72** | 91.61 | 86.75 | 113.69 | 105.56 | 149.05 | 186.98 | 122.03 | 0.00 | 0.00 | 124.24 | 278.07 | 165.94 | 0.00 | 0.00 | 113.87 |
| P00491 | Purine nucleoside phosphorylase | **1.30** | **0.72** | 24.23 | 17.09 | 31.54 | 40.71 | 20.53 | 0.00 | 36.63 | 44.10 | 19.88 | 10.14 | 11.60 | 0.00 | 23.08 | 33.11 | 111.31 |
| Q14240 | Eukaryotic initiation factor 4A-II | **1.11** | **0.72** | 158.09 | 71.79 | 175.44 | 81.48 | 160.29 | 44.26 | 196.01 | 236.78 | 153.10 | 147.90 | 104.97 | 139.98 | 105.53 | 259.22 | 295.05 |
| P04350 | Tubulin beta-4A chain | **1.11** | **0.72** | 767.20 | 399.49 | 852.62 | 368.01 | 766.43 | 279.51 | 915.56 | 1337.67 | 536.82 | 616.21 | 858.78 | 423.71 | 658.27 | 1412.15 | 1146.60 |
| Q14980 | Nuclear mitotic apparatus protein 1 | **1.18** | **0.72** | 32.34 | 25.17 | 38.23 | 27.65 | 42.46 | 0.00 | 17.79 | 34.94 | 66.52 | 72.44 | 62.98 | 0.00 | 14.66 | 36.57 | 42.73 |
| Q13510 | Acid ceramidase | **0.84** | **0.72** | 62.21 | 39.09 | 52.15 | 50.00 | 107.07 | 0.00 | 64.67 | 62.18 | 77.12 | 40.88 | 91.53 | 0.00 | 0.00 | 55.01 | 125.47 |
| P61088 | Ubiquitin-conjugating enzyme E2 N | **0.93** | **0.72** | 188.92 | 78.22 | 175.92 | 36.57 | 319.92 | 186.03 | 112.12 | 169.88 | 156.66 | 228.67 | 193.96 | 179.20 | 123.02 | 148.82 | 181.87 |
| P15259 | Phosphoglycerate mutase 2 | **0.93** | **0.72** | 320.21 | 144.80 | 296.32 | 66.39 | 338.38 | 90.71 | 364.37 | 490.25 | 317.36 | 236.54 | 295.97 | 248.42 | 298.28 | 276.76 | 421.95 |
| P53999 | Activated RNA polymerase II transcriptional coactivator p15 | **1.20** | **0.73** | 31.58 | 26.35 | 38.04 | 31.63 | 50.34 | 0.00 | 9.36 | 61.76 | 36.42 | 44.01 | 44.81 | 0.00 | 0.00 | 66.42 | 72.98 |
| Q00765 | Receptor expression-enhancing protein 5 | **0.84** | **0.73** | 49.75 | 32.67 | 41.65 | 39.79 | 70.54 | 32.87 | 76.25 | 69.09 | 0.00 | 96.14 | 0.00 | 31.92 | 0.00 | 43.01 | 78.84 |
| Q5SW79 | Centrosomal protein of 170 kDa | **1.23** | **0.73** | 302.59 | 299.99 | 371.38 | 324.92 | 280.25 | 0.00 | 804.40 | 198.78 | 229.53 | 352.40 | 381.94 | 0.00 | 961.51 | 363.95 | 168.49 |
| P42166 | Lamina-associated polypeptide 2, isoform alpha | **0.81** | **0.73** | 59.43 | 36.50 | 48.38 | 59.68 | 89.07 | 0.00 | 64.64 | 88.89 | 54.55 | 124.89 | 0.00 | 0.00 | 0.00 | 46.81 | 118.60 |
| O76013 | Keratin, type I cuticular Ha6 | **0.89** | **0.73** | 789.76 | 321.59 | 699.24 | 477.03 | 1084.40 | 1001.08 | 294.41 | 920.06 | 648.83 | 889.15 | 667.08 | 1532.46 | 169.04 | 566.70 | 371.00 |
| O76014 | Keratin, type I cuticular Ha7 | **0.89** | **0.73** | 789.76 | 321.59 | 699.24 | 477.03 | 1084.40 | 1001.08 | 294.41 | 920.06 | 648.83 | 889.15 | 667.08 | 1532.46 | 169.04 | 566.70 | 371.00 |
| Q14532 | Keratin, type I cuticular Ha2 | **0.89** | **0.73** | 789.76 | 321.59 | 699.24 | 477.03 | 1084.40 | 1001.08 | 294.41 | 920.06 | 648.83 | 889.15 | 667.08 | 1532.46 | 169.04 | 566.70 | 371.00 |
| Q8WZ42 | Titin | **0.84** | **0.73** | 142.51 | 143.35 | 119.23 | 65.35 | 200.99 | 0.00 | 334.83 | 0.00 | 176.73 | 148.94 | 105.09 | 0.00 | 190.62 | 120.56 | 150.15 |
| P32119 | Peroxiredoxin-2 | **0.94** | **0.73** | 655.79 | 216.74 | 618.73 | 122.59 | 823.54 | 382.18 | 551.99 | 599.14 | 922.11 | 517.68 | 704.51 | 551.58 | 472.73 | 786.10 | 679.78 |
| P26599 | Polypyrimidine tract-binding protein 1 | **0.87** | **0.73** | 138.53 | 96.33 | 120.23 | 75.18 | 97.25 | 0.00 | 225.44 | 231.63 | 138.31 | 146.87 | 138.04 | 0.00 | 69.20 | 151.03 | 216.26 |
| P55060 | Exportin-2 | **1.08** | **0.73** | 4447.96 | 2215.87 | 4802.61 | 992.87 | 6393.08 | 6768.11 | 3704.29 | 4050.94 | 1323.37 | 4682.81 | 3405.52 | 5519.63 | 6158.72 | 4047.58 | 5001.38 |
| P00734 | Prothrombin | **0.76** | **0.73** | 23.24 | 24.56 | 17.67 | 27.61 | 25.90 | 58.68 | 31.64 | 0.00 | 0.00 | 47.19 | 0.00 | 58.81 | 0.00 | 0.00 | 0.00 |
| Q99729 | Heterogeneous nuclear ribonucleoprotein A/B | **1.11** | **0.73** | 123.52 | 44.03 | 137.06 | 75.94 | 89.35 | 173.59 | 130.91 | 155.20 | 68.54 | 125.68 | 37.93 | 55.87 | 202.92 | 196.02 | 203.91 |
| P21397 | Amine oxidase [flavin-containing] A | **1.26** | **0.74** | 52.17 | 38.22 | 65.91 | 80.03 | 65.29 | 0.00 | 104.65 | 52.78 | 38.15 | 129.65 | 187.44 | 0.00 | 0.00 | 0.00 | 78.38 |
| P39687 | Acidic leucine-rich nuclear phosphoprotein 32 family member A | **0.86** | **0.74** | 172.68 | 84.20 | 149.20 | 129.61 | 258.19 | 162.94 | 166.49 | 234.06 | 41.73 | 226.07 | 103.88 | 0.00 | 44.73 | 164.89 | 355.63 |
| P07585 | Decorin | **1.32** | **0.74** | 252.10 | 508.03 | 332.37 | 241.51 | 103.10 | 1157.38 | 0.00 | 0.00 | 0.00 | 215.59 | 274.59 | 660.46 | 564.19 | 279.38 | 0.00 |
| P08574 | Cytochrome c1, heme protein, mitochondrial | **0.76** | **0.74** | 65.21 | 101.81 | 49.83 | 39.53 | 0.00 | 0.00 | 232.18 | 93.86 | 0.00 | 71.48 | 86.84 | 0.00 | 0.00 | 61.19 | 79.46 |
| P45974 | Ubiquitin carboxyl-terminal hydrolase 5 | **0.87** | **0.74** | 36.69 | 22.06 | 32.08 | 22.14 | 37.46 | 0.00 | 56.06 | 51.56 | 38.38 | 50.27 | 22.40 | 27.34 | 0.00 | 29.31 | 63.19 |
| P22090 | 40S ribosomal protein S4, Y isoform 1 | **1.20** | **0.74** | 63.09 | 60.21 | 75.44 | 59.84 | 79.91 | 0.00 | 106.09 | 129.47 | 0.00 | 0.00 | 133.00 | 116.68 | 0.00 | 92.76 | 110.18 |
| P62879 | Guanine nucleotide-binding protein G(I)/G(S)/G(T) subunit beta-2 | **1.15** | **0.74** | 85.63 | 80.70 | 98.24 | 40.69 | 31.11 | 0.00 | 91.57 | 210.64 | 94.81 | 114.32 | 67.93 | 66.36 | 58.36 | 161.18 | 121.29 |
| Q9UL46 | Proteasome activator complex subunit 2 | **0.92** | **0.75** | 99.31 | 49.75 | 91.05 | 31.72 | 184.84 | 86.15 | 79.78 | 90.74 | 55.06 | 52.13 | 138.39 | 113.98 | 95.10 | 80.73 | 65.99 |
| Q14258 | E3 ubiquitin/ISG15 ligase TRIM25 | **1.27** | **0.75** | 11.73 | 17.53 | 14.90 | 13.94 | 39.27 | 0.00 | 0.00 | 19.38 | 0.00 | 36.81 | 17.24 | 0.00 | 0.00 | 14.05 | 21.28 |
| Q9UHD8 | Septin-9 | **0.87** | **0.75** | 73.26 | 48.56 | 63.77 | 45.44 | 66.93 | 0.00 | 126.09 | 107.59 | 65.70 | 92.04 | 115.08 | 0.00 | 25.21 | 98.38 | 51.94 |
| P13073 | Cytochrome c oxidase subunit 4 isoform 1, mitochondrial | **0.92** | **0.75** | 176.52 | 74.68 | 161.95 | 69.67 | 279.18 | 91.80 | 150.84 | 224.56 | 136.21 | 225.16 | 258.00 | 166.74 | 76.42 | 140.39 | 104.96 |
| P17066 | Heat shock 70 kDa protein 6 | **0.90** | **0.75** | 785.54 | 249.89 | 708.04 | 462.64 | 933.22 | 502.96 | 907.15 | 1050.28 | 534.07 | 809.17 | 326.38 | 331.38 | 284.12 | 1313.08 | 1184.10 |
| P78371 | T-complex protein 1 subunit beta | **0.87** | **0.75** | 58.07 | 37.66 | 50.29 | 39.31 | 66.32 | 0.00 | 72.10 | 102.37 | 49.57 | 110.98 | 47.60 | 25.42 | 0.00 | 39.62 | 78.14 |
| P01834 | Ig kappa chain C region | **0.82** | **0.75** | 2500.26 | 2852.84 | 2040.90 | 1683.31 | 294.97 | 7299.90 | 2863.43 | 747.99 | 1295.01 | 1245.48 | 740.65 | 1358.60 | 5303.91 | 2352.43 | 1244.32 |
| P20618 | Proteasome subunit beta type-1 | **1.11** | **0.75** | 44.39 | 13.31 | 49.19 | 30.07 | 42.67 | 30.35 | 57.11 | 58.97 | 32.83 | 59.92 | 61.19 | 0.00 | 29.29 | 58.76 | 85.98 |
| Q13409 | Cytoplasmic dynein 1 intermediate chain 2 | **0.84** | **0.75** | 39.90 | 36.80 | 33.54 | 27.82 | 61.55 | 0.00 | 62.90 | 75.07 | 0.00 | 60.73 | 36.48 | 0.00 | 0.00 | 42.28 | 61.75 |
| Q15661 | Tryptase alpha/beta-1 | **0.73** | **0.75** | 70.47 | 121.74 | 51.15 | 72.73 | 30.19 | 286.19 | 0.00 | 35.99 | 0.00 | 0.00 | 58.61 | 61.37 | 186.93 | 0.00 | 0.00 |
| Q9BYT9 | Anoctamin-3 | **0.80** | **0.75** | 314.25 | 330.92 | 251.51 | 305.73 | 85.57 | 391.95 | 840.74 | 253.00 | 0.00 | 0.00 | 0.00 | 0.00 | 671.18 | 569.61 | 268.28 |
| P54819 | Adenylate kinase 2, mitochondrial | **0.88** | **0.75** | 76.01 | 55.18 | 66.74 | 39.09 | 137.18 | 52.89 | 121.48 | 68.49 | 0.00 | 85.11 | 115.70 | 50.15 | 0.00 | 79.61 | 69.85 |
| Q9H223 | EH domain-containing protein 4 | **0.87** | **0.75** | 34.29 | 29.59 | 29.73 | 7.25 | 54.78 | 0.00 | 72.16 | 31.82 | 12.69 | 24.55 | 39.37 | 30.19 | 22.75 | 23.98 | 37.54 |
| P19652 | Alpha-1-acid glycoprotein 2 | **1.27** | **0.75** | 116.09 | 175.70 | 147.71 | 148.36 | 47.96 | 427.90 | 0.00 | 51.20 | 53.40 | 14.12 | 91.28 | 399.18 | 255.02 | 60.00 | 66.66 |
| Q9P1U1 | Actin-related protein 3B | **1.17** | **0.76** | 26.36 | 28.06 | 30.73 | 16.77 | 20.46 | 0.00 | 0.00 | 57.47 | 53.86 | 59.33 | 32.48 | 9.02 | 21.71 | 34.91 | 26.90 |
| P01617 | Ig kappa chain V-II region TEW | **0.77** | **0.76** | 217.47 | 351.53 | 167.61 | 140.46 | 0.00 | 834.86 | 156.98 | 0.00 | 95.52 | 113.68 | 165.14 | 177.56 | 424.01 | 125.26 | 0.00 |
| P01777 | Ig heavy chain V-III region TEI | **0.79** | **0.76** | 202.82 | 309.83 | 160.59 | 96.77 | 0.00 | 735.81 | 208.11 | 70.16 | 0.00 | 125.43 | 135.89 | 157.10 | 351.83 | 86.40 | 106.89 |
| Q6NVV1 | Putative 60S ribosomal protein L13a-like MGC87657 | **1.14** | **0.76** | 117.29 | 90.56 | 134.09 | 84.12 | 94.31 | 0.00 | 192.03 | 223.50 | 76.63 | 163.37 | 123.58 | 0.00 | 90.03 | 183.87 | 243.69 |
| P55795 | Heterogeneous nuclear ribonucleoprotein H2 | **1.13** | **0.76** | 162.13 | 135.06 | 183.56 | 65.31 | 96.04 | 0.00 | 315.79 | 288.64 | 110.18 | 237.46 | 150.90 | 112.90 | 112.64 | 236.99 | 250.47 |
| P07339 | Cathepsin D | **1.12** | **0.76** | 193.30 | 98.64 | 217.09 | 140.50 | 254.02 | 51.96 | 236.78 | 291.48 | 132.26 | 40.63 | 279.61 | 229.36 | 99.64 | 213.31 | 440.01 |
| P69891 | Hemoglobin subunit gamma-1 | **0.87** | **0.76** | 10348.82 | 5680.77 | 8975.58 | 8122.32 | 5457.66 | 4727.64 | 13382.26 | 9822.42 | 18354.11 | 2704.06 | 2067.49 | 4208.00 | 23297.30 | 12836.93 | 8739.71 |
| P31943 | Heterogeneous nuclear ribonucleoprotein H | **1.13** | **0.76** | 166.33 | 139.15 | 187.40 | 80.41 | 112.14 | 0.00 | 325.25 | 296.37 | 97.88 | 184.49 | 186.04 | 112.90 | 87.10 | 295.40 | 258.44 |
| Q9GZV4 | Eukaryotic translation initiation factor 5A-2 | **1.12** | **0.76** | 98.53 | 75.04 | 110.52 | 50.80 | 95.36 | 0.00 | 165.48 | 178.03 | 53.79 | 97.18 | 102.61 | 57.41 | 62.79 | 163.99 | 179.12 |
| P26368 | Splicing factor U2AF 65 kDa subunit | **1.19** | **0.76** | 30.54 | 26.77 | 36.39 | 33.57 | 14.74 | 0.00 | 60.14 | 57.20 | 20.64 | 19.41 | 63.55 | 0.00 | 0.00 | 66.44 | 68.91 |
| P62304 | Small nuclear ribonucleoprotein E | **1.18** | **0.76** | 46.64 | 38.94 | 55.04 | 48.11 | 29.63 | 0.00 | 93.99 | 79.53 | 30.03 | 27.44 | 38.17 | 0.00 | 38.14 | 98.45 | 128.02 |
| Q9H444 | Charged multivesicular body protein 4b | **1.18** | **0.76** | 39.12 | 37.33 | 45.98 | 35.68 | 76.37 | 0.00 | 0.00 | 47.63 | 71.59 | 66.14 | 70.99 | 0.00 | 0.00 | 71.58 | 67.15 |
| P28482 | Mitogen-activated protein kinase 1 | **0.82** | **0.76** | 25.33 | 20.03 | 20.75 | 27.20 | 19.46 | 0.00 | 55.12 | 30.55 | 21.51 | 31.38 | 68.50 | 0.00 | 0.00 | 0.00 | 24.64 |
| Q9UJU6 | Drebrin-like protein | **1.10** | **0.76** | 32.83 | 23.04 | 35.97 | 9.23 | 54.29 | 0.00 | 55.41 | 24.78 | 29.64 | 37.26 | 32.56 | 24.97 | 30.99 | 37.87 | 52.18 |
| P48047 | ATP synthase subunit O, mitochondrial | **0.89** | **0.77** | 120.61 | 89.04 | 107.48 | 51.18 | 191.98 | 0.00 | 197.05 | 161.13 | 52.87 | 157.66 | 81.13 | 78.38 | 47.87 | 98.79 | 181.02 |
| P49411 | Elongation factor Tu, mitochondrial | **1.11** | **0.77** | 104.50 | 85.16 | 116.42 | 41.14 | 90.75 | 0.00 | 210.47 | 168.05 | 53.23 | 111.97 | 136.94 | 102.55 | 52.81 | 116.31 | 177.91 |
| P0CG05 | Ig lambda-2 chain C regions | **1.19** | **0.77** | 867.80 | 1055.60 | 1035.75 | 769.47 | 273.95 | 2743.32 | 571.01 | 458.56 | 292.18 | 466.05 | 484.22 | 569.68 | 2296.47 | 1684.68 | 713.42 |
| P0CG06 | Ig lambda-3 chain C regions | **1.19** | **0.77** | 867.80 | 1055.60 | 1035.75 | 769.47 | 273.95 | 2743.32 | 571.01 | 458.56 | 292.18 | 466.05 | 484.22 | 569.68 | 2296.47 | 1684.68 | 713.42 |
| P28472 | Gamma-aminobutyric acid receptor subunit beta-3 | **1.18** | **0.77** | 119.26 | 113.33 | 140.95 | 121.72 | 147.43 | 0.00 | 227.10 | 221.77 | 0.00 | 228.83 | 119.99 | 0.00 | 0.00 | 209.23 | 287.67 |
| Q08AD1 | Calmodulin-regulated spectrin-associated protein 2 | **1.29** | **0.77** | 2284.58 | 4373.02 | 2938.96 | 2779.57 | 137.28 | 10092.87 | 750.60 | 368.38 | 73.75 | 3671.12 | 228.93 | 3829.00 | 7522.62 | 0.00 | 2382.07 |
| Q6ZU15 | Septin-14 | **0.82** | **0.77** | 45.22 | 46.24 | 37.29 | 41.11 | 0.00 | 41.41 | 90.34 | 94.33 | 0.00 | 74.10 | 0.00 | 82.14 | 0.00 | 0.00 | 67.48 |
| P08572 | Collagen alpha-2(IV) chain | **1.22** | **0.77** | 188.84 | 262.70 | 230.42 | 203.05 | 105.91 | 93.17 | 656.08 | 39.16 | 49.90 | 158.66 | 160.42 | 500.81 | 101.45 | 461.19 | 0.00 |
| P54652 | Heat shock-related 70 kDa protein 2 | **0.92** | **0.77** | 837.47 | 282.67 | 768.97 | 445.74 | 978.08 | 531.10 | 993.79 | 1143.44 | 540.94 | 783.09 | 434.89 | 420.16 | 358.09 | 1373.08 | 1244.54 |
| P60510 | Serine/threonine-protein phosphatase 4 catalytic subunit | **0.78** | **0.78** | 25.76 | 24.05 | 20.17 | 36.14 | 35.95 | 0.00 | 50.13 | 42.74 | 0.00 | 0.00 | 0.00 | 0.00 | 0.00 | 31.80 | 89.21 |
| Q15637 | Splicing factor 1 | **0.78** | **0.78** | 20.49 | 18.72 | 15.96 | 29.66 | 35.61 | 0.00 | 33.40 | 33.42 | 0.00 | 75.62 | 8.48 | 0.00 | 0.00 | 0.00 | 11.63 |
| P30154 | Serine/threonine-protein phosphatase 2A 65 kDa regulatory subunit A beta isoform | **0.86** | **0.78** | 33.35 | 30.86 | 28.71 | 21.52 | 56.91 | 0.00 | 61.97 | 47.87 | 0.00 | 19.69 | 50.91 | 0.00 | 27.43 | 56.80 | 17.42 |
| Q13724 | Mannosyl-oligosaccharide glucosidase | **1.16** | **0.78** | 19.62 | 21.93 | 22.85 | 14.69 | 13.44 | 0.00 | 48.81 | 35.88 | 0.00 | 16.52 | 28.38 | 0.00 | 23.15 | 24.20 | 44.86 |
| Q9NR48 | Histone-lysine N-methyltransferase ASH1L | **0.92** | **0.78** | 427.41 | 167.25 | 395.02 | 195.70 | 486.84 | 620.30 | 453.28 | 414.38 | 162.24 | 476.38 | 0.00 | 460.80 | 487.36 | 428.69 | 516.89 |
| O14950 | Myosin regulatory light chain 12B | **0.90** | **0.78** | 432.24 | 327.89 | 390.46 | 121.31 | 985.95 | 459.88 | 312.19 | 232.15 | 171.03 | 328.83 | 362.54 | 589.01 | 448.90 | 384.64 | 228.82 |
| P19105 | Myosin regulatory light chain 12A | **0.90** | **0.78** | 432.24 | 327.89 | 390.46 | 121.31 | 985.95 | 459.88 | 312.19 | 232.15 | 171.03 | 328.83 | 362.54 | 589.01 | 448.90 | 384.64 | 228.82 |
| P23528 | Cofilin-1 | **0.92** | **0.78** | 767.36 | 242.96 | 709.27 | 385.97 | 905.44 | 519.57 | 917.09 | 1004.67 | 490.04 | 696.64 | 462.84 | 242.89 | 563.03 | 964.56 | 1325.63 |
| P22102 | Trifunctional purine biosynthetic protein adenosine-3 | **1.19** | **0.78** | 11.83 | 13.51 | 14.03 | 11.70 | 6.49 | 0.00 | 25.53 | 27.13 | 0.00 | 23.82 | 0.00 | 21.79 | 0.00 | 25.60 | 12.94 |
| P51153 | Ras-related protein Rab-13 | **1.08** | **0.78** | 321.25 | 114.68 | 348.34 | 180.08 | 371.11 | 169.55 | 334.47 | 472.82 | 258.29 | 582.41 | 133.13 | 328.58 | 209.05 | 293.13 | 543.77 |
| P46778 | 60S ribosomal protein L21 | **1.15** | **0.78** | 117.00 | 75.60 | 134.82 | 120.01 | 126.99 | 0.00 | 196.04 | 165.88 | 96.12 | 176.41 | 316.00 | 0.00 | 0.00 | 159.93 | 156.60 |
| P02743 | Serum amyloid P-component | **1.33** | **0.78** | 129.48 | 262.78 | 172.14 | 234.13 | 49.39 | 597.99 | 0.00 | 0.00 | 0.00 | 40.89 | 112.20 | 611.75 | 251.18 | 16.80 | 0.00 |
| P07864 | L-lactate dehydrogenase C chain | **0.93** | **0.78** | 1362.48 | 715.49 | 1260.94 | 358.66 | 1783.49 | 502.12 | 1902.61 | 1957.61 | 666.57 | 813.23 | 1294.27 | 1684.45 | 980.34 | 1125.89 | 1667.48 |
| Q6ZMR3 | L-lactate dehydrogenase A-like 6A | **0.93** | **0.78** | 1362.48 | 715.49 | 1260.94 | 358.66 | 1783.49 | 502.12 | 1902.61 | 1957.61 | 666.57 | 813.23 | 1294.27 | 1684.45 | 980.34 | 1125.89 | 1667.48 |
| P13010 | X-ray repair cross-complementing protein 5 | **0.88** | **0.78** | 85.62 | 54.95 | 75.39 | 63.46 | 40.31 | 39.87 | 165.45 | 118.58 | 63.91 | 82.81 | 72.85 | 34.13 | 0.00 | 74.55 | 188.03 |
| Q14108 | Lysosome membrane protein 2 | **1.13** | **0.79** | 34.70 | 32.17 | 39.37 | 22.79 | 0.00 | 66.95 | 0.00 | 53.19 | 53.37 | 39.02 | 70.34 | 0.00 | 38.62 | 39.59 | 48.63 |
| P00390 | Glutathione reductase, mitochondrial | **0.93** | **0.79** | 49.59 | 29.08 | 46.04 | 11.56 | 59.53 | 0.00 | 75.96 | 60.79 | 51.70 | 59.89 | 41.38 | 44.00 | 52.65 | 51.67 | 26.67 |
| P27695 | DNA-(apurinic or apyrimidinic site) lyase | **1.19** | **0.79** | 43.78 | 42.50 | 52.07 | 54.54 | 59.07 | 0.00 | 96.41 | 63.44 | 0.00 | 49.68 | 45.51 | 0.00 | 0.00 | 69.86 | 147.39 |
| Q96TA1 | Niban-like protein 1 | **1.21** | **0.79** | 24.12 | 15.93 | 29.09 | 37.46 | 19.69 | 0.00 | 37.28 | 39.63 | 23.98 | 0.00 | 47.83 | 0.00 | 0.00 | 33.50 | 93.19 |
| P84103 | Serine/arginine-rich splicing factor 3 | **1.14** | **0.79** | 84.50 | 64.97 | 96.51 | 78.33 | 88.85 | 0.00 | 121.49 | 167.01 | 45.15 | 88.63 | 80.22 | 0.00 | 35.59 | 166.80 | 207.81 |
| Q07955 | Serine/arginine-rich splicing factor 1 | **1.14** | **0.79** | 53.11 | 47.63 | 60.50 | 42.19 | 39.50 | 0.00 | 109.69 | 95.68 | 20.66 | 63.60 | 43.61 | 39.79 | 0.00 | 109.03 | 106.97 |
| Q9BQS8 | FYVE and coiled-coil domain-containing protein 1 | **0.74** | **0.79** | 337.67 | 419.48 | 249.64 | 611.49 | 573.20 | 0.00 | 959.84 | 155.32 | 0.00 | 1497.85 | 0.00 | 0.00 | 0.00 | 0.00 | 0.00 |
| P50502 | Hsc70-interacting protein | **0.93** | **0.80** | 162.95 | 86.99 | 151.73 | 51.65 | 313.79 | 139.54 | 91.31 | 146.91 | 123.22 | 239.96 | 133.18 | 91.23 | 153.61 | 118.93 | 173.45 |
| P35241 | Radixin | **0.93** | **0.80** | 297.61 | 145.03 | 276.96 | 115.68 | 396.00 | 117.81 | 183.45 | 465.98 | 324.82 | 132.10 | 238.91 | 266.01 | 198.92 | 387.38 | 438.45 |
| Q3SY05 | Putative uncharacterized protein encoded by LINC00303 | **0.86** | **0.80** | 221.12 | 207.52 | 190.26 | 185.09 | 293.99 | 0.00 | 427.42 | 384.19 | 0.00 | 374.26 | 129.25 | 0.00 | 0.00 | 200.39 | 437.66 |
| O95831 | Apoptosis-inducing factor 1, mitochondrial | **1.11** | **0.80** | 21.50 | 15.72 | 23.87 | 14.46 | 36.94 | 0.00 | 31.08 | 29.50 | 9.98 | 21.29 | 42.27 | 0.00 | 18.31 | 32.62 | 28.71 |
| Q05639 | Elongation factor 1-alpha 2 | **0.94** | **0.80** | 1661.59 | 729.45 | 1562.26 | 539.78 | 1440.81 | 729.00 | 1692.72 | 2760.89 | 1684.52 | 2449.94 | 835.50 | 1362.32 | 1325.86 | 1758.65 | 1641.27 |
| P26583 | High mobility group protein B2 | **1.12** | **0.80** | 143.63 | 53.39 | 161.40 | 144.48 | 155.07 | 124.76 | 224.29 | 136.38 | 77.63 | 120.73 | 124.82 | 48.78 | 22.98 | 237.06 | 414.02 |
| P13645 | Keratin, type I cytoskeletal 10 | **0.90** | **0.80** | 502.91 | 343.16 | 451.93 | 312.19 | 397.66 | 443.73 | 161.39 | 430.43 | 1081.32 | 934.17 | 302.00 | 731.20 | 125.72 | 249.45 | 369.01 |
| Q13148 | TAR DNA-binding protein 43 | **1.11** | **0.80** | 35.89 | 29.44 | 39.84 | 21.72 | 19.44 | 0.00 | 73.59 | 56.97 | 29.42 | 53.95 | 43.89 | 0.00 | 30.79 | 53.83 | 56.56 |
| P09104 | Gamma-enolase | **1.08** | **0.80** | 563.78 | 325.15 | 609.04 | 261.45 | 850.25 | 152.10 | 453.11 | 937.92 | 425.52 | 522.36 | 458.59 | 594.96 | 375.72 | 1115.51 | 587.08 |
| Q6IS14 | Eukaryotic translation initiation factor 5A-1-like | **0.93** | **0.80** | 120.25 | 53.68 | 112.10 | 51.48 | 95.36 | 101.08 | 172.99 | 178.03 | 53.79 | 111.56 | 102.61 | 52.55 | 62.79 | 163.99 | 179.12 |
| P18859 | ATP synthase-coupling factor 6, mitochondrial | **0.85** | **0.80** | 113.04 | 140.68 | 95.68 | 82.28 | 82.74 | 81.24 | 43.91 | 357.33 | 0.00 | 149.29 | 0.00 | 0.00 | 109.49 | 206.63 | 108.65 |
| P39060 | Collagen alpha-1(XVIII) chain | **0.86** | **0.81** | 63.90 | 74.84 | 54.92 | 43.46 | 115.43 | 0.00 | 167.88 | 36.18 | 0.00 | 50.35 | 21.81 | 70.89 | 0.00 | 125.80 | 60.66 |
| Q08211 | ATP-dependent RNA helicase A | **1.11** | **0.81** | 66.95 | 54.31 | 74.06 | 40.41 | 41.55 | 0.00 | 111.38 | 133.02 | 48.78 | 89.97 | 53.11 | 34.55 | 34.92 | 94.95 | 136.87 |
| O14773 | Tripeptidyl-peptidase 1 | **1.10** | **0.81** | 69.85 | 41.25 | 76.74 | 48.85 | 80.33 | 110.24 | 81.13 | 77.56 | 0.00 | 0.00 | 124.41 | 123.43 | 78.70 | 93.39 | 40.54 |
| P54707 | Potassium-transporting ATPase alpha chain 2 | **0.87** | **0.81** | 47.75 | 46.14 | 41.75 | 35.42 | 69.88 | 0.00 | 104.11 | 64.74 | 0.00 | 78.85 | 65.78 | 0.00 | 35.85 | 0.00 | 70.02 |
| Q92600 | Cell differentiation protein RCD1 homolog | **0.82** | **0.81** | 74.47 | 71.27 | 61.39 | 99.50 | 143.17 | 0.00 | 89.20 | 139.97 | 0.00 | 137.94 | 0.00 | 0.00 | 0.00 | 230.42 | 0.00 |
| P08107 | Heat shock 70 kDa protein 1A/1B | **0.93** | **0.81** | 792.65 | 247.13 | 739.40 | 461.19 | 939.57 | 553.55 | 916.67 | 1050.28 | 503.19 | 938.42 | 375.48 | 325.30 | 300.00 | 1313.08 | 1184.10 |
| P01859 | Ig gamma-2 chain C region | **0.86** | **0.81** | 1696.92 | 2144.56 | 1462.88 | 944.10 | 246.74 | 5440.77 | 1512.99 | 547.82 | 736.28 | 891.30 | 805.07 | 1282.38 | 3286.90 | 1610.70 | 900.96 |
| Q8IZP2 | Putative protein FAM10A4 | **0.94** | **0.82** | 161.64 | 84.15 | 151.73 | 51.65 | 307.23 | 139.54 | 91.31 | 146.91 | 123.22 | 239.96 | 133.18 | 91.23 | 153.61 | 118.93 | 173.45 |
| P07195 | L-lactate dehydrogenase B chain | **0.94** | **0.82** | 821.55 | 443.40 | 768.81 | 200.82 | 960.07 | 290.13 | 1268.67 | 1170.89 | 417.98 | 757.67 | 757.23 | 850.81 | 475.20 | 684.75 | 1087.23 |
| P04004 | Vitronectin | **0.88** | **0.82** | 84.63 | 78.01 | 74.16 | 66.43 | 42.36 | 223.46 | 61.99 | 51.87 | 43.47 | 43.25 | 36.90 | 191.97 | 91.00 | 81.85 | 0.00 |
| P18077 | 60S ribosomal protein L35a | **0.86** | **0.82** | 49.96 | 47.99 | 42.90 | 49.03 | 60.88 | 0.00 | 86.06 | 102.85 | 0.00 | 74.20 | 0.00 | 0.00 | 0.00 | 71.92 | 111.28 |
| P18754 | Regulator of chromosome condensation | **0.86** | **0.82** | 16.48 | 15.35 | 14.13 | 16.77 | 27.62 | 0.00 | 31.70 | 23.08 | 0.00 | 39.92 | 0.00 | 0.00 | 0.00 | 23.74 | 21.14 |
| Q92841 | Probable ATP-dependent RNA helicase DDX17 | **1.09** | **0.82** | 101.07 | 57.23 | 110.26 | 67.88 | 68.26 | 47.44 | 158.75 | 167.46 | 63.46 | 149.74 | 59.39 | 42.51 | 47.21 | 170.80 | 191.89 |
| Q00796 | Sorbitol dehydrogenase | **1.22** | **0.82** | 33.46 | 40.70 | 40.69 | 56.37 | 0.00 | 0.00 | 17.89 | 55.48 | 93.95 | 143.20 | 0.00 | 0.00 | 0.00 | 60.97 | 39.95 |
| Q9H299 | SH3 domain-binding glutamic acid-rich-like protein 3 | **1.13** | **0.82** | 34.30 | 32.27 | 38.86 | 31.06 | 49.17 | 0.00 | 69.75 | 52.58 | 0.00 | 0.00 | 47.46 | 0.00 | 52.66 | 64.59 | 68.48 |
| O95470 | Sphingosine-1-phosphate lyase 1 | **1.17** | **0.82** | 37.19 | 35.20 | 43.44 | 49.20 | 50.49 | 0.00 | 76.30 | 59.17 | 0.00 | 85.40 | 107.35 | 0.00 | 0.00 | 67.87 | 0.00 |
| Q9H3N1 | Thioredoxin-related transmembrane protein 1 | **0.84** | **0.82** | 33.95 | 36.06 | 28.65 | 37.54 | 42.53 | 0.00 | 86.67 | 40.57 | 0.00 | 81.60 | 0.00 | 0.00 | 20.30 | 0.00 | 69.99 |
| Q05707 | Collagen alpha-1(XIV) chain | **0.90** | **0.82** | 888.93 | 408.02 | 803.18 | 714.72 | 375.17 | 932.69 | 990.07 | 1474.50 | 672.24 | 412.36 | 630.78 | 635.96 | 242.51 | 675.46 | 2221.99 |
| Q53EL6 | Programmed cell death protein 4 | **1.23** | **0.82** | 11.72 | 10.96 | 14.42 | 23.34 | 17.33 | 0.00 | 17.92 | 23.36 | 0.00 | 53.94 | 0.00 | 0.00 | 0.00 | 0.00 | 32.57 |
| P62750 | 60S ribosomal protein L23a | **1.08** | **0.82** | 112.96 | 62.44 | 121.52 | 57.79 | 111.92 | 92.24 | 181.61 | 157.88 | 21.13 | 151.91 | 88.75 | 120.16 | 30.00 | 139.25 | 199.03 |
| P42167 | Lamina-associated polypeptide 2, isoforms beta/gamma | **0.87** | **0.82** | 58.13 | 34.84 | 50.68 | 63.09 | 89.07 | 0.00 | 69.54 | 77.49 | 54.55 | 124.89 | 0.00 | 0.00 | 0.00 | 46.81 | 132.37 |
| Q9NZN4 | EH domain-containing protein 2 | **1.20** | **0.82** | 37.63 | 40.98 | 45.09 | 60.79 | 41.83 | 99.08 | 47.21 | 0.00 | 0.00 | 26.30 | 163.46 | 40.20 | 0.00 | 40.55 | 0.00 |
| Q5HYI8 | Rab-like protein 3 | **1.17** | **0.82** | 87.94 | 80.46 | 103.32 | 127.52 | 147.70 | 0.00 | 0.00 | 153.58 | 138.44 | 142.65 | 164.08 | 0.00 | 0.00 | 0.00 | 313.17 |
| Q3ZCM7 | Tubulin beta-8 chain | **1.09** | **0.82** | 486.83 | 304.18 | 529.48 | 302.62 | 402.10 | 197.03 | 600.40 | 957.89 | 276.75 | 403.55 | 300.70 | 233.20 | 442.87 | 1004.05 | 792.51 |
| P07108 | Acyl-CoA-binding protein | **0.83** | **0.82** | 80.91 | 97.85 | 66.83 | 103.60 | 229.98 | 49.17 | 125.38 | 0.00 | 0.00 | 0.00 | 206.59 | 0.00 | 0.00 | 0.00 | 194.38 |
| O75964 | ATP synthase subunit g, mitochondrial | **0.88** | **0.82** | 76.10 | 44.92 | 67.23 | 75.18 | 85.80 | 0.00 | 119.26 | 92.91 | 82.52 | 195.32 | 66.33 | 0.00 | 0.00 | 33.57 | 108.16 |
| P09651 | Heterogeneous nuclear ribonucleoprotein A1 | **1.09** | **0.82** | 205.58 | 134.36 | 224.32 | 134.89 | 131.01 | 56.85 | 321.92 | 371.48 | 146.66 | 273.51 | 105.39 | 106.31 | 107.26 | 389.66 | 363.78 |
| P49368 | T-complex protein 1 subunit gamma | **0.95** | **0.82** | 115.67 | 43.31 | 109.93 | 39.27 | 156.48 | 62.39 | 116.95 | 159.57 | 82.95 | 166.93 | 108.97 | 105.56 | 52.41 | 89.22 | 136.48 |
| P56192 | Methionine--tRNA ligase, cytoplasmic | **0.86** | **0.82** | 17.18 | 17.58 | 14.71 | 17.83 | 19.69 | 0.00 | 24.94 | 41.25 | 0.00 | 0.00 | 16.94 | 0.00 | 0.00 | 30.33 | 41.01 |
| P55084 | Trifunctional enzyme subunit beta, mitochondrial | **0.93** | **0.82** | 83.51 | 55.58 | 77.35 | 33.26 | 131.71 | 0.00 | 128.18 | 101.40 | 56.28 | 65.72 | 119.83 | 73.02 | 31.63 | 61.46 | 112.44 |
| Q07666 | KH domain-containing, RNA-binding, signal transduction-associated protein 1 | **0.91** | **0.82** | 105.37 | 64.06 | 96.11 | 69.15 | 169.08 | 0.00 | 101.66 | 117.89 | 138.25 | 106.41 | 171.30 | 0.00 | 32.25 | 165.44 | 101.26 |
| P0CF74 | Ig lambda-6 chain C region | **1.14** | **0.83** | 908.19 | 1045.21 | 1032.09 | 762.28 | 273.95 | 2743.32 | 772.94 | 458.56 | 292.18 | 466.05 | 484.22 | 569.68 | 2274.49 | 1684.68 | 713.42 |
| P02649 | Apolipoprotein E | **1.19** | **0.83** | 50.05 | 90.02 | 59.74 | 50.32 | 42.59 | 207.66 | 0.00 | 0.00 | 0.00 | 54.35 | 88.25 | 103.55 | 112.31 | 0.00 | 0.00 |
| Q92598 | Heat shock protein 105 kDa | **1.15** | **0.83** | 33.64 | 30.99 | 38.64 | 40.49 | 49.25 | 0.00 | 59.95 | 59.00 | 0.00 | 81.43 | 25.56 | 0.00 | 0.00 | 30.21 | 94.66 |
| P60763 | Ras-related C3 botulinum toxin substrate 3 | **0.95** | **0.83** | 143.90 | 31.64 | 136.75 | 63.89 | 162.04 | 124.43 | 165.58 | 169.92 | 97.55 | 132.62 | 205.43 | 34.80 | 92.67 | 180.43 | 174.58 |
| P01860 | Ig gamma-3 chain C region | **0.86** | **0.83** | 2321.58 | 3022.77 | 2006.27 | 1547.38 | 335.26 | 7643.50 | 1823.22 | 844.39 | 961.54 | 1153.70 | 842.57 | 1495.14 | 4921.66 | 2538.51 | 1086.01 |
| P46060 | Ran GTPase-activating protein 1 | **1.25** | **0.83** | 13.56 | 13.00 | 17.01 | 32.27 | 22.86 | 0.00 | 28.08 | 16.84 | 0.00 | 21.57 | 0.00 | 0.00 | 0.00 | 0.00 | 80.48 |
| O00567 | Nucleolar protein 56 | **1.20** | **0.83** | 17.84 | 17.65 | 21.42 | 32.35 | 18.81 | 0.00 | 37.07 | 33.30 | 0.00 | 3.35 | 10.13 | 0.00 | 0.00 | 32.46 | 82.56 |
| Q92688 | Acidic leucine-rich nuclear phosphoprotein 32 family member B | **0.91** | **0.83** | 121.31 | 48.81 | 109.83 | 107.97 | 155.75 | 124.79 | 130.56 | 157.46 | 38.01 | 96.19 | 88.64 | 0.00 | 37.15 | 127.10 | 309.86 |
| O43399 | Tumor protein D54 | **1.09** | **0.83** | 46.53 | 38.08 | 50.82 | 27.69 | 97.25 | 64.27 | 19.62 | 0.00 | 51.53 | 39.63 | 100.90 | 41.25 | 25.10 | 34.18 | 63.87 |
| Q14525 | Keratin, type I cuticular Ha3-II | **0.93** | **0.83** | 809.17 | 313.79 | 751.68 | 517.64 | 1084.40 | 1001.08 | 294.41 | 920.06 | 745.91 | 1203.78 | 667.08 | 1532.46 | 169.04 | 566.70 | 371.00 |
| Q15323 | Keratin, type I cuticular Ha1 | **0.93** | **0.83** | 809.17 | 313.79 | 751.68 | 517.64 | 1084.40 | 1001.08 | 294.41 | 920.06 | 745.91 | 1203.78 | 667.08 | 1532.46 | 169.04 | 566.70 | 371.00 |
| P01871 | Ig mu chain C region | **1.14** | **0.84** | 145.97 | 114.64 | 166.28 | 183.79 | 0.00 | 301.67 | 143.56 | 201.40 | 83.21 | 158.81 | 78.84 | 101.13 | 533.11 | 40.48 | 85.29 |
| P62306 | Small nuclear ribonucleoprotein F | **1.13** | **0.84** | 35.26 | 22.00 | 39.70 | 44.39 | 54.16 | 0.00 | 48.76 | 45.31 | 28.08 | 66.18 | 0.00 | 0.00 | 0.00 | 77.92 | 94.10 |
| P62333 | 26S protease regulatory subunit 10B | **1.13** | **0.84** | 18.99 | 19.67 | 21.54 | 20.22 | 23.20 | 0.00 | 24.94 | 46.79 | 0.00 | 17.93 | 0.00 | 0.00 | 23.43 | 37.03 | 50.87 |
| P13667 | Protein disulfide-isomerase A4 | **1.03** | **0.84** | 149.97 | 40.07 | 154.25 | 27.21 | 139.82 | 127.39 | 154.40 | 216.01 | 112.19 | 163.18 | 129.30 | 114.92 | 155.78 | 180.86 | 181.48 |
| P61970 | Nuclear transport factor 2 | **1.11** | **0.84** | 36.29 | 33.14 | 40.12 | 27.15 | 59.00 | 0.00 | 60.94 | 61.52 | 0.00 | 71.81 | 38.11 | 40.83 | 22.41 | 0.00 | 67.55 |
| P19525 | Interferon-induced, double-stranded RNA-activated protein kinase | **0.85** | **0.84** | 8.18 | 8.77 | 6.92 | 10.76 | 0.00 | 0.00 | 14.30 | 19.79 | 6.81 | 0.00 | 19.22 | 0.00 | 0.00 | 22.29 | 0.00 |
| P40227 | T-complex protein 1 subunit zeta | **0.92** | **0.84** | 65.87 | 42.31 | 60.79 | 37.99 | 83.40 | 0.00 | 88.36 | 107.74 | 49.86 | 99.19 | 71.02 | 35.90 | 0.00 | 61.49 | 97.14 |
| P06733 | Alpha-enolase | **1.06** | **0.84** | 1053.01 | 518.32 | 1111.10 | 404.09 | 1364.29 | 464.80 | 931.84 | 1770.63 | 733.50 | 915.47 | 1012.76 | 763.55 | 984.09 | 1905.40 | 1085.31 |
| P49006 | MARCKS-related protein | **1.15** | **0.84** | 66.11 | 65.75 | 75.73 | 83.68 | 0.00 | 0.00 | 114.44 | 144.77 | 71.34 | 153.20 | 0.00 | 0.00 | 0.00 | 167.88 | 133.31 |
| P00338 | L-lactate dehydrogenase A chain | **1.05** | **0.84** | 672.45 | 328.92 | 704.75 | 177.24 | 1011.08 | 316.71 | 715.98 | 966.89 | 351.61 | 429.63 | 620.82 | 938.19 | 690.73 | 704.26 | 844.90 |
| P62314 | Small nuclear ribonucleoprotein Sm D1 | **1.13** | **0.84** | 66.32 | 68.36 | 75.13 | 72.34 | 85.90 | 0.00 | 83.36 | 162.35 | 0.00 | 82.79 | 56.85 | 0.00 | 0.00 | 183.32 | 127.83 |
| A6NEC2 | Puromycin-sensitive aminopeptidase-like protein | **1.08** | **0.84** | 26.11 | 16.31 | 28.10 | 15.75 | 31.39 | 0.00 | 31.01 | 44.16 | 23.97 | 39.83 | 33.73 | 20.73 | 0.00 | 42.79 | 31.54 |
| Q9H8H3 | Methyltransferase-like protein 7A | **0.89** | **0.84** | 77.07 | 57.64 | 68.55 | 76.36 | 131.29 | 0.00 | 106.08 | 116.10 | 31.86 | 206.16 | 51.17 | 0.00 | 0.00 | 63.28 | 90.72 |
| Q9NP72 | Ras-related protein Rab-18 | **1.19** | **0.85** | 8.43 | 8.66 | 10.03 | 15.75 | 13.10 | 0.00 | 8.96 | 20.08 | 0.00 | 0.00 | 25.93 | 0.00 | 0.00 | 0.00 | 34.23 |
| Q16629 | Serine/arginine-rich splicing factor 7 | **1.11** | **0.85** | 93.03 | 81.01 | 103.05 | 83.94 | 68.24 | 0.00 | 176.24 | 179.10 | 41.58 | 88.74 | 92.58 | 0.00 | 35.59 | 219.01 | 182.41 |
| A0M8Q6 | Ig lambda-7 chain C region | **1.08** | **0.85** | 529.16 | 334.01 | 571.75 | 366.24 | 170.58 | 812.97 | 924.55 | 247.62 | 490.09 | 197.01 | 339.67 | 343.78 | 1086.80 | 964.01 | 499.25 |
| P09874 | Poly [ADP-ribose] polymerase 1 | **1.15** | **0.85** | 23.80 | 33.13 | 27.27 | 25.05 | 0.00 | 0.00 | 51.17 | 67.85 | 0.00 | 40.66 | 19.36 | 0.00 | 0.00 | 61.93 | 41.70 |
| P60953 | Cell division control protein 42 homolog | **0.96** | **0.85** | 135.10 | 52.19 | 129.49 | 41.38 | 185.66 | 111.83 | 98.08 | 196.43 | 83.48 | 136.77 | 133.25 | 64.60 | 101.81 | 160.37 | 180.13 |
| P27338 | Amine oxidase [flavin-containing] B | **1.15** | **0.85** | 14.76 | 13.65 | 16.99 | 21.98 | 13.60 | 0.00 | 10.70 | 12.31 | 37.21 | 51.41 | 35.92 | 0.00 | 0.00 | 0.00 | 14.64 |
| O15523 | ATP-dependent RNA helicase DDX3Y | **1.09** | **0.85** | 37.98 | 32.15 | 41.32 | 24.06 | 23.87 | 0.00 | 69.21 | 73.90 | 22.94 | 21.95 | 39.51 | 31.49 | 14.27 | 69.17 | 71.53 |
| P62714 | Serine/threonine-protein phosphatase 2A catalytic subunit beta isoform | **0.91** | **0.85** | 38.62 | 25.45 | 35.25 | 30.19 | 37.37 | 0.00 | 57.89 | 64.95 | 32.88 | 37.43 | 10.17 | 0.00 | 29.61 | 49.54 | 84.71 |
| P67775 | Serine/threonine-protein phosphatase 2A catalytic subunit alpha isoform | **0.91** | **0.85** | 38.62 | 25.45 | 35.25 | 30.19 | 37.37 | 0.00 | 57.89 | 64.95 | 32.88 | 37.43 | 10.17 | 0.00 | 29.61 | 49.54 | 84.71 |
| Q58FG0 | Putative heat shock protein HSP 90-alpha A5 | **0.97** | **0.85** | 340.02 | 98.59 | 328.48 | 94.65 | 306.80 | 185.26 | 414.90 | 426.72 | 366.44 | 307.28 | 517.60 | 294.34 | 301.49 | 252.91 | 297.28 |
| Q09666 | Neuroblast differentiation-associated protein AHNAK | **0.94** | **0.85** | 417.22 | 191.05 | 393.45 | 204.98 | 572.23 | 662.98 | 289.46 | 217.50 | 343.93 | 764.37 | 250.95 | 335.42 | 196.28 | 337.55 | 476.09 |
| O14556 | Glyceraldehyde-3-phosphate dehydrogenase, testis-specific | **1.05** | **0.85** | 1232.12 | 548.33 | 1289.97 | 434.79 | 1625.89 | 538.99 | 956.53 | 1923.30 | 1115.87 | 1312.63 | 700.36 | 2027.38 | 1403.56 | 1151.91 | 1143.99 |
| Q5JXB2 | Putative ubiquitin-conjugating enzyme E2 N-like | **0.97** | **0.85** | 205.95 | 47.45 | 199.43 | 60.19 | 275.76 | 225.21 | 187.34 | 149.13 | 192.29 | 297.72 | 226.74 | 217.03 | 155.48 | 133.35 | 166.25 |
| P62273 | 40S ribosomal protein S29 | **1.14** | **0.85** | 15.90 | 16.32 | 18.18 | 21.43 | 16.22 | 0.00 | 26.02 | 37.28 | 0.00 | 25.94 | 0.00 | 0.00 | 0.00 | 32.87 | 50.26 |
| Q15185 | Prostaglandin E synthase 3 | **1.13** | **0.85** | 67.66 | 45.90 | 76.27 | 88.71 | 91.39 | 0.00 | 119.55 | 80.44 | 46.90 | 34.07 | 65.15 | 31.59 | 0.00 | 78.42 | 248.40 |
| P32969 | 60S ribosomal protein L9 | **1.07** | **0.85** | 62.80 | 34.55 | 67.49 | 43.65 | 51.91 | 26.36 | 82.28 | 112.51 | 40.94 | 55.23 | 117.82 | 0.00 | 42.38 | 85.54 | 103.97 |
| Q13263 | Transcription intermediary factor 1-beta | **1.09** | **0.85** | 80.48 | 40.90 | 87.58 | 72.70 | 72.12 | 31.36 | 144.25 | 85.15 | 69.54 | 112.79 | 79.88 | 45.49 | 24.76 | 41.31 | 221.26 |
| P62834 | Ras-related protein Rap-1A | **1.09** | **0.85** | 82.28 | 75.35 | 89.74 | 45.49 | 137.74 | 0.00 | 128.35 | 145.32 | 0.00 | 28.02 | 90.46 | 158.36 | 118.12 | 85.45 | 58.06 |
| P63151 | Serine/threonine-protein phosphatase 2A 55 kDa regulatory subunit B alpha isoform | **0.86** | **0.85** | 764.15 | 1165.80 | 653.89 | 723.95 | 2660.12 | 0.00 | 1130.89 | 29.76 | 0.00 | 508.20 | 1208.08 | 0.00 | 1822.11 | 0.00 | 384.98 |
| P07814 | Bifunctional glutamate/proline--tRNA ligase | **0.91** | **0.85** | 24.39 | 19.81 | 22.09 | 19.69 | 20.14 | 0.00 | 30.95 | 53.74 | 17.13 | 36.60 | 17.95 | 0.00 | 0.00 | 29.98 | 48.02 |
| P00739 | Haptoglobin-related protein | **1.08** | **0.85** | 333.60 | 275.48 | 358.79 | 156.28 | 139.08 | 820.58 | 234.65 | 242.15 | 231.55 | 409.27 | 212.78 | 623.55 | 412.08 | 230.79 | 264.27 |
| P63000 | Ras-related C3 botulinum toxin substrate 1 | **0.96** | **0.85** | 143.90 | 31.64 | 137.85 | 64.72 | 162.04 | 124.43 | 165.58 | 169.92 | 97.55 | 132.62 | 205.43 | 34.80 | 92.67 | 180.43 | 181.15 |
| P50991 | T-complex protein 1 subunit delta | **0.96** | **0.85** | 92.14 | 35.30 | 88.24 | 33.04 | 109.57 | 46.21 | 115.02 | 126.83 | 63.09 | 139.44 | 84.04 | 72.07 | 44.64 | 77.67 | 111.56 |
| O95715 | C-X-C motif chemokine 14 | **1.13** | **0.86** | 45.19 | 43.15 | 51.02 | 56.54 | 56.10 | 0.00 | 78.36 | 91.52 | 0.00 | 115.09 | 102.92 | 0.00 | 0.00 | 0.00 | 88.10 |
| P55957 | BH3-interacting domain death agonist | **0.90** | **0.86** | 26.92 | 28.28 | 24.27 | 18.90 | 66.22 | 0.00 | 0.00 | 41.25 | 27.11 | 43.44 | 31.44 | 13.81 | 0.00 | 46.25 | 10.68 |
| P47914 | 60S ribosomal protein L29 | **1.12** | **0.86** | 160.44 | 154.17 | 179.49 | 181.94 | 74.70 | 0.00 | 351.90 | 296.95 | 78.63 | 279.79 | 191.06 | 0.00 | 0.00 | 129.31 | 476.77 |
| O75526 | RNA-binding motif protein, X-linked-like-2 | **1.11** | **0.86** | 58.54 | 39.06 | 65.12 | 70.94 | 52.21 | 0.00 | 97.31 | 91.49 | 51.68 | 38.31 | 66.83 | 0.00 | 0.00 | 98.70 | 186.90 |
| O75390 | Citrate synthase, mitochondrial | **1.07** | **0.86** | 90.77 | 63.36 | 96.79 | 47.64 | 90.64 | 0.00 | 125.95 | 168.77 | 68.49 | 93.35 | 104.47 | 71.55 | 34.16 | 98.47 | 178.73 |
| Q2M2I5 | Keratin, type I cytoskeletal 24 | **1.08** | **0.86** | 558.90 | 320.24 | 605.64 | 496.55 | 1084.40 | 575.11 | 224.68 | 471.90 | 438.43 | 714.76 | 405.23 | 1532.46 | 169.04 | 566.70 | 245.63 |
| Q13630 | GDP-L-fucose synthase | **1.10** | **0.86** | 17.94 | 17.40 | 19.78 | 16.68 | 34.99 | 0.00 | 20.29 | 34.40 | 0.00 | 0.00 | 32.90 | 30.11 | 17.84 | 0.00 | 37.84 |
| Q15102 | Platelet-activating factor acetylhydrolase IB subunit gamma | **1.14** | **0.86** | 200.32 | 196.40 | 229.27 | 311.53 | 122.11 | 0.00 | 294.22 | 495.36 | 89.93 | 774.14 | 83.32 | 23.68 | 0.00 | 436.43 | 58.03 |
| Q8N7X1 | RNA-binding motif protein, X-linked-like-3 | **0.90** | **0.86** | 75.44 | 60.15 | 67.70 | 79.36 | 52.21 | 0.00 | 146.19 | 127.13 | 51.68 | 13.11 | 66.83 | 0.00 | 0.00 | 139.38 | 186.90 |
| Q32P51 | Heterogeneous nuclear ribonucleoprotein A1-like 2 | **1.08** | **0.86** | 195.67 | 141.34 | 210.46 | 133.09 | 131.01 | 56.85 | 321.67 | 371.48 | 97.35 | 190.35 | 105.39 | 106.31 | 107.26 | 389.66 | 363.78 |
| Q9UDY2 | Tight junction protein ZO-2 | **0.89** | **0.86** | 10.46 | 10.40 | 9.31 | 11.07 | 23.14 | 0.00 | 11.49 | 17.69 | 0.00 | 0.00 | 24.46 | 0.00 | 0.00 | 11.17 | 20.23 |
| P54920 | Alpha-soluble NSF attachment protein | **1.11** | **0.87** | 14.55 | 13.93 | 16.22 | 18.29 | 30.71 | 0.00 | 0.00 | 22.96 | 19.08 | 13.10 | 31.25 | 0.00 | 7.78 | 0.00 | 45.19 |
| Q6WRI0 | Immunoglobulin superfamily member 10 | **0.87** | **0.87** | 545.56 | 542.39 | 477.28 | 768.44 | 0.00 | 0.00 | 936.29 | 1198.67 | 592.81 | 0.00 | 1100.97 | 0.00 | 0.00 | 0.00 | 1762.71 |
| Q9NQC3 | Reticulon-4 | **1.03** | **0.87** | 214.68 | 87.25 | 222.13 | 60.38 | 226.02 | 144.83 | 146.96 | 358.08 | 197.52 | 201.97 | 283.77 | 235.02 | 115.52 | 271.98 | 224.55 |
| P04216 | Thy-1 membrane glycoprotein | **1.08** | **0.87** | 95.99 | 73.14 | 103.26 | 72.02 | 103.37 | 159.17 | 171.21 | 46.21 | 0.00 | 70.62 | 61.60 | 87.37 | 239.65 | 120.24 | 40.06 |
| P46783 | 40S ribosomal protein S10 | **0.93** | **0.87** | 91.89 | 60.00 | 85.40 | 68.80 | 75.81 | 0.00 | 127.75 | 158.05 | 97.83 | 82.14 | 69.87 | 0.00 | 58.92 | 92.06 | 209.39 |
| Q16563 | Synaptophysin-like protein 1 | **0.92** | **0.87** | 172.56 | 104.13 | 159.55 | 147.92 | 221.88 | 0.00 | 213.97 | 158.23 | 268.71 | 0.00 | 315.63 | 70.61 | 76.17 | 126.46 | 368.43 |
| P06744 | Glucose-6-phosphate isomerase | **1.05** | **0.87** | 282.23 | 200.79 | 296.31 | 58.90 | 275.87 | 62.04 | 253.41 | 609.05 | 210.78 | 211.81 | 358.21 | 335.56 | 265.72 | 259.70 | 346.86 |
| P08865 | 40S ribosomal protein SA | **1.04** | **0.87** | 237.39 | 106.70 | 248.02 | 107.99 | 252.32 | 116.21 | 325.66 | 352.85 | 139.90 | 282.33 | 141.85 | 134.16 | 217.99 | 289.90 | 421.90 |
| O60716 | Catenin delta-1 | **0.92** | **0.88** | 70.96 | 46.41 | 65.39 | 63.83 | 54.09 | 0.00 | 119.61 | 99.50 | 81.60 | 123.17 | 76.35 | 0.00 | 0.00 | 39.69 | 153.16 |
| P11021 | 78 kDa glucose-regulated protein | **1.03** | **0.88** | 639.77 | 127.31 | 658.14 | 225.27 | 725.67 | 542.00 | 637.59 | 801.39 | 492.22 | 659.45 | 466.61 | 460.68 | 495.73 | 947.04 | 919.33 |
| P0C6E5 | Putative high mobility group protein B3-like protein | **0.89** | **0.88** | 157.39 | 125.61 | 140.70 | 205.50 | 173.88 | 0.00 | 345.65 | 161.13 | 106.28 | 81.68 | 59.50 | 0.00 | 0.00 | 160.98 | 542.03 |
| P05387 | 60S acidic ribosomal protein P2 | **0.98** | **0.88** | 301.41 | 79.76 | 295.60 | 38.63 | 426.00 | 329.64 | 278.90 | 244.27 | 228.22 | 251.88 | 266.24 | 282.48 | 303.30 | 308.61 | 361.09 |
| Q96EP5 | DAZ-associated protein 1 | **1.11** | **0.88** | 28.95 | 39.70 | 32.12 | 21.92 | 0.00 | 0.00 | 75.33 | 69.44 | 0.00 | 30.66 | 52.75 | 0.00 | 17.44 | 32.76 | 59.10 |
| Q00688 | Peptidyl-prolyl cis-trans isomerase FKBP3 | **1.08** | **0.88** | 37.80 | 31.71 | 40.97 | 35.43 | 79.09 | 20.35 | 60.35 | 29.21 | 0.00 | 69.58 | 30.30 | 0.00 | 21.46 | 27.76 | 96.73 |
| Q9BRL6 | Serine/arginine-rich splicing factor 8 | **0.92** | **0.88** | 69.11 | 55.94 | 63.88 | 54.97 | 56.71 | 37.50 | 115.94 | 135.41 | 0.00 | 113.41 | 0.00 | 89.67 | 55.06 | 125.14 | 0.00 |
| P17980 | 26S protease regulatory subunit 6A | **1.09** | **0.88** | 18.90 | 18.70 | 20.57 | 17.26 | 24.57 | 0.00 | 26.75 | 43.19 | 0.00 | 20.69 | 0.00 | 0.00 | 26.86 | 38.82 | 37.03 |
| O43150 | Arf-GAP with SH3 domain, ANK repeat and PH domain-containing protein 2 | **1.10** | **0.88** | 57.66 | 89.57 | 63.39 | 23.13 | 47.25 | 213.88 | 27.18 | 0.00 | 0.00 | 35.91 | 85.67 | 83.26 | 83.59 | 43.49 | 48.43 |
| P11142 | Heat shock cognate 71 kDa protein | **0.96** | **0.88** | 862.38 | 259.01 | 830.08 | 407.87 | 1002.22 | 584.42 | 993.79 | 1143.44 | 588.04 | 828.74 | 519.99 | 535.07 | 442.44 | 1373.08 | 1281.19 |
| P35232 | Prohibitin | **1.06** | **0.88** | 108.51 | 57.95 | 114.84 | 77.12 | 127.34 | 46.16 | 182.21 | 134.05 | 52.77 | 163.08 | 73.84 | 63.60 | 53.88 | 84.00 | 250.61 |
| P61158 | Actin-related protein 3 | **1.04** | **0.89** | 127.51 | 71.27 | 132.62 | 41.19 | 114.17 | 74.75 | 201.98 | 200.05 | 46.58 | 88.53 | 189.68 | 144.95 | 97.58 | 107.03 | 167.97 |
| P31948 | Stress-induced-phosphoprotein 1 | **0.96** | **0.89** | 153.96 | 82.58 | 147.63 | 60.54 | 299.03 | 143.94 | 113.00 | 110.81 | 103.03 | 172.14 | 195.59 | 84.38 | 70.61 | 141.59 | 221.49 |
| Q13200 | 26S proteasome non-ATPase regulatory subunit 2 | **0.95** | **0.89** | 34.18 | 21.05 | 32.43 | 18.93 | 41.67 | 0.00 | 45.74 | 53.97 | 29.52 | 21.35 | 37.90 | 37.41 | 0.00 | 51.77 | 46.16 |
| P62318 | Small nuclear ribonucleoprotein Sm D3 | **0.96** | **0.89** | 118.27 | 44.15 | 113.30 | 66.13 | 87.20 | 68.27 | 158.30 | 169.51 | 108.08 | 170.11 | 123.64 | 0.00 | 71.07 | 155.42 | 159.56 |
| P0CW22 | 40S ribosomal protein S17-like | **0.93** | **0.89** | 56.82 | 39.58 | 53.07 | 48.00 | 58.44 | 0.00 | 78.72 | 105.11 | 41.84 | 0.00 | 83.84 | 0.00 | 43.92 | 69.94 | 120.70 |
| P04264 | Keratin, type II cytoskeletal 1 | **1.05** | **0.89** | 127.62 | 97.78 | 133.75 | 45.34 | 112.67 | 295.33 | 90.05 | 100.41 | 39.62 | 70.38 | 185.85 | 162.79 | 168.76 | 113.89 | 100.82 |
| P04196 | Histidine-rich glycoprotein | **1.10** | **0.90** | 9.57 | 8.99 | 10.49 | 12.62 | 0.00 | 0.00 | 17.86 | 17.46 | 12.56 | 5.87 | 0.00 | 0.00 | 31.14 | 20.58 | 5.35 |
| P30049 | ATP synthase subunit delta, mitochondrial | **1.05** | **0.90** | 79.12 | 56.67 | 82.82 | 32.76 | 140.17 | 0.00 | 113.33 | 98.98 | 43.14 | 135.08 | 98.43 | 59.66 | 64.81 | 45.24 | 93.70 |
| P09972 | Fructose-bisphosphate aldolase C | **1.09** | **0.90** | 129.51 | 148.02 | 140.62 | 126.42 | 46.50 | 0.00 | 222.99 | 344.19 | 33.88 | 285.53 | 0.00 | 68.78 | 78.71 | 101.01 | 309.67 |
| P30613 | Pyruvate kinase PKLR | **0.97** | **0.90** | 692.36 | 277.03 | 674.39 | 167.55 | 1018.00 | 403.68 | 401.16 | 835.21 | 803.74 | 755.42 | 587.62 | 958.20 | 594.28 | 476.16 | 674.65 |
| P06748 | Nucleophosmin | **0.96** | **0.90** | 376.41 | 210.68 | 360.86 | 175.57 | 331.17 | 267.18 | 657.05 | 509.34 | 117.31 | 505.62 | 261.40 | 152.43 | 202.58 | 482.06 | 561.08 |
| P40926 | Malate dehydrogenase, mitochondrial | **1.05** | **0.90** | 245.80 | 136.47 | 258.75 | 179.31 | 244.76 | 43.48 | 427.77 | 266.97 | 246.00 | 458.80 | 191.30 | 90.40 | 87.99 | 225.33 | 498.69 |
| Q9NZN3 | EH domain-containing protein 3 | **0.93** | **0.90** | 18.36 | 18.78 | 17.06 | 14.71 | 30.61 | 0.00 | 42.56 | 18.62 | 0.00 | 0.00 | 21.43 | 37.46 | 0.00 | 24.92 | 18.54 |
| P61586 | Transforming protein RhoA | **1.03** | **0.90** | 131.45 | 41.92 | 134.87 | 46.21 | 171.22 | 88.64 | 140.09 | 170.62 | 86.66 | 112.25 | 217.10 | 86.55 | 105.03 | 142.46 | 145.85 |
| Q9NS69 | Mitochondrial import receptor subunit TOM22 homolog | **0.94** | **0.90** | 21.23 | 20.04 | 19.87 | 16.11 | 27.48 | 0.00 | 41.64 | 37.04 | 0.00 | 35.19 | 22.41 | 0.00 | 0.00 | 27.09 | 34.52 |
| Q9Y3U8 | 60S ribosomal protein L36 | **1.08** | **0.91** | 63.47 | 71.58 | 68.26 | 58.47 | 38.11 | 0.00 | 149.62 | 129.61 | 0.00 | 82.65 | 0.00 | 68.74 | 0.00 | 119.91 | 138.25 |
| P52597 | Heterogeneous nuclear ribonucleoprotein F | **1.05** | **0.91** | 115.49 | 83.06 | 120.86 | 65.81 | 55.81 | 41.37 | 216.36 | 194.49 | 69.44 | 93.93 | 164.98 | 48.90 | 46.84 | 189.04 | 181.49 |
| P17844 | Probable ATP-dependent RNA helicase DDX5 | **1.04** | **0.91** | 92.16 | 43.05 | 95.97 | 58.74 | 61.41 | 56.02 | 135.07 | 143.10 | 65.21 | 106.84 | 36.71 | 30.42 | 78.32 | 148.47 | 175.06 |
| P62847 | 40S ribosomal protein S24 | **1.07** | **0.91** | 38.71 | 42.16 | 41.56 | 37.34 | 32.70 | 0.00 | 63.12 | 97.72 | 0.00 | 43.12 | 56.94 | 0.00 | 0.00 | 51.33 | 97.98 |
| P01857 | Ig gamma-1 chain C region | **0.93** | **0.91** | 2672.80 | 3320.19 | 2484.67 | 1838.62 | 413.58 | 8492.37 | 2258.46 | 1061.93 | 1137.64 | 1357.86 | 1262.06 | 1616.82 | 5664.31 | 3782.98 | 1224.00 |
| Q9ULZ3 | Apoptosis-associated speck-like protein containing a CARD | **0.94** | **0.91** | 31.63 | 30.75 | 29.74 | 24.54 | 68.50 | 50.94 | 0.00 | 38.69 | 0.00 | 70.27 | 29.15 | 0.00 | 19.47 | 43.49 | 16.02 |
| Q9Y536 | Peptidyl-prolyl cis-trans isomerase A-like 4A/B/C | **1.05** | **0.91** | 991.86 | 854.19 | 1042.72 | 637.39 | 987.55 | 0.00 | 2232.05 | 1298.19 | 441.53 | 859.72 | 697.93 | 691.10 | 562.80 | 1174.15 | 2270.62 |
| Q9NZM1 | Myoferlin | **0.96** | **0.91** | 31.85 | 20.38 | 30.44 | 20.37 | 49.86 | 0.00 | 45.71 | 39.92 | 23.76 | 62.26 | 32.96 | 37.31 | 28.51 | 21.62 | 0.00 |
| B2RPK0 | Putative high mobility group protein B1-like 1 | **1.03** | **0.91** | 464.81 | 149.49 | 480.15 | 269.80 | 398.87 | 619.71 | 569.95 | 492.57 | 242.94 | 601.84 | 352.87 | 707.43 | 51.49 | 388.52 | 778.72 |
| P62937 | Peptidyl-prolyl cis-trans isomerase A | **0.97** | **0.92** | 1091.93 | 482.31 | 1059.12 | 536.05 | 1214.49 | 534.68 | 1687.03 | 1356.66 | 666.78 | 959.04 | 786.57 | 891.79 | 453.06 | 1244.51 | 2019.73 |
| P61803 | Dolichyl-diphosphooligosaccharide--protein glycosyltransferase subunit DAD1 | **0.97** | **0.92** | 79.79 | 33.72 | 77.26 | 43.16 | 63.91 | 48.91 | 110.40 | 121.60 | 54.14 | 73.95 | 116.12 | 0.00 | 64.32 | 113.58 | 95.61 |
| P43307 | Translocon-associated protein subunit alpha | **1.03** | **0.92** | 54.10 | 5.55 | 55.61 | 34.39 | 62.03 | 49.11 | 55.49 | 48.48 | 55.41 | 91.42 | 53.58 | 0.00 | 75.47 | 80.44 | 32.74 |
| P04220 | Ig mu heavy chain disease protein | **1.06** | **0.92** | 128.45 | 107.41 | 136.71 | 151.87 | 0.00 | 288.05 | 102.36 | 167.61 | 84.21 | 109.06 | 78.84 | 78.94 | 443.56 | 41.84 | 68.02 |
| P30153 | Serine/threonine-protein phosphatase 2A 65 kDa regulatory subunit A alpha isoform | **1.04** | **0.92** | 70.65 | 43.37 | 73.77 | 58.70 | 70.29 | 0.00 | 104.54 | 107.85 | 70.56 | 105.92 | 63.79 | 0.00 | 22.14 | 88.95 | 161.85 |
| P01024 | Complement C3 | **1.03** | **0.93** | 184.64 | 82.54 | 191.09 | 129.53 | 263.24 | 270.83 | 183.62 | 91.87 | 113.64 | 148.47 | 83.67 | 399.64 | 294.21 | 151.09 | 69.43 |
| Q9BWM7 | Sideroflexin-3 | **0.93** | **0.93** | 16.30 | 15.29 | 15.10 | 24.82 | 23.31 | 0.00 | 25.40 | 32.78 | 0.00 | 0.00 | 0.00 | 0.00 | 0.00 | 32.20 | 58.40 |
| Q14011 | Cold-inducible RNA-binding protein | **1.06** | **0.93** | 34.56 | 23.42 | 36.47 | 39.79 | 30.86 | 0.00 | 57.09 | 55.75 | 29.11 | 106.91 | 26.99 | 0.00 | 0.00 | 34.29 | 50.60 |
| P52789 | Hexokinase-2 | **0.96** | **0.93** | 35.97 | 23.43 | 34.42 | 30.68 | 46.62 | 0.00 | 62.24 | 42.49 | 28.52 | 73.13 | 60.06 | 45.87 | 0.00 | 27.48 | 0.00 |
| P25311 | Zinc-alpha-2-glycoprotein | **1.08** | **0.93** | 41.59 | 79.89 | 44.95 | 39.51 | 24.73 | 183.21 | 0.00 | 0.00 | 0.00 | 0.00 | 34.10 | 68.38 | 91.02 | 76.19 | 0.00 |
| Q96G03 | Phosphoglucomutase-2 | **1.03** | **0.93** | 62.55 | 37.96 | 64.50 | 32.81 | 88.10 | 0.00 | 90.06 | 81.39 | 53.21 | 91.23 | 94.57 | 41.79 | 20.88 | 43.75 | 94.76 |
| O15511 | Actin-related protein 2/3 complex subunit 5 | **0.97** | **0.93** | 151.55 | 84.26 | 147.34 | 70.86 | 276.90 | 186.67 | 141.75 | 77.59 | 74.83 | 134.42 | 217.29 | 221.77 | 174.41 | 92.66 | 43.48 |
| P61163 | Alpha-centractin | **1.07** | **0.93** | 23.97 | 33.33 | 25.55 | 25.70 | 0.00 | 0.00 | 51.75 | 68.11 | 0.00 | 33.05 | 11.03 | 0.00 | 0.00 | 50.00 | 59.20 |
| Q9BUJ2 | Heterogeneous nuclear ribonucleoprotein U-like protein 1 | **1.05** | **0.93** | 29.52 | 23.52 | 30.90 | 28.61 | 18.19 | 0.00 | 49.31 | 57.37 | 22.71 | 0.00 | 49.20 | 0.00 | 17.56 | 54.04 | 64.59 |
| P01861 | Ig gamma-4 chain C region | **0.95** | **0.93** | 1701.04 | 2141.82 | 1612.16 | 1253.38 | 246.74 | 5440.77 | 1512.99 | 568.41 | 736.28 | 891.30 | 846.39 | 1282.38 | 4096.27 | 1629.23 | 927.39 |
| P02766 | Transthyretin | **0.94** | **0.94** | 36.07 | 49.37 | 34.07 | 29.89 | 39.43 | 119.30 | 0.00 | 0.00 | 21.61 | 37.94 | 69.80 | 0.00 | 63.27 | 33.43 | 0.00 |
| O75351 | Vacuolar protein sorting-associated protein 4B | **1.07** | **0.94** | 45.07 | 59.61 | 48.15 | 65.16 | 45.03 | 0.00 | 145.31 | 35.02 | 0.00 | 32.60 | 152.98 | 0.00 | 0.00 | 0.00 | 103.31 |
| P30038 | Delta-1-pyrroline-5-carboxylate dehydrogenase, mitochondrial | **0.94** | **0.94** | 40.89 | 21.68 | 38.62 | 64.04 | 75.07 | 33.35 | 47.46 | 29.79 | 18.76 | 79.73 | 0.00 | 0.00 | 0.00 | 0.00 | 151.98 |
| Q9BRP8 | Partner of Y14 and mago | **0.95** | **0.94** | 27.86 | 31.55 | 26.49 | 26.63 | 75.50 | 0.00 | 23.92 | 39.88 | 0.00 | 38.57 | 20.62 | 0.00 | 0.00 | 29.04 | 70.72 |
| Q13733 | Sodium/potassium-transporting ATPase subunit alpha-4 | **0.96** | **0.94** | 44.13 | 41.04 | 42.34 | 36.18 | 69.88 | 0.00 | 86.01 | 64.74 | 0.00 | 82.38 | 65.78 | 0.00 | 35.85 | 0.00 | 70.02 |
| Q9BQK8 | Phosphatidate phosphatase LPIN3 | **1.03** | **0.94** | 794.91 | 558.76 | 822.21 | 592.46 | 776.56 | 0.00 | 1003.18 | 1539.13 | 655.69 | 473.45 | 918.16 | 0.00 | 630.98 | 1689.76 | 1220.92 |
| Q14444 | Caprin-1 | **0.96** | **0.94** | 31.73 | 34.07 | 30.39 | 25.52 | 82.15 | 39.01 | 0.00 | 37.51 | 0.00 | 27.19 | 49.47 | 0.00 | 0.00 | 56.57 | 49.09 |
| P22234 | Multifunctional protein ADE2 | **0.95** | **0.94** | 26.60 | 25.49 | 25.38 | 28.41 | 31.98 | 0.00 | 52.76 | 48.28 | 0.00 | 54.61 | 0.00 | 0.00 | 0.00 | 57.46 | 40.24 |
| P00738 | Haptoglobin | **1.03** | **0.94** | 502.25 | 359.41 | 515.06 | 226.62 | 263.54 | 1139.28 | 373.22 | 390.02 | 345.19 | 672.72 | 223.33 | 770.47 | 697.75 | 331.51 | 394.57 |
| O43151 | Methylcytosine dioxygenase TET3 | **0.96** | **0.95** | 78.41 | 76.96 | 74.93 | 87.49 | 85.07 | 0.00 | 159.59 | 147.40 | 0.00 | 158.41 | 0.00 | 0.00 | 0.00 | 98.26 | 192.89 |
| Q14624 | Inter-alpha-trypsin inhibitor heavy chain H4 | **0.96** | **0.95** | 26.49 | 14.20 | 25.54 | 27.18 | 14.83 | 49.86 | 20.98 | 17.28 | 29.52 | 26.21 | 14.69 | 70.20 | 42.16 | 0.00 | 0.00 |
| P80748 | Ig lambda chain V-III region LOI | **1.06** | **0.95** | 45.03 | 78.08 | 47.60 | 45.79 | 0.00 | 180.31 | 44.85 | 0.00 | 0.00 | 0.00 | 21.63 | 54.06 | 131.51 | 51.85 | 26.56 |
| Q9H4Z3 | Phosphorylated CTD-interacting factor 1 | **1.05** | **0.95** | 935.60 | 1221.58 | 982.31 | 1096.49 | 1386.82 | 0.00 | 419.51 | 2871.67 | 0.00 | 1613.90 | 0.00 | 0.00 | 0.00 | 2003.28 | 2276.68 |
| Q99829 | Copine-1 | **0.96** | **0.95** | 18.37 | 18.57 | 17.71 | 14.28 | 38.57 | 0.00 | 17.69 | 35.59 | 0.00 | 18.98 | 29.85 | 0.00 | 29.71 | 0.00 | 27.69 |
| P98160 | Basement membrane-specific heparan sulfate proteoglycan core protein | **1.05** | **0.95** | 221.52 | 341.03 | 233.48 | 249.38 | 103.27 | 34.34 | 828.17 | 108.29 | 33.53 | 120.07 | 140.03 | 298.25 | 100.30 | 709.89 | 32.33 |
| Q9Y3B4 | Pre-mRNA branch site protein p14 | **1.06** | **0.95** | 12.87 | 12.11 | 13.59 | 21.55 | 17.12 | 0.00 | 25.38 | 21.85 | 0.00 | 0.00 | 0.00 | 0.00 | 0.00 | 33.54 | 48.01 |
| Q14103 | Heterogeneous nuclear ribonucleoprotein D0 | **1.02** | **0.95** | 213.85 | 86.00 | 217.23 | 82.95 | 163.70 | 199.17 | 282.05 | 317.51 | 106.83 | 262.24 | 168.84 | 117.50 | 146.80 | 298.23 | 309.80 |
| P49590 | Probable histidine--tRNA ligase, mitochondrial | **1.04** | **0.95** | 18.70 | 17.55 | 19.48 | 22.34 | 0.00 | 0.00 | 35.19 | 33.72 | 24.58 | 0.00 | 34.83 | 0.00 | 50.86 | 31.20 | 0.00 |
| P68104 | Elongation factor 1-alpha 1 | **1.01** | **0.95** | 1746.15 | 828.31 | 1771.61 | 491.54 | 1583.49 | 729.00 | 1692.72 | 3041.03 | 1684.52 | 2449.94 | 1017.16 | 1482.64 | 1709.30 | 1985.60 | 1984.99 |
| Q5VTE0 | Putative elongation factor 1-alpha-like 3 | **1.01** | **0.95** | 1746.15 | 828.31 | 1771.61 | 491.54 | 1583.49 | 729.00 | 1692.72 | 3041.03 | 1684.52 | 2449.94 | 1017.16 | 1482.64 | 1709.30 | 1985.60 | 1984.99 |
| P02763 | Alpha-1-acid glycoprotein 1 | **0.96** | **0.95** | 234.06 | 278.93 | 225.39 | 174.73 | 128.70 | 727.99 | 84.48 | 165.64 | 63.50 | 48.52 | 130.37 | 399.18 | 469.86 | 75.83 | 228.59 |
| Q99714 | 3-hydroxyacyl-CoA dehydrogenase type-2 | **1.02** | **0.95** | 49.00 | 38.09 | 50.09 | 13.81 | 53.91 | 0.00 | 90.88 | 78.59 | 21.64 | 42.41 | 46.31 | 49.56 | 35.68 | 50.56 | 76.01 |
| P10809 | 60 kDa heat shock protein, mitochondrial | **0.98** | **0.96** | 273.30 | 116.14 | 267.94 | 175.39 | 287.76 | 70.77 | 357.14 | 313.89 | 336.92 | 525.37 | 287.00 | 96.28 | 101.94 | 179.63 | 417.42 |
| P62310 | U6 snRNA-associated Sm-like protein LSm3 | **1.05** | **0.96** | 18.25 | 17.04 | 19.15 | 31.85 | 34.68 | 31.70 | 0.00 | 0.00 | 24.87 | 0.00 | 75.79 | 0.00 | 0.00 | 0.00 | 39.08 |
| Q86SZ2 | Trafficking protein particle complex subunit 6B | **0.99** | **0.96** | 702.14 | 135.82 | 695.17 | 260.04 | 868.16 | 545.80 | 758.98 | 759.26 | 578.49 | 840.78 | 675.85 | 573.98 | 244.94 | 912.29 | 923.18 |
| Q86W56 | Poly(ADP-ribose) glycohydrolase | **0.99** | **0.96** | 702.14 | 135.82 | 695.17 | 260.04 | 868.16 | 545.80 | 758.98 | 759.26 | 578.49 | 840.78 | 675.85 | 573.98 | 244.94 | 912.29 | 923.18 |
| Q13162 | Peroxiredoxin-4 | **1.03** | **0.96** | 147.90 | 103.31 | 151.99 | 152.09 | 128.45 | 0.00 | 224.01 | 121.10 | 265.92 | 0.00 | 205.66 | 0.00 | 105.99 | 401.54 | 198.73 |
| P07741 | Adenine phosphoribosyltransferase | **0.98** | **0.96** | 67.96 | 45.14 | 66.68 | 38.11 | 93.40 | 0.00 | 96.30 | 106.53 | 43.55 | 73.29 | 69.86 | 33.10 | 43.46 | 43.10 | 137.27 |
| Q96C19 | EF-hand domain-containing protein D2 | **0.98** | **0.96** | 27.17 | 16.05 | 26.55 | 22.67 | 40.34 | 0.00 | 26.01 | 33.43 | 36.05 | 44.06 | 34.36 | 0.00 | 12.46 | 58.53 | 9.91 |
| O00299 | Chloride intracellular channel protein 1 | **0.99** | **0.96** | 300.50 | 122.71 | 296.92 | 117.20 | 462.64 | 137.47 | 244.38 | 364.95 | 293.04 | 228.93 | 370.44 | 378.90 | 218.57 | 443.59 | 141.09 |
| P09429 | High mobility group protein B1 | **1.02** | **0.96** | 650.87 | 245.27 | 661.68 | 452.39 | 554.53 | 535.01 | 1019.87 | 758.85 | 386.10 | 822.53 | 417.73 | 707.43 | 51.49 | 564.83 | 1406.06 |
| P43034 | Platelet-activating factor acetylhydrolase IB subunit alpha | **0.97** | **0.96** | 16.28 | 22.34 | 15.75 | 14.79 | 0.00 | 0.00 | 42.92 | 38.46 | 0.00 | 29.61 | 15.62 | 0.00 | 13.51 | 0.00 | 35.75 |
| Q8WVQ1 | Soluble calcium-activated nucleotidase 1 | **1.03** | **0.97** | 50.63 | 49.55 | 52.18 | 61.58 | 57.58 | 0.00 | 107.71 | 87.88 | 0.00 | 74.76 | 0.00 | 0.00 | 0.00 | 93.50 | 144.79 |
| Q9BPX5 | Actin-related protein 2/3 complex subunit 5-like protein | **1.03** | **0.97** | 20.00 | 21.37 | 20.65 | 26.01 | 49.41 | 0.00 | 18.04 | 32.56 | 0.00 | 0.00 | 64.73 | 28.84 | 0.00 | 30.32 | 0.00 |
| P05388 | 60S acidic ribosomal protein P0 | **1.01** | **0.97** | 135.76 | 62.87 | 137.45 | 65.87 | 101.37 | 64.78 | 188.16 | 214.40 | 110.09 | 137.91 | 92.03 | 64.91 | 113.81 | 164.09 | 251.92 |
| P01614 | Ig kappa chain V-II region Cum | **1.04** | **0.97** | 217.47 | 351.53 | 225.27 | 274.15 | 0.00 | 834.86 | 156.98 | 0.00 | 95.52 | 113.68 | 165.14 | 177.56 | 769.96 | 125.26 | 0.00 |
| P06309 | Ig kappa chain V-II region GM607 (Fragment) | **1.04** | **0.97** | 217.47 | 351.53 | 225.27 | 274.15 | 0.00 | 834.86 | 156.98 | 0.00 | 95.52 | 113.68 | 165.14 | 177.56 | 769.96 | 125.26 | 0.00 |
| P06310 | Ig kappa chain V-II region RPMI 6410 | **1.04** | **0.97** | 217.47 | 351.53 | 225.27 | 274.15 | 0.00 | 834.86 | 156.98 | 0.00 | 95.52 | 113.68 | 165.14 | 177.56 | 769.96 | 125.26 | 0.00 |
| P40939 | Trifunctional enzyme subunit alpha, mitochondrial | **0.99** | **0.97** | 90.44 | 61.92 | 89.26 | 31.02 | 135.44 | 0.00 | 145.57 | 117.59 | 53.60 | 67.21 | 137.49 | 69.14 | 58.77 | 87.90 | 115.02 |
| Q13185 | Chromobox protein homolog 3 | **1.02** | **0.97** | 31.18 | 30.57 | 31.80 | 22.35 | 37.66 | 0.00 | 68.87 | 49.39 | 0.00 | 32.34 | 43.65 | 15.69 | 0.00 | 34.45 | 64.65 |
| P47756 | F-actin-capping protein subunit beta | **1.01** | **0.97** | 119.77 | 34.08 | 120.78 | 48.59 | 73.69 | 136.68 | 111.58 | 165.58 | 111.32 | 120.90 | 192.99 | 76.98 | 70.72 | 100.46 | 162.65 |
| Q12792 | Twinfilin-1 | **1.02** | **0.97** | 20.01 | 18.41 | 20.50 | 23.59 | 0.00 | 0.00 | 32.89 | 36.80 | 30.36 | 28.12 | 44.95 | 0.00 | 0.00 | 0.00 | 49.92 |
| P35080 | Profilin-2 | **1.02** | **0.97** | 71.55 | 46.23 | 73.06 | 81.07 | 81.13 | 0.00 | 121.43 | 57.88 | 97.29 | 0.00 | 220.35 | 89.56 | 0.00 | 69.99 | 58.48 |
| P61978 | Heterogeneous nuclear ribonucleoprotein K | **1.01** | **0.97** | 375.66 | 146.32 | 378.78 | 133.70 | 323.02 | 249.69 | 597.86 | 445.18 | 262.53 | 413.74 | 447.66 | 233.71 | 205.52 | 417.67 | 554.37 |
| P51157 | Ras-related protein Rab-28 | **0.98** | **0.97** | 250.22 | 170.67 | 245.36 | 251.70 | 248.79 | 0.00 | 297.11 | 476.57 | 228.63 | 341.43 | 0.00 | 271.13 | 186.48 | 0.00 | 673.14 |
| P36873 | Serine/threonine-protein phosphatase PP1-gamma catalytic subunit | **0.98** | **0.97** | 50.11 | 37.95 | 49.15 | 51.79 | 41.77 | 0.00 | 88.80 | 87.26 | 32.73 | 42.24 | 45.12 | 0.00 | 0.00 | 68.08 | 139.47 |
| P62136 | Serine/threonine-protein phosphatase PP1-alpha catalytic subunit | **1.02** | **0.97** | 48.81 | 38.80 | 49.78 | 53.11 | 41.77 | 0.00 | 88.80 | 87.26 | 26.20 | 42.24 | 45.12 | 0.00 | 0.00 | 68.08 | 143.23 |
| Q92616 | Translational activator GCN1 | **0.99** | **0.97** | 238.96 | 76.13 | 236.27 | 164.48 | 301.59 | 217.65 | 335.40 | 166.93 | 173.23 | 416.39 | 289.54 | 0.00 | 229.56 | 389.51 | 92.62 |
| Q9NVN8 | Guanine nucleotide-binding protein-like 3-like protein | **1.02** | **0.98** | 89.56 | 101.48 | 91.77 | 120.82 | 67.11 | 0.00 | 236.89 | 143.81 | 0.00 | 61.24 | 0.00 | 0.00 | 0.00 | 242.30 | 247.10 |
| P20908 | Collagen alpha-1(V) chain | **1.02** | **0.98** | 279.17 | 398.85 | 284.82 | 162.71 | 200.64 | 966.04 | 229.18 | 0.00 | 0.00 | 310.06 | 192.61 | 408.83 | 418.61 | 378.81 | 0.00 |
| P04003 | C4b-binding protein alpha chain | **0.96** | **0.98** | 19.28 | 21.94 | 18.56 | 45.46 | 0.00 | 51.52 | 30.26 | 0.00 | 14.59 | 0.00 | 0.00 | 111.35 | 0.00 | 0.00 | 0.00 |
| Q04760 | Lactoylglutathione lyase | **1.01** | **0.98** | 52.09 | 45.80 | 52.82 | 28.53 | 121.50 | 0.00 | 24.02 | 54.68 | 60.23 | 45.41 | 107.85 | 32.74 | 37.90 | 34.64 | 58.38 |
| A8MWD9 | Small nuclear ribonucleoprotein G-like protein | **0.98** | **0.98** | 11.54 | 17.17 | 11.30 | 10.13 | 19.31 | 0.00 | 38.37 | 0.00 | 0.00 | 18.88 | 0.00 | 0.00 | 8.09 | 23.91 | 16.90 |
| P62308 | Small nuclear ribonucleoprotein G | **0.98** | **0.98** | 11.54 | 17.17 | 11.30 | 10.13 | 19.31 | 0.00 | 38.37 | 0.00 | 0.00 | 18.88 | 0.00 | 0.00 | 8.09 | 23.91 | 16.90 |
| A6NKZ8 | Putative tubulin beta chain-like protein ENSP00000290377 | **1.01** | **0.98** | 316.39 | 181.38 | 319.43 | 169.84 | 31.70 | 334.75 | 379.18 | 531.20 | 305.16 | 293.38 | 218.00 | 115.56 | 304.62 | 618.15 | 366.86 |
| P00505 | Aspartate aminotransferase, mitochondrial | **1.01** | **0.98** | 129.02 | 50.46 | 129.95 | 54.98 | 156.05 | 43.98 | 148.56 | 171.71 | 124.82 | 122.62 | 214.27 | 103.07 | 59.11 | 171.90 | 108.74 |
| O43684 | Mitotic checkpoint protein BUB3 | **0.98** | **0.98** | 18.29 | 17.90 | 17.97 | 20.40 | 19.94 | 0.00 | 35.88 | 35.61 | 0.00 | 38.05 | 0.00 | 0.00 | 0.00 | 26.65 | 43.14 |
| Q9H9S4 | Calcium-binding protein 39-like | **1.02** | **0.98** | 8.62 | 9.48 | 8.79 | 13.68 | 9.09 | 0.00 | 11.06 | 22.94 | 0.00 | 28.55 | 24.18 | 0.00 | 0.00 | 0.00 | 0.00 |
| P27824 | Calnexin | **1.00** | **0.98** | 177.06 | 76.27 | 176.19 | 34.46 | 190.11 | 112.31 | 226.72 | 268.73 | 87.41 | 161.37 | 233.44 | 165.81 | 131.17 | 193.18 | 172.16 |
| Q86V81 | THO complex subunit 4 | **0.99** | **0.98** | 28.53 | 29.42 | 28.15 | 33.03 | 27.56 | 0.00 | 66.19 | 48.92 | 0.00 | 43.08 | 0.00 | 0.00 | 0.00 | 48.12 | 77.71 |
| Q8WUD1 | Ras-related protein Rab-2B | **1.01** | **0.98** | 46.77 | 28.11 | 47.12 | 26.77 | 67.44 | 0.00 | 65.40 | 59.77 | 41.23 | 67.02 | 44.70 | 0.00 | 65.29 | 36.06 | 69.65 |
| P30101 | Protein disulfide-isomerase A3 | **1.00** | **0.98** | 479.86 | 73.06 | 478.05 | 179.64 | 466.99 | 450.41 | 453.30 | 607.14 | 421.45 | 778.97 | 520.30 | 264.19 | 427.75 | 532.43 | 344.67 |
| P62140 | Serine/threonine-protein phosphatase PP1-beta catalytic subunit | **1.01** | **0.99** | 55.25 | 42.75 | 55.78 | 46.76 | 49.16 | 0.00 | 107.10 | 87.26 | 32.73 | 42.24 | 50.48 | 0.00 | 34.45 | 68.08 | 139.47 |
| O00487 | 26S proteasome non-ATPase regulatory subunit 14 | **0.99** | **0.99** | 23.07 | 23.29 | 22.88 | 12.69 | 40.87 | 0.00 | 0.00 | 51.15 | 23.35 | 29.79 | 0.00 | 22.16 | 19.83 | 28.93 | 36.56 |
| P24844 | Myosin regulatory light polypeptide 9 | **1.01** | **0.99** | 371.34 | 270.69 | 373.53 | 134.00 | 826.65 | 390.38 | 293.69 | 191.73 | 154.24 | 311.11 | 362.54 | 589.01 | 414.16 | 384.64 | 179.73 |
| P30085 | UMP-CMP kinase | **1.01** | **0.99** | 47.43 | 29.92 | 47.72 | 27.98 | 66.64 | 0.00 | 77.88 | 42.36 | 50.25 | 31.06 | 95.86 | 40.80 | 16.88 | 38.60 | 63.12 |
| P62081 | 40S ribosomal protein S7 | **0.99** | **0.99** | 90.33 | 42.82 | 89.68 | 77.18 | 75.05 | 34.37 | 138.94 | 127.62 | 75.64 | 93.44 | 107.16 | 0.00 | 34.61 | 78.06 | 224.79 |
| P01598 | Ig kappa chain V-I region EU | **1.01** | **0.99** | 64.77 | 144.84 | 65.73 | 59.08 | 0.00 | 323.87 | 0.00 | 0.00 | 0.00 | 31.50 | 48.87 | 57.52 | 171.80 | 84.67 | 0.00 |
| Q03252 | Lamin-B2 | **1.01** | **0.99** | 58.59 | 47.29 | 58.97 | 33.49 | 34.08 | 0.00 | 50.72 | 85.20 | 122.95 | 99.85 | 66.23 | 0.00 | 67.30 | 73.15 | 47.27 |
| P63241 | Eukaryotic translation initiation factor 5A-1 | **1.01** | **0.99** | 129.55 | 65.80 | 130.23 | 76.54 | 95.36 | 101.08 | 195.84 | 201.68 | 53.79 | 121.04 | 111.25 | 52.55 | 70.94 | 159.63 | 266.00 |
| Q9NQH7 | Probable Xaa-Pro aminopeptidase 3 | **0.99** | **0.99** | 163.19 | 153.94 | 161.36 | 260.99 | 301.97 | 0.00 | 208.65 | 305.31 | 0.00 | 365.50 | 0.00 | 0.00 | 0.00 | 0.00 | 602.67 |
| Q9NVC6 | Mediator of RNA polymerase II transcription subunit 17 | **0.99** | **0.99** | 226.07 | 314.55 | 223.62 | 316.36 | 486.27 | 0.00 | 644.08 | 0.00 | 0.00 | 839.57 | 0.00 | 127.19 | 120.03 | 254.95 | 0.00 |
| P01620 | Ig kappa chain V-III region SIE | **1.01** | **0.99** | 216.23 | 346.47 | 217.47 | 190.67 | 0.00 | 830.69 | 123.78 | 46.83 | 79.85 | 86.73 | 142.55 | 147.42 | 591.08 | 238.46 | 98.57 |
| P01623 | Ig kappa chain V-III region WOL | **1.01** | **0.99** | 216.23 | 346.47 | 217.47 | 190.67 | 0.00 | 830.69 | 123.78 | 46.83 | 79.85 | 86.73 | 142.55 | 147.42 | 591.08 | 238.46 | 98.57 |
| Q13151 | Heterogeneous nuclear ribonucleoprotein A0 | **1.00** | **1.00** | 51.71 | 31.55 | 51.84 | 61.82 | 57.70 | 0.00 | 59.86 | 86.28 | 54.69 | 66.28 | 0.00 | 0.00 | 0.00 | 143.47 | 101.26 |
| P36957 | Dihydrolipoyllysine-residue succinyltransferase component of 2-oxoglutarate dehydrogenase complex, mitochondrial | **1.00** | **1.00** | 43.42 | 24.83 | 43.37 | 14.79 | 49.91 | 0.00 | 57.17 | 61.38 | 48.63 | 70.41 | 41.75 | 31.90 | 43.98 | 28.28 | 43.90 |
| P56134 | ATP synthase subunit f, mitochondrial | **1.00** | **1.00** | 50.72 | 54.21 | 50.79 | 30.46 | 38.60 | 0.00 | 110.15 | 104.87 | 0.00 | 59.55 | 58.61 | 0.00 | 41.16 | 51.88 | 93.55 |
| P02774 | Vitamin D-binding protein | **1.00** | **1.00** | 64.27 | 35.29 | 64.27 | 39.37 | 42.28 | 114.85 | 75.19 | 67.33 | 21.71 | 53.89 | 49.01 | 37.32 | 141.97 | 64.63 | 38.82 |

1. **Trans Proteomic Pipeline**

| **Accession** | **Description** | **Fold change pT2+/pTa** | **p-value** | **Mean_pTa** | **SD_pTa** | **Mean_pT2+** | **SD_pT2+** | **3_pTa** | **6_pTa** | **11_pTa** | **16_pTa** | **19_pTa** | **9_pT2+** | **12_pT2+** | **13_pT2+** | **14_pT2+** | **15_pT2+** | **17_pT2+** |
| --- | --- | --- | --- | --- | --- | --- | --- | --- | --- | --- | --- | --- | --- | --- | --- | --- | --- | --- |
| O43493 | Trans-Golgi network integral membrane protein 2 | **4.67** | **0.00** | 30.55 | 41.91 | 142.80 | 32.24 | 0.00 | 0.00 | 0.00 | 80.08 | 72.68 | 153.97 | 183.32 | 169.40 | 132.13 | 96.29 | 121.68 |
| Q7KZF4 | Staphylococcal nuclease domain-containing protein 1 | **5.21** | **0.00** | 40.33 | 51.08 | 209.98 | 57.41 | 31.38 | 0.00 | 30.83 | 128.52 | 10.94 | 97.74 | 232.76 | 204.55 | 247.62 | 226.85 | 250.36 |
| P50552 | Vasodilator-stimulated phosphoprotein | **0.26** | **0.00** | 195.94 | 58.96 | 50.24 | 46.97 | 245.43 | 182.44 | 267.50 | 128.60 | 155.72 | 113.01 | 63.51 | 34.46 | 0.00 | 90.45 | 0.00 |
| P17612 | cAMP-dependent protein kinase catalytic subunit alpha | **only in pT2+** | **0.00** | 0.00 | 0.00 | 113.09 | 111.96 | 0.00 | 0.00 | 0.00 | 0.00 | 0.00 | 16.36 | 321.19 | 133.51 | 27.36 | 65.71 | 114.41 |
| Q14956 | Transmembrane glycoprotein NMB | **only in pT2+** | **0.00** | 0.00 | 0.00 | 204.59 | 85.29 | 0.00 | 0.00 | 0.00 | 0.00 | 0.00 | 212.02 | 220.91 | 77.24 | 200.39 | 174.83 | 342.16 |
| Q9UN86 | Ras GTPase-activating protein-binding protein 2 | **5.01** | **0.00** | 24.90 | 41.51 | 124.79 | 44.94 | 0.00 | 0.00 | 0.00 | 95.73 | 28.75 | 46.14 | 120.83 | 134.72 | 116.17 | 179.92 | 150.95 |
| Q9Y3I0 | tRNA-splicing ligase RtcB homolog | **4.06** | **0.00** | 42.25 | 62.50 | 171.36 | 51.20 | 0.00 | 0.00 | 72.22 | 139.05 | 0.00 | 229.13 | 159.14 | 119.86 | 113.05 | 230.90 | 176.06 |
| P49720 | Proteasome subunit beta type-3 | **5.48** | **0.00** | 100.99 | 71.86 | 553.16 | 253.17 | 68.09 | 0.00 | 117.93 | 192.97 | 125.98 | 343.25 | 613.09 | 897.97 | 695.71 | 188.64 | 580.32 |
| O95994 | Anterior gradient protein 2 homolog | **0.02** | **0.00** | 1361.63 | 511.63 | 31.18 | 76.39 | 1602.62 | 720.12 | 917.02 | 1890.60 | 1677.81 | 187.11 | 0.00 | 0.00 | 0.00 | 0.00 | 0.00 |
| O43852 | Calumenin | **3.75** | **0.01** | 141.79 | 128.01 | 532.39 | 202.46 | 269.35 | 248.33 | 13.31 | 177.94 | 0.00 | 390.92 | 764.73 | 525.95 | 764.04 | 491.21 | 257.50 |
| P52815 | 39S ribosomal protein L12, mitochondrial | **0.21** | **0.01** | 198.20 | 84.44 | 42.51 | 56.66 | 315.12 | 157.83 | 257.56 | 145.40 | 115.07 | 96.03 | 129.66 | 0.00 | 0.00 | 0.00 | 29.38 |
| P14854 | Cytochrome c oxidase subunit 6B1 | **0.17** | **0.01** | 669.36 | 343.10 | 113.77 | 134.27 | 1193.72 | 710.09 | 702.57 | 286.00 | 454.45 | 91.58 | 84.63 | 0.00 | 0.00 | 362.82 | 143.61 |
| P60866 | 40S ribosomal protein S20 | **1.65** | **0.01** | 1015.25 | 351.42 | 1678.55 | 258.25 | 914.48 | 585.08 | 1196.04 | 1510.33 | 870.35 | 1738.16 | 1881.38 | 1240.02 | 1681.27 | 1563.45 | 1967.00 |
| P49721 | Proteasome subunit beta type-2 | **3.66** | **0.01** | 75.28 | 53.01 | 275.78 | 119.61 | 74.24 | 0.00 | 149.68 | 72.03 | 80.45 | 119.34 | 236.79 | 374.61 | 423.26 | 170.58 | 330.13 |
| P62318 | Small nuclear ribonucleoprotein Sm D3 | **1.85** | **0.01** | 512.15 | 243.69 | 945.33 | 174.63 | 486.48 | 117.67 | 636.27 | 765.12 | 555.22 | 1176.84 | 1000.85 | 903.49 | 766.32 | 739.31 | 1085.16 |
| P27348 | 14-3-3 protein theta | **1.33** | **0.01** | 1476.79 | 204.20 | 1968.53 | 258.07 | 1214.35 | 1403.12 | 1439.35 | 1766.84 | 1560.27 | 1967.16 | 2186.04 | 1934.82 | 1514.98 | 2249.15 | 1959.02 |
| P08727 | Keratin, type I cytoskeletal 19 | **0.48** | **0.01** | 5787.64 | 1540.68 | 2794.09 | 1304.71 | 7541.52 | 5468.82 | 3395.41 | 6005.28 | 6527.15 | 4520.18 | 3254.60 | 2828.20 | 626.72 | 3324.09 | 2210.75 |
| P49257 | Protein ERGIC-53 | **4.50** | **0.01** | 43.95 | 53.05 | 197.77 | 87.31 | 23.06 | 0.00 | 75.55 | 121.14 | 0.00 | 159.69 | 126.76 | 250.64 | 242.73 | 87.76 | 319.04 |
| Q04917 | 14-3-3 protein eta | **1.91** | **0.01** | 647.24 | 376.99 | 1237.98 | 182.10 | 663.72 | 0.00 | 937.52 | 876.94 | 758.03 | 1403.75 | 1147.01 | 1512.00 | 1087.76 | 1059.09 | 1218.25 |
| P52209 | 6-phosphogluconate dehydrogenase, decarboxylating | **2.52** | **0.01** | 417.40 | 286.18 | 1053.81 | 331.67 | 230.75 | 59.12 | 658.47 | 395.95 | 742.71 | 543.71 | 1475.99 | 1024.03 | 1057.84 | 1338.93 | 882.38 |
| P27797 | Calreticulin | **1.51** | **0.01** | 1409.63 | 490.80 | 2127.26 | 171.33 | 1114.70 | 1106.70 | 1530.80 | 2220.89 | 1075.05 | 2080.19 | 2354.45 | 1934.75 | 2068.89 | 2004.57 | 2320.71 |
| P07355 | Annexin A2 | **1.49** | **0.01** | 2022.64 | 370.36 | 3005.14 | 547.37 | 2104.07 | 1955.20 | 1539.76 | 2568.84 | 1945.33 | 2631.53 | 2659.10 | 3532.42 | 3789.40 | 2417.20 | 3001.22 |
| O76070 | Gamma-synuclein | **0.21** | **0.01** | 2478.29 | 1314.74 | 512.52 | 536.18 | 2885.31 | 316.71 | 2981.89 | 2382.65 | 3824.90 | 1175.17 | 1017.61 | 118.92 | 0.00 | 763.43 | 0.00 |
| P38606 | V-type proton ATPase catalytic subunit A | **15.98** | **0.01** | 5.66 | 7.75 | 90.45 | 50.27 | 0.00 | 0.00 | 14.26 | 14.05 | 0.00 | 37.69 | 113.06 | 98.10 | 68.88 | 49.87 | 175.12 |
| P61009 | Signal peptidase complex subunit 3 | **4.88** | **0.01** | 47.00 | 64.36 | 229.43 | 110.13 | 0.00 | 0.00 | 118.66 | 116.34 | 0.00 | 124.47 | 314.14 | 99.80 | 300.18 | 362.57 | 175.44 |
| Q02978 | Mitochondrial 2-oxoglutarate/malate carrier protein | **3.77** | **0.01** | 53.64 | 72.23 | 202.03 | 75.65 | 16.09 | 0.00 | 85.96 | 166.15 | 0.00 | 212.71 | 289.88 | 190.76 | 138.14 | 100.07 | 280.62 |
| P04083 | Annexin A1 | **2.46** | **0.01** | 676.10 | 272.27 | 1661.89 | 605.42 | 700.56 | 830.22 | 405.27 | 1034.13 | 410.31 | 744.93 | 1796.07 | 1899.92 | 1924.91 | 1167.02 | 2438.49 |
| Q9UGJ0 | 5'-AMP-activated protein kinase subunit gamma-2 | **0.32** | **0.01** | 739.95 | 317.23 | 238.00 | 174.01 | 918.72 | 802.69 | 590.51 | 281.55 | 1106.28 | 522.66 | 246.09 | 134.69 | 0.00 | 232.25 | 292.29 |
| P48735 | Isocitrate dehydrogenase [NADP], mitochondrial | **2.41** | **0.01** | 453.01 | 206.90 | 1090.65 | 393.02 | 616.16 | 162.73 | 362.65 | 681.20 | 442.34 | 1204.61 | 1064.83 | 744.80 | 582.59 | 1264.12 | 1682.98 |
| P26639 | Threonine--tRNA ligase, cytoplasmic | **7.98** | **0.01** | 14.86 | 21.41 | 118.64 | 69.81 | 0.00 | 0.00 | 0.00 | 46.57 | 27.72 | 51.91 | 108.21 | 244.53 | 118.41 | 57.10 | 131.69 |
| P14091 | Cathepsin E | **only in pTa** | **0.01** | 467.65 | 421.62 | 0.00 | 0.00 | 972.16 | 561.94 | 70.18 | 733.99 | 0.00 | 0.00 | 0.00 | 0.00 | 0.00 | 0.00 | 0.00 |
| Q5VW32 | BRO1 domain-containing protein BROX | **only in pTa** | **0.01** | 90.44 | 81.56 | 0.00 | 0.00 | 26.36 | 0.00 | 79.68 | 156.64 | 189.55 | 0.00 | 0.00 | 0.00 | 0.00 | 0.00 | 0.00 |
| Q9NUU7 | ATP-dependent RNA helicase DDX19A | **only in pTa** | **0.01** | 19.59 | 15.62 | 0.00 | 0.00 | 35.81 | 0.00 | 17.16 | 10.13 | 34.87 | 0.00 | 0.00 | 0.00 | 0.00 | 0.00 | 0.00 |
| P07237 | Protein disulfide-isomerase | **1.58** | **0.01** | 1473.39 | 326.72 | 2331.89 | 542.73 | 1464.34 | 989.45 | 1386.00 | 1854.76 | 1672.41 | 1864.40 | 2965.06 | 1922.93 | 2530.26 | 1786.14 | 2922.56 |
| P61204 | ADP-ribosylation factor 3 | **1.45** | **0.01** | 1847.77 | 523.32 | 2685.00 | 346.76 | 1407.05 | 1231.12 | 2021.02 | 2513.55 | 2066.12 | 2604.93 | 3349.65 | 2616.61 | 2692.45 | 2348.30 | 2498.07 |
| P15531 | Nucleoside diphosphate kinase A | **1.50** | **0.01** | 2915.50 | 796.64 | 4372.50 | 751.59 | 2672.22 | 1807.38 | 3196.64 | 3998.18 | 2903.06 | 4324.58 | 5470.83 | 4772.44 | 3183.97 | 4308.60 | 4174.59 |
| P05164 | Myeloperoxidase | **only in pT2+** | **0.01** | 0.00 | 0.00 | 606.45 | 756.07 | 0.00 | 0.00 | 0.00 | 0.00 | 0.00 | 65.96 | 1636.13 | 1501.10 | 350.91 | 84.60 | 0.00 |
| P07099 | Epoxide hydrolase 1 | **only in pT2+** | **0.01** | 0.00 | 0.00 | 117.70 | 96.42 | 0.00 | 0.00 | 0.00 | 0.00 | 0.00 | 15.12 | 257.52 | 0.00 | 131.36 | 142.67 | 159.55 |
| P32455 | Interferon-induced guanylate-binding protein 1 | **only in pT2+** | **0.01** | 0.00 | 0.00 | 107.96 | 94.52 | 0.00 | 0.00 | 0.00 | 0.00 | 0.00 | 0.00 | 112.61 | 237.52 | 201.27 | 61.54 | 34.84 |
| P57088 | Transmembrane protein 33 | **only in pT2+** | **0.01** | 0.00 | 0.00 | 121.82 | 88.75 | 0.00 | 0.00 | 0.00 | 0.00 | 0.00 | 51.45 | 124.61 | 256.19 | 152.08 | 0.00 | 146.56 |
| Q32P28 | Prolyl 3-hydroxylase 1 | **only in pT2+** | **0.01** | 0.00 | 0.00 | 74.33 | 121.47 | 0.00 | 0.00 | 0.00 | 0.00 | 0.00 | 0.00 | 41.63 | 54.86 | 318.91 | 17.85 | 12.77 |
| Q9Y696 | Chloride intracellular channel protein 4 | **14.81** | **0.01** | 10.07 | 13.81 | 149.20 | 91.45 | 26.28 | 0.00 | 0.00 | 24.08 | 0.00 | 45.37 | 67.89 | 134.90 | 130.50 | 252.18 | 264.39 |
| P53621 | Coatomer subunit alpha | **5.07** | **0.01** | 33.74 | 50.69 | 171.22 | 87.96 | 6.77 | 0.00 | 43.46 | 118.47 | 0.00 | 84.21 | 218.76 | 216.27 | 302.54 | 92.58 | 112.94 |
| Q15582 | Transforming growth factor-beta-induced protein ig-h3 | **5.95** | **0.01** | 157.32 | 176.37 | 935.42 | 536.02 | 79.06 | 414.14 | 263.69 | 29.72 | 0.00 | 868.89 | 683.18 | 1363.73 | 1295.79 | 1387.79 | 13.14 |
| P30566 | Adenylosuccinate lyase | **3.29** | **0.01** | 23.68 | 33.40 | 77.86 | 25.87 | 15.73 | 0.00 | 0.00 | 80.90 | 21.75 | 111.09 | 41.21 | 88.72 | 66.98 | 97.70 | 61.45 |
| O75396 | Vesicle-trafficking protein SEC22b | **2.27** | **0.01** | 234.79 | 156.20 | 533.47 | 165.55 | 288.82 | 0.00 | 157.68 | 350.45 | 376.98 | 299.43 | 713.04 | 469.96 | 585.18 | 423.24 | 709.99 |
| P46776 | 60S ribosomal protein L27a | **1.93** | **0.01** | 487.26 | 348.23 | 938.72 | 112.90 | 318.51 | 0.00 | 781.09 | 851.70 | 484.98 | 825.55 | 1048.46 | 1036.57 | 920.21 | 786.57 | 1014.98 |
| Q13938 | Calcyphosin | **0.30** | **0.01** | 1212.91 | 410.23 | 366.63 | 496.37 | 1653.58 | 1213.10 | 695.16 | 928.92 | 1573.79 | 1265.36 | 486.00 | 448.45 | 0.00 | 0.00 | 0.00 |
| P15428 | 15-hydroxyprostaglandin dehydrogenase [NAD(+)] | **0.02** | **0.01** | 1065.64 | 561.98 | 22.41 | 36.03 | 1480.42 | 371.28 | 1587.26 | 552.36 | 1336.89 | 0.00 | 82.46 | 52.00 | 0.00 | 0.00 | 0.00 |
| P46940 | Ras GTPase-activating-like protein IQGAP1 | **2.13** | **0.02** | 398.71 | 213.92 | 849.53 | 275.29 | 381.88 | 77.16 | 460.57 | 673.54 | 400.39 | 1220.20 | 1058.70 | 692.70 | 601.79 | 542.29 | 981.50 |
| P08758 | Annexin A5 | **2.12** | **0.02** | 938.73 | 285.83 | 1988.45 | 724.54 | 721.59 | 1310.20 | 935.34 | 608.04 | 1118.46 | 1522.95 | 1590.60 | 3145.11 | 2599.16 | 1791.78 | 1281.12 |
| Q15365 | Poly(rC)-binding protein 1 | **1.37** | **0.02** | 926.08 | 181.23 | 1268.56 | 192.89 | 867.67 | 742.63 | 1229.39 | 909.86 | 880.85 | 1499.26 | 1110.72 | 1411.87 | 1114.12 | 1062.20 | 1413.18 |
| Q96A26 | Protein FAM162A | **0.33** | **0.02** | 639.64 | 210.30 | 211.12 | 257.04 | 904.29 | 463.87 | 413.91 | 796.53 | 619.61 | 625.01 | 295.97 | 0.00 | 0.00 | 0.00 | 345.76 |
| P21266 | Glutathione S-transferase Mu 3 | **0.20** | **0.02** | 3012.40 | 1820.08 | 616.89 | 671.34 | 2373.76 | 123.27 | 4343.00 | 4505.68 | 3716.29 | 0.00 | 1679.08 | 216.85 | 0.00 | 759.99 | 1045.40 |
| O43707 | Alpha-actinin-4 | **1.67** | **0.02** | 1178.63 | 562.56 | 1963.55 | 299.48 | 1289.16 | 367.79 | 1865.63 | 1434.45 | 936.12 | 1427.30 | 1954.80 | 2304.90 | 1893.60 | 2131.47 | 2069.25 |
| P09488 | Glutathione S-transferase Mu 1 | **0.28** | **0.02** | 4270.30 | 2015.59 | 1204.34 | 1432.05 | 4207.85 | 804.41 | 5634.00 | 5478.51 | 5226.71 | 0.00 | 3525.97 | 1449.36 | 63.17 | 2100.44 | 87.10 |
| P08134 | Rho-related GTP-binding protein RhoC | **7.19** | **0.02** | 143.39 | 320.63 | 1031.05 | 638.00 | 0.00 | 0.00 | 0.00 | 0.00 | 716.95 | 650.82 | 1549.79 | 928.52 | 0.00 | 1365.94 | 1691.24 |
| P12004 | Proliferating cell nuclear antigen | **3.68** | **0.02** | 180.22 | 181.29 | 663.17 | 342.36 | 61.44 | 0.00 | 335.53 | 410.86 | 93.25 | 354.63 | 1151.56 | 556.60 | 244.70 | 779.74 | 891.83 |
| P05787 | Keratin, type II cytoskeletal 8 | **0.56** | **0.02** | 4729.80 | 536.18 | 2656.88 | 1560.98 | 4583.51 | 4806.65 | 5395.28 | 4932.84 | 3930.70 | 5202.15 | 2799.16 | 3166.49 | 963.06 | 2749.56 | 1060.85 |
| Q96CX2 | BTB/POZ domain-containing protein KCTD12 | **15.88** | **0.02** | 16.84 | 37.66 | 267.52 | 186.81 | 0.00 | 0.00 | 84.22 | 0.00 | 0.00 | 148.35 | 176.63 | 372.54 | 450.98 | 456.65 | 0.00 |
| P62263 | 40S ribosomal protein S14 | **2.14** | **0.02** | 590.29 | 431.78 | 1262.35 | 366.70 | 299.74 | 0.00 | 735.06 | 1060.81 | 855.84 | 1476.10 | 1480.00 | 1341.28 | 590.38 | 1110.33 | 1576.02 |
| P46781 | 40S ribosomal protein S9 | **1.64** | **0.02** | 548.83 | 262.15 | 899.45 | 149.22 | 311.36 | 299.29 | 496.30 | 881.54 | 755.65 | 766.65 | 1153.02 | 759.96 | 981.31 | 842.83 | 892.96 |
| P04844 | Dolichyl-diphosphooligosaccharide--protein glycosyltransferase subunit 2 | **2.41** | **0.02** | 241.79 | 180.11 | 582.33 | 221.74 | 193.46 | 0.00 | 237.21 | 502.09 | 276.19 | 351.00 | 776.12 | 511.55 | 914.60 | 385.88 | 554.83 |
| P07738 | Bisphosphoglycerate mutase | **0.13** | **0.02** | 39.93 | 27.50 | 5.37 | 13.16 | 69.65 | 0.00 | 29.66 | 60.94 | 39.42 | 0.00 | 0.00 | 0.00 | 0.00 | 32.24 | 0.00 |
| P26038 | Moesin | **1.73** | **0.02** | 711.80 | 178.97 | 1228.42 | 386.10 | 648.49 | 915.80 | 649.37 | 867.02 | 478.33 | 784.38 | 1234.09 | 1692.74 | 1575.47 | 1308.88 | 774.93 |
| Q03013 | Glutathione S-transferase Mu 4 | **0.18** | **0.03** | 2457.44 | 1402.42 | 445.78 | 1091.94 | 3487.18 | 0.00 | 3121.77 | 2971.54 | 2706.69 | 0.00 | 2674.69 | 0.00 | 0.00 | 0.00 | 0.00 |
| Q9NYL9 | Tropomodulin-3 | **0.34** | **0.03** | 75.92 | 38.49 | 26.11 | 22.60 | 142.38 | 70.48 | 45.41 | 67.15 | 54.20 | 23.43 | 0.00 | 0.00 | 35.16 | 54.09 | 43.97 |
| P62899 | 60S ribosomal protein L31 | **3.17** | **0.03** | 148.09 | 168.97 | 469.01 | 219.20 | 212.58 | 0.00 | 0.00 | 123.69 | 404.20 | 367.32 | 874.70 | 459.97 | 221.84 | 483.81 | 406.45 |
| P29034 | Protein S100-A2 | **0.22** | **0.03** | 1999.99 | 1265.52 | 444.32 | 629.24 | 3651.02 | 1116.42 | 413.98 | 2252.49 | 2566.04 | 0.00 | 683.23 | 1596.28 | 0.00 | 0.00 | 386.42 |
| Q9Y678 | Coatomer subunit gamma-1 | **6.26** | **0.03** | 21.50 | 37.97 | 134.62 | 89.12 | 0.00 | 0.00 | 19.86 | 87.66 | 0.00 | 0.00 | 242.05 | 100.76 | 211.42 | 164.68 | 88.79 |
| P25787 | Proteasome subunit alpha type-2 | **2.68** | **0.03** | 88.16 | 100.60 | 236.12 | 85.29 | 199.55 | 0.00 | 48.45 | 192.80 | 0.00 | 317.86 | 119.19 | 166.80 | 238.31 | 232.64 | 341.95 |
| Q9UHQ9 | NADH-cytochrome b5 reductase 1 | **0.11** | **0.03** | 317.68 | 259.73 | 35.92 | 40.94 | 234.76 | 0.00 | 719.64 | 332.34 | 301.64 | 89.40 | 72.45 | 0.00 | 0.00 | 0.00 | 53.66 |
| P08729 | Keratin, type II cytoskeletal 7 | **0.58** | **0.03** | 4516.81 | 1151.58 | 2626.10 | 1211.53 | 4936.88 | 4932.20 | 2524.07 | 5492.23 | 4698.66 | 3334.66 | 1846.69 | 4187.03 | 872.28 | 3342.06 | 2173.86 |
| O00264 | Membrane-associated progesterone receptor component 1 | **0.37** | **0.03** | 730.43 | 347.30 | 270.36 | 227.02 | 1243.41 | 399.08 | 813.13 | 782.32 | 414.19 | 483.42 | 575.57 | 69.88 | 0.00 | 295.25 | 198.05 |
| Q9Y3Z3 | Deoxynucleoside triphosphate triphosphohydrolase SAMHD1 | **104.33** | **0.03** | 1.74 | 3.90 | 181.86 | 143.54 | 0.00 | 0.00 | 8.72 | 0.00 | 0.00 | 25.70 | 260.56 | 282.86 | 352.20 | 169.82 | 0.00 |
| P61019 | Ras-related protein Rab-2A | **1.76** | **0.03** | 419.62 | 266.74 | 740.42 | 131.72 | 415.23 | 69.50 | 441.25 | 816.41 | 355.68 | 609.86 | 726.13 | 619.36 | 817.93 | 709.91 | 959.31 |
| P12814 | Alpha-actinin-1 | **3.18** | **0.03** | 686.07 | 436.64 | 2184.42 | 1222.34 | 983.50 | 0.00 | 1113.93 | 765.52 | 567.42 | 935.56 | 1739.70 | 4042.12 | 3259.51 | 1976.15 | 1153.46 |
| P62269 | 40S ribosomal protein S18 | **1.46** | **0.03** | 1061.00 | 361.43 | 1548.75 | 264.81 | 813.84 | 590.02 | 1361.84 | 1445.78 | 1093.51 | 1290.45 | 1950.12 | 1713.77 | 1282.50 | 1636.94 | 1418.74 |
| P14174 | Macrophage migration inhibitory factor | **0.40** | **0.03** | 3859.42 | 1928.94 | 1554.84 | 956.12 | 6393.93 | 4678.96 | 1881.59 | 1961.15 | 4381.49 | 2651.57 | 950.18 | 1820.73 | 2266.86 | 1639.72 | 0.00 |
| P26447 | Protein S100-A4 | **0.36** | **0.03** | 4852.81 | 2654.85 | 1723.48 | 1260.39 | 9541.79 | 4200.01 | 3820.31 | 3032.34 | 3669.61 | 706.53 | 1562.44 | 2851.78 | 1687.77 | 3440.21 | 92.13 |
| Q8TCJ2 | Dolichyl-diphosphooligosaccharide--protein glycosyltransferase subunit STT3B | **5.41** | **0.03** | 13.21 | 29.54 | 71.47 | 43.26 | 0.00 | 0.00 | 0.00 | 66.06 | 0.00 | 55.10 | 131.31 | 57.79 | 26.27 | 39.43 | 118.93 |
| O43169 | Cytochrome b5 type B | **0.17** | **0.03** | 654.43 | 487.51 | 108.59 | 184.68 | 1356.22 | 0.00 | 529.05 | 767.97 | 618.89 | 0.00 | 446.25 | 0.00 | 0.00 | 205.28 | 0.00 |
| P35580 | Myosin-10 | **2.75** | **0.03** | 182.45 | 113.79 | 501.53 | 258.90 | 196.47 | 164.50 | 293.28 | 257.98 | 0.00 | 203.70 | 479.78 | 861.81 | 733.66 | 477.08 | 253.16 |
| P02792 | Ferritin light chain | **9.69** | **0.03** | 157.71 | 161.72 | 1528.47 | 1158.81 | 119.34 | 415.31 | 56.15 | 0.00 | 197.75 | 1115.20 | 2729.38 | 2528.24 | 2354.31 | 272.45 | 171.25 |
| P08779 | Keratin, type I cytoskeletal 16 | **0.40** | **0.03** | 920.41 | 290.95 | 367.92 | 409.40 | 633.51 | 930.59 | 751.49 | 889.87 | 1396.58 | 0.00 | 800.19 | 0.00 | 0.00 | 604.51 | 802.81 |
| O00442 | RNA 3'-terminal phosphate cyclase | **only in pT2+** | **0.03** | 0.00 | 0.00 | 26.29 | 21.54 | 0.00 | 0.00 | 0.00 | 0.00 | 0.00 | 0.00 | 35.99 | 47.56 | 45.97 | 0.00 | 28.25 |
| O94832 | Unconventional myosin-Id | **only in pT2+** | **0.03** | 0.00 | 0.00 | 6.98 | 7.01 | 0.00 | 0.00 | 0.00 | 0.00 | 0.00 | 6.60 | 18.34 | 0.00 | 11.16 | 0.00 | 5.77 |
| P00533 | Epidermal growth factor receptor | **only in pT2+** | **0.03** | 0.00 | 0.00 | 20.06 | 18.57 | 0.00 | 0.00 | 0.00 | 0.00 | 0.00 | 43.06 | 19.64 | 0.00 | 0.00 | 17.97 | 39.69 |
| P05546 | Heparin cofactor 2 | **only in pT2+** | **0.03** | 0.00 | 0.00 | 128.75 | 220.32 | 0.00 | 0.00 | 0.00 | 0.00 | 0.00 | 113.42 | 25.19 | 569.69 | 64.18 | 0.00 | 0.00 |
| P08195 | 4F2 cell-surface antigen heavy chain | **only in pT2+** | **0.03** | 0.00 | 0.00 | 10.10 | 8.23 | 0.00 | 0.00 | 0.00 | 0.00 | 0.00 | 17.78 | 13.20 | 0.00 | 0.00 | 18.12 | 11.52 |
| P11172 | Uridine 5'-monophosphate synthase | **only in pT2+** | **0.03** | 0.00 | 0.00 | 21.97 | 20.91 | 0.00 | 0.00 | 0.00 | 0.00 | 0.00 | 38.14 | 21.07 | 0.00 | 0.00 | 19.73 | 52.86 |
| P15924 | Desmoplakin | **only in pT2+** | **0.03** | 0.00 | 0.00 | 202.41 | 271.34 | 0.00 | 0.00 | 0.00 | 0.00 | 0.00 | 392.91 | 148.51 | 0.00 | 11.69 | 0.00 | 661.33 |
| P21283 | V-type proton ATPase subunit C 1 | **only in pT2+** | **0.03** | 0.00 | 0.00 | 14.57 | 15.45 | 0.00 | 0.00 | 0.00 | 0.00 | 0.00 | 0.00 | 12.10 | 41.47 | 20.62 | 13.25 | 0.00 |
| P27694 | Replication protein A 70 kDa DNA-binding subunit | **only in pT2+** | **0.03** | 0.00 | 0.00 | 22.64 | 21.29 | 0.00 | 0.00 | 0.00 | 0.00 | 0.00 | 34.28 | 41.41 | 0.00 | 0.00 | 12.13 | 48.01 |
| P33993 | DNA replication licensing factor MCM7 | **only in pT2+** | **0.03** | 0.00 | 0.00 | 19.93 | 17.79 | 0.00 | 0.00 | 0.00 | 0.00 | 0.00 | 39.28 | 30.47 | 0.00 | 0.00 | 13.69 | 36.14 |
| P40261 | Nicotinamide N-methyltransferase | **only in pT2+** | **0.03** | 0.00 | 0.00 | 508.18 | 632.44 | 0.00 | 0.00 | 0.00 | 0.00 | 0.00 | 0.00 | 225.30 | 1336.47 | 1294.31 | 193.03 | 0.00 |
| P49736 | DNA replication licensing factor MCM2 | **only in pT2+** | **0.03** | 0.00 | 0.00 | 25.29 | 22.88 | 0.00 | 0.00 | 0.00 | 0.00 | 0.00 | 22.66 | 26.99 | 0.00 | 0.00 | 49.83 | 52.25 |
| P63267 | Actin, gamma-enteric smooth muscle | **only in pT2+** | **0.03** | 0.00 | 0.00 | 7666.83 | 7391.49 | 0.00 | 0.00 | 0.00 | 0.00 | 0.00 | 0.00 | 6683.72 | 19001.01 | 12781.74 | 7534.53 | 0.00 |
| P78344 | Eukaryotic translation initiation factor 4 gamma 2 | **only in pT2+** | **0.03** | 0.00 | 0.00 | 14.67 | 18.47 | 0.00 | 0.00 | 0.00 | 0.00 | 0.00 | 0.00 | 48.45 | 10.64 | 0.00 | 22.10 | 6.80 |
| P98095 | Fibulin-2 | **only in pT2+** | **0.03** | 0.00 | 0.00 | 76.33 | 140.65 | 0.00 | 0.00 | 0.00 | 0.00 | 0.00 | 11.55 | 0.00 | 75.56 | 357.55 | 13.29 | 0.00 |
| Q06828 | Fibromodulin | **only in pT2+** | **0.03** | 0.00 | 0.00 | 155.40 | 211.17 | 0.00 | 0.00 | 0.00 | 0.00 | 0.00 | 0.00 | 165.98 | 565.39 | 115.29 | 85.77 | 0.00 |
| Q14194 | Dihydropyrimidinase-related protein 1 | **only in pT2+** | **0.03** | 0.00 | 0.00 | 182.21 | 179.47 | 0.00 | 0.00 | 0.00 | 0.00 | 0.00 | 0.00 | 126.35 | 388.63 | 403.25 | 175.00 | 0.00 |
| Q15746 | Myosin light chain kinase, smooth muscle | **only in pT2+** | **0.03** | 0.00 | 0.00 | 82.23 | 83.41 | 0.00 | 0.00 | 0.00 | 0.00 | 0.00 | 0.00 | 54.07 | 213.85 | 138.07 | 87.38 | 0.00 |
| Q15858 | Sodium channel protein type 9 subunit alpha | **only in pT2+** | **0.03** | 0.00 | 0.00 | 22.70 | 18.66 | 0.00 | 0.00 | 0.00 | 0.00 | 0.00 | 23.93 | 32.43 | 36.69 | 43.17 | 0.00 | 0.00 |
| Q6DD88 | Atlastin-3 | **only in pT2+** | **0.03** | 0.00 | 0.00 | 70.12 | 76.51 | 0.00 | 0.00 | 0.00 | 0.00 | 0.00 | 0.00 | 199.89 | 73.85 | 109.26 | 0.00 | 37.71 |
| Q86UX7 | Fermitin family homolog 3 | **only in pT2+** | **0.03** | 0.00 | 0.00 | 19.75 | 21.51 | 0.00 | 0.00 | 0.00 | 0.00 | 0.00 | 8.99 | 48.95 | 43.39 | 17.18 | 0.00 | 0.00 |
| Q96DI7 | U5 small nuclear ribonucleoprotein 40 kDa protein | **only in pT2+** | **0.03** | 0.00 | 0.00 | 18.20 | 14.14 | 0.00 | 0.00 | 0.00 | 0.00 | 0.00 | 26.75 | 28.65 | 0.00 | 0.00 | 28.33 | 25.47 |
| Q96SI9 | Spermatid perinuclear RNA-binding protein | **only in pT2+** | **0.03** | 0.00 | 0.00 | 97.07 | 80.82 | 0.00 | 0.00 | 0.00 | 0.00 | 0.00 | 107.11 | 191.30 | 0.00 | 122.10 | 161.92 | 0.00 |
| Q9HC07 | Transmembrane protein 165 | **only in pT2+** | **0.03** | 0.00 | 0.00 | 48.08 | 42.08 | 0.00 | 0.00 | 0.00 | 0.00 | 0.00 | 40.55 | 101.61 | 0.00 | 0.00 | 68.21 | 78.13 |
| Q9NUV9 | GTPase IMAP family member 4 | **only in pT2+** | **0.03** | 0.00 | 0.00 | 39.76 | 36.05 | 0.00 | 0.00 | 0.00 | 0.00 | 0.00 | 0.00 | 35.99 | 47.66 | 91.94 | 62.95 | 0.00 |
| Q9NZU5 | LIM and cysteine-rich domains protein 1 | **only in pT2+** | **0.03** | 0.00 | 0.00 | 46.94 | 65.31 | 0.00 | 0.00 | 0.00 | 0.00 | 0.00 | 0.00 | 20.94 | 79.12 | 166.16 | 15.42 | 0.00 |
| O60763 | General vesicular transport factor p115 | **2.09** | **0.04** | 113.00 | 68.39 | 236.20 | 91.96 | 99.51 | 0.00 | 170.83 | 152.61 | 142.07 | 167.65 | 245.68 | 283.37 | 372.45 | 241.02 | 107.02 |
| Q13404 | Ubiquitin-conjugating enzyme E2 variant 1 | **1.54** | **0.04** | 450.98 | 163.04 | 694.11 | 160.16 | 313.73 | 276.19 | 527.56 | 676.76 | 460.68 | 557.58 | 885.18 | 656.35 | 776.90 | 817.32 | 471.30 |
| Q8WUY1 | Protein THEM6 | **0.20** | **0.04** | 270.35 | 207.94 | 55.19 | 53.01 | 181.77 | 0.00 | 570.57 | 308.78 | 290.61 | 27.50 | 85.17 | 0.00 | 0.00 | 124.26 | 94.19 |
| P50395 | Rab GDP dissociation inhibitor beta | **1.48** | **0.04** | 810.20 | 364.43 | 1201.07 | 133.76 | 862.19 | 278.51 | 1159.48 | 1115.55 | 635.29 | 1023.63 | 1373.26 | 1221.92 | 1139.54 | 1331.40 | 1116.70 |
| O15327 | Type II inositol 3,4-bisphosphate 4-phosphatase | **only in pTa** | **0.04** | 9.57 | 9.09 | 0.00 | 0.00 | 18.95 | 0.00 | 12.03 | 16.89 | 0.00 | 0.00 | 0.00 | 0.00 | 0.00 | 0.00 | 0.00 |
| O60840 | Voltage-dependent L-type calcium channel subunit alpha-1F | **only in pTa** | **0.04** | 18.19 | 17.09 | 0.00 | 0.00 | 27.93 | 26.19 | 0.00 | 0.00 | 36.83 | 0.00 | 0.00 | 0.00 | 0.00 | 0.00 | 0.00 |
| P02549 | Spectrin alpha chain, erythrocytic 1 | **only in pTa** | **0.04** | 37.62 | 81.24 | 0.00 | 0.00 | 3.02 | 0.00 | 2.15 | 0.00 | 182.92 | 0.00 | 0.00 | 0.00 | 0.00 | 0.00 | 0.00 |
| P05026 | Sodium/potassium-transporting ATPase subunit beta-1 | **only in pTa** | **0.04** | 46.53 | 80.14 | 0.00 | 0.00 | 188.53 | 0.00 | 22.82 | 21.31 | 0.00 | 0.00 | 0.00 | 0.00 | 0.00 | 0.00 | 0.00 |
| P09110 | 3-ketoacyl-CoA thiolase, peroxisomal | **only in pTa** | **0.04** | 41.21 | 52.47 | 0.00 | 0.00 | 49.67 | 0.00 | 0.00 | 127.22 | 29.17 | 0.00 | 0.00 | 0.00 | 0.00 | 0.00 | 0.00 |
| P12931 | Proto-oncogene tyrosine-protein kinase Src | **only in pTa** | **0.04** | 95.78 | 125.37 | 0.00 | 0.00 | 0.00 | 0.00 | 111.43 | 303.75 | 63.71 | 0.00 | 0.00 | 0.00 | 0.00 | 0.00 | 0.00 |
| P23229 | Integrin alpha-6 | **only in pTa** | **0.04** | 31.93 | 35.47 | 0.00 | 0.00 | 33.11 | 0.00 | 40.60 | 85.93 | 0.00 | 0.00 | 0.00 | 0.00 | 0.00 | 0.00 | 0.00 |
| P29144 | Tripeptidyl-peptidase 2 | **only in pTa** | **0.04** | 6.37 | 6.66 | 0.00 | 0.00 | 15.85 | 0.00 | 0.00 | 7.31 | 8.72 | 0.00 | 0.00 | 0.00 | 0.00 | 0.00 | 0.00 |
| P35573 | Glycogen debranching enzyme | **only in pTa** | **0.04** | 3.92 | 4.59 | 0.00 | 0.00 | 3.46 | 0.00 | 5.00 | 11.14 | 0.00 | 0.00 | 0.00 | 0.00 | 0.00 | 0.00 | 0.00 |
| P55290 | Cadherin-13 | **only in pTa** | **0.04** | 12.27 | 11.83 | 0.00 | 0.00 | 17.47 | 26.67 | 17.23 | 0.00 | 0.00 | 0.00 | 0.00 | 0.00 | 0.00 | 0.00 | 0.00 |
| Q01740 | Dimethylaniline monooxygenase [N-oxide-forming] 1 | **only in pTa** | **0.04** | 49.64 | 48.94 | 0.00 | 0.00 | 66.79 | 112.88 | 0.00 | 0.00 | 68.53 | 0.00 | 0.00 | 0.00 | 0.00 | 0.00 | 0.00 |
| Q14CN2 | Calcium-activated chloride channel regulator 4 | **only in pTa** | **0.04** | 139.68 | 264.76 | 0.00 | 0.00 | 39.25 | 0.00 | 611.67 | 0.00 | 47.47 | 0.00 | 0.00 | 0.00 | 0.00 | 0.00 | 0.00 |
| Q4VC31 | Coiled-coil domain-containing protein 58 | **only in pTa** | **0.04** | 80.55 | 148.52 | 0.00 | 0.00 | 344.84 | 0.00 | 35.58 | 22.35 | 0.00 | 0.00 | 0.00 | 0.00 | 0.00 | 0.00 | 0.00 |
| Q8WXH0 | Nesprin-2 | **only in pTa** | **0.04** | 2.42 | 3.23 | 0.00 | 0.00 | 3.56 | 7.58 | 0.00 | 0.96 | 0.00 | 0.00 | 0.00 | 0.00 | 0.00 | 0.00 | 0.00 |
| Q9H8M5 | Metal transporter CNNM2 | **only in pTa** | **0.04** | 55.04 | 53.58 | 0.00 | 0.00 | 79.22 | 121.97 | 0.00 | 0.00 | 74.02 | 0.00 | 0.00 | 0.00 | 0.00 | 0.00 | 0.00 |
| Q9UJ72 | Annexin A10 | **only in pTa** | **0.04** | 291.91 | 409.26 | 0.00 | 0.00 | 448.24 | 0.00 | 67.64 | 943.68 | 0.00 | 0.00 | 0.00 | 0.00 | 0.00 | 0.00 | 0.00 |
| O60749 | Sorting nexin-2 | **2.22** | **0.04** | 71.68 | 56.29 | 159.36 | 61.29 | 103.23 | 23.98 | 101.27 | 129.90 | 0.00 | 196.22 | 127.41 | 167.93 | 244.02 | 156.89 | 63.68 |
| Q8TB22 | Spermatogenesis-associated protein 20 | **0.21** | **0.04** | 25.76 | 18.37 | 5.41 | 8.44 | 17.49 | 25.62 | 0.00 | 38.70 | 46.98 | 0.00 | 0.00 | 0.00 | 0.00 | 14.68 | 17.80 |
| Q9BRA2 | Thioredoxin domain-containing protein 17 | **0.38** | **0.04** | 338.75 | 159.61 | 127.85 | 129.42 | 192.71 | 174.12 | 388.91 | 561.26 | 376.78 | 59.14 | 0.00 | 0.00 | 153.99 | 301.26 | 252.68 |
| O15371 | Eukaryotic translation initiation factor 3 subunit D | **3.23** | **0.04** | 27.54 | 44.77 | 88.99 | 39.63 | 0.00 | 0.00 | 34.75 | 102.96 | 0.00 | 54.50 | 44.90 | 147.97 | 114.00 | 103.64 | 68.93 |
| P15374 | Ubiquitin carboxyl-terminal hydrolase isozyme L3 | **0.20** | **0.04** | 227.03 | 139.74 | 45.00 | 110.22 | 376.55 | 0.00 | 277.03 | 266.53 | 215.03 | 0.00 | 0.00 | 0.00 | 0.00 | 0.00 | 269.98 |
| P62277 | 40S ribosomal protein S13 | **2.02** | **0.04** | 475.67 | 368.58 | 963.00 | 132.57 | 269.34 | 155.05 | 802.65 | 943.84 | 207.44 | 989.72 | 1066.17 | 961.61 | 716.37 | 1088.07 | 956.07 |
| P30626 | Sorcin | **0.51** | **0.04** | 1442.45 | 701.88 | 736.39 | 154.67 | 2649.66 | 1386.36 | 1127.92 | 849.27 | 1199.05 | 777.47 | 925.68 | 488.10 | 787.83 | 626.80 | 812.48 |
| P04066 | Tissue alpha-L-fucosidase | **0.31** | **0.04** | 106.26 | 40.39 | 33.32 | 56.30 | 84.53 | 159.96 | 85.37 | 137.28 | 64.17 | 0.00 | 135.49 | 0.00 | 0.00 | 64.46 | 0.00 |
| O95782 | AP-2 complex subunit alpha-1 | **3.87** | **0.04** | 27.91 | 47.98 | 108.11 | 61.09 | 0.00 | 0.00 | 28.78 | 110.79 | 0.00 | 50.71 | 217.36 | 127.35 | 107.91 | 89.20 | 56.11 |
| O15144 | Actin-related protein 2/3 complex subunit 2 | **1.94** | **0.04** | 438.81 | 341.19 | 850.64 | 232.87 | 505.07 | 0.00 | 704.04 | 802.80 | 182.16 | 578.75 | 1181.28 | 648.82 | 795.92 | 1060.43 | 838.61 |
| P19971 | Thymidine phosphorylase | **14.94** | **0.04** | 52.17 | 85.54 | 779.22 | 673.84 | 63.91 | 196.96 | 0.00 | 0.00 | 0.00 | 66.24 | 499.73 | 797.44 | 2048.22 | 510.00 | 753.67 |
| O14773 | Tripeptidyl-peptidase 1 | **2.93** | **0.04** | 79.93 | 66.51 | 233.96 | 131.03 | 165.87 | 108.87 | 97.60 | 27.31 | 0.00 | 0.00 | 286.64 | 304.74 | 313.53 | 340.29 | 158.57 |
| P25205 | DNA replication licensing factor MCM3 | **13.95** | **0.04** | 3.03 | 4.30 | 42.22 | 35.86 | 0.00 | 0.00 | 5.92 | 9.21 | 0.00 | 42.66 | 63.49 | 0.00 | 0.00 | 58.63 | 88.51 |
| P00846 | ATP synthase subunit a | **2.39** | **0.04** | 271.35 | 258.32 | 649.65 | 273.14 | 392.54 | 0.00 | 392.26 | 571.94 | 0.00 | 609.54 | 982.14 | 965.76 | 305.32 | 456.39 | 578.76 |
| O95336 | 6-phosphogluconolactonase | **0.45** | **0.04** | 534.47 | 261.56 | 240.60 | 151.60 | 769.08 | 154.40 | 646.63 | 725.82 | 376.44 | 261.05 | 180.83 | 173.68 | 346.27 | 459.16 | 22.63 |
| P28838 | Cytosol aminopeptidase | **4.06** | **0.05** | 106.90 | 83.70 | 433.75 | 301.60 | 206.22 | 45.38 | 187.83 | 27.80 | 67.28 | 122.17 | 345.47 | 527.30 | 951.69 | 486.87 | 168.98 |
| Q15847 | Adipogenesis regulatory factor | **0.15** | **0.05** | 1944.31 | 1303.66 | 297.16 | 460.54 | 3657.69 | 1410.76 | 2963.93 | 600.28 | 1088.89 | 0.00 | 0.00 | 0.00 | 0.00 | 870.86 | 912.09 |
| Q9UJZ1 | Stomatin-like protein 2, mitochondrial | **3.26** | **0.05** | 25.89 | 36.98 | 84.39 | 45.40 | 0.00 | 0.00 | 79.62 | 49.82 | 0.00 | 26.57 | 134.88 | 35.60 | 80.31 | 124.54 | 104.46 |
| P35268 | 60S ribosomal protein L22 | **1.51** | **0.05** | 515.08 | 182.72 | 779.58 | 194.25 | 457.64 | 326.96 | 374.09 | 719.84 | 696.89 | 632.61 | 1035.67 | 992.89 | 577.72 | 782.41 | 656.18 |
| P23284 | Peptidyl-prolyl cis-trans isomerase B | **1.41** | **0.05** | 1824.06 | 457.60 | 2565.08 | 578.78 | 1386.83 | 1764.97 | 1892.69 | 2560.76 | 1515.03 | 2906.85 | 1860.76 | 2894.39 | 3373.20 | 2164.84 | 2190.43 |
| P23381 | Tryptophan--tRNA ligase, cytoplasmic | **11.29** | **0.05** | 45.16 | 66.04 | 510.00 | 435.07 | 0.00 | 0.00 | 145.69 | 80.09 | 0.00 | 43.80 | 235.72 | 1173.86 | 601.85 | 822.23 | 182.55 |
| Q15063 | Periostin | **18.61** | **0.05** | 79.63 | 97.81 | 1482.03 | 1313.98 | 57.73 | 59.53 | 249.18 | 31.72 | 0.00 | 682.35 | 1168.76 | 3269.23 | 2929.55 | 842.28 | 0.00 |
| P13797 | Plastin-3 | **2.03** | **0.05** | 410.11 | 169.05 | 833.57 | 379.50 | 449.54 | 285.17 | 428.00 | 661.34 | 226.51 | 808.76 | 1109.83 | 796.03 | 1333.49 | 738.43 | 214.88 |
| Q00325 | Phosphate carrier protein, mitochondrial | **1.73** | **0.05** | 453.34 | 294.26 | 782.38 | 181.10 | 382.97 | 35.62 | 724.50 | 748.69 | 374.91 | 935.90 | 945.48 | 756.71 | 517.30 | 623.58 | 915.29 |
| P30740 | Leukocyte elastase inhibitor | **10.33** | **0.05** | 10.16 | 22.72 | 104.93 | 89.73 | 50.80 | 0.00 | 0.00 | 0.00 | 0.00 | 52.99 | 189.84 | 0.00 | 158.39 | 204.50 | 23.88 |
| O00232 | 26S proteasome non-ATPase regulatory subunit 12 | **5.28** | **0.05** | 10.99 | 15.97 | 58.09 | 43.60 | 0.00 | 0.00 | 0.00 | 35.02 | 19.94 | 17.91 | 137.40 | 24.07 | 56.81 | 41.43 | 70.94 |
| P42224 | Signal transducer and activator of transcription 1-alpha/beta | **15.71** | **0.05** | 19.32 | 20.64 | 303.64 | 268.61 | 15.69 | 0.00 | 35.45 | 45.48 | 0.00 | 0.00 | 312.33 | 568.56 | 664.55 | 219.73 | 56.66 |
| P08133 | Annexin A6 | **5.77** | **0.05** | 192.02 | 196.50 | 1108.08 | 873.65 | 80.69 | 337.41 | 463.59 | 54.39 | 24.01 | 322.67 | 896.42 | 2325.82 | 1961.81 | 998.50 | 143.25 |
| Q16851 | UTP--glucose-1-phosphate uridylyltransferase | **2.00** | **0.05** | 122.37 | 90.37 | 245.18 | 88.86 | 242.09 | 19.47 | 47.31 | 137.09 | 165.90 | 150.59 | 358.61 | 341.41 | 254.34 | 165.56 | 200.58 |
| P16402 | Histone H1.3 | **1.42** | **0.05** | 3343.15 | 1196.89 | 4763.70 | 888.03 | 2719.80 | 5459.84 | 2789.19 | 3111.31 | 2635.63 | 5810.41 | 5239.45 | 5096.19 | 3831.45 | 3522.68 | 5081.98 |
| Q9Y295 | Developmentally-regulated GTP-binding protein 1 | **10.44** | **0.05** | 3.86 | 8.62 | 40.25 | 34.92 | 0.00 | 0.00 | 0.00 | 19.28 | 0.00 | 0.00 | 77.68 | 0.00 | 31.93 | 71.73 | 60.16 |
| P30050 | 60S ribosomal protein L12 | **1.61** | **0.05** | 884.57 | 361.13 | 1423.65 | 420.23 | 541.39 | 530.76 | 1206.96 | 1300.57 | 843.16 | 867.44 | 2065.62 | 1521.63 | 1318.51 | 1121.93 | 1646.78 |
| Q5XKE5 | Keratin, type II cytoskeletal 79 | **0.58** | **0.05** | 606.42 | 108.29 | 350.79 | 233.10 | 747.34 | 548.03 | 462.77 | 660.77 | 613.18 | 552.76 | 360.18 | 639.46 | 194.71 | 0.00 | 357.65 |
| P36776 | Lon protease homolog, mitochondrial | **7.53** | **0.05** | 4.31 | 9.63 | 32.42 | 27.14 | 0.00 | 0.00 | 0.00 | 21.54 | 0.00 | 0.00 | 42.27 | 34.77 | 0.00 | 52.04 | 65.47 |
| Q562R1 | Beta-actin-like protein 2 | **1.86** | **0.05** | 2235.29 | 1301.99 | 4164.88 | 1523.18 | 2460.63 | 2843.13 | 2499.90 | 3372.78 | 0.00 | 3097.73 | 3435.16 | 5843.35 | 6324.21 | 3572.49 | 2716.37 |
| P0C0S5 | Histone H2A.Z | **1.43** | **0.05** | 4975.96 | 1641.72 | 7129.52 | 1553.70 | 3787.18 | 7375.70 | 5186.89 | 5394.99 | 3135.04 | 9112.14 | 7762.08 | 7906.57 | 4836.21 | 5782.53 | 7377.59 |
| P06703 | Protein S100-A6 | **0.68** | **0.05** | 4379.19 | 782.76 | 2959.60 | 1229.12 | 4498.61 | 3830.03 | 3948.29 | 5698.04 | 3920.99 | 4295.74 | 3361.07 | 2118.98 | 1679.02 | 1889.04 | 4413.77 |
| Q96A19 | Coiled-coil domain-containing protein 102A | **0.16** | **0.05** | 423.18 | 297.50 | 65.73 | 85.16 | 530.62 | 543.48 | 216.16 | 35.38 | 790.28 | 214.50 | 90.36 | 0.00 | 0.00 | 89.51 | 0.00 |
| O14818 | Proteasome subunit alpha type-7 | **1.76** | **0.05** | 223.23 | 128.83 | 392.69 | 124.76 | 277.84 | 0.00 | 272.54 | 327.77 | 237.99 | 216.04 | 428.71 | 452.10 | 273.51 | 554.18 | 431.62 |
| P06899 | Histone H2B type 1-J | **1.48** | **0.05** | 6050.84 | 1711.75 | 8961.66 | 2471.38 | 4464.21 | 4995.48 | 7370.44 | 8366.34 | 5057.72 | 13519.50 | 9345.59 | 7806.45 | 6912.94 | 6957.95 | 9227.56 |
| P35579 | Myosin-9 | **1.66** | **0.06** | 1190.60 | 342.45 | 1981.51 | 735.51 | 1269.21 | 808.88 | 1630.25 | 1361.69 | 882.95 | 1594.45 | 1520.78 | 2751.42 | 2944.76 | 2002.64 | 1075.01 |
| P21980 | Protein-glutamine gamma-glutamyltransferase 2 | **13.90** | **0.06** | 52.16 | 60.02 | 724.90 | 665.70 | 125.20 | 0.00 | 107.46 | 28.16 | 0.00 | 81.12 | 367.04 | 1476.98 | 863.17 | 1516.17 | 44.88 |
| P18669 | Phosphoglycerate mutase 1 | **0.61** | **0.06** | 2092.28 | 897.65 | 1268.43 | 219.75 | 2269.99 | 1025.02 | 1727.60 | 3472.04 | 1966.76 | 1420.17 | 1449.35 | 1401.06 | 863.46 | 1204.42 | 1272.11 |
| P21333 | Filamin-A | **3.51** | **0.06** | 506.32 | 331.41 | 1775.24 | 1258.30 | 642.04 | 248.84 | 1014.01 | 419.20 | 207.53 | 862.40 | 1276.03 | 3370.71 | 3124.39 | 1813.35 | 204.59 |
| P26641 | Elongation factor 1-gamma | **1.90** | **0.06** | 716.20 | 501.32 | 1361.49 | 476.76 | 522.59 | 69.67 | 1001.94 | 1389.92 | 596.85 | 738.81 | 2081.87 | 1004.97 | 1347.59 | 1678.61 | 1317.10 |
| P63244 | Guanine nucleotide-binding protein subunit beta-2-like 1 | **1.83** | **0.06** | 601.03 | 461.39 | 1098.55 | 290.01 | 485.00 | 36.17 | 876.37 | 1228.61 | 378.99 | 1032.11 | 1134.34 | 664.58 | 1287.22 | 960.10 | 1512.96 |
| P52565 | Rho GDP-dissociation inhibitor 1 | **1.39** | **0.06** | 850.04 | 287.86 | 1178.73 | 212.50 | 806.63 | 724.83 | 1120.93 | 1141.90 | 455.93 | 975.66 | 1493.56 | 984.41 | 1377.07 | 1149.22 | 1092.43 |
| P00918 | Carbonic anhydrase 2 | **0.24** | **0.06** | 810.61 | 683.03 | 191.31 | 154.05 | 731.45 | 225.37 | 753.37 | 379.67 | 1963.16 | 189.58 | 225.73 | 0.00 | 461.07 | 166.74 | 104.72 |
| O43175 | D-3-phosphoglycerate dehydrogenase | **42.52** | **0.06** | 13.28 | 12.81 | 564.51 | 551.92 | 17.31 | 0.00 | 0.00 | 28.63 | 20.45 | 666.58 | 697.46 | 0.00 | 0.00 | 532.96 | 1490.07 |
| O00515 | Ladinin-1 | **0.24** | **0.06** | 108.79 | 86.17 | 25.81 | 34.58 | 65.90 | 0.00 | 113.12 | 233.21 | 131.70 | 85.37 | 0.00 | 23.04 | 0.00 | 0.00 | 46.45 |
| P26373 | 60S ribosomal protein L13 | **1.52** | **0.06** | 688.80 | 350.63 | 1045.70 | 185.16 | 524.68 | 269.44 | 986.45 | 1114.13 | 549.30 | 1042.59 | 1187.38 | 1138.34 | 689.02 | 1046.53 | 1170.36 |
| P52434 | DNA-directed RNA polymerases I, II, and III subunit RPABC3 | **18.06** | **0.06** | 6.34 | 14.19 | 114.61 | 110.15 | 0.00 | 0.00 | 0.00 | 31.72 | 0.00 | 159.52 | 122.37 | 0.00 | 0.00 | 111.44 | 294.36 |
| P36957 | Dihydrolipoyllysine-residue succinyltransferase component of 2-oxoglutarate dehydrogenase complex, mitochondrial | **0.51** | **0.06** | 261.39 | 127.85 | 132.14 | 66.56 | 192.53 | 83.24 | 401.32 | 272.55 | 357.31 | 224.55 | 178.24 | 37.19 | 151.09 | 109.72 | 92.02 |
| P68363 | Tubulin alpha-1B chain | **1.23** | **0.06** | 3189.31 | 668.91 | 3916.74 | 455.62 | 3144.62 | 2930.67 | 3579.70 | 4030.29 | 2261.29 | 3199.82 | 3809.83 | 4106.16 | 4037.80 | 4579.76 | 3767.04 |
| Q9NR45 | Sialic acid synthase | **5.65** | **0.06** | 24.61 | 24.94 | 138.95 | 117.20 | 0.00 | 0.00 | 56.49 | 25.85 | 40.72 | 0.00 | 219.64 | 0.00 | 280.39 | 135.20 | 198.45 |
| P43490 | Nicotinamide phosphoribosyltransferase | **4.51** | **0.06** | 173.53 | 221.94 | 782.25 | 615.10 | 217.17 | 0.00 | 0.00 | 536.03 | 114.47 | 69.14 | 1010.42 | 1630.95 | 473.85 | 247.52 | 1261.62 |
| Q9C005 | Protein dpy-30 homolog | **0.30** | **0.06** | 422.96 | 305.74 | 127.21 | 140.84 | 774.98 | 0.00 | 619.55 | 470.95 | 249.35 | 0.00 | 291.36 | 0.00 | 0.00 | 231.69 | 240.23 |
| Q01469 | Fatty acid-binding protein, epidermal | **0.40** | **0.06** | 2105.95 | 1094.78 | 849.22 | 161.40 | 3380.52 | 2268.00 | 2888.16 | 1183.53 | 809.54 | 904.45 | 1076.88 | 798.50 | 601.46 | 932.19 | 781.87 |
| P48444 | Coatomer subunit delta | **2.36** | **0.06** | 125.26 | 122.11 | 295.74 | 141.11 | 67.86 | 0.00 | 83.21 | 320.21 | 155.00 | 193.47 | 188.47 | 441.68 | 507.95 | 232.03 | 210.83 |
| Q15907 | Ras-related protein Rab-11B | **1.52** | **0.06** | 844.04 | 337.17 | 1286.39 | 349.29 | 947.75 | 312.88 | 863.26 | 1245.83 | 850.47 | 1052.76 | 1253.46 | 1376.94 | 1013.46 | 1080.22 | 1941.50 |
| O60814 | Histone H2B type 1-K | **1.51** | **0.06** | 6328.00 | 2180.68 | 9580.06 | 2778.75 | 4786.73 | 5005.92 | 7998.68 | 9311.34 | 4537.31 | 14543.33 | 9951.54 | 8092.34 | 6878.14 | 7653.61 | 10361.39 |
| P24821 | Tenascin | **642.04** | **0.06** | 1.01 | 2.25 | 645.26 | 664.84 | 0.00 | 0.00 | 5.03 | 0.00 | 0.00 | 156.28 | 592.66 | 1335.39 | 1583.59 | 203.62 | 0.00 |
| P62244 | 40S ribosomal protein S15a | **1.90** | **0.06** | 432.43 | 343.21 | 820.23 | 268.25 | 206.71 | 0.00 | 811.07 | 731.57 | 412.78 | 1232.27 | 956.87 | 588.63 | 488.62 | 765.71 | 889.26 |
| P28066 | Proteasome subunit alpha type-5 | **1.75** | **0.06** | 391.70 | 240.69 | 686.19 | 221.54 | 604.88 | 0.00 | 410.77 | 570.86 | 371.98 | 482.39 | 804.10 | 681.41 | 366.62 | 848.52 | 934.10 |
| P62906 | 60S ribosomal protein L10a | **1.90** | **0.07** | 584.96 | 476.92 | 1109.17 | 352.87 | 413.92 | 38.23 | 885.87 | 1242.95 | 343.81 | 1170.36 | 1439.93 | 918.45 | 592.98 | 986.44 | 1546.83 |
| P50570 | Dynamin-2 | **0.43** | **0.07** | 268.08 | 172.64 | 115.34 | 44.56 | 190.85 | 33.05 | 330.37 | 286.07 | 500.05 | 81.47 | 180.15 | 54.75 | 106.15 | 140.75 | 128.79 |
| P68871 | Hemoglobin subunit beta | **0.37** | **0.07** | 64455.92 | 46919.93 | 24081.64 | 6724.47 | 64178.92 | 55360.35 | 40501.03 | 19500.21 | 142739.12 | 29841.34 | 20005.19 | 24616.83 | 31428.35 | 25475.37 | 13122.74 |
| P36955 | Pigment epithelium-derived factor | **6.70** | **0.07** | 64.23 | 120.03 | 430.19 | 380.40 | 0.00 | 276.10 | 45.06 | 0.00 | 0.00 | 79.61 | 252.83 | 499.92 | 968.29 | 758.70 | 21.78 |
| Q99715 | Collagen alpha-1(XII) chain | **14.97** | **0.07** | 44.68 | 50.82 | 668.92 | 650.75 | 20.60 | 42.31 | 131.34 | 29.16 | 0.00 | 64.07 | 326.32 | 754.16 | 1374.18 | 1494.77 | 0.00 |
| P59998 | Actin-related protein 2/3 complex subunit 4 | **1.50** | **0.07** | 792.44 | 348.76 | 1187.85 | 215.28 | 1104.02 | 443.70 | 988.79 | 1041.93 | 383.75 | 1094.83 | 1007.29 | 1484.03 | 1437.21 | 1094.21 | 1009.54 |
| Q99426 | Tubulin-folding cofactor B | **0.29** | **0.07** | 43.92 | 29.48 | 12.87 | 19.95 | 38.76 | 0.00 | 38.42 | 71.41 | 71.01 | 39.77 | 0.00 | 0.00 | 0.00 | 0.00 | 37.46 |
| Q9Y5K6 | CD2-associated protein | **0.15** | **0.07** | 88.63 | 86.66 | 13.24 | 20.78 | 72.49 | 0.00 | 55.09 | 232.73 | 82.83 | 45.01 | 34.43 | 0.00 | 0.00 | 0.00 | 0.00 |
| Q5BJF2 | Transmembrane protein 97 | **0.14** | **0.07** | 315.21 | 245.65 | 42.74 | 104.69 | 558.21 | 414.22 | 492.62 | 111.01 | 0.00 | 0.00 | 0.00 | 0.00 | 0.00 | 256.44 | 0.00 |
| P15121 | Aldose reductase | **2.43** | **0.07** | 280.54 | 366.32 | 682.80 | 279.47 | 152.53 | 0.00 | 119.70 | 921.39 | 209.07 | 790.77 | 828.53 | 794.31 | 239.45 | 452.06 | 991.66 |
| P37837 | Transaldolase | **1.71** | **0.07** | 536.35 | 162.32 | 919.48 | 396.31 | 517.48 | 303.71 | 749.33 | 604.66 | 506.58 | 459.86 | 1399.23 | 1363.31 | 698.98 | 983.02 | 612.49 |
| P25786 | Proteasome subunit alpha type-1 | **2.03** | **0.07** | 123.74 | 122.98 | 250.63 | 80.76 | 168.14 | 0.00 | 164.92 | 285.63 | 0.00 | 278.91 | 332.08 | 273.60 | 99.89 | 229.95 | 289.33 |
| P06396 | Gelsolin | **1.61** | **0.07** | 672.03 | 219.22 | 1081.55 | 395.39 | 638.47 | 1041.49 | 552.61 | 655.73 | 471.83 | 525.66 | 904.03 | 1161.14 | 1627.59 | 1391.22 | 879.65 |
| P61604 | 10 kDa heat shock protein, mitochondrial | **0.54** | **0.07** | 1822.98 | 530.38 | 984.81 | 773.53 | 2252.91 | 2510.80 | 1551.65 | 1266.67 | 1532.87 | 2136.93 | 1065.06 | 311.74 | 0.00 | 928.78 | 1466.38 |
| P23396 | 40S ribosomal protein S3 | **1.47** | **0.07** | 1215.85 | 533.12 | 1791.09 | 154.34 | 825.08 | 635.56 | 1483.79 | 1972.64 | 1162.19 | 1767.83 | 1935.11 | 1969.09 | 1776.95 | 1535.99 | 1761.57 |
| P25815 | Protein S100-P | **0.32** | **0.07** | 17332.02 | 10844.16 | 5611.92 | 3244.52 | 32334.98 | 23837.87 | 15664.48 | 8705.59 | 6117.18 | 4524.62 | 11631.45 | 5648.88 | 2519.54 | 3278.48 | 6068.55 |
| Q96C12 | Armadillo repeat-containing protein 5 | **15.77** | **0.07** | 3.09 | 6.91 | 48.71 | 49.33 | 0.00 | 0.00 | 15.45 | 0.00 | 0.00 | 118.12 | 18.54 | 0.00 | 78.22 | 77.40 | 0.00 |
| P35609 | Alpha-actinin-2 | **6.86** | **0.07** | 69.22 | 154.79 | 474.56 | 420.68 | 346.12 | 0.00 | 0.00 | 0.00 | 0.00 | 0.00 | 668.27 | 1097.54 | 545.44 | 536.12 | 0.00 |
| Q9Y490 | Talin-1 | **4.82** | **0.07** | 129.73 | 101.38 | 625.34 | 534.75 | 195.59 | 0.00 | 261.25 | 82.48 | 109.31 | 194.15 | 498.28 | 1476.19 | 1049.26 | 450.60 | 83.54 |
| Q7L1Q6 | Basic leucine zipper and W2 domain-containing protein 1 | **4.95** | **0.07** | 18.58 | 34.93 | 91.96 | 74.21 | 12.61 | 0.00 | 0.00 | 80.29 | 0.00 | 44.10 | 210.06 | 102.56 | 134.03 | 0.00 | 60.99 |
| P07858 | Cathepsin B | **1.73** | **0.07** | 494.49 | 231.90 | 856.26 | 337.63 | 697.69 | 252.95 | 358.49 | 783.87 | 379.44 | 585.56 | 1394.38 | 540.61 | 994.76 | 605.88 | 1016.36 |
| P31949 | Protein S100-A11 | **0.71** | **0.07** | 7014.30 | 1697.83 | 4956.21 | 1674.26 | 9783.50 | 6826.91 | 5193.60 | 6300.34 | 6967.13 | 5608.52 | 6890.84 | 2715.70 | 3174.18 | 6231.44 | 5116.57 |
| P10620 | Microsomal glutathione S-transferase 1 | **0.41** | **0.07** | 776.84 | 425.72 | 314.79 | 334.42 | 1015.08 | 147.95 | 896.15 | 1245.39 | 579.60 | 631.43 | 375.91 | 0.00 | 0.00 | 105.05 | 776.32 |
| Q9NX63 | Coiled-coil-helix-coiled-coil-helix domain-containing protein 3, mitochondrial | **4.30** | **0.08** | 13.12 | 17.98 | 56.40 | 44.82 | 0.00 | 0.00 | 33.30 | 32.33 | 0.00 | 72.69 | 41.14 | 51.69 | 0.00 | 38.80 | 134.06 |
| P02794 | Ferritin heavy chain | **5.02** | **0.08** | 91.52 | 75.84 | 459.76 | 399.91 | 114.14 | 137.82 | 26.27 | 0.00 | 179.38 | 529.44 | 581.88 | 447.31 | 1114.26 | 85.67 | 0.00 |
| P39656 | Dolichyl-diphosphooligosaccharide--protein glycosyltransferase 48 kDa subunit | **1.45** | **0.08** | 432.83 | 121.80 | 625.49 | 181.80 | 390.24 | 248.47 | 446.59 | 521.75 | 557.11 | 842.88 | 562.00 | 740.83 | 666.21 | 630.11 | 310.89 |
| P18859 | ATP synthase-coupling factor 6, mitochondrial | **0.08** | **0.08** | 275.95 | 236.48 | 23.08 | 35.80 | 640.15 | 328.70 | 279.08 | 62.81 | 69.01 | 72.05 | 0.00 | 0.00 | 0.00 | 0.00 | 66.44 |
| Q8NFU3 | Thiosulfate sulfurtransferase/rhodanese-like domain-containing protein 1 | **0.07** | **0.08** | 656.41 | 577.58 | 42.90 | 67.40 | 1286.10 | 1273.10 | 353.46 | 82.76 | 286.65 | 0.00 | 110.90 | 0.00 | 0.00 | 146.48 | 0.00 |
| P11413 | Glucose-6-phosphate 1-dehydrogenase | **31.46** | **0.08** | 9.60 | 21.46 | 301.91 | 324.25 | 0.00 | 0.00 | 0.00 | 47.98 | 0.00 | 44.29 | 941.51 | 248.22 | 267.10 | 115.91 | 194.44 |
| Q9NQR4 | Omega-amidase NIT2 | **0.20** | **0.08** | 139.82 | 106.07 | 27.91 | 46.90 | 252.56 | 0.00 | 198.19 | 190.68 | 57.64 | 24.94 | 0.00 | 0.00 | 0.00 | 21.80 | 120.72 |
| Q14847 | LIM and SH3 domain protein 1 | **0.32** | **0.08** | 547.94 | 351.45 | 176.62 | 155.49 | 842.91 | 757.51 | 241.16 | 95.87 | 802.24 | 291.30 | 0.00 | 0.00 | 275.21 | 360.27 | 132.97 |
| P20810 | Calpastatin | **0.20** | **0.08** | 266.27 | 254.29 | 54.20 | 61.28 | 704.67 | 44.34 | 225.05 | 170.13 | 187.16 | 118.93 | 80.92 | 0.00 | 0.00 | 0.00 | 125.33 |
| O14979 | Heterogeneous nuclear ribonucleoprotein D-like | **0.53** | **0.08** | 566.44 | 168.73 | 297.90 | 270.76 | 394.63 | 390.83 | 663.60 | 771.47 | 611.67 | 555.25 | 173.97 | 0.00 | 0.00 | 546.17 | 511.99 |
| P27816 | Microtubule-associated protein 4 | **0.37** | **0.08** | 143.13 | 83.64 | 53.37 | 66.47 | 284.35 | 151.64 | 75.10 | 103.21 | 101.34 | 34.01 | 0.00 | 0.00 | 172.34 | 87.26 | 26.58 |
| P02786 | Transferrin receptor protein 1 | **11.25** | **0.08** | 26.12 | 42.29 | 293.87 | 297.37 | 33.38 | 0.00 | 0.00 | 97.22 | 0.00 | 201.74 | 494.52 | 111.58 | 0.00 | 156.55 | 798.80 |
| P11678 | Eosinophil peroxidase | **4.50** | **0.08** | 121.00 | 161.48 | 544.52 | 451.62 | 165.84 | 383.24 | 0.00 | 55.90 | 0.00 | 196.50 | 584.90 | 771.02 | 1288.05 | 405.05 | 21.60 |
| P60981 | Destrin | **1.43** | **0.08** | 518.63 | 210.75 | 740.24 | 161.02 | 590.50 | 321.08 | 851.82 | 393.38 | 436.38 | 828.27 | 514.53 | 678.25 | 711.59 | 712.63 | 996.19 |
| P50502 | Hsc70-interacting protein | **0.71** | **0.08** | 697.06 | 113.52 | 494.03 | 202.95 | 859.44 | 709.31 | 572.06 | 608.32 | 736.17 | 795.56 | 530.44 | 549.40 | 182.57 | 524.77 | 381.46 |
| P05783 | Keratin, type I cytoskeletal 18 | **0.55** | **0.08** | 1278.70 | 423.38 | 699.08 | 527.92 | 1260.60 | 1140.58 | 843.99 | 1982.83 | 1165.49 | 850.54 | 444.59 | 1497.73 | 50.15 | 1026.28 | 325.19 |
| Q13423 | NAD(P) transhydrogenase, mitochondrial | **0.07** | **0.08** | 46.22 | 41.30 | 3.27 | 8.00 | 25.11 | 0.00 | 38.00 | 109.69 | 58.29 | 19.60 | 0.00 | 0.00 | 0.00 | 0.00 | 0.00 |
| P01903 | HLA class II histocompatibility antigen, DR alpha chain | **5.06** | **0.08** | 168.42 | 247.08 | 852.15 | 759.78 | 295.65 | 546.46 | 0.00 | 0.00 | 0.00 | 206.30 | 1751.74 | 1828.08 | 745.44 | 471.76 | 109.57 |
| P40616 | ADP-ribosylation factor-like protein 1 | **3.93** | **0.08** | 45.28 | 50.98 | 177.78 | 142.37 | 101.30 | 0.00 | 26.75 | 98.32 | 0.00 | 110.87 | 462.07 | 155.60 | 78.78 | 105.99 | 153.36 |
| P62826 | GTP-binding nuclear protein Ran | **1.63** | **0.08** | 1124.11 | 594.05 | 1835.80 | 603.06 | 1315.68 | 361.95 | 1382.76 | 1862.52 | 697.65 | 2094.76 | 1508.06 | 1223.35 | 1554.99 | 1713.80 | 2919.81 |
| P40121 | Macrophage-capping protein | **1.56** | **0.08** | 950.67 | 416.99 | 1480.27 | 467.16 | 1373.86 | 1411.04 | 816.76 | 628.65 | 523.03 | 1208.54 | 1901.45 | 1558.10 | 2033.80 | 1423.54 | 756.19 |
| P63104 | 14-3-3 protein zeta/delta | **1.12** | **0.08** | 2579.39 | 362.75 | 2896.47 | 150.97 | 2289.26 | 3163.22 | 2291.89 | 2672.79 | 2479.78 | 3009.56 | 2913.25 | 3033.81 | 2677.77 | 2743.74 | 3000.71 |
| P49915 | GMP synthase [glutamine-hydrolyzing] | **26.33** | **0.08** | 1.75 | 3.91 | 45.99 | 50.00 | 8.73 | 0.00 | 0.00 | 0.00 | 0.00 | 39.72 | 31.59 | 0.00 | 0.00 | 131.25 | 73.39 |
| P61026 | Ras-related protein Rab-10 | **1.55** | **0.08** | 684.83 | 429.63 | 1060.33 | 182.19 | 645.69 | 0.00 | 1161.17 | 880.20 | 737.07 | 1338.83 | 1151.27 | 991.89 | 897.13 | 851.21 | 1131.65 |
| P08865 | 40S ribosomal protein SA | **1.22** | **0.08** | 1453.72 | 296.30 | 1769.74 | 242.75 | 1852.78 | 1116.93 | 1422.34 | 1634.53 | 1242.03 | 1670.32 | 1541.28 | 1638.49 | 2221.52 | 1698.58 | 1848.23 |
| O00584 | Ribonuclease T2 | **0.17** | **0.08** | 375.80 | 389.99 | 63.27 | 73.10 | 436.27 | 336.06 | 0.00 | 999.92 | 106.76 | 96.49 | 192.33 | 0.00 | 0.00 | 32.84 | 57.98 |
| Q6NVY1 | 3-hydroxyisobutyryl-CoA hydrolase, mitochondrial | **0.16** | **0.08** | 177.54 | 145.48 | 29.27 | 45.36 | 280.01 | 0.00 | 228.89 | 330.33 | 48.48 | 0.00 | 86.41 | 0.00 | 0.00 | 0.00 | 89.23 |
| Q15436 | Protein transport protein Sec23A | **4.10** | **0.09** | 37.67 | 42.35 | 154.26 | 128.03 | 30.12 | 0.00 | 0.00 | 99.66 | 58.57 | 46.90 | 167.59 | 204.26 | 380.35 | 68.77 | 57.68 |
| P50454 | Serpin H1 | **3.82** | **0.09** | 408.90 | 244.69 | 1560.50 | 1314.84 | 301.85 | 166.08 | 740.24 | 590.10 | 246.25 | 536.48 | 585.53 | 2876.87 | 3261.79 | 1916.82 | 185.51 |
| O15400 | Syntaxin-7 | **0.45** | **0.09** | 151.87 | 91.45 | 67.98 | 50.15 | 173.49 | 267.27 | 41.06 | 79.43 | 198.10 | 134.88 | 105.52 | 88.73 | 39.23 | 0.00 | 39.50 |
| A6NIZ1 | Ras-related protein Rap-1b-like protein | **16.13** | **0.09** | 13.91 | 31.11 | 224.37 | 240.24 | 0.00 | 69.57 | 0.00 | 0.00 | 0.00 | 154.96 | 0.00 | 320.91 | 229.20 | 641.18 | 0.00 |
| P18085 | ADP-ribosylation factor 4 | **1.75** | **0.09** | 931.63 | 612.48 | 1626.85 | 582.27 | 1158.02 | 0.00 | 1004.86 | 1681.37 | 813.89 | 1246.76 | 1759.33 | 1521.95 | 2737.32 | 1218.35 | 1277.36 |
| P29692 | Elongation factor 1-delta | **1.63** | **0.09** | 445.54 | 277.73 | 724.14 | 202.12 | 342.72 | 239.21 | 550.12 | 882.13 | 213.50 | 775.24 | 346.14 | 760.47 | 687.38 | 852.27 | 923.34 |
| Q04695 | Keratin, type I cytoskeletal 17 | **0.63** | **0.09** | 1936.80 | 690.63 | 1228.66 | 531.31 | 1311.36 | 1285.63 | 1987.99 | 2137.68 | 2961.36 | 1344.11 | 1195.66 | 2230.83 | 868.88 | 849.24 | 883.25 |
| Q9BTT0 | Acidic leucine-rich nuclear phosphoprotein 32 family member E | **9.31** | **0.09** | 26.19 | 35.86 | 243.69 | 251.46 | 0.00 | 0.00 | 66.30 | 64.64 | 0.00 | 0.00 | 517.86 | 102.65 | 81.01 | 159.02 | 601.58 |
| O75223 | Gamma-glutamylcyclotransferase | **6.97** | **0.09** | 80.24 | 109.88 | 559.57 | 554.36 | 202.54 | 0.00 | 198.66 | 0.00 | 0.00 | 1175.91 | 300.02 | 49.54 | 0.00 | 554.04 | 1277.92 |
| Q14697 | Neutral alpha-glucosidase AB | **1.66** | **0.09** | 641.30 | 432.53 | 1066.23 | 304.00 | 366.71 | 173.83 | 899.28 | 1251.23 | 515.47 | 1082.07 | 1549.51 | 711.46 | 977.95 | 1253.90 | 822.49 |
| Q9Y6N5 | Sulfide:quinone oxidoreductase, mitochondrial | **30.82** | **0.09** | 6.97 | 15.58 | 214.75 | 241.32 | 34.84 | 0.00 | 0.00 | 0.00 | 0.00 | 0.00 | 365.74 | 113.44 | 137.33 | 38.86 | 633.15 |
| Q05925 | Homeobox protein engrailed-1 | **4.39** | **0.09** | 39.15 | 87.55 | 172.08 | 133.38 | 195.77 | 0.00 | 0.00 | 0.00 | 0.00 | 51.01 | 147.20 | 354.79 | 311.28 | 136.21 | 31.99 |
| Q15366 | Poly(rC)-binding protein 2 | **1.47** | **0.09** | 806.78 | 460.57 | 1185.92 | 168.74 | 848.91 | 0.00 | 1086.16 | 1045.00 | 1053.85 | 1147.97 | 1321.26 | 1441.41 | 988.79 | 1051.71 | 1164.39 |
| P62753 | 40S ribosomal protein S6 | **1.54** | **0.09** | 351.46 | 174.87 | 539.95 | 156.65 | 344.78 | 116.36 | 563.67 | 470.00 | 262.51 | 635.47 | 567.46 | 685.62 | 241.26 | 523.96 | 585.96 |
| Q7Z406 | Myosin-14 | **1.44** | **0.09** | 327.31 | 97.99 | 471.20 | 144.91 | 374.74 | 189.57 | 438.92 | 269.21 | 364.11 | 289.94 | 586.85 | 655.28 | 511.21 | 318.75 | 465.19 |
| Q8NBX0 | Saccharopine dehydrogenase-like oxidoreductase | **0.33** | **0.09** | 461.17 | 292.07 | 151.50 | 253.24 | 517.29 | 124.24 | 789.33 | 678.06 | 196.93 | 116.77 | 0.00 | 90.25 | 42.58 | 0.00 | 659.37 |
| Q16555 | Dihydropyrimidinase-related protein 2 | **5.88** | **0.09** | 70.28 | 131.11 | 413.33 | 399.82 | 32.42 | 0.00 | 303.59 | 15.37 | 0.00 | 0.00 | 169.80 | 783.39 | 948.35 | 529.13 | 49.30 |
| P51911 | Calponin-1 | **4.54** | **0.09** | 166.22 | 245.94 | 754.45 | 680.70 | 566.42 | 242.40 | 22.26 | 0.00 | 0.00 | 97.69 | 1553.76 | 1177.70 | 1338.90 | 358.66 | 0.00 |
| Q9ULV4 | Coronin-1C | **55.12** | **0.09** | 2.93 | 6.55 | 161.55 | 188.32 | 0.00 | 0.00 | 0.00 | 14.66 | 0.00 | 11.73 | 108.43 | 511.58 | 209.57 | 127.98 | 0.00 |
| P04062 | Glucosylceramidase | **0.08** | **0.09** | 32.07 | 30.16 | 2.62 | 6.43 | 44.44 | 0.00 | 0.00 | 64.59 | 51.30 | 0.00 | 15.74 | 0.00 | 0.00 | 0.00 | 0.00 |
| P02751 | Fibronectin | **5.23** | **0.09** | 311.36 | 365.39 | 1628.71 | 1554.48 | 147.95 | 208.93 | 961.44 | 104.72 | 133.77 | 594.36 | 730.50 | 4152.71 | 2797.71 | 1426.29 | 70.72 |
| P12110 | Collagen alpha-2(VI) chain | **2.31** | **0.09** | 574.98 | 544.98 | 1330.28 | 749.28 | 294.09 | 1341.80 | 954.21 | 181.48 | 103.35 | 1360.32 | 1424.18 | 2337.50 | 998.19 | 1753.34 | 108.13 |
| P11940 | Polyadenylate-binding protein 1 | **1.76** | **0.09** | 330.32 | 245.72 | 580.54 | 198.46 | 213.32 | 41.76 | 418.50 | 696.89 | 281.11 | 544.39 | 708.94 | 267.01 | 465.46 | 822.93 | 674.54 |
| P25788 | Proteasome subunit alpha type-3 | **1.76** | **0.10** | 153.94 | 129.09 | 270.62 | 76.69 | 204.86 | 0.00 | 301.43 | 225.57 | 37.82 | 389.39 | 208.90 | 333.35 | 269.03 | 194.58 | 228.49 |
| P99999 | Cytochrome c | **1.37** | **0.10** | 1367.29 | 373.06 | 1873.93 | 500.16 | 1362.19 | 883.53 | 1617.51 | 1826.87 | 1146.34 | 2229.88 | 2123.98 | 1399.93 | 1129.80 | 1961.17 | 2398.85 |
| P69905 | Hemoglobin subunit alpha | **0.40** | **0.10** | 69569.46 | 42597.11 | 28143.00 | 10978.74 | 60846.41 | 98138.32 | 41770.30 | 20813.97 | 126278.29 | 44760.09 | 19141.78 | 29558.46 | 35530.36 | 25137.01 | 14730.28 |
| Q05315 | Galectin-10 | **4.64** | **0.10** | 202.69 | 191.52 | 940.03 | 877.15 | 393.04 | 259.22 | 0.00 | 361.21 | 0.00 | 651.98 | 582.91 | 1396.46 | 2481.00 | 527.83 | 0.00 |
[truncated: 226,332 more chars]
